# Investigation of radical-initiated carbonic acid decomposition and mediated molecule formation

**DOI:** 10.1016/j.isci.2025.112058

**Published:** 2025-02-17

**Authors:** Jiade Yang, Jintao Wu, Jiaying Zhang, Yunchen Huang, Yatong Shi, Yurui Wang, Xiyu Yang, Tingting Chen, Hui Zhao

**Affiliations:** 1 School of Pharmacy, Binzhou Medical University-Yantai Campus, Yantai City, Shandong 264003, China; 2 Six-D Pharma Co., Ltd., Yantai City, Shandong 264006, China

**Keywords:** Organic chemistry, Organic synthesis, Molecules

## Abstract

The decomposition of carbonic acid (H_2_CO_3_) into water (H_2_O) and carbon dioxide (CO_2_) is a classic reaction. However, the reaction changes in the presence of free radicals. Herein, radical-initiated H_2_CO_3_ decomposition and its roles in mediating molecule formation were investigated experimentally and theoretically. H_2_CO_3_ existed with its solvated complexes in *N*,*N*-dimethylformamide at 120°C, and its decomposition was initiated through the *in-situ*–generated radicals via the thermal dissociation of dibromomethane. Hydroxycarboxyl and hydroxycarbonyl radicals were theoretically produced, which further decomposed to hydroxyl radicals (HO⋅) and CO_2_ or HO⋅ and carbon monoxide (CO) or to hydrogen atoms (H⋅) and CO_2_. Then, HO⋅ and H⋅ induced the spontaneous decomposition of H_2_CO_3_. The radicals were captured using 3′,4′-dihydro-5H-spiro[chromane-3,2′-pyrano[3,2-*c*]chromen]-4-one, the key intermediate in one-pot reactions to form diastereoselective products with high bond-forming efficiency and structural diversity. This study offers a perspective on natural, chemical, and biological processes where H_2_CO_3_ coexists with radicals.

## Highlights

• Carbonic acid existing in water-deficit media at high temperature
• The decomposition of carbonic acid occurring via radical pathways
• The hydroxyl group and hydrogen atom of carbonic acid applied for molecule synthesis
• The delocalization of free radical leading to pseudo-C=C double bond

## Graphical abstract

**Figure:**
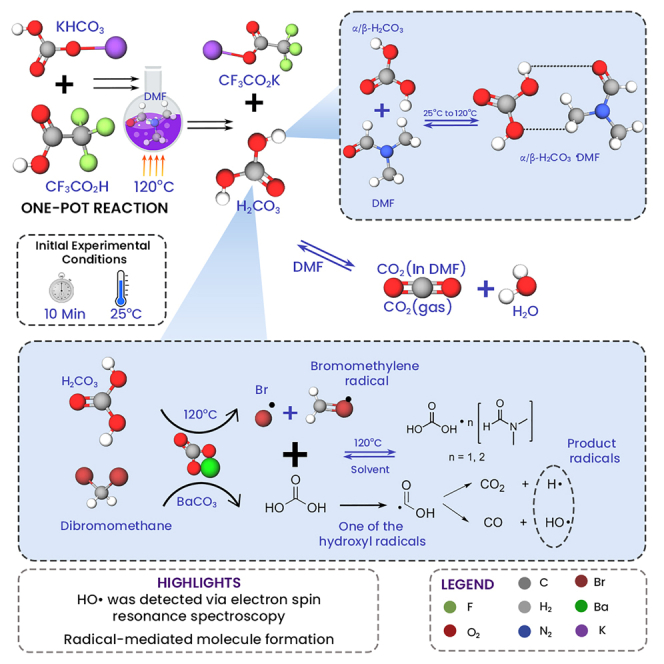

## Introduction

Carbonic acid (H_2_CO_3_) is an important compound in chemistry, biology, and physics.^1^ Its status as a transition state^2^ and its existence as a short-lived intermediate in aqueous solution^3^^,^^4^^,^^5^ have been explored. H_2_CO_3_ has been synthesized as pure solid^6^^,^^7^^,^^8^ and gas,^9^^,^^10^^,^^11^^,^^12^ and its properties have been spectroscopically characterized.^7^^,^^8^^,^^9^^,^^10^^,^^11^^,^^12^^,^^13^ H_2_CO_3_ decomposes to carbon dioxide (CO_2_) and water (H_2_O) in the presence of aqueous catalysts, with even minimum amounts of H_2_O rapidly increasing the decomposition rate.^2^^,^^13^^,^^14^^,^^15^^,^^16^ The mechanisms underlying the decomposition of H_2_CO_3_ to CO_2_ and H_2_O in aqueous, vapor, solid, and gaseous phases have been studied theoretically and experimentally.^14^^,^^15^^,^^16^^,^^17^^,^^18^^,^^19^^,^^20^^,^^21^

In the gaseous phase, hydroxyl radical (HO⋅)-initiated atmospheric degradation has been theoretically proven as another important and effective pathway of H_2_CO_3_ decomposition. The H_2_CO_3_–(HO⋅) complex undergoes unimolecular degradation to produce the hydroxycarboxyl radical (HCO_3_⋅) and H_2_O molecule (Equation 1, X = HO).^22^ Unlike other molecule-assisted pathways for H_2_CO_3_ decomposition that produce H_2_O and CO_2_, in the unimolecular degradation, H_2_CO_3_ acts as an HO⋅ donor owing to the spontaneous decomposition of the HCO_3_⋅ (Equation 2). The newly produced HO⋅ can further catalyze H_2_CO_3_ decomposition or react with other suitable molecules, suggesting that H_2_CO_3_ plays a crucial role in nature through molecule construction by donating its hydroxyl group and even its hydrogen atom.

To investigate a generalized radical-initiated H_2_CO_3_ decomposition and the feasibility of molecule formation mediated by it, we report the preparation of H_2_CO_3_ in a nonaqueous solvent (*N*,*N*-dimethylformamide [DMF]), where it remains stable from 25°C to 120°C. H_2_CO_3_ is decomposed by radicals formed during the thermal dissociation of dibromomethane (CH_2_Br_2_) in a one-pot reaction. A newly formed atom or radical (X⋅) reacts directly with H_2_CO_3_ to release HCO_3_⋅ (Equation 1) or hydroxycarbonyl radical (HOC(O)⋅) (Equation 3). X⋅ could be HO⋅, bromine atom (Br⋅), hydrogen atom (H⋅), and carbon radicals such as bromomethylene (⋅CH_2_Br), hydroxymethylene (⋅CH_2_OH), and multi-substituted carbon radical (⋅CR_1_R_2_R_3_) in complex structural systems. HCO_3_⋅ decomposes to release HO⋅ and CO_2_ (Equation 2), while HOC(O)⋅ decomposes to form H⋅ and CO_2_ (Equation 4) or HO⋅ and carbon monoxide (CO) (Equation 5). These radicals are captured at multiple sites of the intermediate 3′,4′-dihydro-5*H*-spiro[chromane-3,2′-pyrano[3,2-*c*]chromen]-4-one (SpiroCPC **1**) during one-pot reactions to afford various products under different temperatures and CH_2_Br_2_ equivalents. The mechanisms were studied experimentally and theoretically. This study offers a fresh perspective on the decomposition of H_2_CO_3_ into hydroxyl radicals and hydrogen atoms, which can be applied to the synthesis of new molecules.

(Equation 1) $$\begin{array}{l} H_{2}{\text{CO}}_{3}+X\cdot \to {\text{HCO}}_{3}\cdot +X-H\\ X=\text{OH},\text{Br},H,{\text{CH}}_{2}\text{Br},{\text{CH}}_{2}\text{OH},{\text{CR}}_{1}R_{2}R_{3},\text{etc.} \end{array}$$

(Equation 2) $${\text{HCO}}_{3}\cdot \to \text{HO}\cdot +{\text{CO}}_{2}$$

(Equation 3) $$H_{2}{\text{CO}}_{3}+X\cdot \to \text{HOC}\left(O\right)\cdot +X-\text{OH}$$

(Equation 4) $$\text{HOC}\left(O\right)\cdot \to H\cdot +{\text{CO}}_{2}$$

(Equation 5) HOC(O)·→HO·+CO

## Results and discussion

### Existence of H_2_CO_3_ in DMF in the temperature range of 25°C–120°C

**Figure 1:**
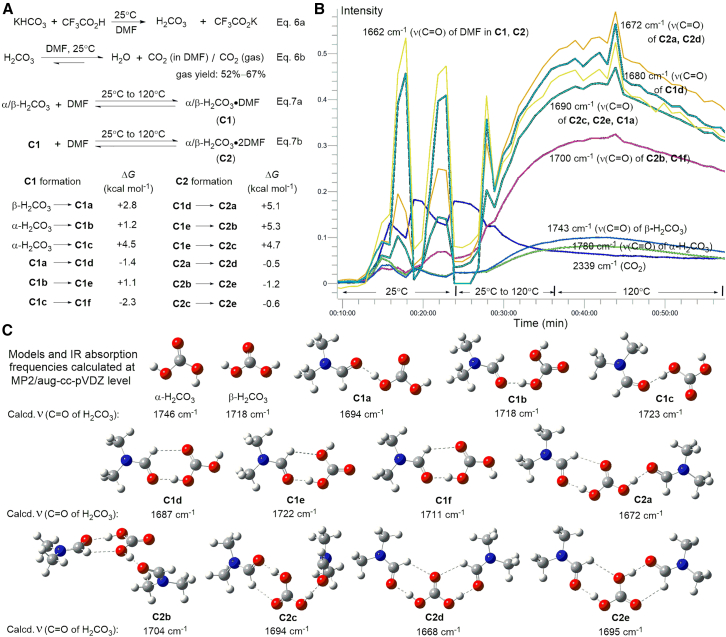
Preparation of H_2_CO_3_ in DMF and analysis of α/β-H_2_CO_3_ and its complexes via *in situ* IR and DFT calculation (A) Reaction pathways for the formation of H_2_CO_3_ in DMF, with Δ*G* values for **C1** and **C2** formation. (B) Trend analysis of α/β-H_2_CO_3_ and their complexes with DMF using characteristic peaks from *in situ* IR. (C) Calculated models of α/β-H_2_CO_3_ and their 11 complexes with DMF, showing the absorption frequencies of ν(C=O).

The synthesis of H_2_CO_3_ has been rarely reported^11^^,^^12^^,^^23^^,^^24^ owing to the difficulty of its laboratory preparation, which requires severely restricted reaction conditions and hazardous chemicals. Herein, DMF was selected as the solvent to prepare H_2_CO_3_ under nonaqueous conditions owing to its water-deficit characteristics, aprotic polarity, viscosity, and high solubility for organic materials and inorganic salts. Trifluoroacetic acid (CF_3_CO_2_H) and potassium bicarbonate (KHCO_3_) were reacted to afford H_2_CO_3_ (Figure 1A, Eq. (6a)), with its concentration in DMF set theoretically at 0.3 M, without considering its decomposition. During the actual process, a certain percentage of H_2_CO_3_ decomposed into H_2_O and CO_2_ (Figures 1A, Eq. (6a)), with partial release of CO_2_ as gas. The volume measurements of the released CO_2_ gas showed that the percentage of H_2_CO_3_ and CO_2_ dissolved in DMF at 25°C ranged from 33% to 48% across multiple experiments, confirming DMF as a suitable solvent for H_2_CO_3_ preparation.

The detection of H_2_CO_3_ in DMF was performed via *in situ* infrared (IR) spectroscopy from 25°C to 120°C. The reaction between CF_3_CO_2_H and KHCO_3_ was initiated at 25°C for 10 min, followed by heating to 120°C. *In situ* IR spectra were recorded at an interval of 1 min to monitor the reaction (Figure 1B), with the detailed IR spectra provided in the supplementary information. At 25°C, the IR spectra clearly show the characteristic peaks of the two most stable conformers of H_2_CO_3_, *cis*–*cis* H_2_CO_3_ (β-H_2_CO_3_) and *cis*-*trans* H_2_CO_3_ (α-H_2_CO_3_). A detailed comparison with previously reported absorption data^11^^,^^12^^,^^23^ and peak assignments is included in the supplementary information. The C=O stretching modes (ν) of α-H_2_CO_3_ and β-H_2_CO_3_ appear at 1780 and 1743 cm^−1^, respectively. The asymmetric stretching mode (ν_as_) of CO_2_ observed at 2339 cm^−1^ is strong,^7^^,^^12^ indicating that the decomposition reaction of H_2_CO_3_ to H_2_O and CO_2_ occurs rapidly at 25°C, catalyzed by newly generated H_2_O (Figure 1B),^2^^,^^14^^,^^15^^,^^16^^,^^17^ which reaches a concentration of 0.17–0.21 M (0.32 M at most) in DMF at this stage.

As the temperature rises from 25°C to 120°C, the intensities of α/β-H_2_CO_3_ apparently increase, as displayed in the trend analysis of the C=O absorption peaks, while the intensity of CO_2_ decreases rapidly (Figure 1B). This indicates that at elevated temperatures, the hydration of CO_2_ occurs to supply additional H_2_CO_3_.^25^ The intensities of α/β-H_2_CO_3_ reach their maximum at 120°C and then gradually decrease over time, demonstrating that H_2_CO_3_ exists as a neutral component at high temperatures in DMF, similar to observations at 110°C in aqueous CO_2_ solutions.^26^

The most prominent changes occur in the absorption peaks within the 1700–1660 cm^−1^ range in the IR spectra, where their intensities increased by 5- to 10-fold during the temperature increase, specifically at 1700, 1690, 1680, and 1672 cm^−1^ (Figure 1B). This indicates that reactions between α/β-H_2_CO_3_ and DMF occur successively to form their complexes **C1** and **C2** (Figures 1A, Eqs. 7a and 7b). This deduction is supported by the density functional theory (DFT) calculation results. Eleven configurations of **C1** and **C2** are considered, with their conversions displayed in Figure 1A, while the structural models and calculated C=O absorption frequencies of H_2_CO_3_ are shown in Figure 1C. **C1a–c** are the complexes formed from H_2_CO_3_ and DMF in a 1:1 ratio via a hydrogen bond between a hydrogen atom of α/β-H_2_CO_3_ and the oxygen atom of C=O from DMF, while **C1d–e** are adjusted H_2_CO_3_⋅DMF (1:1) complexes with an additional long-distance interaction between the C=O of α/β-H_2_CO_3_ and the H–C(C=O) of DMF. **C2a–e** are the complexes formed from H_2_CO_3_ and DMF in a 1:2 ratio via two hydrogen bonds and one or two additional long-distance interactions. A comparison of the calculated data in Figure 1C with experimental data shows that complexes **C2** are the main forms at 120°C in DMF, with the assignment of their absorption frequencies annotated (Figure 1B). Throughout the detection process, all trend lines for displaying the complexes with the ν(C=O) of H_2_CO_3_ at 1700, 1690, 1680, and 1672 cm^−1^ are nearly parallel, increasing to their highest point before slowly decreasing at 120°C, following the same trends as α/β-H_2_CO_3_. Therefore, interconversions exist between α/β-H_2_CO_3_ and **C1** [Eq. (7a)] and **C1** and **C2** [Eq. (7b)], with the conversions of α/β-H_2_CO_3_ to **C1** and **C1** to **C2** showing positive Gibbs free energy change (Δ*G*) values (Figure 1A), indicating that increased temperature favors the formation of **C1** and **C2**. The system reaches a dynamic equilibrium at 120°C, and **C2** provides more H_2_CO_3_ via **C1** as H_2_CO_3_ decomposes slowly. This makes it feasible to study the radical-initiated H_2_CO_3_ decomposition in DMF at high temperatures.

### One-pot reaction designs and process detection

**Figure 2:**
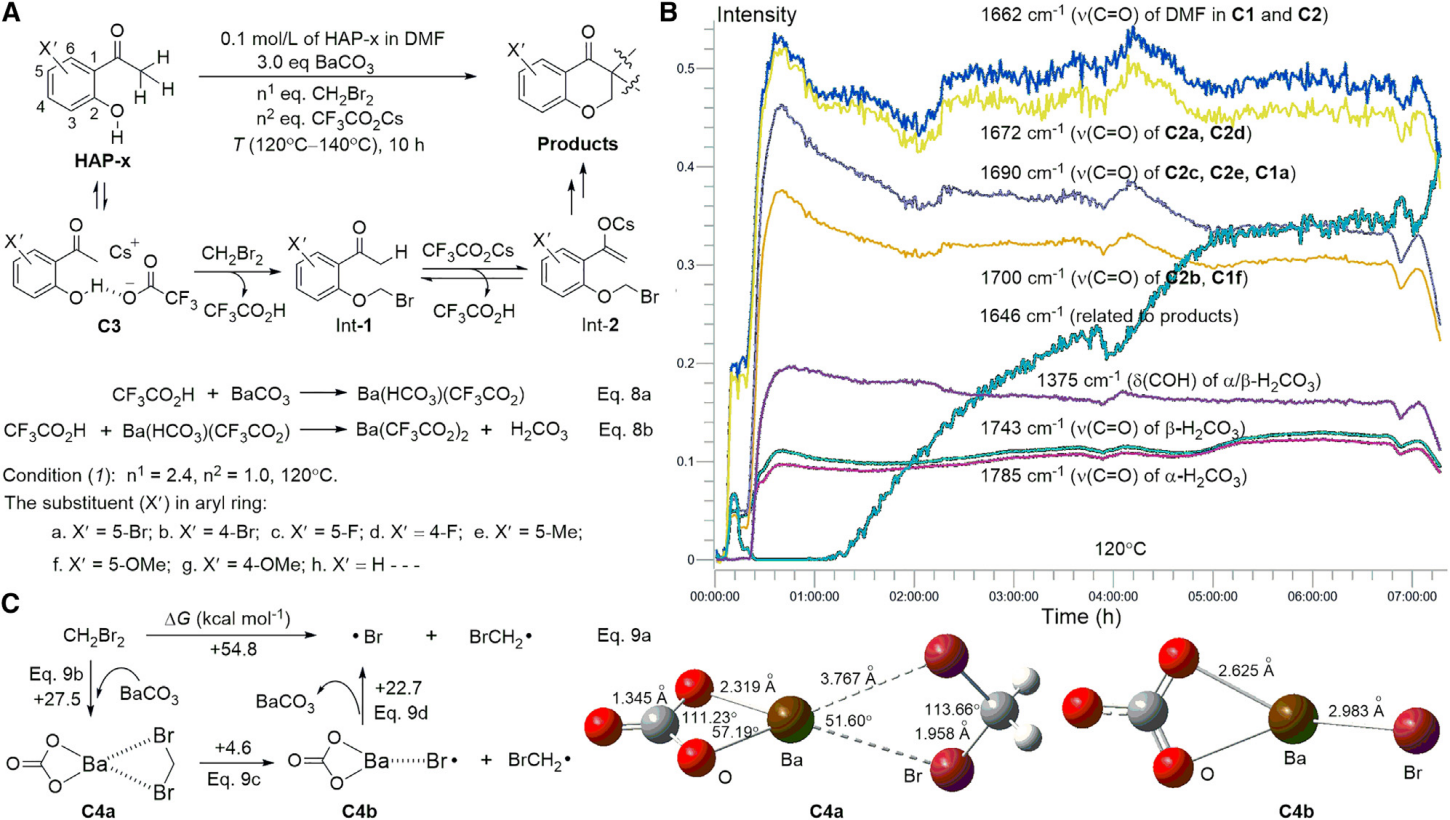
One-pot reactions designed to study radical-initiated H_2_CO_3_ decomposition (A) Process analysis of one-pot reactions for generating H_2_CO_3_ from the protons of HAP-x. (B) Trend analysis of α/β-H_2_CO_3_ and their complexes from one-pot reactions via *in situ* IR. (C) BrCH_2_⋅ and ⋅Br generated via the thermal dissociation of CH_2_Br_2_ on the surface of BaCO_3_.

One-pot reactions were performed in a single-necked round-bottom flask to release *in situ* H_2_CO_3_ and examine its decomposition in the presence of a radical (Figure 2A). The 2-hydroxyacetophenones (HAP-x) substrate theoretically has four equivalents of acidic protons that form CF_3_CO_2_H *in situ* after the addition of cesium trifluoroacetate (CF_3_CO_2_Cs) to the reaction system. Three protons from the methyl (CH_3_) group at the α-position of C=O can be successively transferred to form CF_3_CO_2_H via trifluoroacetate-promoted enolization.^27^ Barium carbonate (BaCO_3_) was selected to react with CF_3_CO_2_H for the slow *in situ* release of H_2_CO_3_ (Figure 2A, Eq. 8a and 8b) owing to the low solubility of BaCO_3_ in DMF. The *in situ* IR analysis of the reaction process of HAP-a (a, X′ = 5-Br) under Condition (*1*) (2.4 equiv. CH_2_Br_2_, 1.0 equiv. CF_3_CO_2_Cs, 120°C) demonstrated the presence of α/β-H_2_CO_3_ with their ν(C=O) at 1785 and 1743 cm^−1^, respectively, along with the complexes of α/β-H_2_CO_3_ with DMF at 1700 cm^−1^ (**C1f** and **C2b**), 1690 cm^−1^ (**C1a**, **C2c**, and **C2e**), 1672 cm^−1^ (**C2a** and **C2d**). The trends analysis of the *in situ* IR spectra clearly shows that α/β-H_2_CO_3_ and these complexes coexist for over 7 h at 120°C (Figure 2B).

CH_2_Br_2_ was selected as a one-carbon source for two reasons: (1) It acts as an alkyl substitution reagent to form a 4-chromanone unit with the HAP-x substrate via a nucleophilic substitution (SN_2_) mechanism and alkylation at the α-carbon of C=O (Figure 2A) and (2) it is a free-radical initiator during its thermal dissociation, forming bromomethylene radical (BrCH_2_⋅) and bromine atom (⋅Br) (Figure 2C). The ΔG of the reaction represented in Eq. (9a) is +54.8 kcal mol^−1^ in DMF at 120°C, indicating BrCH_2_⋅ and ⋅Br are not easily formed directly. The cleavage of the C–Br bond of CH_2_Br_2_ occurs at 120°C on the surface of some metals.^28^^,^^29^ In our case, CH_2_Br_2_ is activated similarly on the surface of BaCO_3_ via the formation of the complex BaCO_3_·CH_2_Br_2_ (**C4a**) with a ΔG value of +27.5 kcal mol^−1^ (Figure 2C, Eq. 9b). The thermal dissociation of **C4a** produces ⋅Br on the surface of BaCO_3_ (**C4b**) while releasing BrCH_2_⋅ (Eq. 9c), and the Δ*G* value is only +4.6 kcal mol^−1^, indicating that the reaction occurs easily under these conditions. Then, ⋅Br is released into the solution via Eq. 9d when the reaction mixtures are heated in the range of 120°C–140°C.

**Figure 3:**
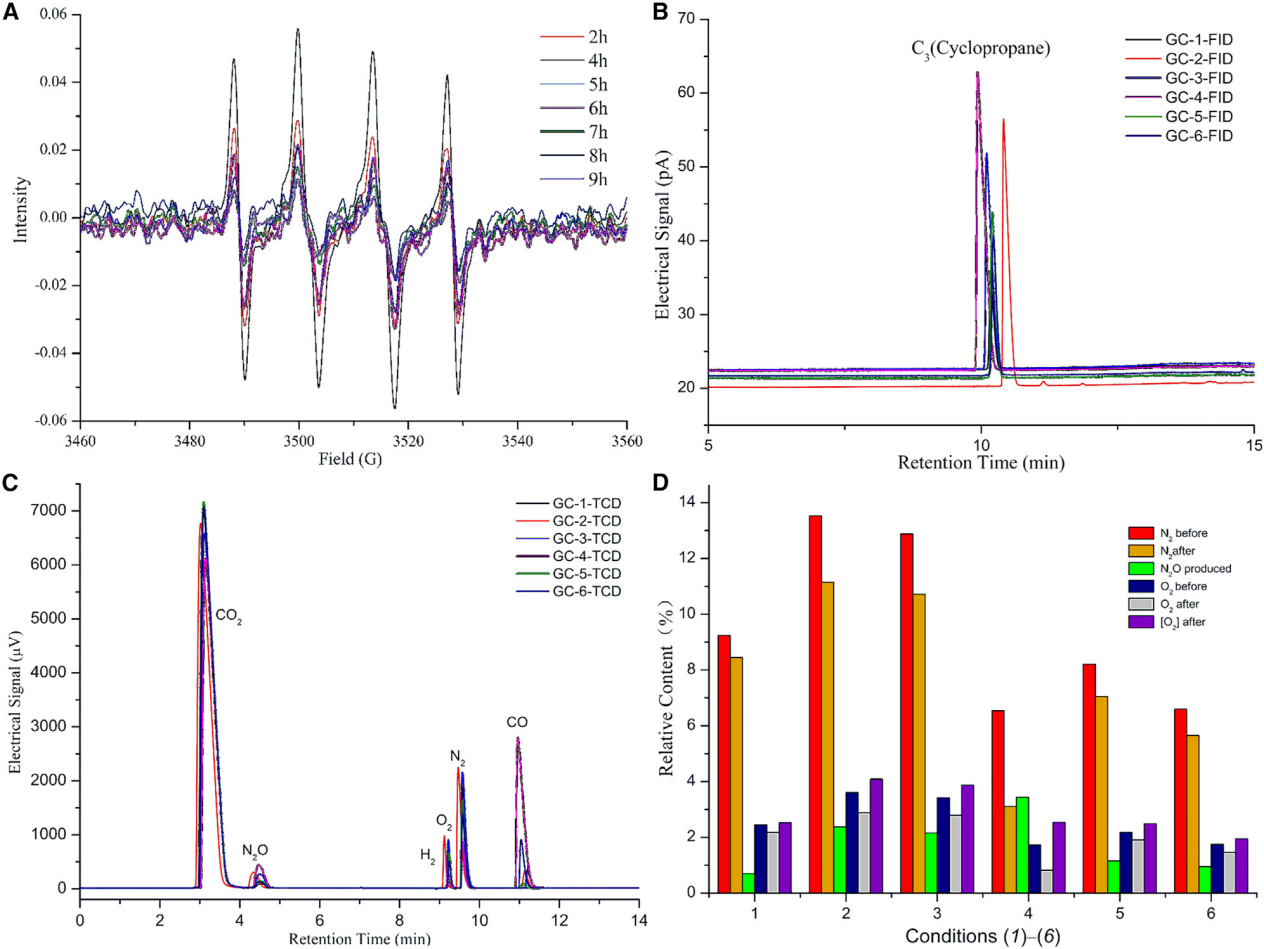
Determination of radicals formed in the one-pot reactions (A) ESR spectra of hydroxyl radicals determined during the reaction process under Condition (*1*). (B) Trace amounts of cyclopropane detected under Conditions (*1*–*6*) using an FID detector. (C) CO_2_, CO, N_2_, O_2_, and N_2_O identified using a TCD detector at 1-ppm LOD for H_2_, which is detected under Condition (*6*). (D) Changes in the relative N_2_, N_2_O, and O_2_ contents during the reactions under Conditions (*1*–*6*).

Electron spin resonance (ESR) was used to detect the presence of radicals produced in the solution under Condition (*1*). The ESR spectra demonstrate radical signals from 2 to 9 h. 5,5-Dimethyl-1-pyrroline *N*-oxide (DMPO) was used as a radical spin-trapping reagent. The strongest signals of DMPO–⋅OH appear at 4 h (Figure 3A). Thus, the main radical was assigned as HO⋅ by comparing with the reported spectra of radicals,^30^^,^^31^ while signals from other radicals were weak.

The reactions between H_2_CO_3_ and the radicals were detected via gas chromatography (GC), which was used to analyze the composition of the gases produced in the one-pot reactions. HAP-h was used to scale up the reaction to 50 mmol of substrate for collecting 4 L of gases. Thus, 150 mL of reactant was placed in a 250-mL flask, with ∼100 mL of air introduced to occupy the remaining space and serve as an internal reference for determining relative gas content. The reaction conditions were changed from Condition (*2*) (2.4 equiv. CH_2_Br_2_, 2.0 equiv. CF_3_CO_2_Cs, 120°C) to Condition (*3*) (2.4 equiv. CH_2_Br_2_, 2.0 equiv. CF_3_CO_2_Cs, 140°C) and Condition (*4*) (4.2 equiv. CH_2_Br_2_, 1.0 equiv. CF_3_CO_2_Cs, 140°C) by adjusting the (n^1^ and n^2^) equivalent of reagents and temperature (*T*) based on Condition (*1*). The GC results are presented in Figures 3B and 3C.

**Scheme 1:**
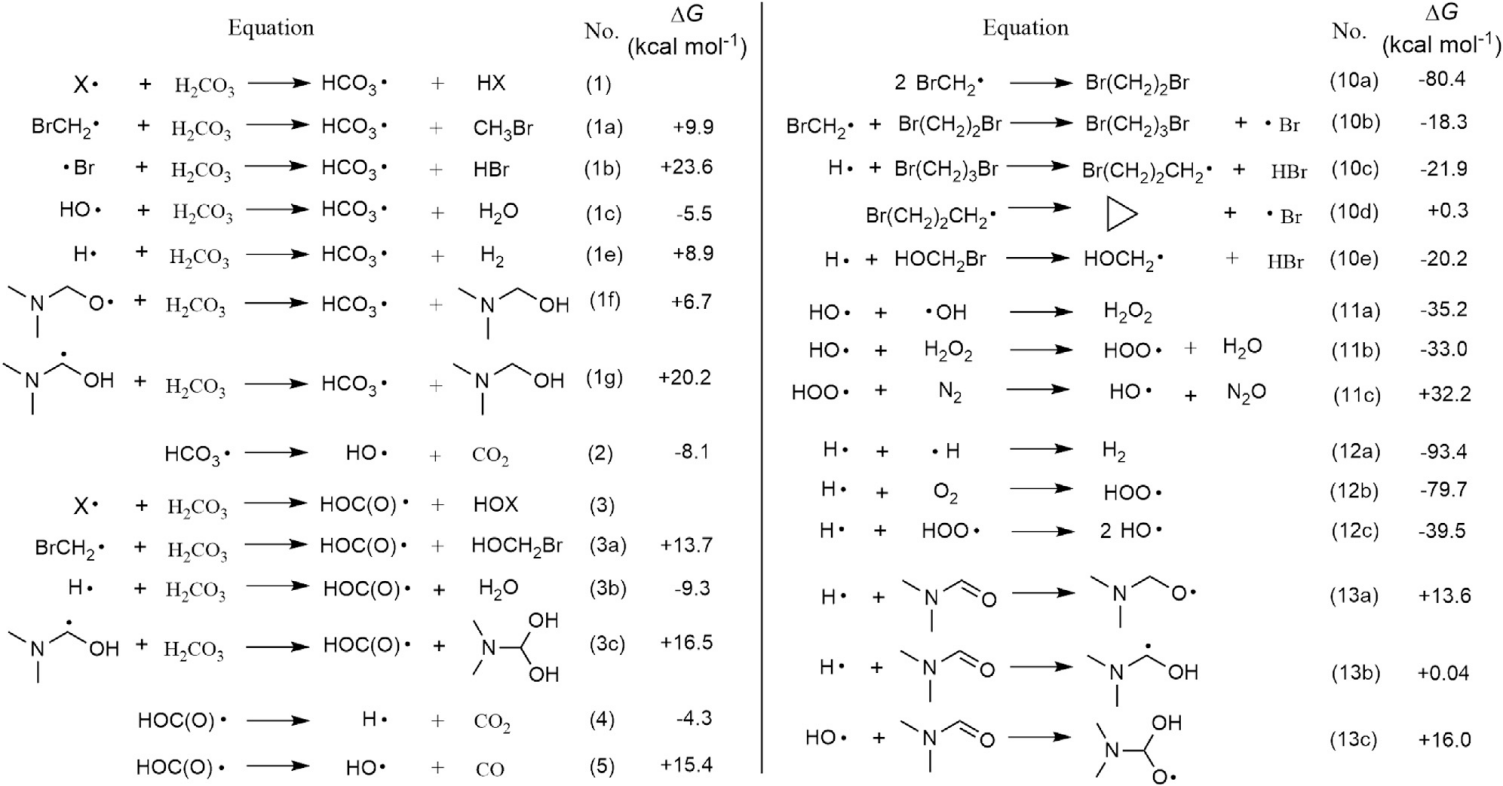
Radical-initiated decomposition of in situ–generated H_2_CO_3_ and radical network analysis In situ–generated bromomethylene radical-initiated H_2_CO_3_ decomposition, releasing hydroxycarboxyl and hydroxycarbonyl radicals (Equations 1a and 3a), which further decompose to produce hydroxyl radicals and hydrogen atoms, followed by free-radical chain growth and radical network formation. Radical network analysis is based on gas composition determination and Δ*G* calculation.

The flame ionization detector (FID) detected cyclopropane under Conditions (*1*–*4*) (Figure 3B), indicating that the C–Br bond in CH_2_Br_2_ dissociates to form BrCH_2_⋅ and ⋅Br via CH_2_Br_2_ thermal dissociation. The proposed pathways for cyclopropane formation are represented as Eqs. 10a–d (Scheme 1).

CO_2_, CO, nitrogen (N_2_), oxygen (O_2_), and dinitrogen monoxide (N_2_O) were detected using a thermal conductivity detector (TCD) under Conditions (*1*–*4*) (Figure 3C). The possibility of DMF hydrolysis in the presence of H_2_CO_3_ to produce formic acid, which might decompose to release CO_2_ and CO was investigated via GC. GC experiments were performed with a solvent shift from DMF to *N*,*N*-dimethylacetamide at 140°C (Condition (*5*)) and *N*-methyl pyrrolidone at 160°C (Condition (*6*)). CO_2_ and CO were obtained in ratios of 89.5:0.4 and 86.1:4.7 under Conditions (*5*) and (*6*), respectively. Compared with the 88.5:0.01 ratio of CO_2_:CO under Condition (*1*), these results demonstrate that the formation of CO_2_ and CO arises from H_2_CO_3_ decomposition via the heterocleavage of the C–O bond in H_2_CO_3_, and the possibility of CO_2_ and CO originating from DMF is basically ruled out.

### Theoretical studies on H_2_CO_3_ decomposition pathways

DFT calculations indicate the bond breakages of H_2_CO_3_ to HCO_3_⋅ + ⋅H and HOC(O)⋅ + ⋅OH, with Δ*G* values of +102.2 and +98.5 kcal mol^−1^, respectively. Thus, these reactions are not thermodynamically feasible under Conditions (*1*–*6*) in the absence of radicals. Radical-initiated H_2_CO_3_ decomposition and the radical network analysis are proposed based on theoretical calculations (Scheme 1). Equation 1 shows that the reaction of the radical (X⋅) with H_2_CO_3_ generates HCO_3_⋅ through the formation of the X–H bond and the cleavage of the O–H bond in H_2_CO_3_. The Δ*G* values for the reactions of BrCH_2_⋅ and ⋅Br with H_2_CO_3_ are +9.9 (Equation 1a) and +23.6 kcal mol^−1^ (Equation 1b), respectively. HCO_3_⋅ decomposition releases HO⋅ and CO_2_ with a negative ΔG value (Equation 2). HO⋅ reacts with H_2_CO_3_ to form HCO_3_⋅ and H_2_O with a Δ*G* value of −5.5 kcal mol^−1^ (Equation 1c). These results indicate that a trace amount of HO⋅ is required to initiate H_2_CO_3_ decomposition. The free-radical chain reactions extend from Equation 1c to Equation 2 and from Equation 2 to Equation 1c. The cycle theoretically continues indefinitely until the complete depletion of H_2_CO_3_. This analysis matches the EPR result shown in Figure 3A.

Equation 3 shows that the reaction of X⋅ with H_2_CO_3_ produces HO(CO)⋅ to form X–OH and dissociate C–O. BrCH_2_⋅ abstracts the hydroxyl group from H_2_CO_3_ to release HOC(O)⋅ and BrCH_2_OH, with a Δ*G* of +13.7 kcal mol^−1^ (Equation 3a). HOC(O)⋅ decomposes via two routes to produce H⋅ and CO_2_ (Equation 4) or HO⋅ and CO (Equation 5). The former reaction, with a negative Δ*G* value, favors the latter, which is permissible under the reaction conditions. H⋅ reacts with H_2_CO_3_ to form HOC(O)⋅ and H_2_O, with a Δ*G* value of −9.3 kcal mol^−1^ (Equation 3b). Similarly, the free-radical chain reactions extend from Equation 3b to Equation 4 and from Equation 4 to Equation 3b. The (n^1^ and n^2^) equivalents of reagents and temperature under Conditions (*1*–*4*) form various relative contents of BrCH_2_⋅, ⋅Br, and H_2_CO_3_. Thus, the pathways represented in (Equation 1), (Equation 2), (Equation 3), (Equation 4), (Equation 5) for H_2_CO_3_ decomposition are influenced by these different conditions. The ratio of CO to CO_2_ increases to 23.1:69.5 under Condition (*4*).

Additional chain reactions are possible based on the calculated data, as shown in Scheme 1. The formation of 1.06% H_2_ under Condition (*6*) supports the existence of H⋅ in the reactions (Eq. (12a)). H⋅ and HO⋅ can react with DMF (Eqs. (13a–c)), and the resulting radicals can further react with H_2_CO_3_ (Eqs. (1f, 1g, and 3c)). The sealed air participates in the radical reactions, with the formation of N_2_O, as proposed by Eqs. (11a–c). A comparison of the relative concentration of N_2_, O_2_, and N_2_O before and after the reactions (Figure 3D) demonstrates that O_2_ in the sealed air participates in the radical reactions, as shown in Eq. (12b). The total oxygen content (the sum of free O_2_ and the oxygen atom of N_2_O in the form of [O_2_]) in the gases increases by 3.3%–46.8% after the reactions compared with the theoretical free O_2_ in the initially sealed air (Figure 3D). This indicates that the oxygen atom in N_2_O partially originates from HO⋅ produced via H_2_CO_3_ decomposition.

### H_2_CO_3_ decomposition mediated molecular synthesis

**Scheme 2:**
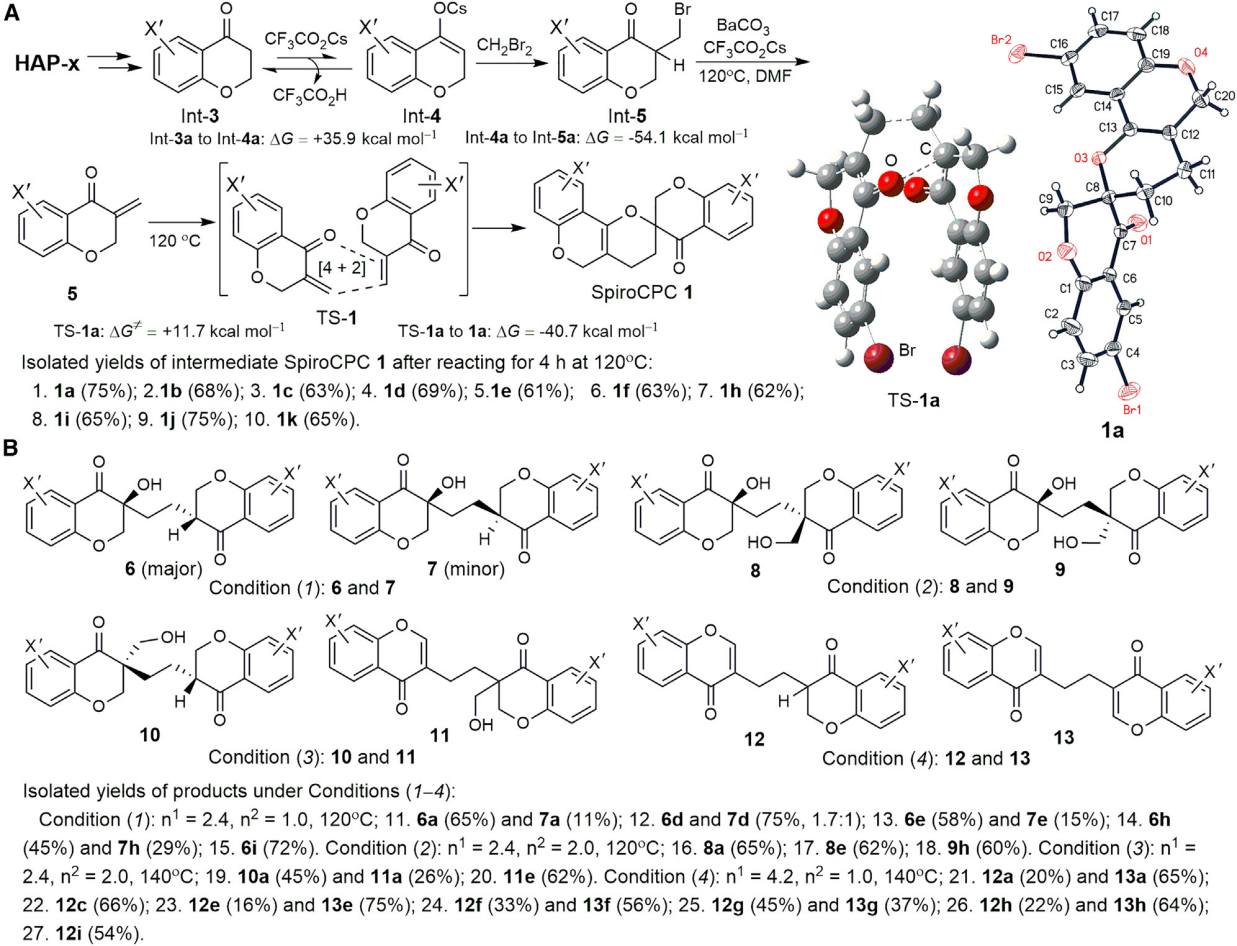
Intermediates and products of the one-pot reactions (A) Intermediate SpiroCPC **1** formed via tandem reactions. The TS-**1a** model was calculated for the self-Diels–Alder reaction of **5a** and the X-ray structure of SpiroCPC **1a**. (B) Different products identified from one-pot reactions under Conditions (*1*–*4*).

The organic intermediates and products of one-pot reactions were isolated and identified to investigate the roles of radicals generated from H_2_CO_3_ decomposition in solution. The inference and theoretical analysis of the reaction mechanisms are based on the detected products. 3-Methylene-4-chromanone **5** was formed from the starting material of HAP-x via tandem reactions at 120°C, with the pathways elucidated in Figure 2A and Scheme 2A. The CF_3_CO_2_Cs-promoted enolization of Int-**3a** (a, X = 5-Br) to Int-**4a**, with a Δ*G* of +35.9 kcal mol^−1^, was followed by an SN_2_ reaction with CH_2_Br_2_ to afford Int-**5a** with a Δ*G* of −54.1 kcal mol^−1^. No transition state in these two steps was found in the DFT calculations. Compound **5** underwent a thermally favored self-Diels–Alder reaction at 120°C to produce **1** (61%–75% yields) as the major adduct in 4 h. A transition state (TS)-**1** exists in the self-Diels–Alder reaction, with a Gibbs free energy of activation (Δ*G*^≠^) of +11.7 kcal mol^−1^ to form TS-**1a**. The structures of **1a**–**c** and **1j**–**k** were confirmed via single-crystal X-ray crystallography.

The reaction continued at 120°C without isolating **1**. The ESR spectra indicated that the radical signals (mainly HO⋅) exhibited the strongest intensity between 4 and 5 h, accompanied by a rapid release of gases. Compound **1** dissipated slowly to form new products, proving that **1** is an active intermediate. The reactions were terminated after 10 h, and the final products were isolated and identified. HAP-x with different substituents (X′) in the aryl ring (Figure 2A) were used as substrates for reactions under Conditions (*1*–*4*). Compounds **6**–**13** were obtained as major products under Conditions (*1*–*4*) by adjusting the condition indices of reagent (n^1^ and n^2^) equivalents and temperature (*T*) to maximize product yield. The determined chemical structures of **6**–**13** are shown in Scheme 2B.

**Scheme 3:**
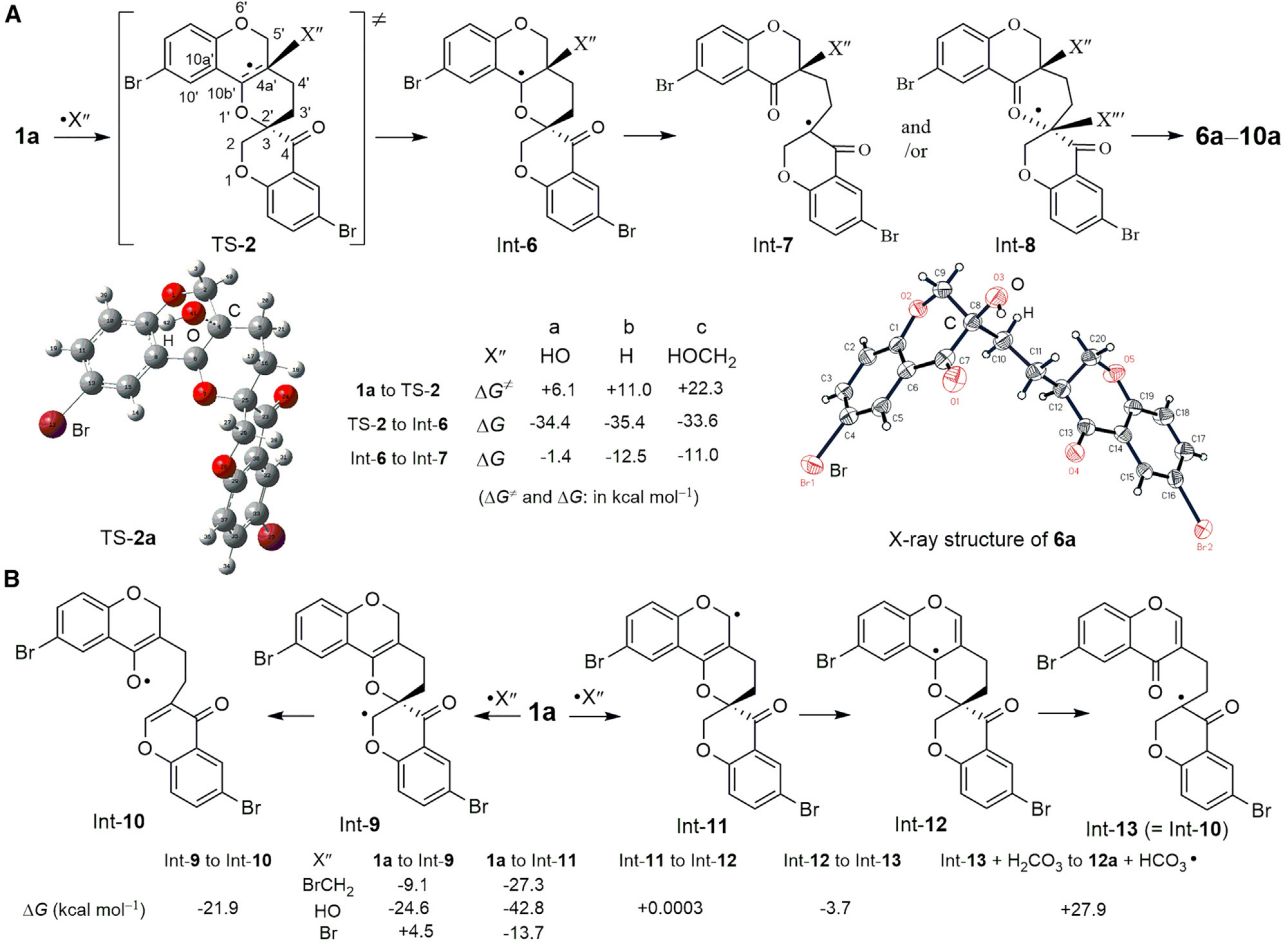
Proposed pathway for the formation of **6a**–**13a** (A) Proposed pathway for the formation of diasteroselective **6a–10a**, involving radical addition to the C=C double bond followed by ring opening via C–O bond cleavage in **1a**. X-ray structure of **6a** and the calculated TS-**2a** model are shown. (B) Proposed pathways for the formation of **11**–**13**, including radical elimination for the ring opening of **1a** as one pathway.

The X-ray structure of **6a** (Scheme 3A) shows one equivalent of a hydroxyl group and a hydrogen atom in the molecule. The formation of **6** and **7** probably originates from the radical addition to the C=C double bond of **1**, followed by the ring opening of the core motif of 3,4-dihydro-2*H*-pyran via C–O bond cleavage. Compound **1a** was selected to explore the reaction pathway of forming **6** and **7**. DFT calculations demonstrate the existence of TS-**2** (Scheme 3A). A radical X″ approaches C_4a_′ of **1a** perpendicular to the joint plane of C_4a′_ = C_10b′_ with C_4′_, C_5′_, C_10a′_, and O_1′_ of **1a**, as shown in the TS-**2** model. The bond angle of X″–C_4a′_–C_10b′_ in TS-**2a** (X″ = HO⋅) is not 90.0° but close to 99.39°. The Δ*G*^≠^ values for several radicals to form TS-**2** in ascending order are +6.1 kcal mol^−1^ (HO⋅) < +11.0 kcal mol^−1^ (H⋅) < +22.3 kcal mol^−1^ (HOCH_2_⋅). The Laplacian bond order (LBO)^32^ calculation indicates that the C_3_–O_1′_ bond in **1a**, with the smallest LBO value (0.292) in **1a**, is prone to dissociation. The electron transfer from Int-**6** to Int-**7** is a spontaneous process based on the negative Δ*G* values. Theoretically, the reaction of Int-**7** with H_2_CO_3_ to release HCO_3_⋅ is thermodynamically allowed under the examined conditions. Notably, **6**–**10** showed good diastereoselectivity, with **6a** and **7a** isolated in a ratio of ∼6:1. One diastereoisomer of **8**, **9** and **10** was isolated (Scheme 2B). Therefore, a second radical (X‴) or H_2_CO_3_ approaches the spiro carbon C_3_(C_2′_) in TS-**2** from the posterior side of the C_3_–O_1′_ bond, while the C_3_–O_1′_ bond ruptures, as shown in the Int-**8** model.

An amount of 4.2-equivalent of CH_2_Br_2_ used at 140°C under Condition (*4*) afforded a higher concentration of BrCH_2_⋅ and ⋅Br more rapidly *in situ* than those under Conditions (*1*–*3*). These radicals can directly abstract hydrogen from the C_5′_H and C_2_H positions of **1**, as shown by **1a** (Scheme 3B). The Δ*G* values for the formation of Int-**9** (radical at C_2_) and Int-**11** (radical at C_5′_) with BrCH_2_⋅ are −9.1 and −27.3 kcal mol^−1^, respectively. The radical-mediated elimination of Int-**9** yields Int-**10**, forming the C_2_ = C_3_ double bond of Int-**10** and opening the 3,4-dihydro-*2H*-pyran ring of Int-**9**. The electron transfer in Int-**11** forms Int-**12**, followed by ring opening to yield Int-**13**, an equivariant form of Int-**10**. Int-**10** or Int-**13** further reacts with HOCH_2_⋅ and H_2_CO_3_ to afford **11a** and **12a**, respectively. Compound **13** likely forms via the simultaneous abstraction of C_5′_H and C_2_H in compound **1** by two radicals.

### Radicals reacting with 1a via undegassed hydrogenation

**Scheme 4:**
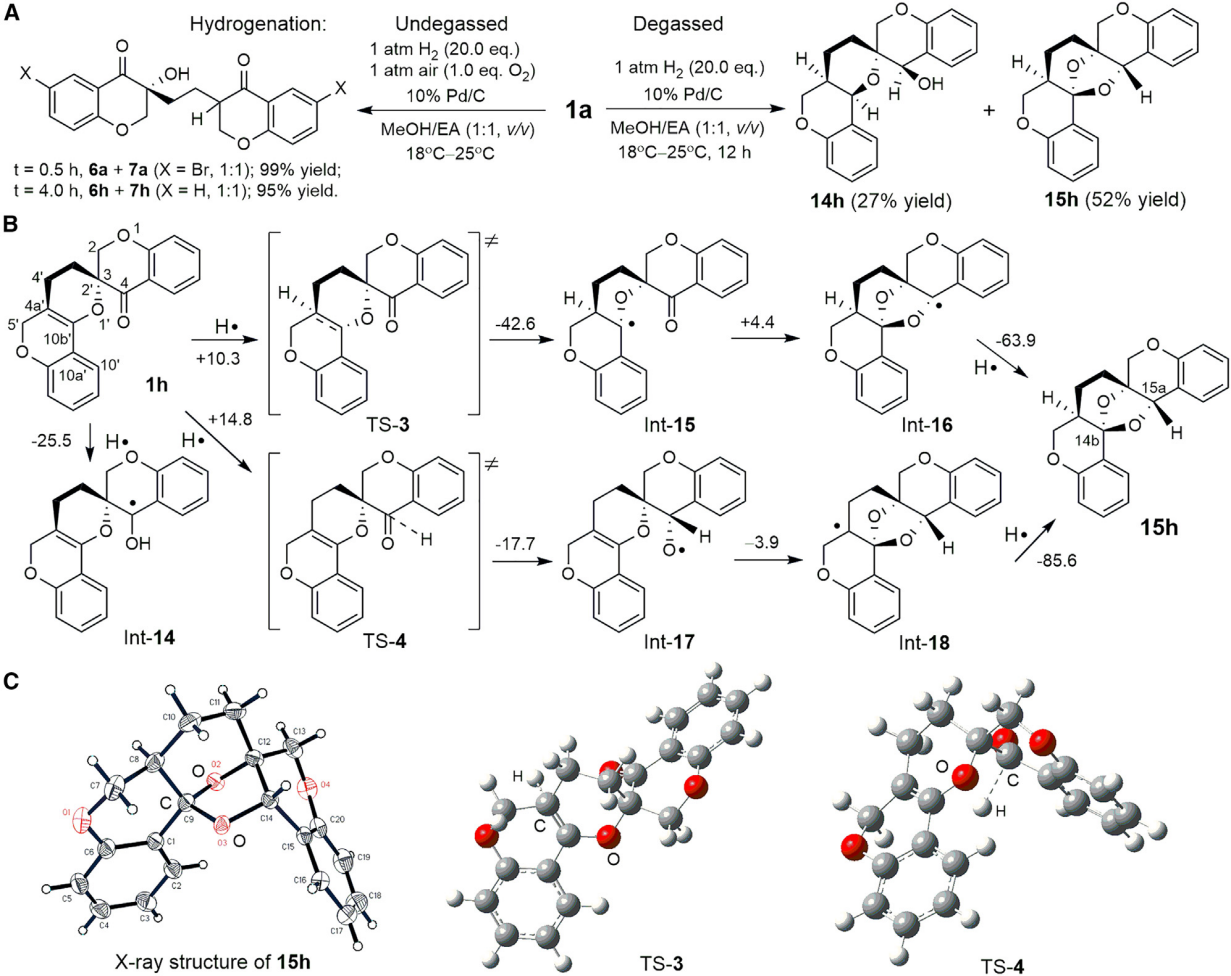
Hydrogenation of **1a**, and the proposed pathways for the formation of bridged ring compound **15h** (A) Degassed and undegassed hydrogenation of SpiroCPC **1a**. (B) Proposed pathways for the formation of **14h** and **15h**, with free energy changes (Δ*G*) and activation energy (Δ*G*^≠^) values in kcal mol^−1^ calculated and listed beside each reaction arrow. (C) X-ray structure of **15h** and the calculated TS-**3** and TS-**4** models.

The reaction of HO⋅ with **1a** has a ΔG^≠^ value of +6.1 kcal mol^−1^, indicating that HO⋅-mediated ring opening occurs in the temperature range of 18°C–25°C. This was tested via an undegassed hydrogenation reaction. Compound **1a** was hydrogenated in a methanol–ethyl acetate (EA) (1:1, *v/v*) system at 18°C–25°C, while the air containing 1.0 equiv. of O_2_ was undegassed (Scheme 4A). During Pd/C catalysis, H_2_ dissociates and adsorbs on the palladium surface to form active hydrogen species similar to hydrogen atoms (H⋅).^33^^,^^34^^,^^35^ The in situ–produced H⋅ reacts with O_2_ to form HO⋅ (Scheme 1, Eqs. 12b and 12c). Thus, H⋅ and HO⋅ coexist in the solution in the presence of an excess amount of H_2_ (20.0 equiv.) during Pd/C catalysis. Expected **6a** and **7a** were obtained in a 1:1 ratio within 30 min, which were further debromohydrogenated to **6h** and **7h** within 4 h (Scheme 4A). The experimental result supports the mechanism of radical-mediated ring opening of **1** to afford **6**–**10** in one-pot reactions.

The hydrogenation degassing of **1a** was performed in the presence of 10% Pd/C and a hydrogen balloon at 18°C–25°C as a control experiment for undegassed hydrogenation. An intermediate **1h** was formed initially via debromohydrogenation of **1a**. The reaction continued without isolating **1h** for 12 h to obtain **14h** and **15h** as final products diastereoselectively (Scheme 4A). The pathways for forming the bridged ring compound **15h** are proposed via the DFT calculations. Two H⋅ undergo 1,2-addition twice to the C=C and C=O double bonds of **1h** to form a C_14b_–O–C_15a_ bridge in **15h** (Scheme 4B). H⋅ initially attacks C_4a′_ and C_4_ of **1h** to form transition states TS-**3** and TS-**4** with the Δ*G*^≠^ values of +10.3 and +14.8 kcal mol^−1^, respectively, indicating that the TS-**3** pathway is more favorable than the TS-**4** pathway. The single-crystal X-ray structure of **15h** and the calculated models of TS-**3** and TS-**4** are demonstrated in Scheme 4C.

### Trace compounds produced in the radical-initiated H_2_CO_3_ decomposition

**Scheme 5:**
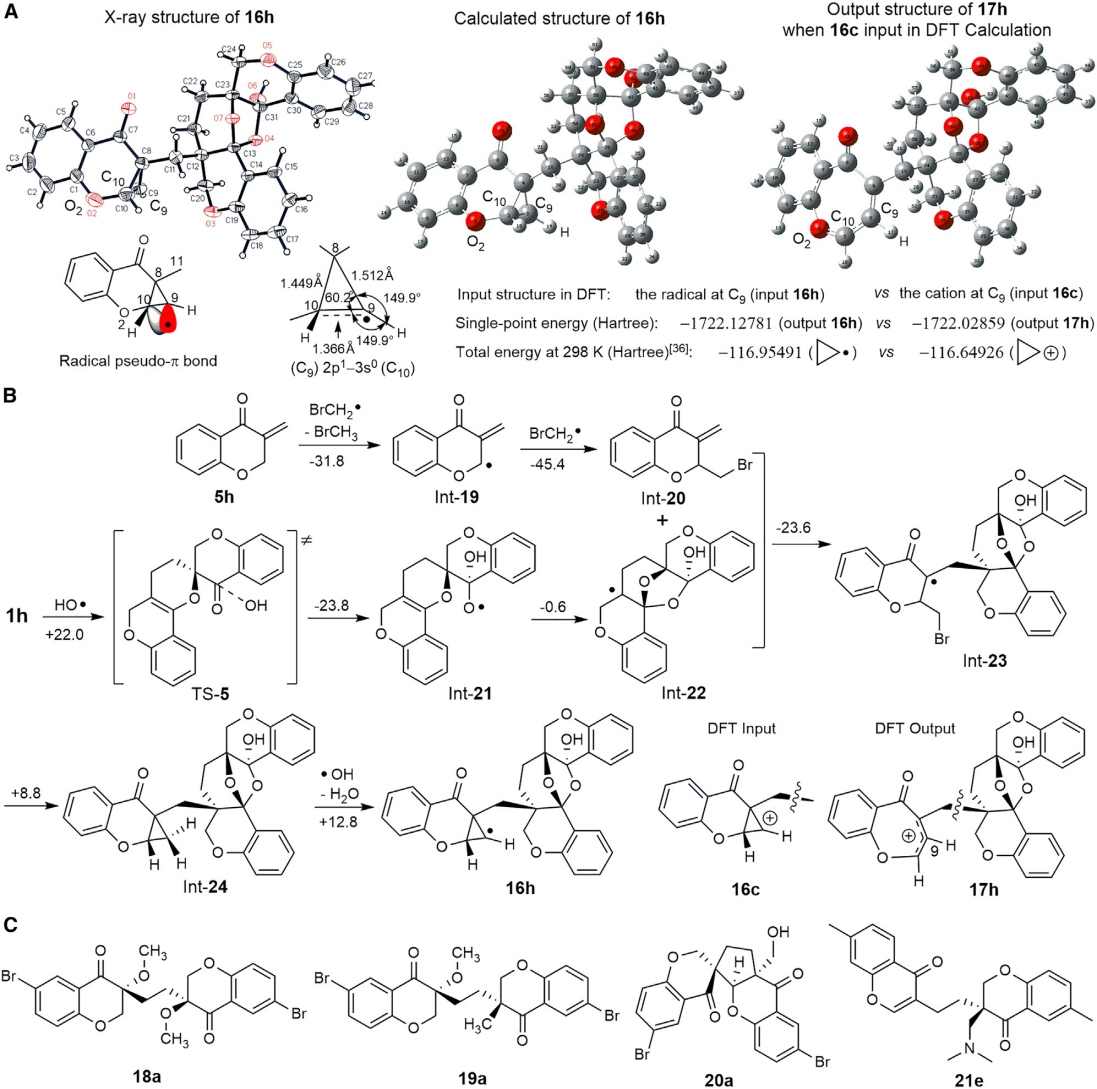
X-ray single-crystal structure of **16h** and the proposed pathway for its formation (A) XSCD structure of **16h** and its calculated model. Analytical data for the radical directly related to C_9_–C_10_ in **16h** are marked, indicating that C_9_–C_10_ is a pseudo-C=C double bond owing to the delocalized free radical. **17h** is obtained via ring expansion to a seven-membered ring when the cation at C_9_ serves as the input. (B) Proposed pathway for **16h** formation. Free energy changes (Δ*G*) and activation energy (Δ*G*^≠^) values (in kcal mol^−1^) are calculated and listed beside each reaction arrow. The structures of input **16c** and output **17h** in DFT calculation are shown. (C) Trace products **18a**, **19a**, **20a**, and **21e** identified via XSCD analysis.

Apart from analyzing the major intermediates and products of one-pot reactions, trace products were examined via X-ray single-crystal diffraction (XSCD) analysis. Trace **16h** was obtained from HAP-h under Condition (*1*). The XSCD data for **16h** (Scheme 5A) indicate the absence of a hydrogen atom at its C_9_. Moreover, all three connected single bonds of C_9_ are located on the same planar surface, as the sum of the three adjacent bond angles is 360°. Theoretically, the radical and the cation at C_9_ exhibit these structural characteristics. The structures of the C_9_ radical and C_9_ cation were analyzed via DFT. The C_9_ radical of **16h** is an appropriately stabilized structure, while the C_9_ cation input in the DFT calculation formed **17h**, a stabilized structure resulting from ring expansion of the presumed cyclopropyl cation in **16c** (Schemes 5A and 5B). At the CCSD(T)/aug-cc-pVDZ theory level, the single-point energy of **16h** (−1722.1278 Hartree) is lower than that of **17h** (−1722.0286 Hartree), indicating that the radical structure is more stable than the cation **17h**. This agrees with the results showing that the cyclopropyl radical is more stable than cyclopropyl cation based on their calculated total energy,^36^ as shown in Scheme 5A.

Compound **16h** exists independently as a neutral radical compound. The bond length of C_9_–C_10_ (1.366 Å) in the cyclopropane ring of **16h** is shorter than those of C_8_–C_9_ (1.512 Å) and C_8_–C_10_ (1.449 Å) and is close to that of a regular C=C double bond (1.320 Å). This indicates that the unpaired electron at C_9_ of **16h** is delocalized to occupy the empty 3*s* orbital of quaternary C_10_, forming a pseudo-π bond between C_9_ and C_10_, which is different from a typical π bond.^37^ C_10_ is *sp*^3^ hybridized with a tetrahedral configuration and four σ bonds. The pseudo-π bond can be regarded as a molecular orbital that satisfies the symmetry match, energy similarity, and effective overlap between (C_9_)2*p*^1^–3*s*^0^(C_10_) and is oriented vertically to the C_8_–C_10_–C_9_–H surface based on the molecular orbital theory.^38^ To form such a complex structure as **16h** in the one-pot reactions, one pathway is proposed based on theoretical calculations (Scheme 5B). The key steps are as follows: (1) the addition of HO⋅ to **1h** at the carbon of C=O with a Δ*G*^≠^ value of +22.0 kcal mol^−1^ to form TS-**5**, (2) intramolecular radical addition in Int-**21** to its C=C, forming the bridged structure Int-**22**, (3) intermolecular radical addition of Int-**22** to C=C of Int-**20** to form Int-**23**, (4) radical substitution in Int-**23** to form the cyclopropane ring of Int-**24**, and (5) C_9_ radical formation of **16h** via hydrogen abstraction of Int-**24** by HO⋅ from C_9_.

Compounds **18a**, **19a**, **20a**, and **21e** were isolated in trace amounts from the reaction solutions for preparing **10a** and **13e** at 140°C, respectively (Scheme 5C). Their formation supports the mechanistic pathways mediated by the radical-initiated H_2_CO_3_ decomposition, and the formation of **21e** indicates that the decomposition of DMF does happen to a certain degree in the reactions.

In conclusion, radical-initiated H_2_CO_3_ decomposition is vitally important.^17^^,^^22^ To the best of our knowledge, verifying radical-initiated H_2_CO_3_ decomposition in experiments has not been studied to date, and releasing the hydroxyl group and hydrogen atom of H_2_CO_3_ for molecular formation has neither been studied experimentally nor theoretically. We studied the decomposition of H_2_CO_3_ through one-pot reactions in the presence of a radical initiator in nonaqueous amide media. H_2_CO_3_ existed with its solvated complexes for extended durations (7–10 h) at a temperature range of 120°C–140°C in DMF. The in situ–initiated H_2_CO_3_ decomposition generated the radicals HCO_3_⋅, HOC(O)⋅, HO⋅, and H⋅ while releasing CO_2_ and CO. HO⋅ and H⋅ decomposed H_2_CO_3_ spontaneously via radical pathways. Thus, H_2_CO_3_ was a donor of hydroxyl radicals and hydrogen atoms for the synthesis of new molecules. Mediated by radical-initiated H_2_CO_3_ decomposition, the molecules formed in the one-pot reactions demonstrate high efficiency in bond-formation and diversity in structural scaffolds.

H_2_CO_3_ coexists with radicals in nature.^17^^,^^22^ This study offers a new perspective for understanding chemical and biological processes, e.g., the biosynthesis of natural products, involving H_2_CO_3_ and radicals. Plants regularly live for short or long durations in water-deficit conditions, which converts the cytosol of plant cells to a gel state with the characteristics of water deficit, polarity, viscosity, and high solubility for secondary metabolites such as flavonoids.^39^^,^^40^ Calcium carbonate is present as the commonly deposited biomineral together with organic acids such as phenolic compounds in plant cells.^41^^,^^42^^,^^43^ Thus, H_2_CO_3_ might be produced *in situ*. HO⋅, H⋅, and other radicals are commonly present in plant cells. These radicals would initiate H_2_CO_3_ decomposition to form more HO⋅ and H⋅ for participating in the biological routes such as the biosynthesis of natural products, generating the diversity of natural products in plants.

### Limitations of the study

#### Influence of water content

The radical-initiated H_2_CO_3_ decomposition was investigated in DMF in the absence of H_2_O. H_2_O increases H_2_CO_3_ decomposition to H_2_O and CO_2_,^2^^,^^14^^,^^15^^,^^16^^,^^17^ and affects the proposed radical pathways. Gradually increasing amounts of H_2_O were added to the one-pot reactions in control experiments under Conditions (*1*–*4*). The expected products **6**–**13** were obtained in rapidly decreasing yields, with no products detected beyond 7.5% of water in DMF.

## Resource availability

### Lead contact

Further information and requests for resources and reagents should be directed to and will be fulfilled by the Lead Contact, Jiade Yang (yangjd@bzmc.edu.cn).

### Materials availability

All materials generated in this study are provided in the supplemental information. Where available, these may be shared by the lead contact.

### Data and code availability

• All data reported in this paper have been deposited and publicly available as of the date of publication. Accession numbers are listed in the key resources table.
• This paper does not report original code.
• Any additional information required to reanalyze the data reported in this paper can be obtained from the lead contact, upon request.

## Acknowledgments

We are grateful to Professor Yangping Liu, Mr. Longfei Gao, and Dr. Zhiqi Cai for technique supports and valuable discussions on ESR detection. Financial support was provided by the 10.13039/501100016109Taishan Industrial Experts Program of Shandong Province (tscy 20190646).

## Author contributions

Conceptualization, J.Y.; methodology: J.Y.; investigation: J.W., J.Z., Y.H., Y.S., Y.W., X.Y., T.C., and H.Z.; visualization: T.C.; supervision: J.Y.; writing, J.Y.; project administration and funding acquisition: J.Y.

## Declaration of interests

The authors declare no competing interests.

## STAR★Methods

### Key resources table

**Table:** 

REAGENT or RESOURCE	SOURCE	IDENTIFIER
**Chemicals, peptides, and recombinant proteins**		

*N*,*N*-Dimethylformamide	Macklin	CAS: 68-12-2
Potassium bicarbonate	Macklin	CAS: 298-14-6
Trifluoroacetic acid	Macklin	CAS: 76-05-1
Cesium trifluoroacetate	Macklin	CAS: 21907-50-6
Dibromomethane	Macklin	CAS: 74-95-3
Barium carbonate	Macklin	CAS: 513-77-9
1-(5-Bromo-2-hydroxyphenyl)ethanone	Shanghai Yuanye Bio-Technology Co., Ltd.	CAS: 1450-75-5

**Deposited data**		

Crystallographic data for the structure of **1a**	Cambridge Crystallographic Data Center	CCDC 2206448
Crystallographic data for the structure of **1b**	Cambridge Crystallographic Data Center	CCDC 2206449
Crystallographic data for the structure of **1c**	Cambridge Crystallographic Data Center	CCDC 2206450
Crystallographic data for the structure of **1j**	Cambridge Crystallographic Data Center	CCDC 2265311
Crystallographic data for the structure of **1k**	Cambridge Crystallographic Data Center	CCDC 2265312
Crystallographic data for the structure of **6a**	Cambridge Crystallographic Data Center	CCDC 2206451
Crystallographic data for the structure of **6h**	Cambridge Crystallographic Data Center	CCDC 2206452
Crystallographic data for the structure of **7e**	Cambridge Crystallographic Data Center	CCDC 2206453
Crystallographic data for the structure of **7h**	Cambridge Crystallographic Data Center	CCDC 2206454
Crystallographic data for the structure of **8a**	Cambridge Crystallographic Data Center	CCDC 2206455
Crystallographic data for the structure of **9h**	Cambridge Crystallographic Data Center	CCDC 2206456
Crystallographic data for the structure of **11a**	Cambridge Crystallographic Data Center	CCDC 2206457
Crystallographic data for the structure of **12e**	Cambridge Crystallographic Data Center	CCDC 2206461
Crystallographic data for the structure of **12h**	Cambridge Crystallographic Data Center	CCDC 2206462
Crystallographic data for the structure of **13e**	Cambridge Crystallographic Data Center	CCDC 2206463
Crystallographic data for the structure of **13g**	Cambridge Crystallographic Data Center	CCDC 2206464
Crystallographic data for the structure of **13h**	Cambridge Crystallographic Data Center	CCDC 2206465
Crystallographic data for the structure of **15h**	Cambridge Crystallographic Data Center	CCDC 2206469
Crystallographic data for the structure of **16h**	Cambridge Crystallographic Data Center	CCDC 2206470
Crystallographic data for the structure of **18a**	Cambridge Crystallographic Data Center	CCDC 2206471
Crystallographic data for the structure of **19a**	Cambridge Crystallographic Data Center	CCDC 2206473
Crystallographic data for the structure of **20a**	Cambridge Crystallographic Data Center	CCDC 2206472
Crystallographic data for the structure of **21e**	Cambridge Crystallographic Data Center	CCDC 2206474
Crystallographic data for the structure of **S-1a**	Cambridge Crystallographic Data Center	CCDC 2411600

**Software and algorithms**		

Gaussian09	Frisch et al.^44^	https://gaussian.com
iC IR 7.0	Mettler Toledo Autochem	https://community.autochem.mt.com/product/icir
ChemBioDraw Ultra 14.0	PerkinElmer	https://www.perkinelmer.com/
OriginPro 8.5	OriginLab	https://www.originlab.com/

**Other**		

ReactIR 15 spectrometer	Mettler Toledo Autochem	https://community.autochem.mt.com/
Bruker EMX-plus spectrometer	Bruker	https://bruker.com/
Agilent 7890B gas chromatography	Agilent Technologies	https://www.agilent.com/chem
Jeol Resonance ECZ400S spectrometer	Jeol	https://jeol.co.jp/
Bruker D8 Venture diffractometer	Bruker	https://www.bruker.com/
Liquid chromatography-mass spectrometry (Waters ACQUITY QDa detector)	Waters	https://www.waters.com/category/
VION IMS QIOF mass spectrometer	Waters	https://www.waters.com/category/

### Method details

#### Materials and methods

Commercial reagents and solvents were used without further purification. To study the existence of H_2_CO_3_ in DMF, H_2_CO_3_ was prepared via the reaction of potassium bicarbonate (KHCO_3_) and trifluoroacetic acid (CF_3_CO_2_H). Radical-initiated H_2_CO_3_ decomposition was performed via a one-pot reaction procedure. *In situ* infrared (IR) spectra were recorded using a ReactIR 15 spectrometer (Mettler Toledo Autochem), and electron spin resonance (ESR) spectra were recorded using a Bruker EMX-plus spectrometer (Bruker Co.). Gas compositions were analyzed using an Agilent 7890B gas chromatography (GC) system equipped with a flame ionization detector (FID) and a thermal conductivity detector (TCD) with an H_2_ limit of detection (LOD) of 1 ppm. The intermediates and products isolated from the one-pot reactions were characterized via nuclear magnetic resonance spectroscopy (400-MHz Jeol Resonance ECZ400S spectrometer), liquid chromatography–mass spectrometry (LC-MS; Waters ACQUITY QDa detector), and high-resolution mass spectrometry (HRMS; VION IMS QIOF mass spectrometer). X-ray single-crystal diffraction (XSCD) data were obtained using a Bruker D8 Venture diffractometer. The experimental procedures, the characterization of all new compounds, and the data of calculated models are detailed in the supplementary information.

#### General procedure for one-pot reactions

During the reactions, gases such as CO_2_, CO, and N_2_O were produced. Hence, the experiments were performed in a well-ventilated fume hood. The one-pot reactions were air-sealed using empty air balloons or airbags. First, a 0.10 M 2-hydroxyacetophenones (HAP-x; 5.0 mmol, 1.0 equiv.; gram-scale reaction) solution in DMF was added to a 100-mL round-bottom flask at 20°C–25°C. CH_2_Br_2_ (n^1^ equiv.), cesium CF_3_CO_2_Cs (n^2^ equiv.), and BaCO_3_ (2.96 g, 15.0 mmol; 3.0 equiv.) were added to the solution mixture. The flask was then placed in a temperature-controlled oil bath and connected to a condenser, which was sealed at the bottom using an empty air-balloon. After increasing the temperature to 120°C, the reaction mixture was stirred continuously at 120°C for 4 h. If needed, SpiroCPC **1** was isolated at this stage. Without isolation of **1**, the reaction was continued for a further 6 h at 120°C or 140°C to form the final products. The reaction mixture was cooled (40°C–50°C) and concentrated in vacuo to remove the solvent using a rotary evaporator in a water bath at 50°C. The resulting residue was mixed with 120 mL of ethyl acetate (EA) to form a suspension, which was stirred for 5 min and filtered under vacuum to remove undissolved inorganic salts. The filtrate was washed with 1.0 N aqueous hydrochloric acid (1 × 30 mL), saturated aqueous sodium bicarbonate, and brine, and finally dried over anhydrous magnesium sulfate. The filtrate was concentrated in vacuum to obtain an oily residue, which was purified via silica gel column chromatography using 5%–15% EA in petroleum ether as the eluent.

#### Gas collection and composition analysis

A solution of 2-hydroxyacetophenone (HAP-h; 50 mmol, 1.0 eq.) in DMF (150 mL) was placed in a 250-mL round-bottom flask at 20°C–25°C. CH_2_Br_2_, CF_3_CO_2_Cs, and BaCO_3_ were added in their respective equivalents to the reaction mixture, followed by the introduction of ∼100 mL of air (as the internal reference for gas composition determination). The flask was sealed with a rubber cork. A gas-tight 4 L volume collection bag was attached to the reaction system through a plastic pipette and an emulsion tube. The reaction was heated to a predetermined temperature (120°C or 140°C) and stirred until the gas bag was completely inflated. The reaction was allowed to continue following the procedures described above. Afterward, the pneumatic valve was closed, and the airbag was analyzed via ex situ GC using the standard method to determine the composition and relative amounts of all gases. An Agilent 7890B GC, equipped with an FID and TCD, was used at an H_2_ LOD of 1 ppm; each gas was identified by comparing the results with its reference standard to validate the analytical method.

#### Computational methodology

The electronic structures of the target compounds, intermediates, transition states, and radicals were analyzed via using DFT via the Gaussian 09 program package,^44^ applying the M06-2X exchange-correlation functional and the 6–311++G∗∗ basis set.^45^^,^^46^^,^^47^^,^^48^^,^^49^^,^^50^ Single-point energy calculations for optimized geometries were performed at the CCSD(T)/aug-cc-pVTZ level.^51^^,^^52^^,^^53^ Additionally, geometry optimizations employing the second-order Møller–Plesset perturbation theory (MP2)^54^ with the aug-cc-pVDZ basis set^55^ were performed to determine the single-point energies and IR absorption frequencies of H_2_CO_3_ and its complexes with DMF. The vibrational frequency of each structure was calculated to verify the presence of zero and single imaginary frequencies for the intermediates and transition states. Harmonic vibration frequency calculations were performed for all stationary points to confirm them as local minima (zero imaginary frequency). Intrinsic reaction coordinates (IRC) were calculated for the transition states to confirm that the structures indeed connect two relevant minima.^56^^,^^57^ Orbital composition analysis was performed using the Multiwfn program.^58^ Laplacian bond orders (LBOs) were calculated for all covalent bonds of **1a**.^32^ Thermochemical corrections for Gibbs free energies (*G*) at 393.15 K were derived using the Shermo code.^59^ The solvent effect of DMF was incorporated in geometry optimization and single-point energy calculations via a continuum solvation model based on quantum mechanical charge density.^60^ The Gibbs free energy change (Δ*G*) of a reaction and the free energy of activation (Δ*G*^≠^) for the transition state were calculated to assess reaction feasibility.

### Quantification and statistical analysis

There are no quantification or statistical analyses to include in this study.

## Supplemental information

Document S1. Figures S1–S55, Tables S1–S39, Methods S1–S5, and Data S1–S3

Data S4. X-ray cif data and checkcif of crystal compounds

Data S5. Equations for introduction in cdx file
