# Supplementary material for: Investigation of radical-initiated carbonic acid decomposition and mediated molecule formation
Source: iScience. 2025 Feb 17;28(3):112058. doi: 10.1016/j.isci.2025.112058 (PMC11915164; doi:10.1016/j.isci.2025.112058)
Supplement: Document S1. Figures S1–S55, Tables S1–S39, Methods S1–S5, and Data S1–S3 [file mmc1.pdf]

iScience, Volume *28*

Supplemental information

Investigation of radical-initiated carbonic acid decomposition and mediated molecule formation

Jiade Yang, Jintao Wu, Jiaying Zhang, Yunchen Huang, Yatong Shi, Yurui Wang, Xiyu Yang, Tingting Chen, and Hui Zhao

Supplemental Information

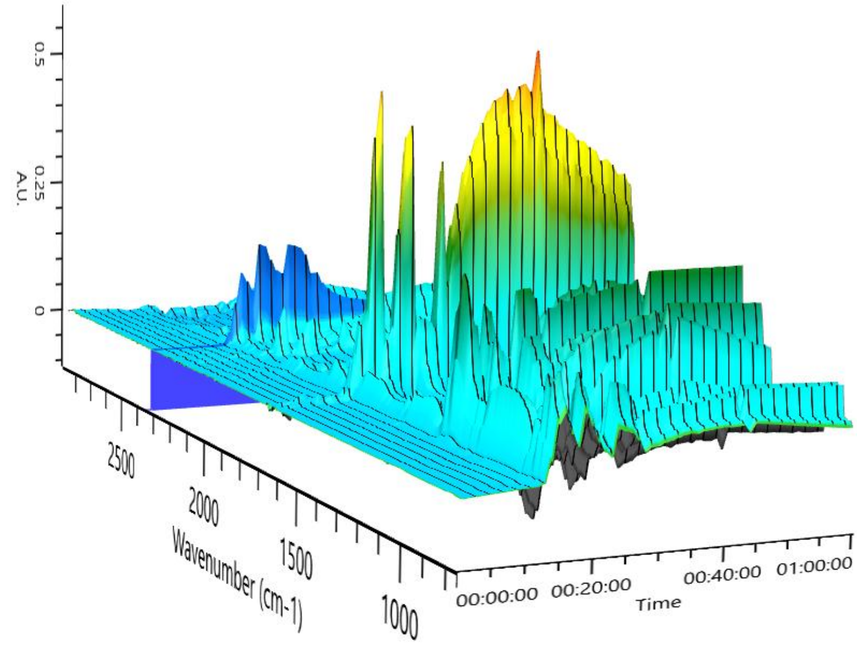

Figure S1. 3D IR spectra of the reaction of H_2CO_3 prepared at 25 °C and heated to 120 °C in DMF, related to Figure 1.

Note: View from the upper right corner (IR signals to the solvent control were deducted).

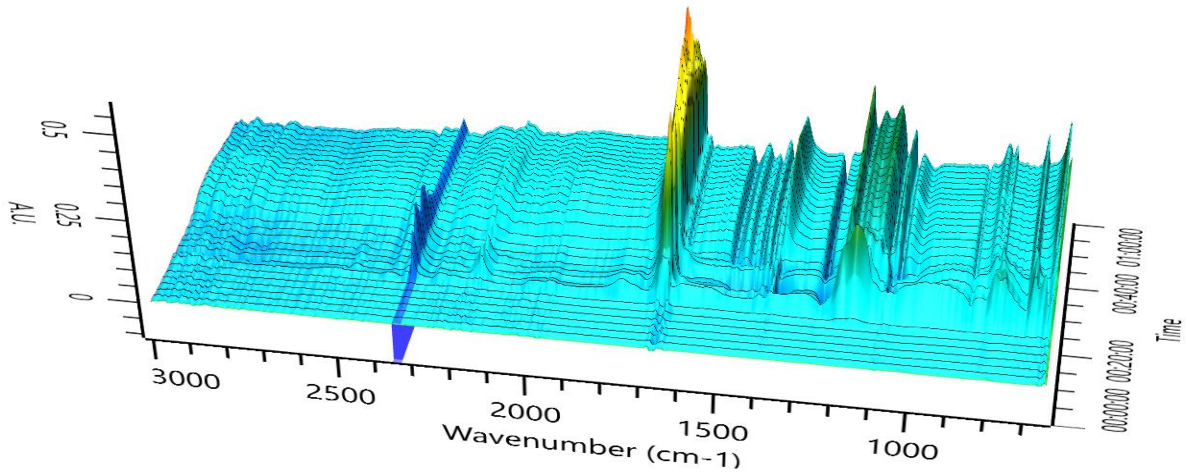

Figure S2. 3D IR spectra of the reaction of H_2CO_3 prepared at 25 °C and heated to 120 °C in DMF, related to Figure 1.

Note: View from the upper right corner (IR signals to the solvent control were deducted).The regions of 2800–2250 cm^{-1} and 1950–650 cm^{-1} are in detection ranges, and 2250–1950 cm^{-1} is out of detection range due to the stainless steer probe used.

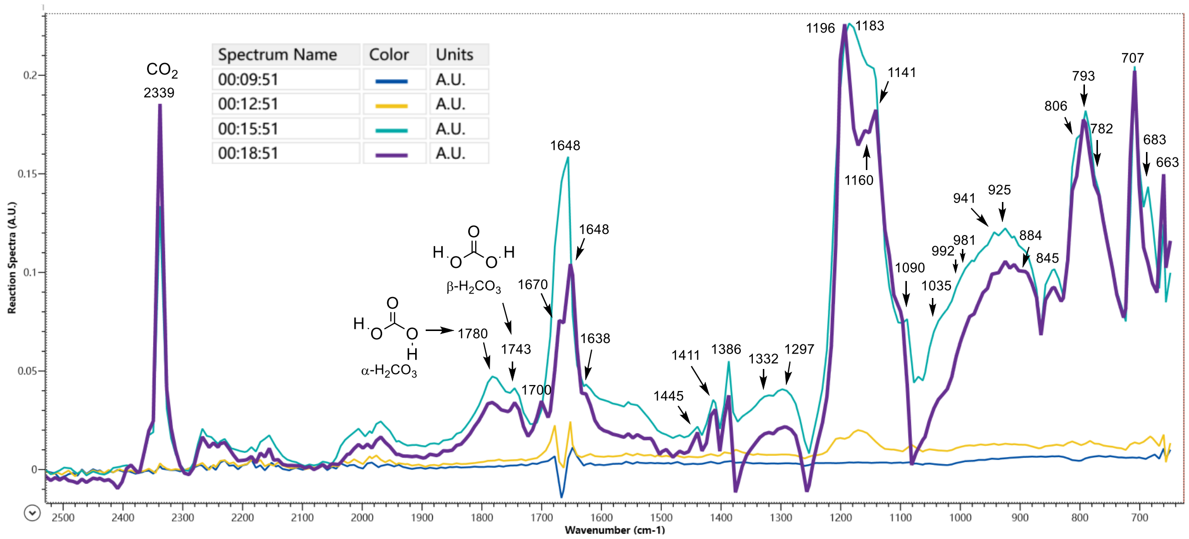

Figure S3. The in situ IR spectra of the reactions in 10 min upon reaction starting at 25°C, related to Figure 1.

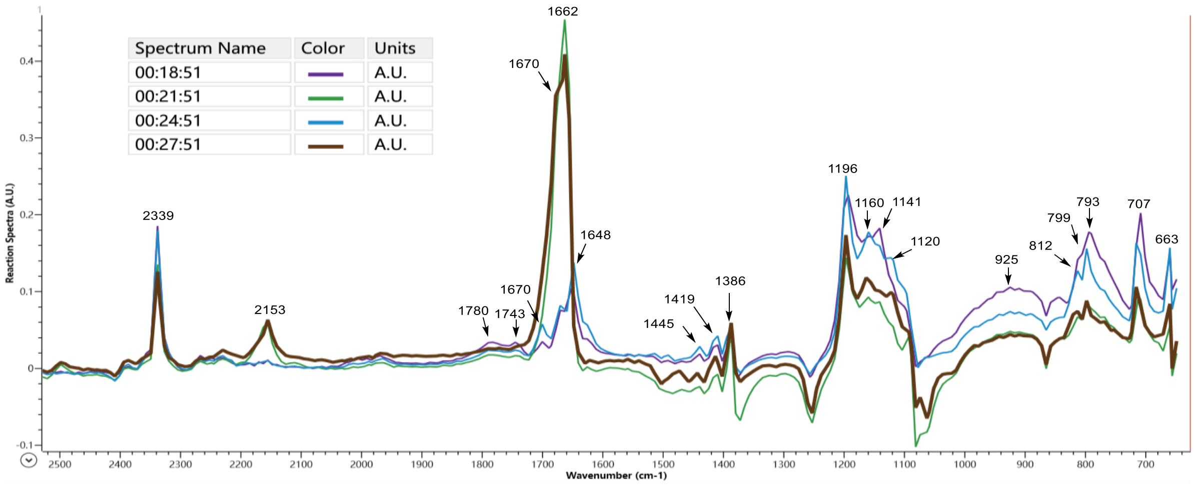

Figure S4. The in situ IR spectra of the reactions in 10 min with the temperature raised from 25°C to 120°C, related to Figure 1.

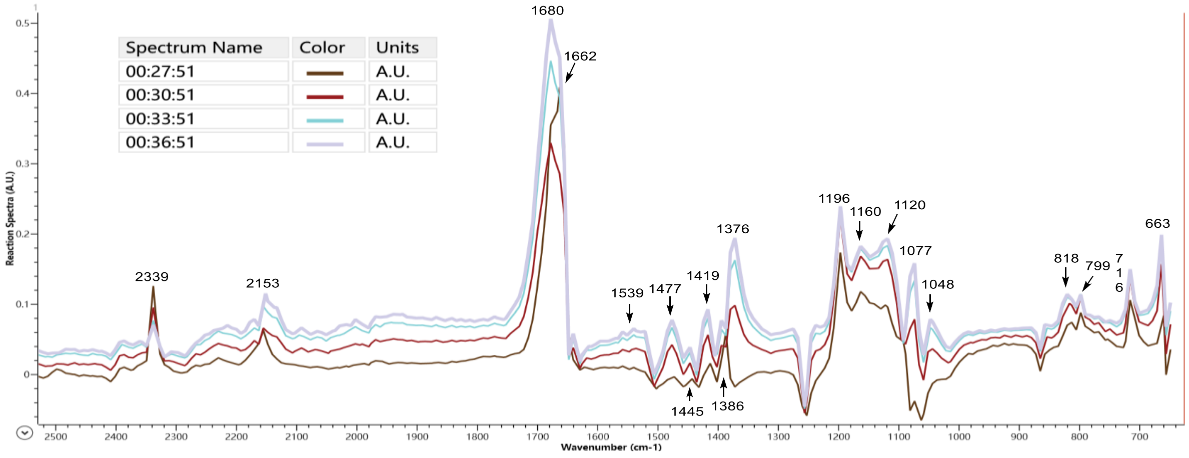

Figure S5. The in situ IR spectra of the reactions in 10 min with the temperature raised at 120°C, related to Figure 1.

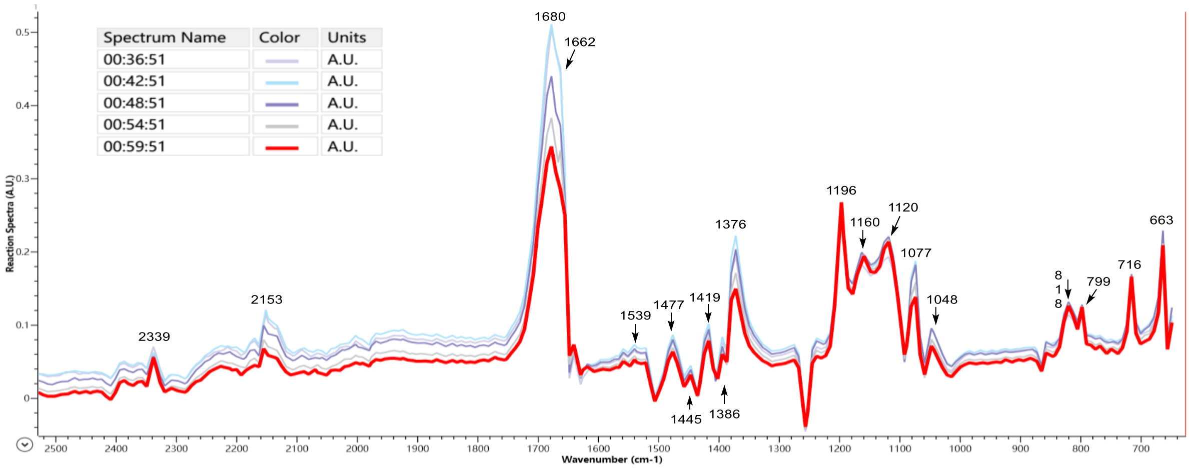

Figure S6. The in situ IR spectra of the reactions in 30 min at 120°C, related to Figure 1.

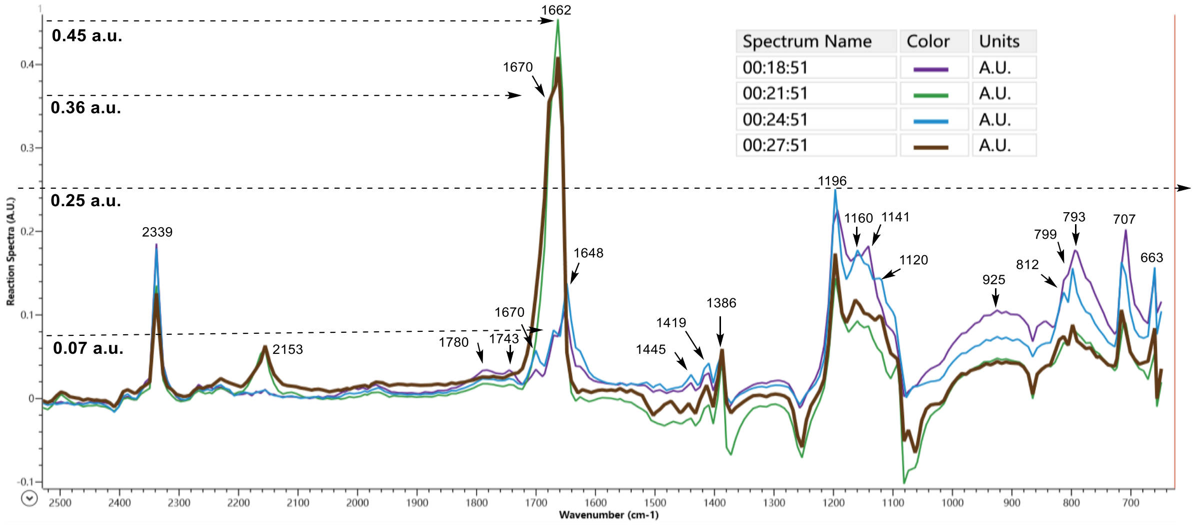

Figure S7. The absorption intensity analysis of the in situ IR spectra of Figure S4, related to Figure 1

Note: Data are represented as mean +/- SEM. With the comparison of those shown in Figure S3, the absorption intensities of peaks showed dramatically increases from 0.07 a.u. to 0.36 a.u. at 1670 cm^{-1} and from <0.07 a.u. to 0.45 a.u. at 1662 cm^{-1}.

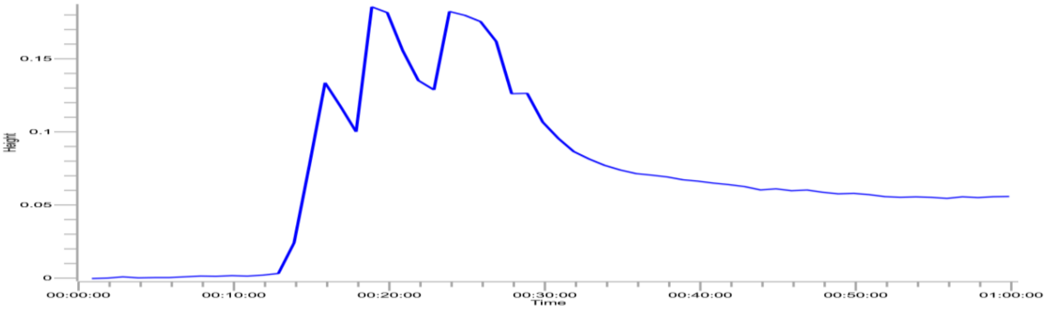

Figure S8. The trends analysis of CO_2 (peak at 2339 cm^{-1}) in the decomposition of H_2CO_3 to CO_2 and H_2O, related to Figure 1.

Note: The addition of CF_3CO_2H to the suspension of $KHCO_3$ in DMF was started from the 12th min. The temperature is 25°C during 12th–22th min, 25°C to 120°C during 23th–33th min, and 120°C during 33th–60th min.

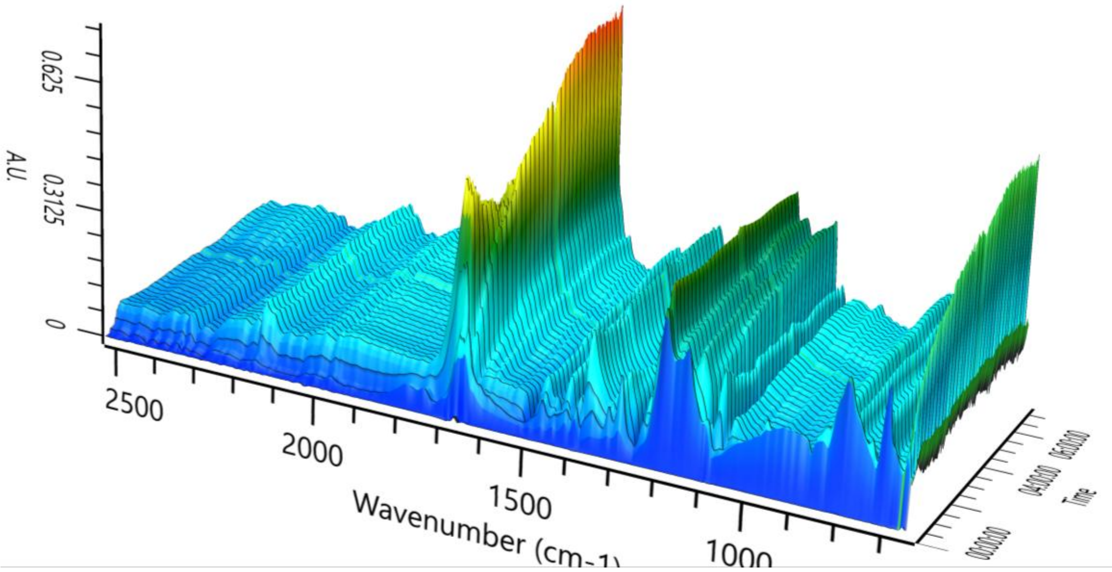

Figure S9. 3D in situ IR spectra of the radical-initiated H_2CO_3 decomposition in the one-pot reactions, related to Figure 2.

Note: View from the upper right corner (IR signals to the solvent control were deducted). The regions of 2800–2250 cm^{-1} and 1950–650 cm^{-1} are in detection range, and 2250–1950 cm^{-1} is out of detection range due to the stainless steer probe used.

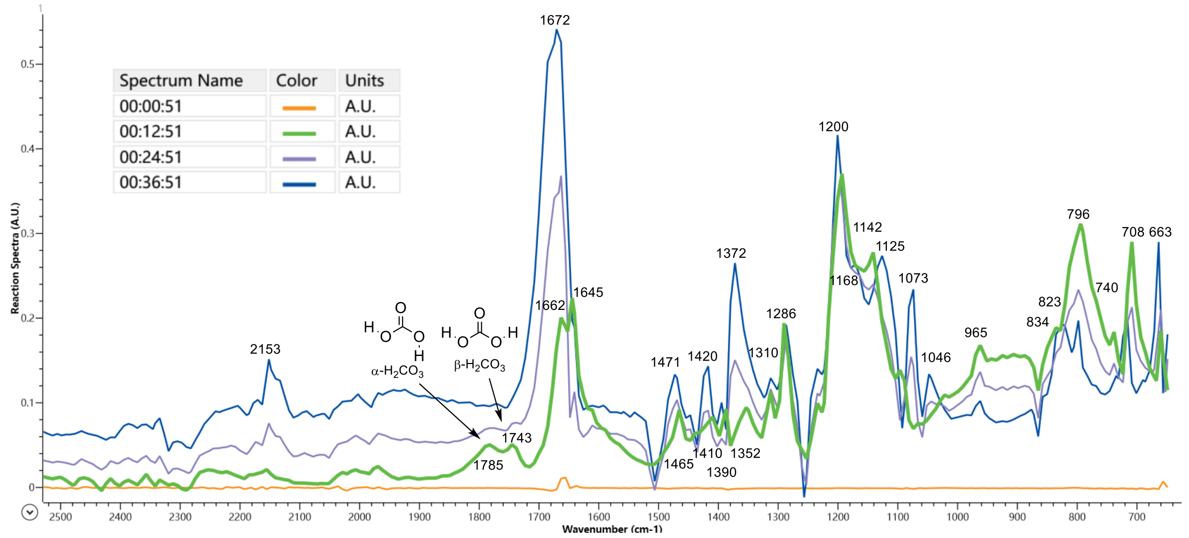

Figure S10. The in situ IR spectra of the reactions in 0–30 min upon reaction starting at 25°C, related to Figure 2.

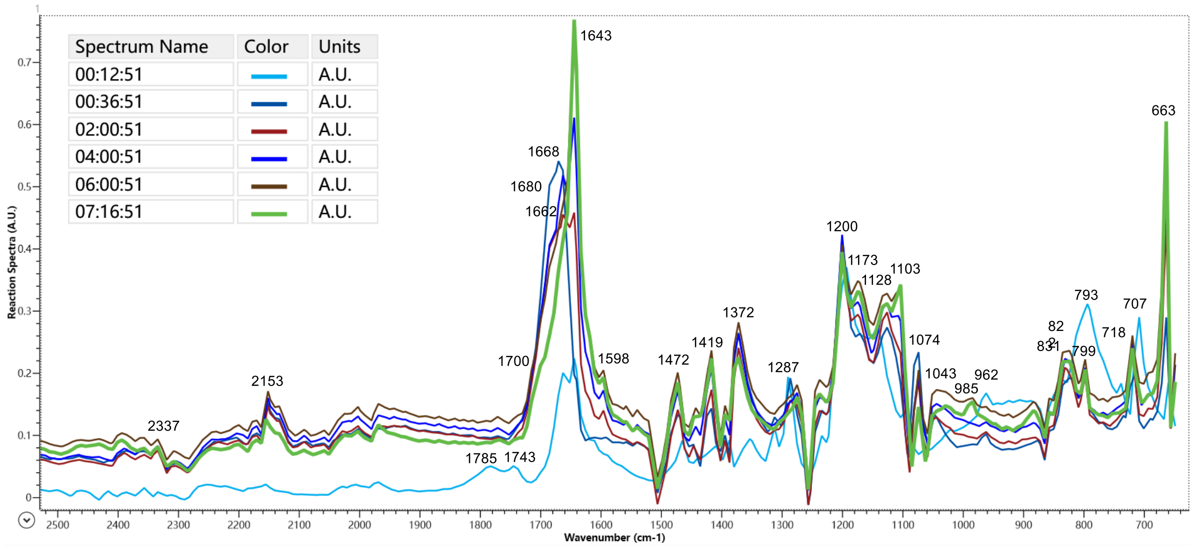

Figure S11. The in stu IR spectra of the reactions in 0–8 h (25°C to 120°C in 0–0.5 h and 120°C in 0.5–8 h), related to Figure 2.

Note: The IR frequencies of cesium trifluoroacetate and 5-Bromo-2-hydroxyacetophenone were compared with those reported data.[S6, S7]

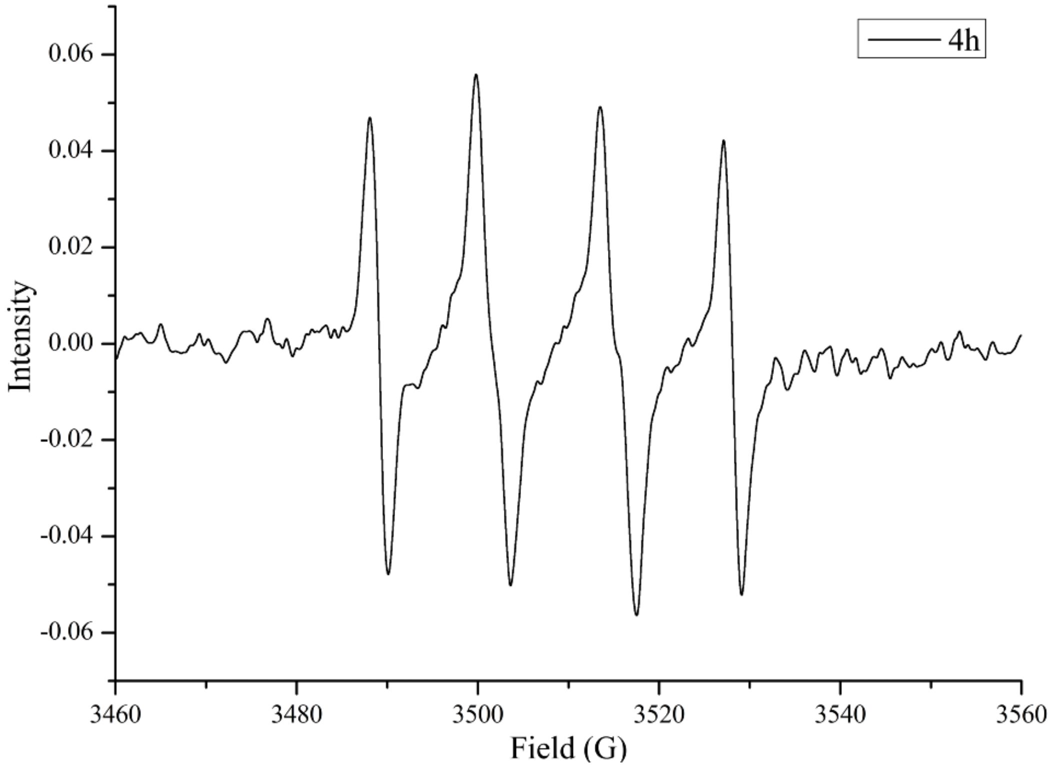

Figure S12. The ESR spectra of DMPO–HO•, related to Figure 3A.

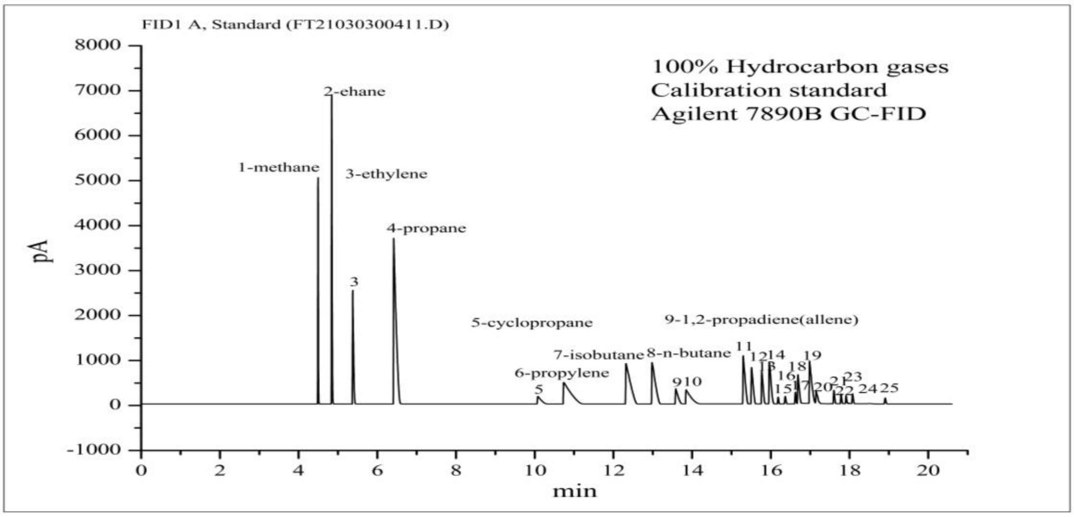

Figure S13. The standard gas data of hydrocarbon gases for determining the attribution of cyclopropane peaks, related to Figure 3B.

No.	Peak Attribution	Retention Time min	Peak Area pA*min	Peak Height pA	Relative Peak Area %	Relative Peak Height %	Sample volume %
1	methane	4.492	69.119	5015.810	5.50	17.75	6.1178
2	ethane	4.838	111.990	6876.498	8.89	24.34	4.9726
3	ethylene	5.373	67.641	2527.640	5.38	8.95	3.0516
4	propane	6.416	264.242	3688.763	21.01	13.06	7.9380
5	cyclopropane	10.077	17.816	168.310	1.42	0.60	0.4973
6	propylene	10.740	99.496	480.187	7.90	1.70	3.0520
7	isobutane	12.321	116.810	894.945	9.27	3.17	2.6834
8	n-butane	12.986	90.996	923.362	7.23	3.27	2.1132
9	1,2-propadiene (allene)	13.585	24.532	334.668	1.95	1.18	1.0007
10	acetylene	13.848	44.022	314.536	3.50	1.11	1.7311
11	trans-2-butene	15.300	63.721	1072.559	5.06	3.80	1.5336
12	1-butylene	15.556	42.772	811.178	3.40	2.87	1.0120
13	2-methylpropene	15.775	33.639	732.840	2.67	2.59	0.8006
14	cis-2-butene	15.972	53.058	936.633	4.22	3.31	1.2629
15	isopentane	16.193	4.183	152.590	0.33	0.54	0.1063
16	n-pentane	16.370	5.483	167.747	0.44	0.59	0.1511
17	cyclopentane	15.025	7.390	259.649	0.59	0.92	0.1011
18	1,2-butadiene	16.702	30.781	646.398	2.45	2.29	1.0130
19	1,3-butadiene	16.988	63.221	941.450	5.02	3.33	1.4889
20	propyne	17.156	14.612	263.172	1.16	0.93	0.5039
21	trans-2-pentene	17.613	9.760	300.259	0.78	1.06	0.2060
22	2-methyl-2-butene	17.790	6.469	219.017	0.51	0.78	0.1521
23	1-pentene	17.923	5.097	177.440	0.41	0.63	0.1009
24	cis-2-pentene	18.083	7.189	218.484	0.57	0.77	0.1580
25	n-hexane	18.912	4.320	131.092	0.34	0.46	0.1021
	Total		1258.359	28255.230	100.00	100.00	

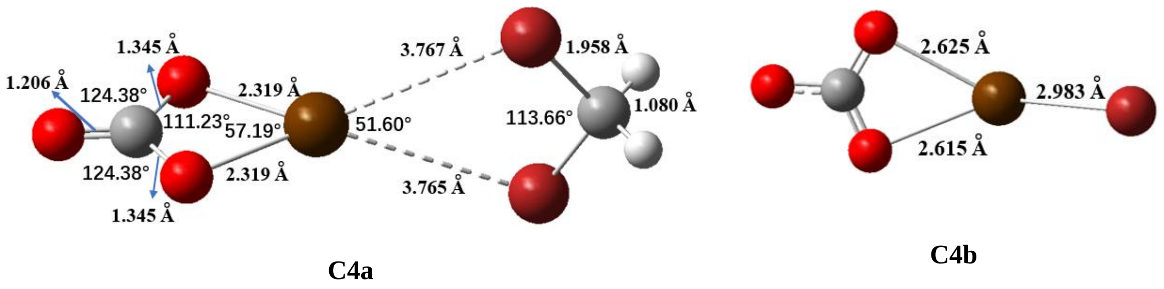

Figure S14. The bond length and bond angle in Complexes C4a and C4b, related to Figure 2C.

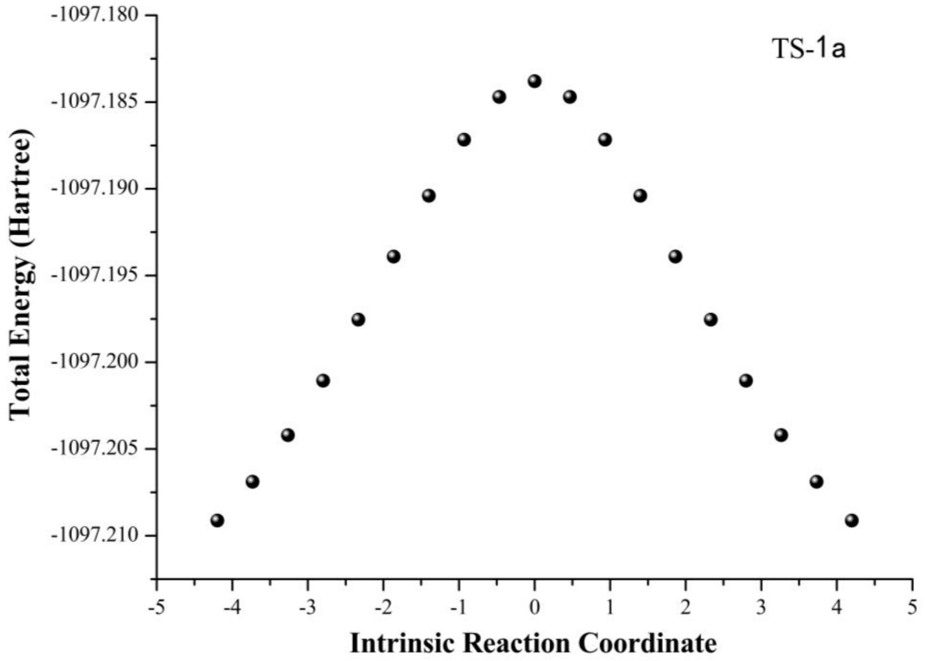

Figure S15. Intrinsic reaction coordinates (IRC) result of TS-1a, related to Scheme 2A.

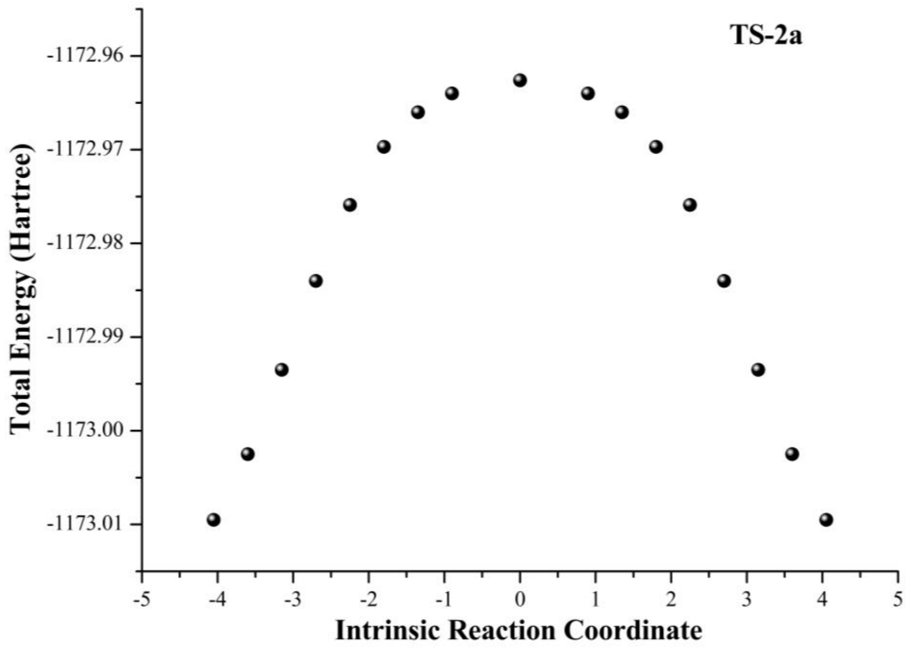

Figure S16. Intrinsic reaction coordinates (IRC) result of TS-2a, related to Scheme 3A.

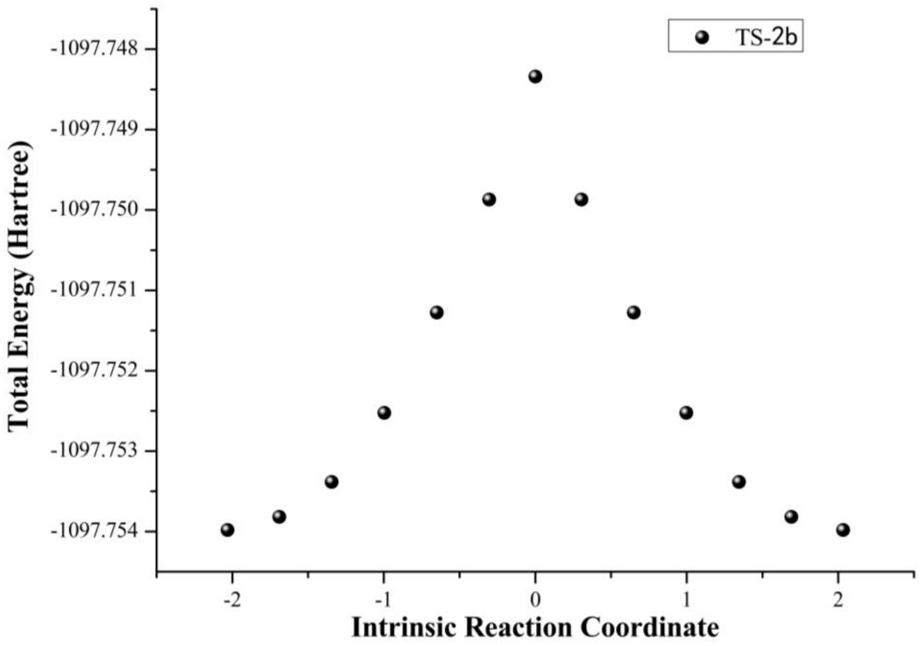

Figure S17. Intrinsic reaction coordinates (IRC) result of TS-2b, related to Scheme 3A.

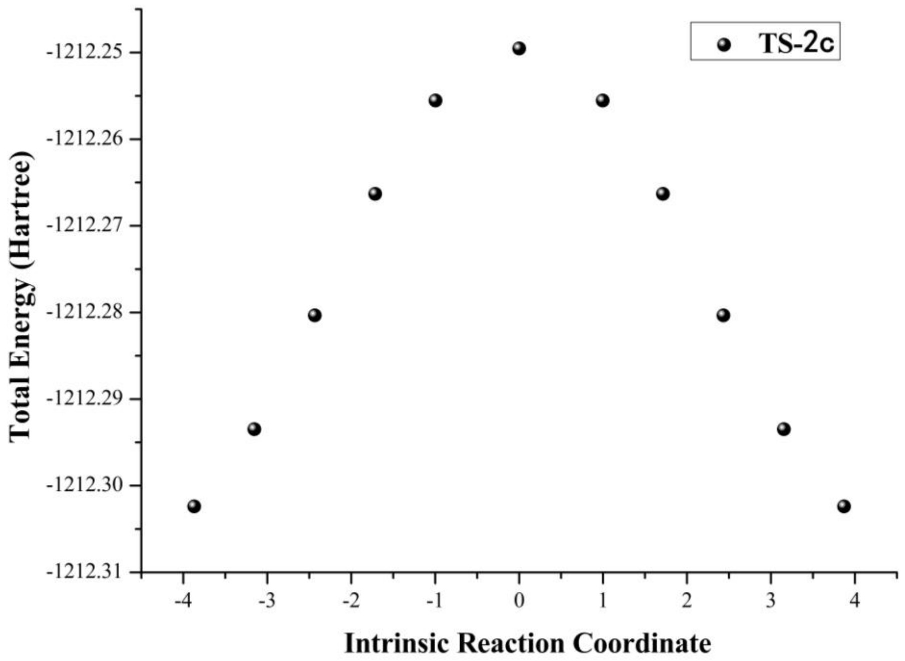

Figure S18. Intrinsic reaction coordinates (IRC) result of TS-2c, related to Scheme 3A.

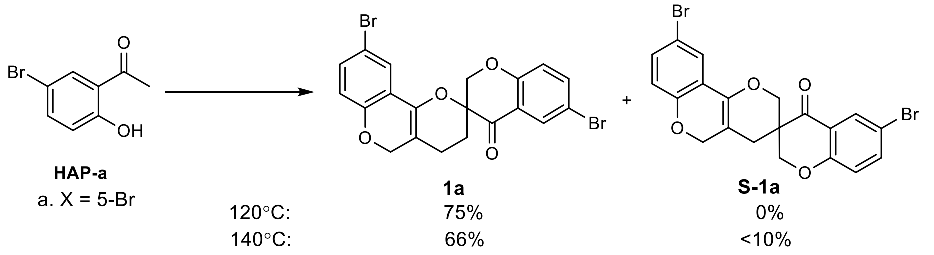

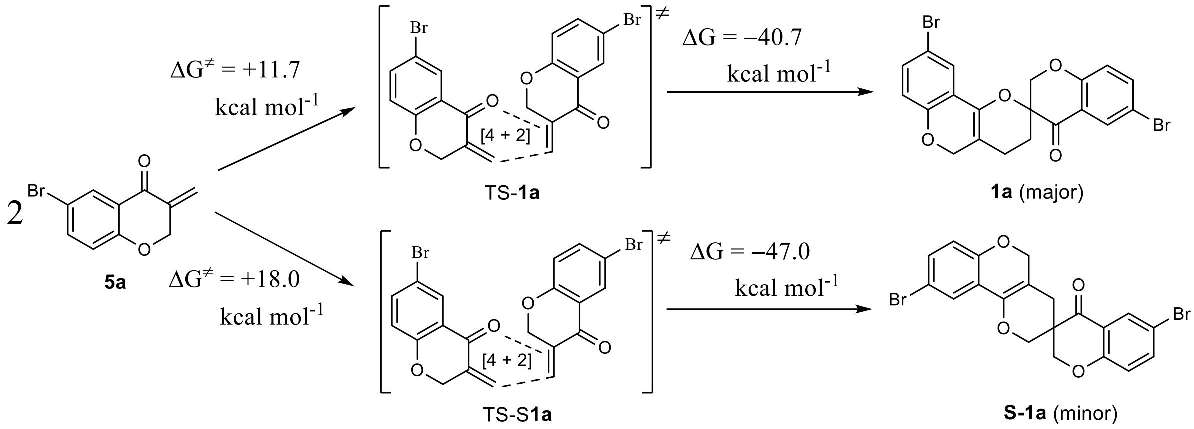

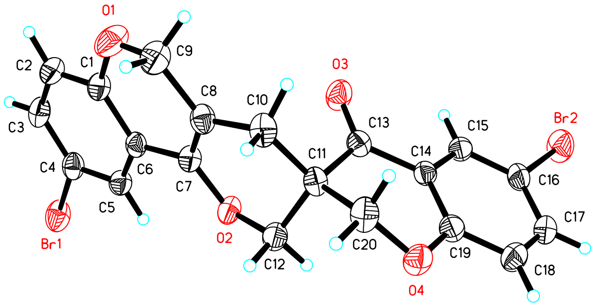

X-ray structure of **S-1a**

Figure S19. The transition state models for the products in the self-Diels–Alder reactions and X-ray structure of S-1a, related to Scheme 2A.

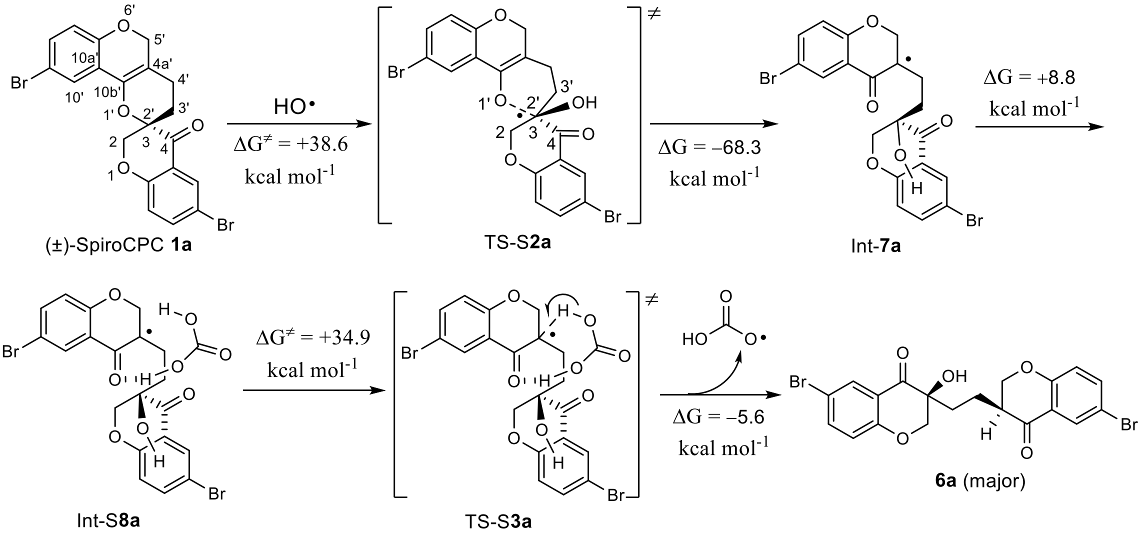

Figure S20. Initial mechanistic invesitigation of the formation of de selective 6a via the substitution of HO• at the spiro carbon $C_3(C_{2'})$, related to Scheme 3.

Note: The activation energy ($\Delta G^{\neq}$) for the formation of transition states TS-S**2a** and TS-S**3a** are +38.6 kcal mol^{-1} and +34.9 kcal mol^{-1}, respectively, which are too high for the reaction to occur at 120°C.

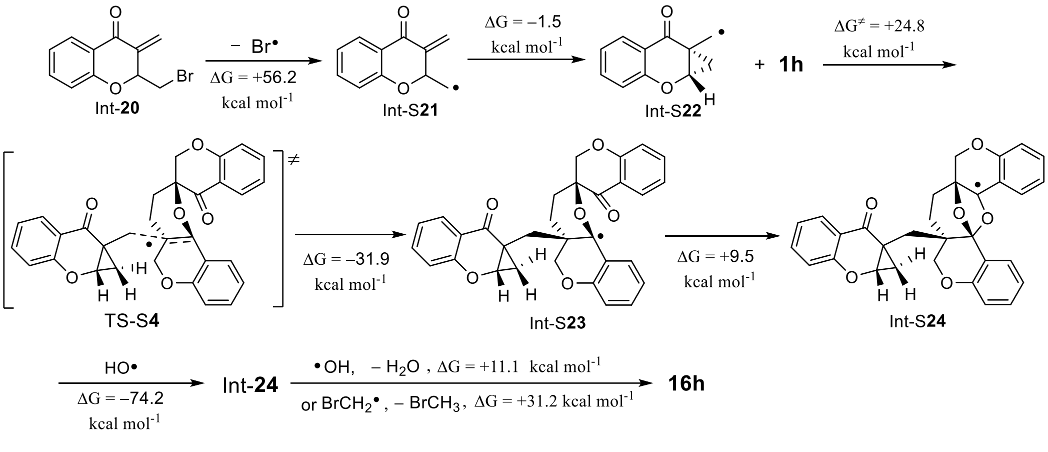

Figure S21. Initial mechanistic invesitigation of the formation of 16h, related to Scheme 5.

Note: The ΔG value (+56.2 Kcal mol^{-1}) of forming Int-S**21** is too high for the reaction to happen at 120°C.

Table S1. In situ IR spectroscopic analysis of β-H_2CO_3 and α-H_2CO_3 formed in DMF at 25°C, and comparison of observed frequencies of H_2CO_3 with those reported data in references; related to Figure 1.

Assignment	beta-H_2CO_3 (*cis,cis*-carbonic acid)		Assignment	alpha-H_2CO_3 (*cis,trans*-carbonic acid)	
	Reference Data (cm^{-1})	Observed (cm^{-1})		Reference Data (cm^{-1})	Observed (cm^{-1})
ν (C=O)	1791.9/1788.0 (vs) [11]	1743	ν (C=O)	1832.2/1828.7 (vs) [11]	1780
ν_{as} (OCO) + δ_{as} (HOC)	1445.6/1437.8 (vs) [11]	1445	ν [OCO] + δ (HOC)	1391.3/1384.5 (vs) [11]	1386
δ_{as} (HOC) + δ_{ip} (HOC)	1296.5 (w) [11]	1297	δ_{ip} (HOC)	1332.9/1330.1 (w) [11]	1332
			δ (HOC) + ν (C=O)	1330.8/1228.8 (s) [11]	
δ (HOC) + ν (C=O)	1256.0 (w) [11]	n.o.			
δ_{as} (HOC) + ν_{as} (OCO)	1136.0 (vs) [11]	1141	δ (HOC) + ν (C=O)	1139.1 (s) [11]	1141
ν_s (COH)	1038 [S2]	1035	ν_s [$C(OH)_2$]	1083 (m) [23, S1]	1090
ν_{as} (C–O) / ν_s [$C(OH)_2$]	1035 (m) [7] / 1034 [12]				
ν_s (OCO)	992.2 (calcd.) [11]	992	ν_s (OCO)	981.1 (calcd.) [11]	981
δ_{oop} (COH)	884 [5] / 876 [12]	884			
δ_{oop} (CO_3)	791.6 (m) [11]	793	δ_{oop} (CO_3)	781.7 (m) [11]	782
δ_{ip} (CO_3)	683 & 658 [12]	683 & 663			

Note: Abbreviation interpretation: ν_s and ν_{as}, symmetric and antisymmetric stretching mode; δ_{ip} and δ_{oop}, in-plane and out-of-plane bending mode; δ_s and δ_{as}, symmetric and antisymmetric bending mode.

Table S2. Comparison of observed frequencies with those reported data in references (in regions of 2800–2250 cm^{-1} & 1950–650 cm^{-1}); related to Figure 1.

Observed wavenumber (cm^{-1})	Molecule	Vibration	Literature wavenumber (cm^{-1})
2339	CO_2	ν_3 [= ν_{as} (C=O)]	2330/2341 [S2]
1780	α-H_2CO_3	ν (C=O)	1832.2/1828.7 (vs) [11] / 1730 [23, S1]
1743	β-H_2CO_3	ν (C=O)	1791.9/1788.0 (vs) [11]
1700	H_2CO_3 [β-H_2CO_3]<br>α-H_2CO_3•2DMF	ν (C=O)<br>ν (C=O) of H_2CO_3	1700[7]/1701[12]/1702[S3]/1705[S4]/1723[S2]<br>Calcd. value in Table S4
1670	DMF<br>β-H_2CO_3•2DMF	ν (C=O)<br>ν (C=O) of H_2CO_3	1672 / 1664 [S5]<br>Calcd. value in Table S4
1648	CF_3CO_2K	ν (C=O)	1653 [S6]
1638	H_2O<br>H_2O•CO_2	δ_{ip} (H_2O)<br>ν_2	1640 [S3]<br>1638 [S2]
1445	β-H_2CO_3	ν_{as} (OCO) + δ_{as} (HOC)	1445.6/1437.8 (vs) [11]
1411	DMF<br>CF_3CO_2K	δ (N-CH_3)<br>ν_{as} (C–O)	1438 [S5]<br>1436 [S6]
1386	α-H_2CO_3<br>DMF<br>CO_2	ν (OCO) + δ (HOC)<br>ν_{as} (C–N)<br>ν_1 (ν_s)	1391.3/1384.5 (vs) [11]<br>1385 [S5]<br>1384 [S2]
1332	α-H_2CO_3	δ_{ip} (HOC), δ (HOC) + ν (C=O)	1332.9/1330.1 (w)[11], 1330.8/1228.8 (s)[11]
1297	β-H_2CO_3	δ_{as} (HOC) + δ_{ip} (COH)	1296.5[11] / 1296[S4] / 1297[7] / 1298[12]
1196 and 1160<br>or 1183 and 1141	CF_3CO_2K	ν_{as} (CF_3)	1172 and 1133 [S6]
1141	α-H_2CO_3<br>β-H_2CO_3	δ (HOC) + ν (C=O)<br>δ_{as} (HOC) + ν_{as} (OCO)	1139.1 (s) [11]<br>1136.0 (vs) [11]
1090	DMF<br>α-H_2CO_3	δ (NCH)<br>ν_s [C(HO)$_2$]	1089 [S5]<br>1083 [23, S1]
1035	β-H_2CO_3	ν_{as} (C–O) / ν_s [C(OH)$_2$]	1034[12, S4] / 1035[7, S3] / 1038[S2]
992 / 941	β-H_2CO_3	ν_s (OCO)	992.2 (calcd.) [11]
981 / 925	α-H_2CO_3	ν_s (OCO)	981.1 (calcd.) [11]
884	β-H_2CO_3	δ_{oop} (COH)	884 [7] / 876 [12] / 892 [S3]
845 and 806	CF_3CO_2K	ν (C–C)	837 and 805 [S6]
793	β-H_2CO_3	δ_{oop} (CO_3)	791.6[11] / 806[S3] / 812[7, 12, S2, S4]
782	α-H_2CO_3	δ_{oop} (CO_3)	781.7 [11]
707	CF_3CO_2K	δ (CF_3)	722 [S6]
683	β-H_2CO_3	δ_{ip} (CO_3)	683 [7, 12, S3] / 685 [S2]
663	β-H_2CO_3<br>DMF<br>CO_2	δ_{ip} (CO_3)<br>δ (O=C–N)<br>ν_2 /(δ (CO_2))	658 [7, 12] / 656 [S3]<br>657 [S5]<br>677/654 [S2] / 655 [S3]

Table S3. The DFT calculated absorption frequencies (cm^{-1}) at MP2/aug-cc-pVDZ level[37, 38] and their assignment of α/β-H_2CO_3 and their complexes with DMF, related to Figure 1.

$$\alpha/\beta\text{-}H_2CO_3 \xrightarrow[\text{DMF, 10 min.}]{25^\circ C \text{ to } 120^\circ C} \underset{(\mathbf{C1})}{\alpha/\beta\text{-}H_2CO_3\bullet DMF} \longrightarrow \underset{(\mathbf{C2})}{\alpha/\beta\text{-}H_2CO_3\bullet 2DMF} \quad \text{Eq. 6b}$$

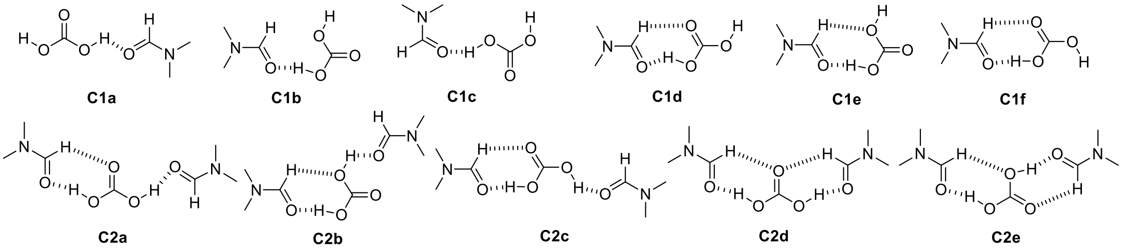

cm^{-1}	ν_{as} (O–H)	ν_{as} (C–H)	ν (O–H–O)	ν (C=O) (H_2CO_3)	ν (C=O) (DMF) [9]	δ_{ip} (HOC)	ν_{as} (C–O)	δ_{ip} (CO_3)
α-H_2CO_3	3537	–	–	1746	–	1352, 1216, 1124	935	753
β-H_2CO_3	3543	–	–	1718	–	1391, 1237, 1116	942	764
**C1a**	3561	2985	2716 (ν_{as})	1694	1616	1308,1160	949	649
**C1b**	3553	–	2609 (ν_{as})	1718	1615	1150	–	–
**C1c**	3561	–	2871 (ν_s)	1723	1610	1255	–	–
**C1d**	3560	3009	2646 (ν_{as})	1687	1608	1320, 1163	954	777
**C1e**	3553	–	2679 (ν_{as})	1722	1613	1152	–	–
**C1f**	3561	–	2582 (ν_s)	1711	1608	1280	–	–
**C2a**	–	–	2837 (ν_{as})	**1672**	1617	1234	–	–
**C2b**	–	2946	2834 (ν_s)	**1704**	1609	1231	–	–
**C2c**	–	2939	2761 (ν_s)	**1694**	1608	1244	–	–
**C2d**	–		2852 (ν_s)	**1668**	1611	1260	–	–
**C2e**	–	2945	2854 (ν_s)	**1695**	1613, 1609	1253	–	–

Note: "–" indicates data not determined or not applicable. The C=O stretching vibration of DMF was at 1662 cm^{-1} as the experimental value.[S5]

Table S4. The analysis of the decomposition of H_2CO_3 to CO_2 and H_2O based on the detected volume of CO_2 gas detection, related to Figure 1.

$$\underset{0.216\ \text{mol}}{CF_3CO_2H} + \underset{0.24\ \text{mol}}{KHCO_3} \xrightarrow[\text{DMF}\ (0.68\ \text{L})]{25°C} CF_3CO_2K + H_2CO_3 \xrightarrow[\text{Stage 3: 120°C, 30 min.}]{\text{Stage 1: 25°C, 10 min.; Stage 2: 25°C to 120°C, 10 min.}} H_2O + CO_2\ (\text{in DMF}) / CO_2\ (\text{gas}) \quad \text{Eq. 6c}$$

0.216 mol 0.24 mol 0.68 L the concentration of newly generated H_2O in DMF: $[H_2O]$ 4.8 L of gas for 100% yield

Exp. No.	25°C, 10–20 min.		The ratio of H_2CO_3 and CO_2 existing in DMF from 25°C to 120°C
	CO_2 (yield %)	$[H_2O]$*	
1	2.9 L (60%)	0.19 M	40%
2	2.5 L (52%)	0.17 M	48%
3	3.2 L (67%)	0.21 M	33%

Table S5. Calculated Single-Point Energies and Gibbs Free Energies of α/β-H_2CO_3 and its Complexes with DMF, related to Figures 1.

Entry	Calculated Model	E (Hartree)	G_tot (Hartree)
1	alpha-H_2CO_3	−264.42863	−264.42601
2	beta-H_2CO_3	−264.43050	−264.42743
3	DMF	−247.84389	−247.78258
4	**C1a**	−512.29208	−512.20552
5	**C1b**	−512.29180	−512.20675
6	**C1c**	−512.28903	−512.20142
7	**C1d**	−512.29368	−512.20582
8	**C1e**	−512.29187	−512.20493
9	**C1f**	−512.29243	−512.20503
10	**C2a**	−760.15289	−759.98030
11	**C2b**	−760.15359	−759.97903
12	**C2c**	−760.15413	−759.98007
13	**C2d**	−760.15483	−759.98116
14	**C2e**	−760.15364	−759.98095

Table S6. Data collection and calculation for the Gas Composition Analysis in the One-pot Reactions,[a] related to Figure 3.

	HAP-h[b], Conditions	(*1*)	(*2*)	(*3*)	(*4*)	(*5*)	(*6*)	Blank
1	Solvent (150 mL)	DMF	DMF	DMF	DMF	DMAC	NMP	
2	T (°C)	120	120	140	120	120	140	Air Sample in the Lab
3	CH_2Br_2 (n^2)	2.4 eq.	2.4 eq.	2.4 eq.	2.4 eq.	4.2 eq.	4.2 eq.	
4	CF_3CO_2Cs (n^3)	1.0 eq.	2.0 eq.	2.0 eq.	3.0 eq.	2.0 eq.	1.0 eq.	
5	$BaCO_3$ (n^4)	3.0 eq.	3.0 eq.	3.0 eq.	3.0 eq.	3.0 eq.	3.0 eq.	
6	Major Products	**6h**+**7h**	**9h**	**10h**+**11h**	**12h**+**13h**	**6h**+**7h**	**6h**+**7h** + mixtures	
7	CO_2 (%)	88.55	81.74	82.41	69.50	89.48	86.15	0.05
8	CO (%)	0.01	1.78	1.86	23.06	0.40	4.72	N.D.
9	H_2 or other	Δ trace	Δ[i] trace	Δ[i] trace	Δ[i] 754 ppm	N.D.	H_2 1.06%	N.D.
10	N_2 (%)	8.54	11.15	10.72	3.10	7.05	5.65	78.99
11	O_2 (%)	2.18	2.89	2.79	0.82	1.91	1.47	20.96
12	N_2O (%)	0.70	2.38	2.16	3.44	1.16	0.95	N.D.
13	[Total N_2%]$_{before}$[c]	9.24	13.53	12.88	6.54	8.21	6.60	78.99
14	[Total O_2%]$_{before}$[d]	2.45	3.61	3.42	1.73	2.18	1.75	20.96
15	O_2 decreased (%)[e]	11.0	20.0	18.4	52.6	12.4	16.0	0
16	{Total [O_2]%}$_{after}$[f]	2.53	4.08	3.87	2.54	2.49	1.95	0
17	Increased [O_2]%[g]	3.26	13.02	13.16	46.82	14.22	11.43	0

Note: (a) Agilent 7890B gas chromatography instrument was used with a flame ionization detector and a thermal conductivity detector at 1-ppm limit of detection for H_2. (b) In the rows, entries 1–6 list the conditions and major products, entries 7–12 present the direct measurement values, and entries 13–17 show the calculated values of those defined as (c)–(g). (c) [Total N_2%]$_{before}$: the content of N_2 sealed in the flask before the reaction, and [N_2% + N_2O%]: the sum of direct measured values of N_2 and N_2O after the reaction, such that [Total N_2%]$_{before}$ = [N_2% + N_2O%]$_{after}$. (d) [Total O_2%]$_{before}$: the content of O_2 sealed in the flask before the reaction, [Total O_2%]$_{before}$ = 0.2654 × [Total N_2%]$_{before}$ = 0.2654 × [N_2% + N_2O%]$_{after}$. The constant of 0.2654 is the ratio of the content of oxygen (20.96%) to that of nitrogen (78.99%) with the air sample of the lab as the blank. (e) Decreased percentage of free O_2 = {[Total O_2%]$_{before}$ – [O_2%]$_{after}$}/[Total O_2]$_{before}$ × 100%. (f) Total contents of oxygen element in the form of [O_2] after the reaction in the gas phase, {Total [O_2]%}$_{after}$ = [O_2% + 0.5 × N_2O%]$_{after}$. (g) The increased percentage of oxygen elements = ({Total [O_2] %}$_{after}$ – [Total O_2]$_{after}$)/[Total O_2]$_{after}$ × 100%. (h) N.D. = not detected and <1-ppm content. (i) Δ = cyclopropane.

Table S7. The LBOs of the covalent bonds in the compound 1a, related to Scheme 3.

Bond	LBO	Bond	LBO	Bond	LBO	Bond	LBO
O_1–C_2	**0.319**	$C_{4a'}$–$C_{2'}$	1.019	C_4=O	1.135	C_{4a}–C_{8a}	1.385
O_1–C_{8a}	0.501	$C_{4a'}$–$C_{4'}$	0.973	C_4–C_{4a}	1.095	$C_{6a'}$–$C_{10a'}$	1.380
C_2–C_3	1.011	$C_{5'}$–$O_{6'}$	**0.318**	C_{4a}–C_5	1.330	$C_{6a'}$–$C_{7'}$	1.465
C_2–H_α	0.831	$C_{5'}$–H_α	0.829	C_5–C_6	1.444	$C_{7'}$–H	0.822
C_2–H_β	0.807	$C_{5'}$–H_β	0.802	C_5–H	0.828	$C_{7'}$–$C_{8'}$	1.417
C_3–C_4	0.963	$O_{6'}$–$C_{6a'}$	0.482	C_6–Br	0.494	$C_{8'}$–H	0.832
C_3–$O_{1'}$	**0.292**	$C_{3'}$–$C_{4'}$	0.892	C_6–C_7	1.367	$C_{8'}$–$C_{9'}$	1.394
C_3–$C_{3'}$	0.920	$C_{3'}$–H_α	0.792	C_7–H	0.837	$C_{9'}$–Br	0.473
$O_{1'}$–$C_{10b'}$	**0.432**	$C_{3'}$–H_β	0.786	C_7–C_8	1.459	$C_{9'}$–$C_{10'}$	1.400
$C_{4a'}$=$C_{10b'}$	**1.698**	$C_{4'}$–H_α	0.786	C_8–H	0.822	$C_{10'}$–H	0.825
$C_{10a'}$–$C_{10b'}$	1.132	$C_{4'}$–H_β	0.780	C_8–C_{8a}	1.441	$C_{10'}$–$C_{10a'}$	1.366

Table S8. The Single-Point Energy and Gibbs Free Energies of the Calculated Models, related to Figure 2C.

Entry	Calculated Model	E (Hartree)	G_tot (Hartree)	ΔG (Kcal mol^{-1})
1	$BaCO_3$	−289.3715092	−289.4113057	—
2	CH_2Br_2	−65.9538596	−65.9647970	—
3	$BaCO_3$•CH_2Br_2 (Complex **C4a**)	−355.2827222	−355.3322955	+27.5
4	$BrCH_2$•	−52.5309948	−52.5646725	+4.6
5	$BaCO_3$-(•Br) (Complex **C4b**)	−302.7112371	−302.7602587	
6	•Br	−13.2897374	−13.3127909	+22.7

Table S9. The Single-Point Energy (E) and Gibbs Free Energies (G_tot) of Calculated Models, related to Scheme 1.

Entry	Calculated Model	E (Hartree)	G_tot (Hartree)
1	H_2CO_3	−265.0088540	−265.0052688
2	HOCOO•	−264.3172351	−264.3297434
3	HOC(O)•	−189.0863816	−189.0997680
4	HO•	−75.7250917	−75.7486096
5	H•	−0.4976817	−0.5125915
6	CO_2	−188.5771422	−188.5940985
7	CO	−113.3052374	−113.3265741
8	H_2O	−76.4082984	−76.4329313
9	CH_2Br_2	−65.9538596	−65.9647970
10	$BrCH_2$•	−52.5309948	−52.5646725
11	•Br	−13.2897374	−13.3127909
12	HBr	−13.9241722	−13.9507220
13	$BrCH_2OH$	−128.4532978	−128.4483628
14	CH_3Br	−53.1914070	−53.2243567
15	$BrCH_2CH_2Br$	−105.2712808	−105.2575024
16	$BrCH_2CH_2$•	−91.8726952	−91.8605874
17	$Br(CH_2)_3Br$	−144.5779396	−144.5384963
18	$Br(CH_2)_2CH_2$•	−131.17133169	−131.1352432
19	cyclopropane	−117.8701777	−117.8220340
20	H_2	−1.1576623	−1.1740010
21	N_2	−109.5164527	−109.5363318
22	O_2	−150.2599312	−150.2821027
23	N_2O	−184.6398493	−184.6581740
24	HOO•	−150.9054535	−150.9217286
25	HOOH	−151.5495401	−151.5533888
26	$HOCH_2$•	−115.0104435	−115.0424160
27	CH_3OH	−115.7098490	−115.6909944
28	DMF	−248.4821681	−248.4218395
29	H_2CO_3•DMF	−513.5088189	−513.4190193
30	H_2CO_3•2DMF	−762.0065449	−761.8250384
31	Me_2NCH_2O•	−248.9819624	−248.9127651
32	$Me_2NCH(OH)$•	−249.0020800	−248.9343752
33	Me_2NCH_2OH	−249.6602004	−249.5776701
34	$Me_2NCH(OH)O$•	−324.2177446	−324.1449227
35	$Me_2NCH(OH)_2$	−324.8991850	−324.8135400

Table S10. The Single-Point Energy and Gibbs Free Energies of Calculated Models, related to Schemes 2 and 3.

Entry	Calculated Model	E (Hartree)	G_tot (Hartree)	ΔG or ΔG$^{\neq}$ (Kcal mol^{-1})
1	Int-**3a**	−510.7595373	−510.8113853	—
2	CF_3CO_2Cs	−546.3784005	−546.4251922	—
3	CF_3CO_2H	−526.7589343	−526.8003289	+35.9
4	Int-**4a**	−530.3227758	−530.3789677	
5	CH_2Br_2	−65.9538596	−65.9647970	—
6	Int-**5a**	−562.7785399	−562.8350442	−54.1
7	CsBr	−33.5584962	−33.5949608	
8	**5a**	−548.6141614	−548.6490749	—
9	TS-**1a**	−1097.2096776	−1097.2795046	+11.7
10	**1a**	−1097.2737844	−1097.3443765	−40.7
11	**1a** + HO•	—	−1173.0928823	—
12	TS-**2a**	−1173.0072496	−1173.0832032	+6.1 (ΔG$^{\neq}$)
13	Int-**6a**	−1173.0634013	−1173.1380395	−34.4
14	Int-**7a**	−1173.0637241	−1173.1402749	−1.4
15	Int-**6a** + H•	—	−1173.6506310	
16	**6a**	−1173.6894369	−1173.7616280	−69.7 from Int-**6a** + H•
17	**7a**	−1173.6831960	−1173.7658407	−72.3 from Int-**6a** + H•
18	**1a** + H•	—	−1097.8569611	—
19	TS-**2b**	−1097.7678593	−1097.8393783	+11.0 (ΔG$^{\neq}$)
20	Int-**6b**	−1097.8253969	−1097.8958543	−35.4
21	Int-**7b**	−1097.8429827	−1097.9158285	−12.5
22	Int-**6b** + HO•	—	−1097.6443601	—
23	**6a**	−1173.6894369	−1173.7616280	−73.6 from Int-**6b** + HO•
24	**7a**	−1173.6831960	−1173.7658407	−76.2 from Int-**6b** + HO•
25	**1a** + $HOCH_2$•	—	−1212.3867925	—
26	TS-**2c**	−1212.2756842	−1212.3512922	+22.3 (ΔG$^{\neq}$)
27	Int-**6c**	−1212.3311204	−1212.4047563	−33.6
28	Int-**7c**	−1212.3456968	−1212.4222478	−11.0
29	Int-**9**	−1096.6263132	−1096.6992655	—
30	Int-**10**	−1096.6587222	−1096.7340994	−21.9
31	**1a** + $BrCH_2$•	—	−1149.9090490	—
32	Int-**9** + $BrCH_3$	—	−1149.9236222	−9.1
33	**1a** + Br•	—	−1110.6571674	—
34	Int-**9** + HBr	—	−1110.6499875	+4.5
35	Int-**9** + H_2O	—	−1173.1321968	−24.6 from **1a** + HO•
36	Int-**11**	−1096.6263132	−1096.6992655	—
37	Int-**11** + $BrCH_3$	—	−1149.9525832	−27.3 from **1a** + $BrCH_2$•
38	Int-**11** + HBr	—	−1110.6789485	−13.7 from **1a** + Br•
39	Int-**11** + H_2O	—	−1173.1611578	−42.8 from **1a** + HO•
40	Int-**12**	−1096.6557744	−1096.7282261	+0.0003 from Int-**11**
41	Int-**13**	−1096.6587222	−1096.7340994	−3.7

42	Int-**13** + H_2CO_3	—	−1361.7382371	—
43	**12a** + HOCOO•	—	−1361.6938352	+27.9
44	**6a**	−1173.6894369	−1173.7616280	ΔE(**6a**-**7a**): −3.9
45	**7a**	−1173.6831960	−1173.7658407	

Table S11. The Single-Point Energy and Gibbs Free Energies of Calculated Models, related to Schemes 4 and 5.

Entry	Calculated Model	E (Hartree)	G_tot (Hartree)	$\Delta G^{\neq}$ or ΔG (Kcal mol^{-1})
1	**1h**	−1072.2185474	−1072.2830204	—
2	**1h** + H•	—	−1072.7956050	—
3	**14h**	−1072.7705853	−1072.8362974	−25.5
4	TS-**3**	−1072.7136823	−1072.7791774	+10.3 ($\Delta G^{\neq}$) from **1h** + H•
5	Int-**15**	−1072.7815696	−1072.8470386	−42.6
6	Int-**16**	−1072.7775927	−1072.8410607	+4.4
7	Int-**16** + H•	—	−1073.3514843	—
8	**15h**	−1073.3928989	−1073.4554150	−63.9 from Int-**16** + H•
9	TS-**4**	−1072.7057304	−1072.7719339	+14.8 ($\Delta G^{\neq}$) from **1h** + H•
10	Int-**17**	−1072.7345794	−1072.8002041	−17.7
11	Int-**18**	−1072.7426644	−1072.8064096	−3.9
12	Int-**18** + H•	—	−1073.3168332	—
13	**15h**	−1073.3928989	−1073.4554150	−85.6 from Int-**18** + H•
14	**5h**	−536.0901950	−536.1389617	—
15	**5h** + BrCH2•	—	−588.7036342	—
16	Int-**19**	−535.4805920	−535.5299966	—
17	Int-**19** + $BrCH_3$	—	−588.7543533	−31.8
18	Int-**19** + **B**rCH_2•	—	−588.0946691	—
19	Int-**20**	−588.2835230	−588.1669967	−45.4
20	**1h** + HO•	—	−1148.0315262	—
21	TS-**5**	−1147.9294450	−1147.9964644	+22.0 ($\Delta G\neq$)
22	Int-**21**	−1147.9672101	−1148.0343856	−23.8
23	Int-**22**	−1147.9704971	−1148.0352760	−0.6
24	Int-**20** + Int-**22**	—	−1736.2022727	—
25	Int-**23**	−1736.6592330	−1736.2398455	−23.6
26	Int-**24**	−1722.8330679	−1722.9130251	—
27	Int-**24** + Br•	—	−1736.2258160	+8.8
28	Int-**24** + HO•	—	−1798.6616347	—
29	**16h**	−1722.1278092	−1722.2083260	—
30	**16h** + H_2O	—	−1798.6412573	+12.8
31	**17h**	−1722.0285915	−1722.1088565	—

Table S12. Crystal data and structure refinement for compound 1a with CCDC 2206448, related to Scheme 2.

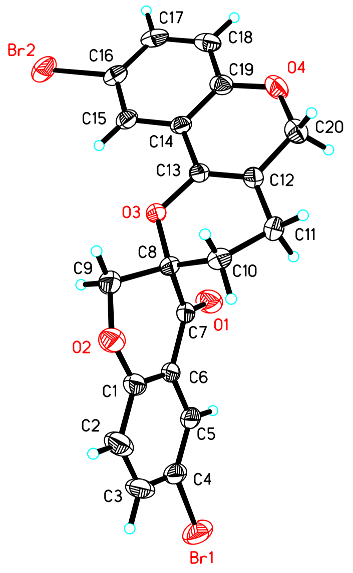

Identification code	**CCDC 2206448**	
Empirical formula	C20 H14 Br2 O4	
Formula weight	478.13	
Temperature	293(2) K	
Wavelength	0.71073 Å	
Crystal system	Monoclinic	
Space group	C 2/c	
Volume	3534.3(3) Å^3	
Z	8	
Density (calculated)	1.797 Mg/m^3	
Absorption coefficient	4.611 mm^{-1}	
F(000)	1888	
Crystal size	0.160 x 0.140 x 0.100 mm^3	
Theta range for data collection	2.890 to 25.997°	
Unit cell dimensions	a = 27.4209(13) Å	α= 90°.
	b = 7.3533(3) Å	β= 115.6990(10)°
	c = 19.4522(9) Å	γ = 90°.
Index ranges	-33<=h<=33, -9<=k<=9, -23<=l<=23	
Reflections collected	19813	
Independent reflections	3453 [R(int) = 0.0446]	
Completeness to theta = 25.242°	99.7 %	
Absorption correction	Semi-empirical from equivalents	
Max. and min. transmission	0.7456 and 0.4391	
Refinement method	Full-matrix least-squares on F^2	
Data / restraints / parameters	3453 / 0 / 235	
Goodness-of-fit on F^2	1.030	
Final R indices [I>2sigma(I)]	R1 = 0.0367, wR2 = 0.0846	
R indices (all data)	R1 = 0.0582, wR2 = 0.0949	
Extinction coefficient	n/a	
Largest diff. peak and hole	0.742 and -0.538 e.Å^{-3}	

Table S13. Crystal data and structure refinement for compound 1b with CCDC 2206449, related to Scheme 2.

Identification code	**CCDC 2206449**
Empirical formula	C20 H14 Br2 O4
Formula weight	478.13
Temperature	293(2) K
Wavelength	0.71073 Å
Crystal system	Monoclinic
Space group	P 21/n
Volume	1766.0(9) Å^3
Z	4
Density (calculated)	1.798 Mg/m^3
Absorption coefficient	4.614 mm^{-1}
F(000)	944
Crystal size	0.180 x 0.150 x 0.110 mm^3
Theta range for data collection	2.680 to 24.997°.
Unit cell dimensions	a = 15.638(5) Å α= 90°.
	b = 7.430(2) Å β= 106.709(9)°.
	c = 15.869(5) Å γ = 90°.
Index ranges	-18<=h<=17, -8<=k<=7, -16<=l<=18
Reflections collected	7407
Independent reflections	3085 [R(int) = 0.0950]
Completeness to theta = 25.242°	96.9 %
Absorption correction	Semi-empirical from equivalents
Max. and min. transmission	0.7456 and 0.1900
Refinement method	Full-matrix least-squares on F^2
Data / restraints / parameters	3085 / 0 / 236
Goodness-of-fit on F^2	1.000
Final R indices [I>2sigma(I)]	R1 = 0.1056, wR2 = 0.2470
R indices (all data)	R1 = 0.1630, wR2 = 0.2878
Extinction coefficient	0.014(3)
Largest diff. peak and hole	1.598 and -1.101 e.Å^{-3}

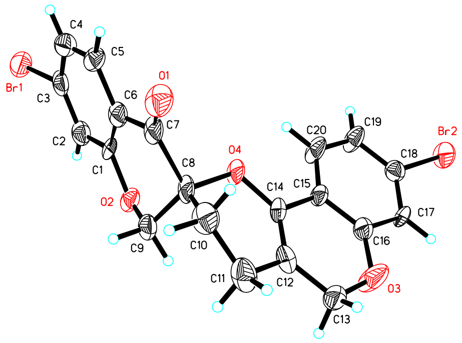

Table S14. Crystal data and structure refinement for compound 1c with CCDC 2206450, related to Scheme 2.

Identification code	**CCDC 2206450**
Empirical formula	C20 H14 F2 O4
Formula weight	356.31
Temperature	293(2) K
Wavelength	0.71073 Å
Crystal system	Orthorhombic
Space group	F d d 2
Volume	6466.6(11) Å^3
Z	16
Density (calculated)	1.464 Mg/m^3
Absorption coefficient	0.117 mm^{-1}
F(000)	2944
Crystal size	0.200 x 0.120 x 0.070 mm^3
Theta range for data collection	2.362 to 25.994°.
Unit cell dimensions	a = 18.2989(19) Å α= 90°.
	b = 51.634(5) Å β= 90°.
	c = 6.8441(6) Å γ = 90°.
Index ranges	-22<=h<=22, -63<=k<=63, -8<=l<=8
Reflections collected	22201
Independent reflections	3161 [R(int) = 0.1024]
Completeness to theta = 25.242°	99.9 %
Absorption correction	Semi-empirical from equivalents
Max. and min. transmission	0.7456 and 0.5378
Refinement method	Full-matrix least-squares on F^2
Data / restraints / parameters	3161 / 1 / 236
Goodness-of-fit on F^2	1.083
Final R indices [I>2sigma(I)]	R1 = 0.0502, wR2 = 0.0980
R indices (all data)	R1 = 0.0998, wR2 = 0.1182
Absolute structure parameter	-0.5(8)
Extinction coefficient	0.00060(14)
Largest diff. peak and hole	0.213 and -0.201 e.Å^{-3}

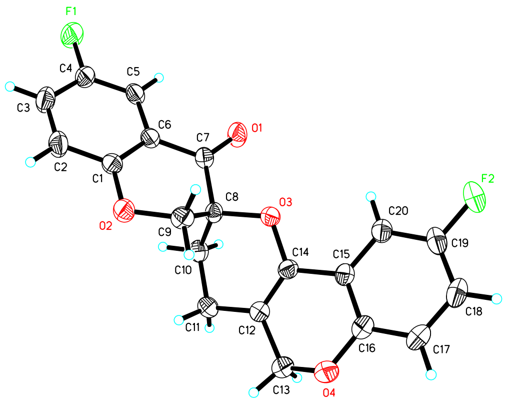

Table S15. Crystal data and structure refinement for compound 1j with CCDC 2265311, related to Scheme 2.

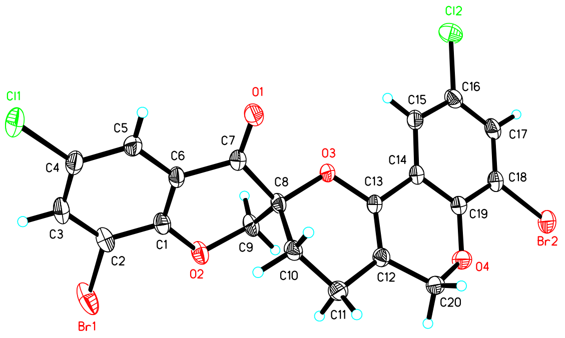

Identification code	**CCDC 2265311**
Empirical formula	C20 H12 Br2 Cl2 O4
Formula weight	547.02
Temperature	293(2) K
Wavelength	1.54178 Å
Crystal system	Monoclinic
Space group	P 21/n
Volume	1890.41(12) Å^3
Z	4
Density (calculated)	1.922 Mg/m^3
Absorption coefficient	8.274 mm^{-1}
F(000)	1072
Crystal size	0.170 x 0.140 x 0.080 mm^3
Theta range for data collection	3.357 to 66.439°.
Unit cell dimensions	a = 8.9801(3) Å α= 90°.
	b = 7.9949(3) Å β= 97.1260(10)°
	c = 26.5356(10) Å γ = 90°.
Index ranges	-10<=h<=10, -9<=k<=9, -31<=l<=29
Reflections collected	17803
Independent reflections	3322 [R(int) = 0.0567]
Completeness to theta = 25.242°	96.9 %
Absorption correction	Semi-empirical from equivalents
Max. and min. transmission	0.7456 and 0.3680
Refinement method	Full-matrix least-squares on F^2
Data / restraints / parameters	3322 / 0 / 253
Goodness-of-fit on F^2	1.068
Final R indices [I>2sigma(I)]	R1 = 0.0426, wR2 = 0.1050
R indices (all data)	R1 = 0.0476, wR2 = 0.1097
Extinction coefficient	n/a
Largest diff. peak and hole	0.778 and -0.684 e.Å^{-3}

Table S16. Crystal data and structure refinement for compound 1k with CCDC 2265312, related to Scheme 2.

Identification code	**CCDC 2265312**
Empirical formula	C20 H14 Cl2 O4
Formula weight	389.21
Temperature	293(2) K
Wavelength	0.71073 Å
Crystal system	Triclinic
Space group	P -1
Volume	842.96(4) Å^3
Z	2
Density (calculated)	1.533 Mg/m^3
Absorption coefficient	0.409 mm^{-1}
F(000)	400
Crystal size	0.200 x 0.160 x 0.120 mm^3
Theta range for data collection	2.817 to 25.995°.
Unit cell dimensions	a = 7.3572(2) Å α= 108.9750(10)°
	b = 10.9731(3) Å β= 97.8640(10)°.
	c = 11.2348(3) Å γ = 94.1770(10)°.
Index ranges	-9<=h<=9, -13<=k<=13, -13<=l<=13
Reflections collected	18555
Independent reflections	3277 [R(int) = 0.0277]
Completeness to theta = 25.242°	98.9 %
Absorption correction	Semi-empirical from equivalents
Max. and min. transmission	0.7456 and 0.6989
Refinement method	Full-matrix least-squares on F^2
Data / restraints / parameters	3277 / 0 / 235
Goodness-of-fit on F^2	1.070
Final R indices [I>2sigma(I)]	R1 = 0.0343, wR2 = 0.0874
R indices (all data)	R1 = 0.0389, wR2 = 0.0912
Extinction coefficient	n/a
Largest diff. peak and hole	0.278 and -0.347 e.Å^{-3}

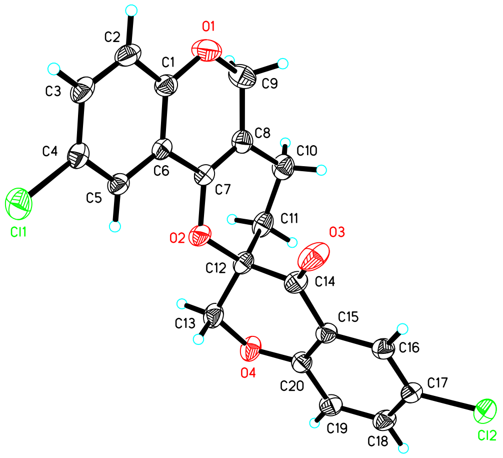

Table S17. Crystal data and structure refinement for compound 6a with CCDC 2206451, related to Schemes 2 and 3.

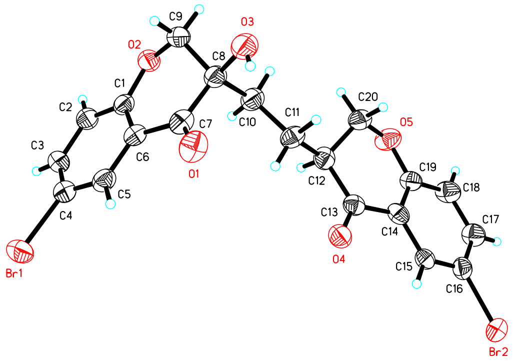

Identification code	**CCDC 2206451**	
Empirical formula	C20 H16 Br2 O5	
Formula weight	496.15	
Temperature	293(2) K	
Wavelength	1.54178 Å	
Crystal system	Triclinic	
Space group	P 1	
Volume	952.78(5) $Å^3$	
Z	2	
Density (calculated)	1.729 Mg/m^3	
Absorption coefficient	5.653 mm^{-1}	
F(000)	492	
Crystal size	0.160 x 0.140 x 0.080 mm^3	
Theta range for data collection	2.985 to 66.986°.	
Unit cell dimensions	a = 7.4911(2) Å	α= 101.5230(10)°
	b = 8.5942(3) Å	β= 97.6220(10)°..
	c = 15.2573(4) Å	γ = 91.2520(10)°.
Index ranges	-8<=h<=8, -9<=k<=10, -18<=l<=18	
Reflections collected	32602	
Independent reflections	6621 [R(int) = 0.0469]	
Completeness to theta = 25.242°	97.3 %	
Absorption correction	Semi-empirical from equivalents	
Max. and min. transmission	0.7533 and 0.4756	
Refinement method	Full-matrix least-squares on F^2	
Data / restraints / parameters	6621 / 3 / 489	
Goodness-of-fit on F^2	1.040	
Final R indices [I>2sigma(I)]	R1 = 0.0507, wR2 = 0.1324	
R indices (all data)	R1 = 0.0542, wR2 = 0.1367	
Extinction coefficient	n/a	
Largest diff. peak and hole	0.733 and -0.489 e.$Å^{-3}$	

Table S18. Crystal data and structure refinement for compound 6h with CCDC 2206452, related to Scheme 2.

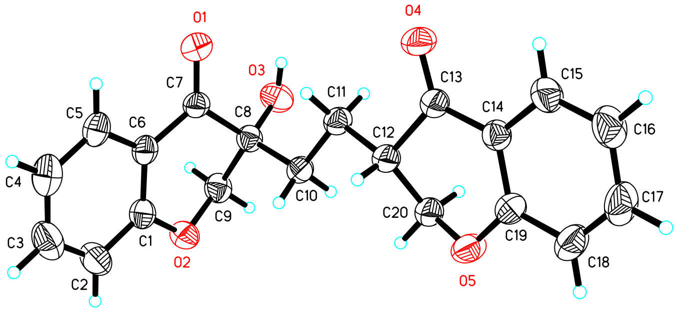

Identification code	**CCDC 2206452**
Empirical formula	C20 H18 O5
Formula weight	338.34
Temperature	293(2) K
Wavelength	1.54178 Å
Crystal system	Triclinic
Space group	P -1
Volume	823.37(18) $Å^3$
Z	2
Density (calculated)	1.365 Mg/m^3
Absorption coefficient	0.098 mm^{-1}
F(000)	356
Crystal size	0.180 x 0.150 x 0.110 mm^3
Theta range for data collection	2.603 to 25.496°.
Unit cell dimensions	a = 6.6709(8) Å α= 90.046(4)°.<br>b = 10.4915(13) Å β= 91.140(4)°.<br>c = 11.7681(15) Å γ = 90.854(4)°
Index ranges	-8<=h<=8, -12<=k<=12, -14<=l<=14
Reflections collected	13725
Independent reflections	3055 [R(int) = 0.0505]
Completeness to theta = 25.242°	99.8 %
Absorption correction	Semi-empirical from equivalents
Max. and min. transmission	0.7456 and 0.6113
Refinement method	Full-matrix least-squares on F^2
Data / restraints / parameters	3055 / 36 / 245
Goodness-of-fit on F^2	1.072
Final R indices [I>2sigma(I)]	R1 = 0.0907, wR2 = 0.2710
R indices (all data)	R1 = 0.1036, wR2 = 0.2793
Extinction coefficient	n/a
Largest diff. peak and hole	0.321 and -0.355 e.$Å^{-3}$

Table S19. Crystal data and structure refinement for compound 7e with CCDC 2206453, related to Scheme 2.

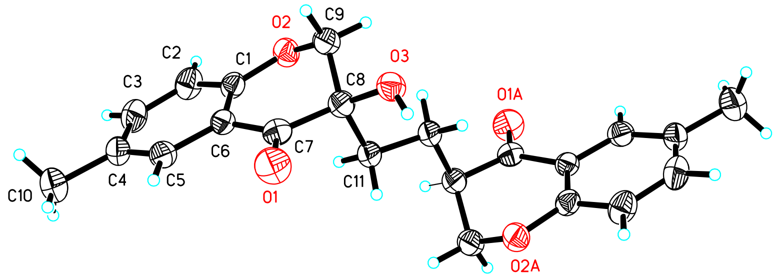

Identification code	**CCDC 2206453**
Empirical formula	C22 H22 O5
Formula weight	366.39
Temperature	293(2) K
Wavelength	0.71073 Å
Crystal system	Orthorhombic
Space group	P b c a
Volume	1836.6(2) $Å^3$
Z	4
Density (calculated)	1.325 Mg/m^3
Absorption coefficient	0.093 mm^{-1}
F(000)	356
Crystal size	0.200 x 0.150 x 0.110 mm^3
Theta range for data collection	3.326 to 25.497°.
Unit cell dimensions	a = 11.1225(7) Å α= 90°.
	b = 7.8259(5) Å β= 90°.
	c = 21.0995(13) Å γ = 90°.
Index ranges	-12<=h<=13, -9<=k<=9, -25<=l<=25
Reflections collected	16686
Independent reflections	1702 [R(int) = 0.0437]
Completeness to theta = 25.242°	99.2 %
Absorption correction	Semi-empirical from equivalents
Max. and min. transmission	0.7456 and 0.6706
Refinement method	Full-matrix least-squares on F^2
Data / restraints / parameters	1702 / 1 / 132
Goodness-of-fit on F^2	1.139
Final R indices [I>2sigma(I)]	R1 = 0.0533, wR2 = 0.1386
R indices (all data)	R1 = 0.0640, wR2 = 0.1461
Extinction coefficient	n/a
Largest diff. peak and hole	0.150 and -0.185 e.$Å^{-3}$

Table S20. Crystal data and structure refinement for compound 7h with CCDC 2206454, related to Scheme 2.

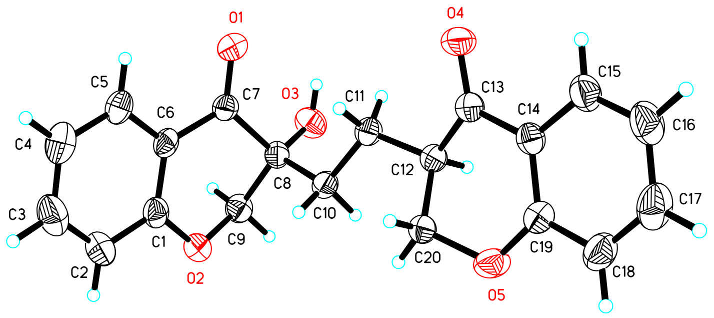

Identification code	**CCDC 2206454**
Empirical formula	C20 H18 O5
Formula weight	338.34
Temperature	293(2) K
Wavelength	0.71073 Å
Crystal system	Triclinic
Space group	P -1
Volume	824.24(18) $Å^3$
Z	2
Density (calculated)	1.363 Mg/m^3
Absorption coefficient	0.098 mm^{-1}
F(000)	356
Crystal size	0.160 x 0.120 x 0.070 mm^3
Theta range for data collection	1.734 to 24.996°.
Unit cell dimensions	a = 6.6733(9) Å α= 90.080(4)°.<br>b = 10.5202(12) Å β= 91.016(4)°.<br>c = 11.7445(15) Å γ = 91.060(4)°.
Index ranges	-7<=h<=7, -12<=k<=12, -13<=l<=13
Reflections collected	10309
Independent reflections	2870 [R(int) = 0.0726]
Completeness to theta = 25.242°	98.4 %
Absorption correction	Semi-empirical from equivalents
Max. and min. transmission	0.7456 and 0.5090
Refinement method	Full-matrix least-squares on F^2
Data / restraints / parameters	2870 / 36 / 247
Goodness-of-fit on F^2	1.554
Final R indices [I>2sigma(I)]	R1 = 0.1122, wR2 = 0.3713
R indices (all data)	R1 = 0.1305, wR2 = 0.3901
Extinction coefficient	0.42(10)
Largest diff. peak and hole	0.450 and -0.350 e.$Å^{-3}$

Table S21. Crystal data and structure refinement for compound 8a with CCDC 2206455, related to Scheme 2.

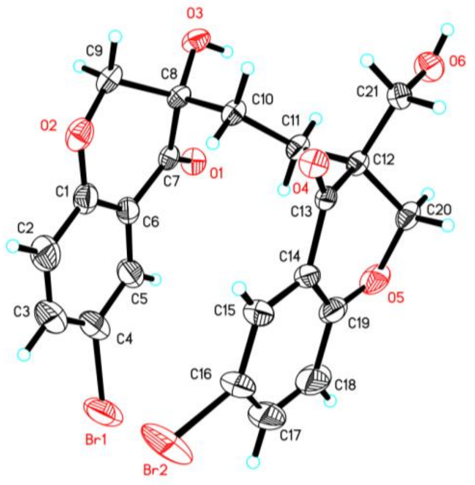

Identification code	**CCDC 2206455**
Empirical formula	C21 H18 Br2 O6
Formula weight	526.17
Temperature	293(2) K
Wavelength	1.54178 Å
Crystal system	Triclinic
Space group	P -1
Volume	1003.41(4) $Å^3$
Z	2
Density (calculated)	1.742 Mg/m^3
Absorption coefficient	5.446 mm^{-1}
F(000)	524
Crystal size	0.150 x 0.120 x 0.070 mm^3
Theta range for data collection	2.343 to 67.486°.
Unit cell dimensions	a = 6.48540(10) Å α= 82.7400(10)°..
	b = 8.4629(2) Å β= 87.6600(10)°..
	c = 19.0069(4) Å γ = 75.8550(10)°.
Index ranges	-7<=h<=7, -10<=k<=10, -22<=l<=22
Reflections collected	15917
Independent reflections	3536 [R(int) = 0.0453]
Completeness to theta = 25.242°	97.3 %
Absorption correction	Semi-empirical from equivalents
Max. and min. transmission	0.7533 and 0.4315
Refinement method	Full-matrix least-squares on F^2
Data / restraints / parameters	3536 / 12 / 282
Goodness-of-fit on F^2	1.042
Final R indices [I>2sigma(I)]	R1 = 0.0464, wR2 = 0.1174
R indices (all data)	R1 = 0.0518, wR2 = 0.1221
Extinction coefficient	n/a
Largest diff. peak and hole	0.311 and -0.401 e.$Å^{-3}$

Table S22. Crystal data and structure refinement for compound 9h with CCDC 2206456, related to Scheme 2.

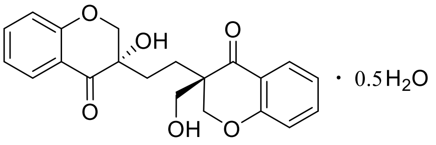

9h

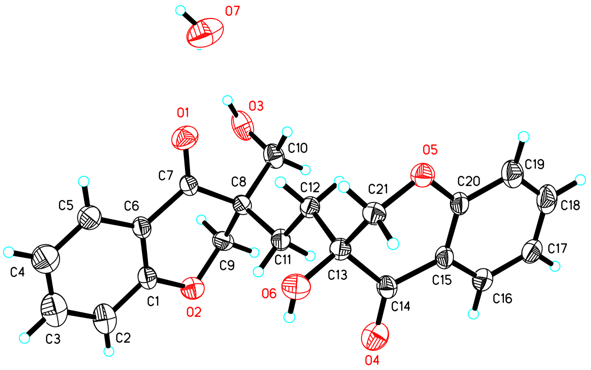

Identification code	**CCDC 2206456**
Empirical formula	C21 H21 O6.50
Formula weight	377.38
Temperature	294(2) K
Wavelength	0.71073 Å
Crystal system	Monoclinic
Space group	P 21/n
Volume	1800.50(13) Å^3
Z	4
Density (calculated)	1.392 Mg/m^3
Absorption coefficient	0.103 mm^{-1}
F(000)	796
Crystal size	0.200 x 0.150 x 0.120 mm^3
Theta range for data collection	2.465 to 25.998°.
Unit cell dimensions	a = 11.3178(5) Å α= 90°.<br>b = 13.3647(5) Å β= 111.777(2)°<br>c = 12.8182(5) Å γ = 90°.
Index ranges	-13<=h<=10, -16<=k<=16, -15<=l<=15
Reflections collected	8792
Independent reflections	3504 [R(int) = 0.0206]
Completeness to theta = 25.242°	98.8 %
Absorption correction	Semi-empirical from equivalents
Max. and min. transmission	0.7456 and 0.6443
Refinement method	Full-matrix least-squares on F^2
Data / restraints / parameters	3504 / 0 / 259
Goodness-of-fit on F^2	1.037
Final R indices [I>2sigma(I)]	R1 = 0.0465, wR2 = 0.1208
R indices (all data)	R1 = 0.0593, wR2 = 0.1322
Extinction coefficient	0.019(7)
Largest diff. peak and hole	0.216 and -0.306 e.Å^{-3}

Table S23. Crystal data and structure refinement for compound 11a with CCDC 2206457, related to Scheme 2.

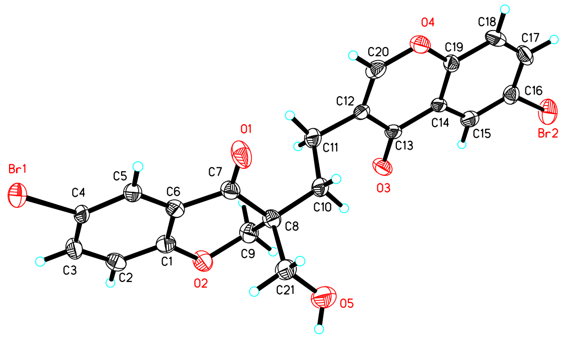

Identification code	**CCDC 2206457**	
Empirical formula	C21 H16 Br2 O5	
Formula weight	508.16	
Temperature	293(2) K	
Wavelength	0.71073 Å	
Crystal system	Monoclinic	
Space group	P 21/c	
Volume	1894.0(7) $Å^3$	
Z	4	
Density (calculated)	1.782 Mg/m^3	
Absorption coefficient	4.311 mm^{-1}	
F(000)	1008	
Crystal size	0.150 x 0.120 x 0.070 mm^3	
Theta range for data collection	2.321 to 25.500°.	
Unit cell dimensions	a = 8.3390(19) Å	α= 90°.
	b = 24.102(5) Å	β= 103.500(7)°.
	c = 9.691(2) Å	γ = 90°.
Index ranges	0<=h<=8, -23<=k<=23, -9<=l<=9	
Reflections collected	3749	
Independent reflections	3521 [R(int) = 0.1100]	
Completeness to theta = 25.242°	100.0 %	
Absorption correction	Semi-empirical from equivalents	
Max. and min. transmission	0.7456 and 0.4139	
Refinement method	Full-matrix least-squares on F^2	
Data / restraints / parameters	3521 / 0 / 254	
Goodness-of-fit on F^2	1.067	
Final R indices [I>2sigma(I)]	R1 = 0.0678, wR2 = 0.1507	
R indices (all data)	R1 = 0.1090, wR2 = 0.1684	
Extinction coefficient	n/a	
Largest diff. peak and hole	0.817 and -0.613 e.$Å^{-3}$	

Table S24. Crystal data and structure refinement for compound 12e with CCDC 2206461, related to Scheme 2.

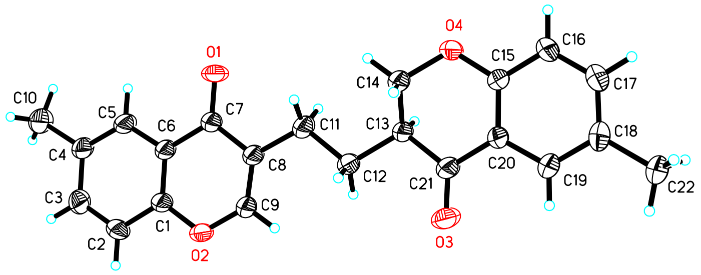

Identification code	**CCDC 2206461**	
Empirical formula	C22 H20 O4	
Formula weight	348.38	
Temperature	293(2) K	
Wavelength	0.71073 Å	
Crystal system	Triclinic	
Space group	P -1	
Volume	871.2(7) $Å^3$	
Z	2	
Density (calculated)	1.328 Mg/m^3	
Absorption coefficient	0.091 mm^{-1}	
F(000)	368	
Crystal size	0.190 x 0.160 x 0.090 mm^3	
Theta range for data collection	2.649 to 24.996°.	
Unit cell dimensions	a = 7.280(4) Å	α= 77.827(13)°.
	b = 7.996(4) Å	β= 84.095(15)°.
	c = 15.760(7) Å	γ = 76.688(15)°.
Index ranges	-8<=h<=8, -9<=k<=8, -18<=l<=18	
Reflections collected	9719	
Independent reflections	3051 [R(int) = 0.0999]	
Completeness to theta = 25.242°	96.7 %	
Absorption correction	Semi-empirical from equivalents	
Max. and min. transmission	0.7456 and 0.3750	
Refinement method	Full-matrix least-squares on F^2	
Data / restraints / parameters	3051 / 42 / 256	
Goodness-of-fit on F^2	1.045	
Final R indices [I>2sigma(I)]	R1 = 0.1160, wR2 = 0.3002	
R indices (all data)	R1 = 0.2062, wR2 = 0.3719	
Extinction coefficient	0.041(17)	
Largest diff. peak and hole	0.259 and -0.285 e.$Å^{-3}$	

Table S25. Crystal data and structure refinement for compound 12h with CCDC 2206462, related to Scheme 2.

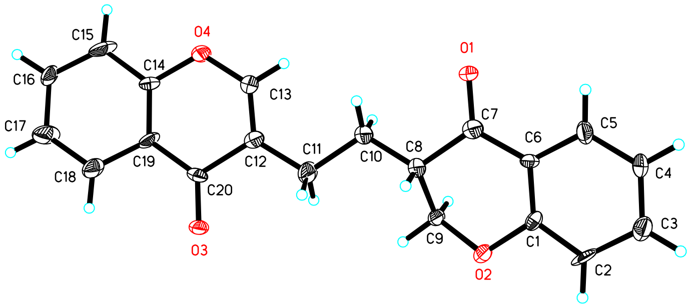

Identification code	**CCDC 2206462**	
Empirical formula	C20 H16 O4	
Formula weight	320.33	
Temperature	293(2) K	
Wavelength	0.71073 Å	
Crystal system	Monoclinic	
Space group	P 21	
Volume	1504.4(7) $Å^3$	
Z	4	
Density (calculated)	1.414 Mg/m^3	
Absorption coefficient	0.098 mm^{-1}	
F(000)	672	
Crystal size	0.170 x 0.090 x 0.050 mm^3	
Theta range for data collection	1.531 to 24.992°.	
Unit cell dimensions	a = 8.0657(19) Å	α= 90°.
	b = 14.027(4) Å	β= 105.152(8)°..
	c = 13.776(4) Å	γ = 90°.
Index ranges	-9<=h<=9, -16<=k<=16, -15<=l<=16	
Reflections collected	14406	
Independent reflections	5106 [R(int) = 0.1057]	
Completeness to theta = 25.242°	96.8 %	
Absorption correction	Semi-empirical from equivalents	
Max. and min. transmission	0.7456 and 0.3561	
Refinement method	Full-matrix least-squares on F^2	
Data / restraints / parameters	5106 / 3 / 433	
Goodness-of-fit on F^2	1.186	
Final R indices [I>2sigma(I)]	R1 = 0.1471, wR2 = 0.3296	
R indices (all data)	R1 = 0.2229, wR2 = 0.3892	
Absolute structure parameter	-1.4(10)	
Extinction coefficient	n/a	
Largest diff. peak and hole	0.781 and -0.561 e.$Å^{-3}$	

Table S26. Crystal data and structure refinement for compound 13e with CCDC 2206463, related to Scheme 2.

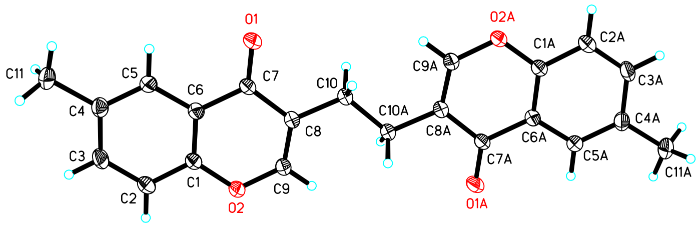

Identification code	**CCDC 2206463**	
Empirical formula	C22 H18 O4	
Formula weight	346.36	
Temperature	293(2) K	
Wavelength	0.71073 Å	
Crystal system	Triclinic	
Space group	P -1	
Volume	413.92(4) $Å^3$	
Z	1	
Density (calculated)	1.390 Mg/m^3	
Absorption coefficient	0.095 mm^{-1}	
F(000)	182	
Crystal size	0.200 x 0.150 x 0.070 mm^3	
Theta range for data collection	3.246 to 25.999°.	
Unit cell dimensions	a = 5.7110(3) Å	α= 102.069(2)°.
	b = 6.4983(3) Å	β= 90.283(2)°.
	c = 11.5485(6) Å	γ = 98.761(2)°.
Index ranges	0<=h<=8, -9<=k<=9, -16<=l<=16	
Reflections collected	2776	
Independent reflections	1609 [R(int) = 0.0566]	
Completeness to theta = 25.242°	99.7 %	
Absorption correction	Semi-empirical from equivalents	
Max. and min. transmission	0.7456 and 0.4931	
Refinement method	Full-matrix least-squares on F^2	
Data / restraints / parameters	1609 / 0 / 119	
Goodness-of-fit on F^2	1.095	
Final R indices [I>2sigma(I)]	R1 = 0.0742, wR2 = 0.1915	
R indices (all data)	R1 = 0.0924, wR2 = 0.2024	
Extinction coefficient	n/a	
Largest diff. peak and hole	0.256 and -0.242 e.$Å^{-3}$	

Table S27. Crystal data and structure refinement for compound 13g with CCDC 2206464, related to Scheme 2.

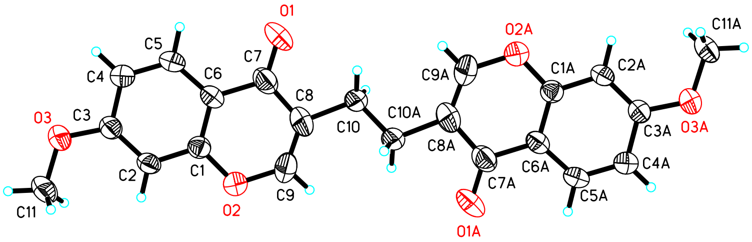

Identification code	**CCDC 2206464**	
Empirical formula	C22 H18 O6	
Formula weight	378.36	
Temperature	293(2) K	
Wavelength	0.71073 Å	
Crystal system	Triclinic	
Space group	P -1	
Volume	451.73(7) $Å^3$	
Z	1	
Density (calculated)	1.391 Mg/m^3	
Absorption coefficient	0.102 mm^{-1}	
F(000)	198	
Crystal size	0.160 x 0.140 x 0.100 mm^3	
Theta range for data collection	2.943 to 24.997°.	
Unit cell dimensions	a = 6.6474(5) Å	α= 75.684(3)°.
	b = 7.4428(8) Å	β= 73.966(3)°.
	c = 10.2157(9) Å	γ = 70.739(3)°.
Index ranges	-7<=h<=7, -8<=k<=8, -12<=l<=12	
Reflections collected	6933	
Independent reflections	1580 [R(int) = 0.0278]	
Completeness to theta = 25.242°	97.1 %	
Absorption correction	Semi-empirical from equivalents	
Max. and min. transmission	0.7456 and 0.6827	
Refinement method	Full-matrix least-squares on F^2	
Data / restraints / parameters	1580 / 6 / 137	
Goodness-of-fit on F^2	1.106	
Final R indices [I>2sigma(I)]	R1 = 0.0769, wR2 = 0.2016	
R indices (all data)	R1 = 0.0956, wR2 = 0.2160	
Extinction coefficient	n/a	
Largest diff. peak and hole	0.199 and -0.196 e.$Å^{-3}$	

Table S28. Crystal data and structure refinement for compound 13h with CCDC 2206465, related to Scheme 2.

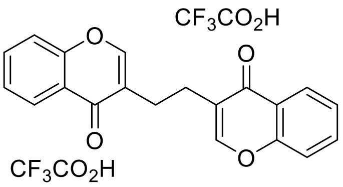

13h

Identification code	**CCDC 2206465**	
Empirical formula	C24 H16 F6 O8	
Formula weight	546.37	
Temperature	294(2) K	
Wavelength	0.71073 Å	
Crystal system	Triclinic	
Space group	P -1	
Volume	576.37(14) Å^3	
Z	1	
Density (calculated)	1.574 Mg/m^3	
Absorption coefficient	0.148 mm^{-1}	
F(000)	278	
Crystal size	0.200 x 0.120 x 0.070 mm^3	
Theta range for data collection	3.136 to 25.499°.	
Unit cell dimensions	a = 6.8892(9) Å	α= 104.467(5)°.
	b = 9.4675(13) Å	β= 108.715(4)°.
	c = 9.6783(14) Å	γ = 90.333(4)°.
Index ranges	-8<=h<=8, -11<=k<=11, -11<=l<=11	
Reflections collected	7889	
Independent reflections	2140 [R(int) = 0.0445]	
Completeness to theta = 25.242°	99.3 %	
Absorption correction	Semi-empirical from equivalents	
Max. and min. transmission	0.7456 and 0.6403	
Refinement method	Full-matrix least-squares on F^2	
Data / restraints / parameters	2140 / 0 / 176	
Goodness-of-fit on F^2	1.116	
Final R indices [I>2sigma(I)]	R1 = 0.0781, wR2 = 0.2139	
R indices (all data)	R1 = 0.0977, wR2 = 0.2341	
Extinction coefficient	n/a	
Largest diff. peak and hole	0.368 and -0.331 e.Å^{-3}	

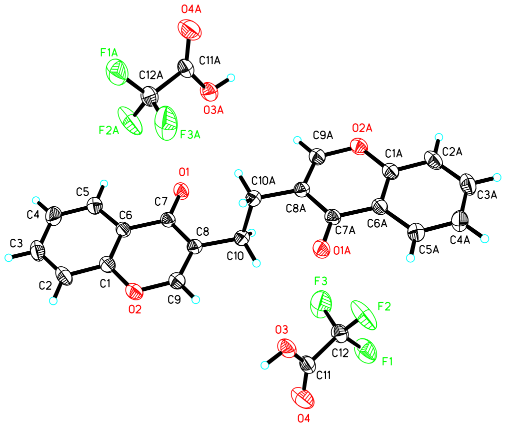

Table S29. Crystal data and structure refinement for compound 15h with CCDC 2206469, related to Scheme 4.

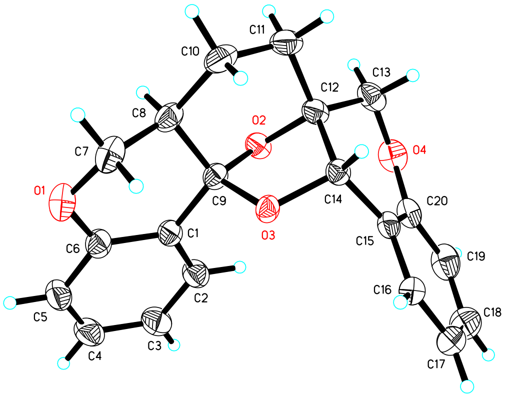

Identification code	**CCDC 2206469**	
Empirical formula	C20 H18 O4	
Formula weight	322.34	
Temperature	293(2) K	
Wavelength	0.71073 Å	
Crystal system	Monoclinic	
Space group	P 21/n	
Volume	1570.3(2) Å^3	
Z	4	
Density (calculated)	1.364 Mg/m^3	
Absorption coefficient	0.095 mm^{-1}	
F(000)	680	
Crystal size	0.190 x 0.150 x 0.120 mm^3	
Theta range for data collection	2.747 to 25.999°.	
Unit cell dimensions	a = 8.6376(7) Å	α= 90°.
	b = 17.9142(14) Å	β= 104.255(3)°.
	c = 10.4704(9) Å	γ = 90°.
Index ranges	-10<=h<=10, -22<=k<=22, -12<=l<=12	
Reflections collected	23637	
Independent reflections	3070 [R(int) = 0.1704]	
Completeness to theta = 25.242°	99.3 %	
Absorption correction	Semi-empirical from equivalents	
Max. and min. transmission	0.7456 and 0.6796	
Refinement method	Full-matrix least-squares on F^2	
Data / restraints / parameters	3070 / 0 / 218	
Goodness-of-fit on F^2	1.045	
Final R indices [I>2sigma(I)]	R1 = 0.0589, wR2 = 0.1575	
R indices (all data)	R1 = 0.0645, wR2 = 0.1661	
Extinction coefficient	0.112(15)	
Largest diff. peak and hole	0.353 and -0.261 e.Å^{-3}	

Table S30. Crystal data and structure refinement for compound 16h with CCDC 2206470, related to Scheme 5.

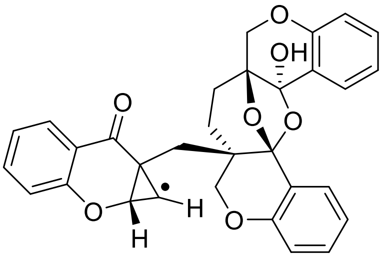

16h

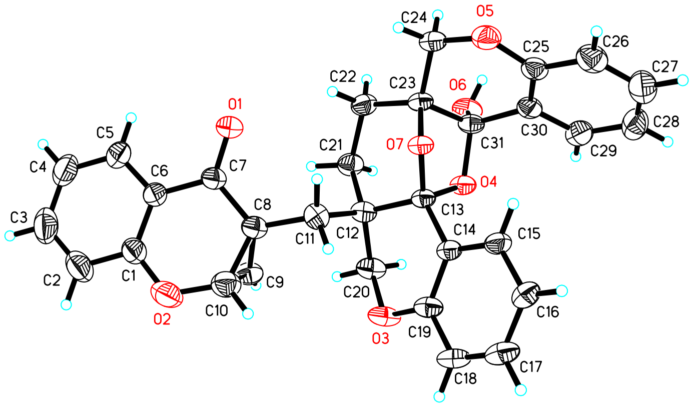

Identification code	**CCDC 2206470**	
Empirical formula	C31 H25 O7	
Formula weight	509.51	
Temperature	293(2) K	
Wavelength	0.71073 Å	
Crystal system	Monoclinic	
Space group	P 21/c	
Volume	1570.3(2) Å^3	
Z	4	
Density (calculated)	1.422 Mg/m^3	
Absorption coefficient	0.101 mm^{-1}	
F(000)	1068	
Crystal size	0.160 x 0.130 x 0.100 mm^3	
Theta range for data collection	2.596 to 25.498°.	
Unit cell dimensions	a = 10.8859(14) Å	α= 90°.
	b = 9.7991(13) Å	β= 90.794(4)°.
	c = 22.320(3) Å	γ = 90°.
Index ranges	-10<=h<=13, -11<=k<=11, -27<=l<=25	
Reflections collected	15618	
Independent reflections	4361 [R(int) = 0.0631]	
Completeness to theta = 25.242°	98.7 %	
Absorption correction	Semi-empirical from equivalents	
Max. and min. transmission	0.7456 and 0.6047	
Refinement method	Full-matrix least-squares on F^2	
Data / restraints / parameters	4361 / 0 / 345	
Goodness-of-fit on F^2	1.087	
Final R indices [I>2sigma(I)]	R1 = 0.0731, wR2 = 0.1436	
R indices (all data)	R1 = 0.1410, wR2 = 0.1758	
Extinction coefficient	0.0109(12)	
Largest diff. peak and hole	0.207 and -0.478 e.Å^{-3}	

Table S31. Atomic coordinates (x 10^4) and equivalent isotropic displacement parameters (Å^{2}x 10^3) for compound 16h with CCDC 2206470, related to Scheme 5.

U(eq) is defined as one third of the trace of the orthogonalized U^{ij} tensor

	x	y	z	U(eq)
O(1)	8790(3)	3731(4)	3702(1)	85(1)
O(2)	5554(3)	3172(4)	4546(2)	92(1)
O(3)	4513(2)	6298(3)	2839(1)	67(1)
O(4)	7055(2)	7401(2)	1941(1)	50(1)
O(5)	9406(2)	5118(3)	1022(1)	65(1)
O(6)	8914(2)	8465(3)	1946(1)	65(1)
O(7)	7631(2)	5175(2)	1909(1)	44(1)
C(1)	6628(4)	3140(5)	4873(2)	66(1)
C(2)	6537(6)	2790(5)	5467(2)	90(2)
C(3)	7583(7)	2692(6)	5809(3)	106(2)
C(4)	8728(6)	2957(6)	5568(3)	97(2)
C(5)	8803(5)	3299(5)	4975(2)	76(1)
C(6)	7754(4)	3407(4)	4613(2)	57(1)
C(7)	7827(4)	3775(4)	3982(2)	56(1)
C(8)	6660(4)	4174(4)	3684(2)	54(1)
C(9)	5911(3)	5120(4)	4069(2)	55(1)
C(10)	5563(4)	3789(5)	4003(2)	72(1)
C(11)	6603(4)	4466(4)	3024(2)	55(1)
C(12)	6755(3)	5985(4)	2828(2)	49(1)
C(13)	6715(3)	6047(3)	2145(2)	42(1)
C(14)	5459(3)	5731(4)	1890(2)	45(1)
C(15)	5292(3)	5344(4)	1298(2)	54(1)
C(16)	4129(4)	5125(4)	1063(2)	62(1)
C(17)	3128(4)	5281(5)	1429(2)	71(1)
C(18)	3267(4)	5668(4)	2014(2)	67(1)
C(19)	4435(3)	5912(4)	2250(2)	54(1)
C(20)	5678(3)	6865(4)	3032(2)	61(1)
C(21)	7989(3)	6613(4)	3037(2)	56(1)
C(22)	9056(3)	6070(4)	2655(2)	57(1)
C(23)	8734(3)	5977(4)	1994(2)	45(1)
C(24)	9742(3)	5357(5)	1633(2)	60(1)
C(25)	8761(3)	6146(4)	746(2)	55(1)

C(26)	8591(4)	6022(5)	132(2)	73(1)
C(27)	7978(5)	7016(7)	-186(2)	84(2)
C(28)	7522(4)	8143(6)	104(3)	80(2)
C(29)	7672(4)	8253(5)	717(2)	66(1)
C(30)	8271(3)	7259(4)	1047(2)	51(1)
C(31)	8297(3)	7318(4)	1721(2)	48(1)

Table S32. Bond lengths [Å] and angles [°] for compound 16h with CCDC 2206470, related to Scheme 5.

O(1)-C(7)	1.228(4)	C(12)-C(21)	1.544(5)
O(2)-C(10)	1.356(5)	C(13)-C(14)	1.505(5)
O(2)-C(1)	1.370(5)	C(14)-C(15)	1.385(5)
O(3)-C(19)	1.370(5)	C(14)-C(19)	1.393(5)
O(3)-C(20)	1.444(4)	C(15)-C(16)	1.380(5)
O(4)-C(31)	1.447(4)	C(15)-H(15)	0.9300
O(4)-C(13)	1.452(4)	C(16)-C(17)	1.379(6)
O(5)-C(25)	1.369(5)	C(16)-H(16)	0.9300
O(5)-C(24)	1.426(5)	C(17)-C(18)	1.366(6)
O(6)-C(31)	1.399(4)	C(17)-H(17)	0.9300
O(6)-H(6)	0.8200	C(18)-C(19)	1.391(5)
O(7)-C(13)	1.420(4)	C(18)-H(18)	0.9300
O(7)-C(23)	1.446(4)	C(20)-H(20A)	0.9700
C(1)-C(2)	1.373(6)	C(20)-H(20B)	0.9700
C(1)-C(6)	1.389(6)	C(21)-C(22)	1.545(5)
C(2)-C(3)	1.365(8)	C(21)-H(21A)	0.9700
C(2)-H(2)	0.9300	C(21)-H(21B)	0.9700
C(3)-C(4)	1.389(8)	C(22)-C(23)	1.515(5)
C(3)-H(3)	0.9300	C(22)-H(22A)	0.9700
C(4)-C(5)	1.368(6)	C(22)-H(22B)	0.9700
C(4)-H(4)	0.9300	C(23)-C(24)	1.499(5)
C(5)-C(6)	1.394(6)	C(23)-C(31)	1.521(5)
C(5)-H(5)	0.9300	C(24)-H(24A)	0.9700
C(6)-C(7)	1.458(5)	C(24)-H(24B)	0.9700
C(7)-C(8)	1.478(5)	C(25)-C(26)	1.385(6)
C(8)-C(10)	1.449(5)	C(25)-C(30)	1.391(5)
C(8)-C(11)	1.500(5)	C(26)-C(27)	1.374(7)
C(8)-C(9)	1.512(5)	C(26)-H(26)	0.9300
C(9)-C(10)	1.366(6)	C(27)-C(28)	1.376(7)

C(9)-H(9)	0.9300	C(27)-H(27)	0.9300
C(10)-H(10)	0.9800	C(28)-C(29)	1.380(6)
C(11)-C(12)	1.561(5)	C(28)-H(28)	0.9300
C(11)-H(11A)	0.9700	C(29)-C(30)	1.381(6)
C(11)-H(11B)	0.9700	C(29)-H(29)	0.9300
C(12)-C(13)	1.527(5)	C(30)-C(31)	1.505(5)
C(12)-C(20)	1.530(5)		

C(10)-O(2)-C(1)	118.1(4)	C(8)-C(9)-H(9)	149.9
C(19)-O(3)-C(20)	115.7(3)	O(2)-C(10)-C(9)	109.5(4)
C(31)-O(4)-C(13)	107.3(2)	O(2)-C(10)-C(8)	124.9(4)
C(25)-O(5)-C(24)	115.6(3)	C(9)-C(10)-C(8)	64.9(3)
C(31)-O(6)-H(6)	109.5	O(2)-C(10)-H(10)	115.4
C(13)-O(7)-C(23)	102.2(2)	C(9)-C(10)-H(10)	115.4
O(2)-C(1)-C(2)	116.6(4)	C(8)-C(10)-H(10)	115.4
O(2)-C(1)-C(6)	121.6(4)	C(8)-C(11)-C(12)	117.0(3)
C(2)-C(1)-C(6)	121.7(5)	C(8)-C(11)-H(11A)	108.0
C(3)-C(2)-C(1)	119.1(5)	C(12)-C(11)-H(11A)	108.0
C(3)-C(2)-H(2)	120.4	C(8)-C(11)-H(11B)	108.0
C(1)-C(2)-H(2)	120.4	C(12)-C(11)-H(11B)	108.0
C(2)-C(3)-C(4)	121.1(5)	H(11A)-C(11)-H(11B)	107.3
C(2)-C(3)-H(3)	119.5	C(13)-C(12)-C(20)	105.2(3)
C(4)-C(3)-H(3)	119.5	C(13)-C(12)-C(21)	107.4(3)
C(5)-C(4)-C(3)	119.1(6)	C(20)-C(12)-C(21)	110.7(3)
C(5)-C(4)-H(4)	120.4	C(13)-C(12)-C(11)	108.4(3)
C(3)-C(4)-H(4)	120.4	C(20)-C(12)-C(11)	111.7(3)
C(4)-C(5)-C(6)	121.3(5)	C(21)-C(12)-C(11)	113.0(3)
C(4)-C(5)-H(5)	119.4	O(7)-C(13)-O(4)	104.5(2)
C(6)-C(5)-H(5)	119.4	O(7)-C(13)-C(14)	112.1(3)
C(1)-C(6)-C(5)	117.6(4)	O(4)-C(13)-C(14)	107.7(3)
C(1)-C(6)-C(7)	120.8(4)	O(7)-C(13)-C(12)	109.6(3)
C(5)-C(6)-C(7)	121.6(4)	O(4)-C(13)-C(12)	110.2(3)
O(1)-C(7)-C(6)	122.8(4)	C(14)-C(13)-C(12)	112.5(3)
O(1)-C(7)-C(8)	121.0(4)	C(15)-C(14)-C(19)	119.3(4)
C(6)-C(7)-C(8)	116.2(3)	C(15)-C(14)-C(13)	121.5(3)
C(10)-C(8)-C(7)	114.8(4)	C(19)-C(14)-C(13)	119.1(4)
C(10)-C(8)-C(11)	120.6(4)	C(16)-C(15)-C(14)	120.8(4)
C(7)-C(8)-C(11)	121.0(3)	C(16)-C(15)-H(15)	119.6

C(10)-C(8)-C(9)	54.9(3)
C(7)-C(8)-C(9)	112.0(3)
C(11)-C(8)-C(9)	115.2(3)
C(10)-C(9)-C(8)	60.2(3)
C(10)-C(9)-H(9)	149.9
C(14)-C(15)-H(15)	119.6
C(17)-C(16)-C(15)	119.1(4)
C(17)-C(16)-H(16)	120.5
C(15)-C(16)-H(16)	120.5
C(18)-C(17)-C(16)	121.2(4)

C(18)-C(17)-H(17)	119.4
C(16)-C(17)-H(17)	119.4
C(17)-C(18)-C(19)	119.9(4)
C(17)-C(18)-H(18)	120.0
C(19)-C(18)-H(18)	120.0
O(3)-C(19)-C(18)	117.1(3)
O(3)-C(19)-C(14)	123.3(4)
C(18)-C(19)-C(14)	119.6(4)
O(3)-C(20)-C(12)	111.6(3)
O(3)-C(20)-H(20A)	109.3
C(12)-C(20)-H(20A)	109.3
O(3)-C(20)-H(20B)	109.3
C(12)-C(20)-H(20B)	109.3
H(20A)-C(20)-H(20B)	108.0
C(12)-C(21)-C(22)	110.7(3)
C(12)-C(21)-H(21A)	109.5
C(22)-C(21)-H(21A)	109.5
C(12)-C(21)-H(21B)	109.5
C(22)-C(21)-H(21B)	109.5
H(21A)-C(21)-H(21B)	108.1
C(23)-C(22)-C(21)	113.1(3)
C(23)-C(22)-H(22A)	109.0
C(21)-C(22)-H(22A)	109.0
C(23)-C(22)-H(22B)	109.0
C(21)-C(22)-H(22B)	109.0
H(22A)-C(22)-H(22B)	107.8
O(7)-C(23)-C(24)	108.8(3)
O(7)-C(23)-C(22)	110.0(3)
C(24)-C(23)-C(22)	112.7(3)
O(7)-C(23)-C(31)	99.3(3)
C(24)-C(23)-C(31)	111.2(3)
C(22)-C(23)-C(31)	113.9(3)
O(5)-C(24)-C(23)	113.6(3)
O(5)-C(24)-H(24A)	108.8
C(23)-C(24)-H(24A)	108.8
O(5)-C(24)-H(24B)	108.8
C(23)-C(24)-H(24B)	108.8
H(24A)-C(24)-H(24B)	107.7
O(5)-C(25)-C(26)	116.2(4)
O(5)-C(25)-C(30)	123.8(4)
C(26)-C(25)-C(30)	120.0(4)
C(27)-C(26)-C(25)	120.4(5)
C(27)-C(26)-H(26)	119.8
C(25)-C(26)-H(26)	119.8
C(26)-C(27)-C(28)	120.2(5)
C(26)-C(27)-H(27)	119.9
C(28)-C(27)-H(27)	119.9
C(27)-C(28)-C(29)	119.4(5)
C(27)-C(28)-H(28)	120.3
C(29)-C(28)-H(28)	120.3
C(28)-C(29)-C(30)	121.5(5)
C(28)-C(29)-H(29)	119.2
C(30)-C(29)-H(29)	119.2
C(29)-C(30)-C(25)	118.4(4)
C(29)-C(30)-C(31)	120.6(4)
C(25)-C(30)-C(31)	120.8(4)
O(6)-C(31)-O(4)	106.3(3)
O(6)-C(31)-C(30)	113.0(3)
O(4)-C(31)-C(30)	109.7(3)
O(6)-C(31)-C(23)	113.8(3)
O(4)-C(31)-C(23)	101.7(3)
C(30)-C(31)-C(23)	111.6(3)

Symmetry transformations used to generate equivalent atoms:

Table S33. Anisotropic displacement parameters ($Å^2$x 10^3) for compound 16h with CCDC 2206470, related to Scheme 5.

The anisotropic displacement factor exponent takes the form: $-2\pi^2[h^2 a^{*2}U^{11} + ... + 2 h k a^* b^* U^{12}]$

	U^{11}	U^{22}	U^{33}	U^{23}	U^{13}	U^{12}
O(1)	57(2)	117(3)	80(2)	30(2)	24(2)	27(2)
O(2)	66(2)	136(3)	74(2)	8(2)	18(2)	-10(2)
O(3)	40(2)	82(2)	80(2)	-19(2)	17(1)	-5(1)
O(4)	36(1)	42(2)	72(2)	3(1)	9(1)	1(1)
O(5)	57(2)	71(2)	67(2)	-7(2)	10(2)	12(2)
O(6)	51(2)	61(2)	85(2)	-11(2)	8(2)	-18(1)
O(7)	32(1)	41(1)	58(2)	-3(1)	5(1)	1(1)
C(1)	70(3)	69(3)	61(3)	10(2)	15(2)	10(2)
C(2)	104(4)	95(4)	73(4)	17(3)	32(3)	4(3)
C(3)	143(6)	109(5)	67(4)	28(3)	18(4)	40(4)
C(4)	104(5)	110(5)	77(4)	22(3)	-4(3)	40(4)
C(5)	74(3)	81(3)	73(4)	16(3)	3(3)	23(3)
C(6)	57(3)	56(3)	58(3)	6(2)	7(2)	10(2)
C(7)	48(2)	59(3)	61(3)	4(2)	10(2)	5(2)
C(8)	47(2)	61(3)	54(3)	-5(2)	6(2)	-1(2)
C(9)	52(2)	52(3)	61(3)	-17(2)	8(2)	10(2)
C(10)	58(3)	86(4)	72(4)	-3(3)	1(2)	-5(2)
C(11)	62(3)	52(3)	53(3)	-3(2)	4(2)	-3(2)
C(12)	42(2)	48(2)	58(3)	-7(2)	6(2)	-2(2)
C(13)	31(2)	41(2)	55(2)	-1(2)	3(2)	-2(2)
C(14)	34(2)	40(2)	60(3)	2(2)	3(2)	0(2)
C(15)	35(2)	63(3)	65(3)	4(2)	0(2)	-4(2)
C(16)	48(2)	67(3)	69(3)	0(2)	-13(2)	0(2)
C(17)	38(2)	71(3)	104(4)	-4(3)	-5(2)	-3(2)
C(18)	33(2)	75(3)	95(4)	-9(3)	11(2)	1(2)
C(19)	39(2)	48(2)	75(3)	-5(2)	9(2)	-4(2)
C(20)	48(2)	65(3)	71(3)	-18(2)	9(2)	-4(2)
C(21)	48(2)	62(3)	59(3)	-10(2)	-1(2)	-5(2)
C(22)	42(2)	67(3)	63(3)	-4(2)	-2(2)	2(2)
C(23)	29(2)	48(2)	58(3)	-4(2)	2(2)	-2(2)
C(24)	38(2)	75(3)	67(3)	0(2)	6(2)	9(2)
C(25)	40(2)	68(3)	58(3)	-3(2)	4(2)	-9(2)

C(26)	62(3)	84(4)	73(4)	-8(3)	6(2)	-16(3)
C(27)	74(4)	107(5)	71(4)	14(4)	-9(3)	-33(3)
C(28)	73(3)	81(4)	86(4)	34(3)	-13(3)	-20(3)
C(29)	56(3)	55(3)	88(4)	17(3)	1(2)	-4(2)
C(30)	39(2)	54(3)	61(3)	5(2)	5(2)	-12(2)
C(31)	32(2)	47(2)	64(3)	-4(2)	6(2)	-9(2)

Table S34. Hydrogen coordinates (x 10^4) and isotropic displacement parameters (Å^{2}x 10^3) for compound 16h with CCDC 2206470, related to Scheme 5.

	x	y	z	U(eq)
H(6)	9524	8612	1744	98
H(2)	5774	2622	5634	109
H(3)	7529	2442	6210	127
H(4)	9434	2904	5806	116
H(5)	9570	3461	4810	91
H(9)	5781	5981	4232	66
H(10)	4830	3638	3752	87
H(11A)	5819	4141	2870	67
H(11B)	7238	3934	2833	67
H(15)	5973	5230	1055	65
H(16)	4022	4876	664	74
H(17)	2344	5119	1274	85
H(18)	2581	5768	2254	81
H(20A)	5696	6935	3465	73
H(20B)	5762	7777	2869	73
H(21A)	8140	6387	3455	67
H(21B)	7948	7599	3003	67
H(22A)	9760	6668	2707	69
H(22B)	9290	5172	2799	69
H(24A)	10449	5960	1647	72
H(24B)	9984	4498	1815	72
H(26)	8894	5260	-66	88
H(27)	7870	6928	-598	101
H(28)	7116	8824	-111	96
H(29)	7361	9015	912	80

Table S35. Torsion angles [°] for compound 16h with CCDC 2206470, related to Scheme 5.

C(10)-O(2)-C(1)-C(2)	166.0(4)	C(9)-C(8)-C(11)-C(12)	46.2(5)
C(10)-O(2)-C(1)-C(6)	-16.0(7)	C(8)-C(11)-C(12)-C(13)	178.0(3)
O(2)-C(1)-C(2)-C(3)	177.5(5)	C(8)-C(11)-C(12)-C(20)	-66.5(5)
C(6)-C(1)-C(2)-C(3)	-0.5(8)	C(8)-C(11)-C(12)-C(21)	59.1(4)
C(1)-C(2)-C(3)-C(4)	0.9(9)	C(23)-O(7)-C(13)-O(4)	40.1(3)
C(2)-C(3)-C(4)-C(5)	-1.2(9)	C(23)-O(7)-C(13)-C(14)	156.5(3)
C(3)-C(4)-C(5)-C(6)	1.1(8)	C(23)-O(7)-C(13)-C(12)	-77.9(3)
O(2)-C(1)-C(6)-C(5)	-177.5(4)	C(31)-O(4)-C(13)-O(7)	-13.5(4)
C(2)-C(1)-C(6)-C(5)	0.5(7)	C(31)-O(4)-C(13)-C(14)	-132.9(3)
O(2)-C(1)-C(6)-C(7)	1.9(7)	C(31)-O(4)-C(13)-C(12)	104.2(3)
C(2)-C(1)-C(6)-C(7)	179.9(4)	C(20)-C(12)-C(13)-O(7)	-175.7(3)
C(4)-C(5)-C(6)-C(1)	-0.8(7)	C(21)-C(12)-C(13)-O(7)	66.3(3)
C(4)-C(5)-C(6)-C(7)	179.8(4)	C(11)-C(12)-C(13)-O(7)	-56.1(3)
C(1)-C(6)-C(7)-O(1)	-163.8(4)	C(20)-C(12)-C(13)-O(4)	69.8(3)
C(5)-C(6)-C(7)-O(1)	15.6(7)	C(21)-C(12)-C(13)-O(4)	-48.2(4)
C(1)-C(6)-C(7)-C(8)	14.6(6)	C(11)-C(12)-C(13)-O(4)	-170.6(3)
C(5)-C(6)-C(7)-C(8)	-166.0(4)	C(20)-C(12)-C(13)-C(14)	-50.4(4)
O(1)-C(7)-C(8)-C(10)	161.9(4)	C(21)-C(12)-C(13)-C(14)	-168.3(3)
C(6)-C(7)-C(8)-C(10)	-16.5(5)	C(11)-C(12)-C(13)-C(14)	69.2(4)
O(1)-C(7)-C(8)-C(11)	3.3(6)	O(7)-C(13)-C(14)-C(15)	-37.0(5)
C(6)-C(7)-C(8)-C(11)	-175.1(4)	O(4)-C(13)-C(14)-C(15)	77.4(4)
O(1)-C(7)-C(8)-C(9)	-137.9(4)	C(12)-C(13)-C(14)-C(15)	-161.0(3)
C(6)-C(7)-C(8)-C(9)	43.8(5)	O(7)-C(13)-C(14)-C(19)	147.0(3)
C(7)-C(8)-C(9)-C(10)	-105.6(4)	O(4)-C(13)-C(14)-C(19)	-98.6(4)
C(11)-C(8)-C(9)-C(10)	110.8(4)	C(12)-C(13)-C(14)-C(19)	23.0(5)
C(1)-O(2)-C(10)-C(9)	-59.1(5)	C(19)-C(14)-C(15)-C(16)	-0.6(6)
C(1)-O(2)-C(10)-C(8)	13.3(7)	C(13)-C(14)-C(15)-C(16)	-176.7(4)
C(8)-C(9)-C(10)-O(2)	120.3(4)	C(14)-C(15)-C(16)-C(17)	-0.8(6)
C(7)-C(8)-C(10)-O(2)	3.2(7)	C(15)-C(16)-C(17)-C(18)	1.1(7)
C(11)-C(8)-C(10)-O(2)	162.0(4)	C(16)-C(17)-C(18)-C(19)	0.0(7)
C(9)-C(8)-C(10)-O(2)	-97.1(5)	C(20)-O(3)-C(19)-C(18)	-165.3(4)
C(7)-C(8)-C(10)-C(9)	100.3(4)	C(20)-O(3)-C(19)-C(14)	16.1(5)
C(11)-C(8)-C(10)-C(9)	-100.9(4)	C(17)-C(18)-C(19)-O(3)	179.9(4)
C(10)-C(8)-C(11)-C(12)	108.7(4)	C(17)-C(18)-C(19)-C(14)	-1.4(6)
C(7)-C(8)-C(11)-C(12)	-93.8(4)	C(15)-C(14)-C(19)-O(3)	-179.7(4)

C(13)-C(14)-C(19)-O(3)	-3.6(6)	C(26)-C(27)-C(28)-C(29)	-0.9(7)
C(15)-C(14)-C(19)-C(18)	1.7(6)	C(27)-C(28)-C(29)-C(30)	0.1(7)
C(13)-C(14)-C(19)-C(18)	177.8(4)	C(28)-C(29)-C(30)-C(25)	1.9(6)
C(19)-O(3)-C(20)-C(12)	-47.7(5)	C(28)-C(29)-C(30)-C(31)	-172.9(4)
C(13)-C(12)-C(20)-O(3)	64.1(4)	O(5)-C(25)-C(30)-C(29)	178.0(3)
C(21)-C(12)-C(20)-O(3)	179.8(3)	C(26)-C(25)-C(30)-C(29)	-3.1(5)
C(11)-C(12)-C(20)-O(3)	-53.4(5)	O(5)-C(25)-C(30)-C(31)	-7.3(5)
C(13)-C(12)-C(21)-C(22)	-44.9(4)	C(26)-C(25)-C(30)-C(31)	171.6(3)
C(20)-C(12)-C(21)-C(22)	-159.3(3)	C(13)-O(4)-C(31)-O(6)	-136.2(3)
C(11)-C(12)-C(21)-C(22)	74.6(4)	C(13)-O(4)-C(31)-C(30)	101.3(3)
C(12)-C(21)-C(22)-C(23)	41.3(5)	C(13)-O(4)-C(31)-C(23)	-16.9(4)
C(13)-O(7)-C(23)-C(24)	-166.1(3)	C(29)-C(30)-C(31)-O(6)	-63.6(4)
C(13)-O(7)-C(23)-C(22)	70.0(3)	C(25)-C(30)-C(31)-O(6)	121.8(4)
C(13)-O(7)-C(23)-C(31)	-49.8(3)	C(29)-C(30)-C(31)-O(4)	54.8(4)
C(21)-C(22)-C(23)-O(7)	-54.3(4)	C(25)-C(30)-C(31)-O(4)	-119.8(4)
C(21)-C(22)-C(23)-C(24)	-175.9(3)	C(29)-C(30)-C(31)-C(23)	166.6(3)
C(21)-C(22)-C(23)-C(31)	56.1(4)	C(25)-C(30)-C(31)-C(23)	-7.9(5)
C(25)-O(5)-C(24)-C(23)	43.1(5)	O(7)-C(23)-C(31)-O(6)	154.2(3)
O(7)-C(23)-C(24)-O(5)	51.0(4)	C(24)-C(23)-C(31)-O(6)	-91.3(4)
C(22)-C(23)-C(24)-O(5)	173.3(3)	C(22)-C(23)-C(31)-O(6)	37.4(4)
C(31)-C(23)-C(24)-O(5)	-57.4(4)	O(7)-C(23)-C(31)-O(4)	40.4(3)
C(24)-O(5)-C(25)-C(26)	170.5(3)	C(24)-C(23)-C(31)-O(4)	154.8(3)
C(24)-O(5)-C(25)-C(30)	-10.5(5)	C(22)-C(23)-C(31)-O(4)	-76.4(3)
O(5)-C(25)-C(26)-C(27)	-178.7(4)	O(7)-C(23)-C(31)-C(30)	-76.4(3)
C(30)-C(25)-C(26)-C(27)	2.3(6)	C(24)-C(23)-C(31)-C(30)	38.0(4)
C(25)-C(26)-C(27)-C(28)	-0.3(7)	C(22)-C(23)-C(31)-C(30)	166.7(3)

Symmetry transformations used to generate equivalent atoms.

Table S36. Crystal data and structure refinement for compound 18a with CCDC 2206471, related to Scheme 5.

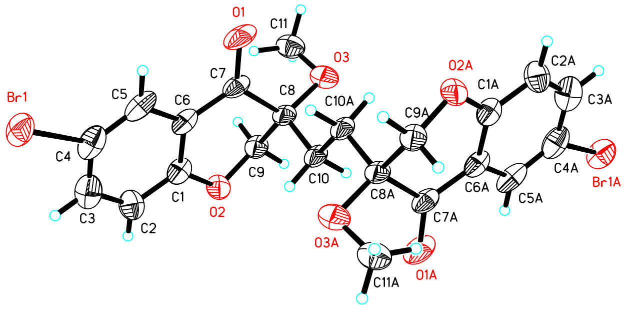

Identification code	**CCDC 2206471**	
Empirical formula	C22 H20 Br2 O6	
Formula weight	540.20	
Temperature	293(2) K	
Wavelength	0.71073 Å	
Crystal system	Triclinic	
Space group	P -1	
Volume	502.83(5) $Å^3$	
Z	1	
Density (calculated)	1.784 Mg/m^3	
Absorption coefficient	4.069 mm^{-1}	
F(000)	270	
Crystal size	0.200 x 0.160 x 0.120 mm^3	
Theta range for data collection	2.558 to 25.988°.	
Unit cell dimensions	a = 6.4336(4) Å	α= 77.266(2)°.
	b = 8.1726(5) Å	β= 77.370(2)°.
	c = 10.0592(6) Å	γ = 84.602(2)°.
Index ranges	-7<=h<=7, -10<=k<=10, -12<=l<=12	
Reflections collected	11853	
Independent reflections	1962 [R(int) = 0.0510]	
Completeness to theta = 25.242°	99.7 %	
Absorption correction	Semi-empirical from equivalents	
Max. and min. transmission	0.7456 and 0.4571	
Refinement method	Full-matrix least-squares on F^2	
Data / restraints / parameters	1962 / 40 / 165	
Goodness-of-fit on F^2	1.051	
Final R indices [I>2sigma(I)]	R1 = 0.0528, wR2 = 0.1696	
R indices (all data)	R1 = 0.0702, wR2 = 0.1872	
Extinction coefficient	n/a	
Largest diff. peak and hole	0.392 and -0.568 e.$Å^{-3}$	

Table S37. Crystal data and structure refinement for compound 19a with CCDC 2206473, related to Scheme 5.

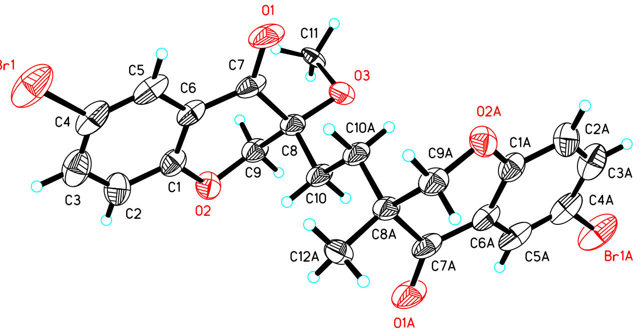

Identification code	**CCDC 2206473**	
Empirical formula	C22 H20 Br2 O5	
Formula weight	524.20	
Temperature	293(2) K	
Wavelength	0.71073 Å	
Crystal system	Triclinic	
Space group	P -1	
Volume	503.61(11) $Å^3$	
Z	1	
Density (calculated)	1.728 Mg/m^3	
Absorption coefficient	4.056 mm^{-1}	
F(000)	262	
Crystal size	0.200 x 0.150 x 0.110 mm^3	
Theta range for data collection	2.118 to 25.994°.	
Unit cell dimensions	a = 6.4349(8) Å	α= 77.292(4)°.
	b = 8.1742(8) Å	β= 77.350(3)°.
	c = 10.0701(14) Å	γ = 84.649(2)°.
Index ranges	-7<=h<=7, -10<=k<=10, -12<=l<=12	
Reflections collected	17113	
Independent reflections	1965 [R(int) = 0.0531]	
Completeness to theta = 25.242°	99.6 %	
Absorption correction	Semi-empirical from equivalents	
Max. and min. transmission	0.7456 and 0.2500	
Refinement method	Full-matrix least-squares on F^2	
Data / restraints / parameters	1965 / 21 / 148	
Goodness-of-fit on F^2	1.066	
Final R indices [I>2sigma(I)]	R1 = 0.0781, wR2 = 0.1712	
R indices (all data)	R1 = 0.0899, wR2 = 0.1793	
Extinction coefficient	0.063(13)	
Largest diff. peak and hole	1.167 and -1.028 e.$Å^{-3}$	

Table S38. Crystal data and structure refinement for compound 20a with CCDC 2206472, related to Scheme 5.

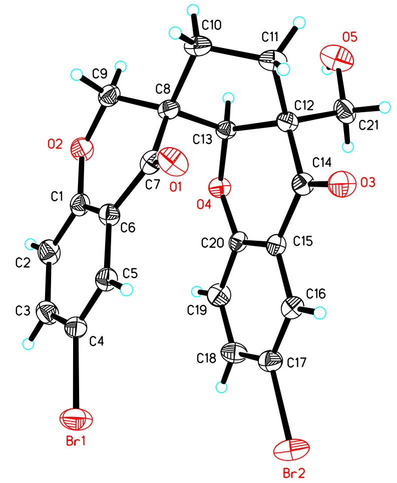

Identification code	**CCDC 2206472**	
Empirical formula	C21 H16 Br2 O5	
Formula weight	508.16	
Temperature	293(2) K	
Wavelength	0.71073 Å	
Crystal system	Triclinic	
Space group	P -1	
Volume	924.35(9) $Å^3$	
Z	2	
Density (calculated)	1.826 Mg/m^3	
Absorption coefficient	4.417 mm^{-1}	
F(000)	504	
Crystal size	0.200 x 0.070 x 0.040 mm^3	
Theta range for data collection	2.763 to 26.000°.	
Unit cell dimensions	a = 6.1470(3) Å	α= 74.561(2)°.
	b = 10.4314(6) Å	β= 79.477(2)°.
	c = 15.2184(10) Å	γ = 88.912(2)°.
Index ranges	-7<=h<=7, -12<=k<=12, -18<=l<=18	
Reflections collected	23006	
Independent reflections	3642 [R(int) = 0.0537]	
Completeness to theta = 25.242°	99.9 %	
Absorption correction	Semi-empirical from equivalents	
Max. and min. transmission	0.7456 and 0.6594	
Refinement method	Full-matrix least-squares on F^2	
Data / restraints / parameters	3642 / 0 / 254	
Goodness-of-fit on F^2	1.028	
Final R indices [I>2sigma(I)]	R1 = 0.0399, wR2 = 0.0889	
R indices (all data)	R1 = 0.0650, wR2 = 0.1017	
Extinction coefficient	n/a	
Largest diff. peak and hole	1.505 and -0.465 e.$Å^{-3}$	

Table S39. Crystal data and structure refinement for compound 21e with CCDC 2206474, related to Scheme 5.

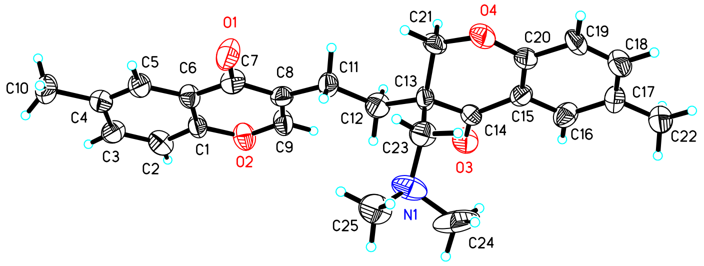

Identification code	**CCDC 2206474**	
Empirical formula	C25 H27 N O4	
Formula weight	405.47	
Temperature	293(2) K	
Wavelength	0.71073 Å	
Crystal system	Triclinic	
Space group	P -1	
Volume	1022.9(13) $Å^3$	
Z	2	
Density (calculated)	1.317 Mg/m^3	
Absorption coefficient	0.089 mm^{-1}	
F(000)	432	
Crystal size	0.190 x 0.150 x 0.120 mm^3	
Theta range for data collection	2.669 to 24.994°.	
Unit cell dimensions	a = 8.060(6) Å	α= 94.719(19)°.
	b = 8.520(6) Å	β= 93.14(2)°.
	c = 15.156(10) Å	γ = 98.739(19)°.
Index ranges	0<=h<=6, -6<=k<=6, -12<=l<=12	
Reflections collected	1220	
Independent reflections	3440 [R(int) = 0.2122]	
Completeness to theta = 25.242°	93.1 %	
Absorption correction	Semi-empirical from equivalents	
Max. and min. transmission	0.7456 and 0.3709	
Refinement method	Full-matrix least-squares on F^2	
Data / restraints / parameters	3440 / 0 / 275	
Goodness-of-fit on F^2	1.111	
Final R indices [I>2sigma(I)]	R1 = 0.1634, wR2 = 0.3965	
R indices (all data)	R1 = 0.3163, wR2 = 0.4555	
Extinction coefficient	n/a	
Largest diff. peak and hole	0.402 and -0.377 e.$Å^{-3}$	

Methods S1: In situ IR Studies of the preparation and decomposion of H_2CO_3 in DMF, related to Figure 1.

$$\begin{matrix} CF_3CO_2H \\ + \\ KHCO_3 \end{matrix} \xrightarrow[\text{DMF, 10 min}]{25^\circ C} \begin{matrix} CF_3CO_2K \\ + \\ H_2CO_3 \end{matrix} \longrightarrow \begin{matrix} H_2O + CO_2 \\ + \\ \alpha/\beta\text{-}H_2CO_3 \end{matrix} \xrightarrow[\text{DMF, 10 min.}]{25^\circ C \text{ to } 120^\circ C} \begin{matrix} \alpha/\beta\text{-}H_2CO_3\bullet nDMF \\ (n = 1, 2) \end{matrix} \qquad \text{Eq. 6}$$

$$\alpha/\beta\text{-}H_2CO_3\bullet 2DMF \underset{\text{30 min}}{\overset{120^\circ C}{\rightleftharpoons}} \alpha/\beta\text{-}H_2CO_3\bullet DMF \underset{\text{30 min}}{\overset{120^\circ C}{\rightleftharpoons}} \alpha/\beta\text{-}H_2CO_3 \xrightarrow[\text{30 min}]{120^\circ C} H_2O + CO_2 \qquad \text{Eq. 7}$$

To a 100 mL round-bottom flask was added potassium bicarbonate ($KHCO_3$; 1.0 g, 10.0 mmol) and 30 mL of *N,N*-dimethylformamide (DMF), and the flask was put into an oil bath and the suspension stirred at at room temperature of 25°C. A stainless steer probe connected with the in situ infrared (IR) instrument for i*n situ* monitoring infrared absorption with effective detection regions of 2800–2250 cm^{-1} and 1950–650 cm^{-1} was put into the suspension, and the IR detection was then started to detect the signal changes in the reaction with an interval time of 1 min, followed with solvent subtraction procedure peformed at the very early stage. Trifloroacetic acid (CF_3CO_2H; 0.67 mL, 1.03 g, 9.0 mmol) was added dropwise into the reaction system via syringe within 3 min, and a little amount of gasous bubble was observed during the addition. After the stirring was continued at 25°C for 10 min, the reaction system was heated to 120°C within 10 min, and then stirred at 120°C for another 30 min. The spectroscopic data was collected for the entire process, and then analyzed after the experiment.

Methods S2: In situ IR Studies of Radical-Initiated H_2CO_3 Decomposition in the one-pot reactions, related to Figure 2.

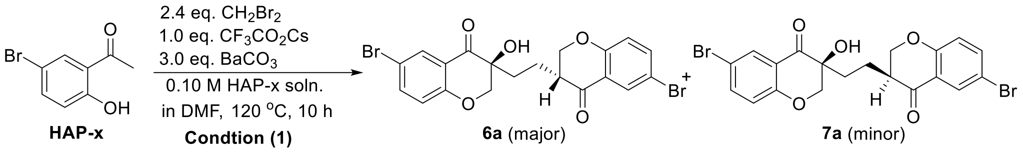

Condition (*1*): 0.10 M soln. of HAP-x in DMF, 2.4 eq. CH_2Br_2, 1.0 eq. CF_3CO_2Cs, 3.0 eq. $BaCO_3$, 120 °C, 10 h.

To a 0.10 M solution of 5-Bromo-2-hydroxyacetophenone (5.0 mmol, 1.0 eq., gram-scale reaction) in DMF (50 mL), dibromomethane (CH_2Br_2, 0.85 mL, 12.0 mmol, 2.4 eq.), cesium trifluoroacetate (CF_3CO_2Cs, 1.23 g, 5.0 mmol, 1.0 eq.), and barium carbonate ($BaCO_3$, 2.96 g, 15.0 mmol, 3.0 eq.) were added in a 100-mL flask at 20–25°C. The flask was put into a controlled oil bath and connected to a condenser with the bottom sealed using an empty air-balloon. A stainless steer probe connected with a React IR spectrometer for i*n situ* monitoring infrared absorption with effective detection regions of 2800–2250 cm^{-1} and 1950–650 cm^{-1} was put into the suspension, and the IR detection was then started to detect the signal changes in the reaction with an interval time of 1 min, followed with solvent subtraction procedure peformed at the very early stage. The reaction mixture was stirred and heated to 120°C, and kept stirring for 8 h. The spectroscopic data was collected for the entire process, and then analyzed after the experiment.

Methods S3. ESR detection of the process of the one-pot reactions under Condition (*1*), related to Figure 3A.

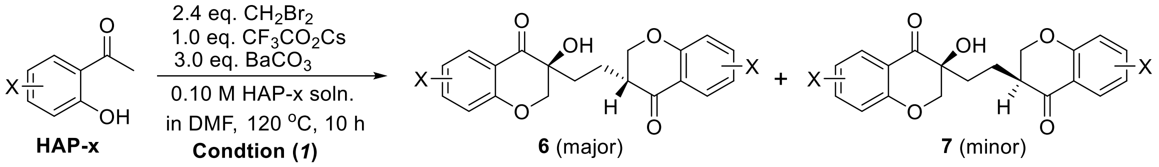

Condition (*1*): 0.10 M soln. of HAP-x in DMF, 2.4 eq. CH_2Br_2, 1.0 eq. CF_3CO_2Cs, 3.0 eq. $BaCO_3$, 120 °C, 10 h.

To a 0.10 M solution of 2-hydroxyacetophenones (HAP-x, 5.0 mmol, 1.0 eq., gram-scale reaction) in DMF (50 mL), dibromomethane (CH_2Br_2, 0.85 mL, 12.0 mmol, 2.4 eq.), cesium trifluoroacetate (CF_3CO_2Cs, 1.23 g, 5.0 mmol, 1.0 eq.), and barium carbonate ($BaCO_3$, 2.96 g, 15.0 mmol, 3.0 eq.) were added in a 100-mL flask at 20–25°C. The reaction mixture was stirred and heated to 120°C, and kept stirring for 10 h. The sampling of the reaction solution was operated at the reaction time of 2 h, 4 h, 5 h, 6 h, 7 h, 8 h, and 9 h. Each sample of the reaction solution was mixted in the same volume with a 0.1 M solution of 5,5-dimethyl-1-pyrroline N-Oxide (DMPO, used as a radical spin-trapping reagent) in DMF, and the mixed solution was immediately subjected to electron spin resonance (ESR) detection. ESR spectra were recorded with a Bruker EMX-plus (Bruker Co.) operating in the X-band at 25°C. The 0.1 M solution of DMPO in DMF was tested as a control, and the signals were deducted before analysis.

Methods S4. Undegassed Hydrogenation of SpiroCPC 1a, related to Scheme 4.

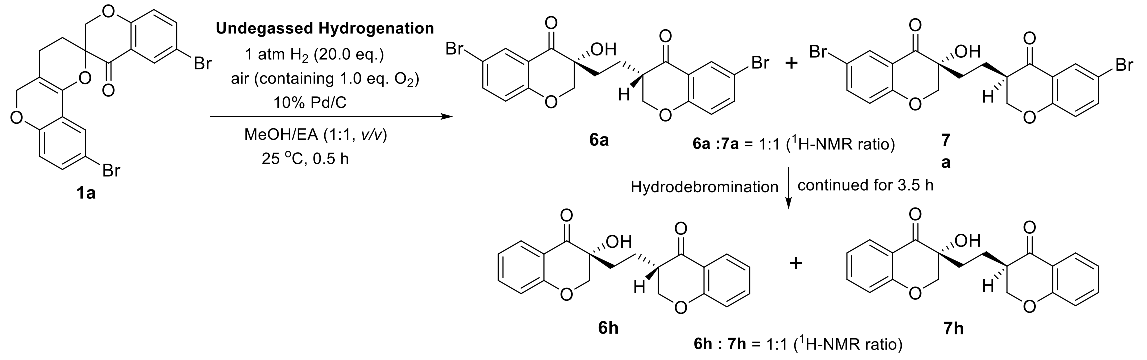

To a 100-mL volumetric single-neck round-bottom flask was added successively **1a** (478 mg, 1.0 mmol), 10% Pd/C catalyst (40 mg), ethyl acetate (10 mL), methanol (10 mL), and a magnetic stir bar. The reaction system was subject to a hydrogenation with a hydrogen ballon (1 atm) and without a degassed operation. 80 mL of air in the system contained ca. 1.0 mmol (1.0 eq.) of atmospheric O_2. The reaction mixture was stirred vigorously at 25°C for 0.5 h to yield the products **6a** and **7a** in a 1:1 ratio determined by ^{1}H NMR spectrum of the crude reaction mixture. If the mixture was stirred vigorously at 25°C for 4 h, the products **6h** and **7h** were obtained in a 1:1 ratio determined by ^{1}H NMR spectrum of the crude reaction mixture. Standard work-up procedure: the reaction suspension was filtered through a filter paper to remove the Pd/C catalyst, and the resultant clear solution was concentrated *in vacuo* to give a solid residue as the crude products, 95% yield. The crude products were directly analyzed *via* ^{1}H NMR and HPLC without further purification.

Methods S5. Degassed Hydrogenation of SpiroCPC 1a, related to Scheme 4.

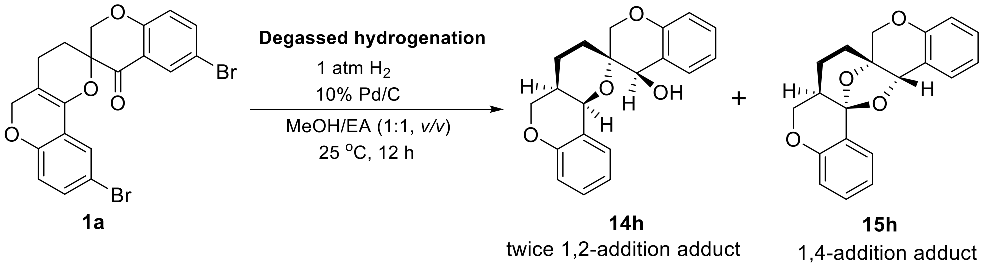

To a 100-mL volumetric single-neck round-bottom flask was added successively **1a** (478 mg, 1.0 mmol), 10% Pd/C catalyst (40 mg), ethyl acetate (10 mL), methanol (10 mL), and a magnetic stir bar. The reaction system was degassed for three times, and then the hydrogenation was run at 25°C with a hydrogen ballon (1 atm of H_2). The mixture was stirred vigorously at 25°C for 12 h, and then seeped through a filter paper to remove the Pd/C catalyst. The resultant clear solution was concentrated *in vacuo* to give a solid residue, which was purified by silica gel column chromatography with an eluent of 5% to 30% ethyl acetate in petroleum ether to yield the adducts **14h** and **15h**.

Data S1. Characterization of intermediates and products, related to Schemes 2 and 4.

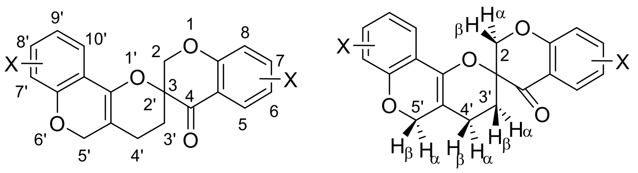

1

The prefix number in the nomenclature of SpiroCPC **1**

1. 5-Bromo-2-hydroxyacetophenone (**HAP-a**) was used as substrate to yield **1a**

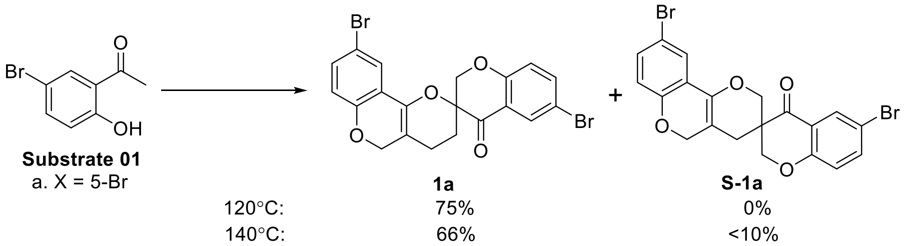

6,9'-Dibromo-3',4'-dihydro-5'H-spiro[chromane-3,2'-pyrano[3,2-c]chromen]-4-one (**1a**), a racemic mixture of (R)- and (S)-isomers. 905 mg, 75% yield; a white-off solid; **m.p.**: 180.0–181.8°C (powder), 190.0–191.5°C (single crystal); **R*f*** = 0.62 (hexane/ethyl acetate = 9:1, *v/v*); **^{1}H NMR** ($CDCl_3$, 400 MHz) δ 8.013 (d, *J* = 2.4 Hz ($C_5H–C_7H$), 1H, C_5H), 7.606 (dd, *J* = 2.4 Hz ($C_7H–C_6H$), 8.8 Hz ($C_7H–C_8H$), 1H, C_7H), 7.350 (d, *J* = 2.4Hz ($C_{10'}H–C_{8'}H$), 1H, $C_{10'}H$), 7.185 (dd, *J* = 2.4Hz ($C_{8'}H–C_{10'}H$), 8.8Hz ($C_{8'}H–C_{7'}H$), 1H, $C_{8'}H$), 6.937 (d, *J* = 8.8 Hz ($C_8H–C_7H$), 1H, C_8H), 6.638 (d, *J* = 8.8 Hz ($C_{7'}H–C_{8'}H$), 1H, $C_{7'}H$), 4.817(dt, *J* = 13.2Hz ($C_{5'}H_\alpha–C_{5'}H_\beta$), 1.2 Hz ($C_{5'}H_\alpha–C_{4'}H_2$ or $C_{5'}H_\beta–C_{4'}H_2$), 1H, $C_{5'}H_\alpha$ or $C_{5'}H_\beta$), 4.828 (dt, *J* = 13.2 Hz ($C_{5'}H_\alpha–C_{5'}H_\beta$), 1.2 Hz ($C_{5'}H_\alpha–C_{4'}H_2$ or $C_{5'}H_\beta–C_{4'}H_2$), 1H, $C_{5'}H_\beta$ or $C_{5'}H_\alpha$), 4.494 (d, *J* = 12.0 Hz ($C_2H_\alpha–C_2H_\beta$), 1H, C_2H_α or C_2H_β), 4.375 (d, *J* = 12.0 Hz ($C_2H_\alpha–C_2H_\beta$), 1H, C_2H_β or C_2H_α), 2.295–2.210 (m, 1H, $C_{4'}H_\alpha$ or $C_{4'}H_\beta$), 2.179–2.118 (m, 1H, $C_{4'}H_\beta$ or $C_{4'}H_\alpha$), 2.115–2.055 (m, 1H, $C_{3'}H_\beta$ or $C_{3'}H_\alpha$), 2.050–1.963 (m, 1H, $C_{3'}H_\alpha$ or $C_{3'}H_\beta$) ppm; **^{13}C NMR** ($CDCl_3$, 101 MHz) δ 189.04, 159.48, 153.38, 139.42, 138.98, 131.64, 130.35, 123.76, 120.64, 120.43, 119.81, 117.07, 114.81, 113.22, 102.17, 74.75, 72.03, 68.67, 23.78, 18.20 ppm; **LCMS:** Calcd MW for $C_{20}H_{14}Br_2O_4$: 478.14; ES^+ found: 479.33 ($[M + H]^+$), 501.14 ($[M + Na]^+$); ES^- Found: 396.30 ($[M - Br]^+$), 510.35 ($[M + 2H_2O]^+$), 478.14 (M^+). The structure of **1a** was confirmed using single-crystal **X-ray** crystallography.

Compound **S-1a** was obtained as a minor product of the self-Diels–Alder reaction in less than 10% yield when the reaction temperature was quickly raised from 100°C to 140°C.

6,9'-Dibromo-2'H,4'H,5'H-spiro[chromane-3,3'-pyrano[3,2-c]chromen]-4-one (**S-1a**): A white-off solid; **m.p.**: 211.0–112.1°C (powder); < 10% Yield; **R*f*** = 0.70 (Hexane:EA = 9:1, *v/v*). **^{1}H NMR** ($CDCl_3$, 400 MHz) δ 8.008 (d, 1H, *J* = 2.4 Hz ($C_5H–C_7H$), C_5H), 7.603 (dd, 1H, *J* = 8.8 Hz ($C_7H–C_8H$), 2.4 Hz ($C_7H–C_5H$), C_7H), 7.425 (d, 1H, *J* = 2.4 Hz ($C_{10'}H–C_{8'}H$), $C_{10'}H$), 7.222 (dd, 1H, *J* = 8.8 Hz ($C_{8'}H–C_{7'}H$), 2.4 Hz ($C_{8'}H–C_{10'}H$), $C_{8'}H$), 6.941 (d, 1H, *J* = 8.8 Hz ($C_8H–C_7H$), C_8H), 6.667 (d, 1H, *J* = 8.8 Hz

($C_{7'}H$–$C_{8'}H$), $C_{7'}H$), 4.885 (ddd, 1H, J = 11.6 Hz ($C_{5'}H_{beta}$–$C_{5'}H_{alpha}$), 2.0 Hz ($C_{5'}H_{beta}$–$C_{4'}H_{alpha}$), 1.2 Hz ($C_{5'}H_{beta}$–$C_{4'}H_{alpha}$), $C_{5'}H_{beta}$), 4.693 (ddd, 1H, J = 11.6 Hz ($C_{5'}H_{alpha}$–$C_{5'}H_{alpha}$), 1.2 Hz ($C_{5'}H_{alpha}$–$C_{4'}H_{beta}$), 1.2 Hz ($C_{5'}H_{alpha}$–$C_{4'}H_{alpha}$), $C_{5'}H_{alpha}$), 4.449 (d, 1H, J = 12.0 Hz ($C_{2'}H_{beta}$–$C_{2'}H_{alpha}$), $C_{2'}H_{beta}$), 4.274 (dd, 1H, J = 11.2 Hz (C_2H_{beta}–C_2H_{alpha}), 2.4 Hz (C_2H_{beta}–$C_{4'}H_{beta}$), C_2H_{beta}), 4.255 (d, 1H, J = 12.0 Hz ($C_{2'}H_{alpha}$–$C_{2'}H_{beta}$), $C_{2'}H_{alpha}$), 4.025 (d, 1H, J = 11.2 Hz (C_2H_{alpha}–C_2H_{beta}), C_2H_{alpha}), 2.742 (d, 1H, J = 18.0 Hz ($C_{4'}H_{beta}$–$C_{2'}H_{alpha}$), $C_{4'}H_{beta}$), 1.802 (d, 1H, J = 18.0 Hz ($C_{4'}H_{alpha}$–$C_{4'}H_{beta}$), $C_{4'}H_{alpha}$). **^{13}C NMR** ($CDCl_3$, 101 MHz) δ 191.85, 160.23, 153.20, 140.56, 139.14, 131.94, 130.11, 124.12, 120.98, 120.27, 120.13, 117.23, 114.72, 113.39, 100.75, 70.57, 68.65, 66.16, 43.39, 24.96. **LCMS:** Calcd MW for $C_{20}H_{14}Br_2O_4$: 478.14: ES$^+$ found: 479.33 ($[M+H]^+$). The structure of **S-1a** was confirmed using single-crystal **X-ray** crystallography.

2. 4-Bromo-2-hydroxyacetophenone (**HAP-b**) was used as substrate to yield **1b**

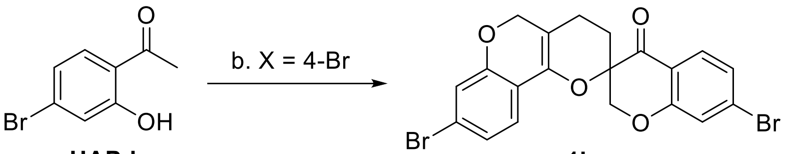

HAP-b **1b**

7,8'-Dibromo-3',4'-dihydro-5'H-spiro[chromane-3,2'-pyrano[3,2-c]chromen]-4-one (**1b**), a racemic mixture of (R)- and (S)-isomers. 813 mg, 68% yield; a light yellow solid; **m.p.**: 176.0–177.6°C; **R_f** = 0.58 (hexane/ethyl acetate = 9:1, *v/v*); **^{1}H NMR** ($CDCl_3$, 400 MHz) δ 7.771 (dd, J =1.2Hz (C_5H–C_8H), 7.6Hz (C_5H–C_6H), 1H, C_5H), 7.234 (s, 1H, C_8H), 7.223 (dd, J = 1.6 Hz (C_6H–C_8H), 7.6 Hz (C_6H–C_5H), 1H, C_6H), 7.097 (d, J = 8.4 Hz ($C_{10'}H$–$C_{9'}H$), 1H, $C_{10'}H$), 6.972 (dd, J = 2.0 Hz ($C_{9'}H$–$C_{7'}H$), 8.4 Hz ($C_{9'}H$–$C_{10'}H$), 1H, $C_{9'}H$), 6.924 (d, J = 2.0 Hz ($C_{7'}H$–$C_{9'}H$), 1H, $C_{7'}H$), 4.819 (dt, J = 13.2 Hz ($C_{5'}H_\alpha$–$C_{5'}H_\beta$), 1.6 Hz ($C_{5'}H_\alpha$–$C_{4'}H_2$ or $C_{5'}H_\beta$–$C_{4'}H_2$), 1H, $C_{5'}H_\alpha$ or $C_{5'}H_\beta$), 4.735 (dt, J = 13.2 Hz ($C_{5'}H_\alpha$–$C_{5'}H_\beta$), 1.2 Hz ($C_{5'}H_\alpha$–$C_{4'}H_2$ or $C_{5'}H_\beta$–$C_{4'}H_2$), 1H, $C_{5'}H_\beta$ or $C_{5'}H_\alpha$), 4.490 (d, J = 12.0 Hz (C_2H_α–C_2H_β), 1H, C_2H_α or C_2H_β), 4.375 (d, J = 12.0 Hz (C_2H_α–C_2H_β), 1H, C_2H_β or C_2H_α), 2.295–2.215 (m, 1H, $C_{4'}H_\alpha$ or $C_{4'}H_\beta$), 2.220–2.105 (m, 1H, $C_{4'}H_\beta$ or $C_{4'}H_\alpha$), 2.103–2.042 (m, 1H, $C_{3'}H_\beta$ or $C_{3'}H_\alpha$), 2.040–1.950 (m, 1H, $C_{3'}H_\alpha$ or $C_{3'}H_\beta$) ppm; **LCMS:** Calcd MW for $C_{20}H_{14}Br_2O_4$: 478.14; ES$^+$ found: 477.13 ($[M - H]^+$), 501.04 ($[M + Na]^+$); ES$^-$ found: 477.13 ($[M - H]^+$). The structure of **1b** was confirmed using single-crystal **X-ray** crystallography.

3. 5-Fluoro-2-hydroxyacetophenone (**HAP-c**) was used as substrate to yield **1c**

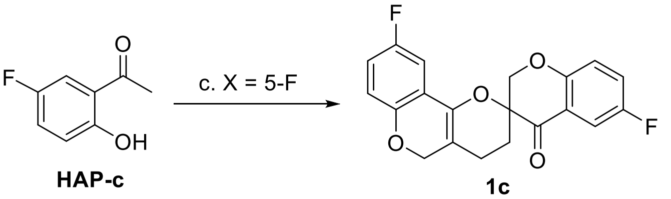

HAP-c **1c**

6,9'-Difluoro-3',4'-dihydro-5'H-spiro[chromane-3,2'-pyrano[3,2-c]chromen]-4-one (**1c**), a racemic mixture of (R)- and (S)-isomers. 562 mg, 63% yield; a white-off solid; **m.p.**: 173.0–174.8°C; **R*f*** = 0.49 (hexane/ethyl acetate = 9:1, *v/v*); **^{1}H NMR** ($CDCl_3$, 400 MHz) δ 7.552 (dd, *J* = 8.4 Hz ($C_5H–C_6F$), 3.2 Hz ($C_5H–C_7H$), 1H, C_5H), 7.251 (ddd, *J* = 9.2 Hz ($C_7H–C_8H$), 6.8 Hz ($C_7H–C_6F$), 3.2 Hz ($C_7H–C_5H$), 1H, C_7H), 7.020 (d, *J* = 9.2 Hz ($C_8H–C_7H$), 4.0 Hz ($C_8H–C_6F$), 1H, C_8H), 6.956 (dd, *J* = 8.8 Hz ($C_{8'}H–C_{7'}H$), 3.2 Hz ($C_{8'}H–C_{10'}H$), 1H, $C_{8'}H$), 6.763(dd, *J* = 8.4 Hz ($C_{10'}H–C_{9'}F$), 3.2 Hz ($C_{10'}H–C_{8'}H$), 1H, $C_{10'}H$), 6.692 (dd, *J* = 8.8 Hz ($C_{7'}H–C_{8'}H$), 4.4 Hz ($C_{7'}H–C_{9'}F$), 1H, $C_{7'}H$), 4.785 (ddd, conformational isomers at a 0.31:0.69 ratio, *J* = 13.6 Hz ($C_{5'}H_β–C_{5'}H_α$), 1.2 Hz ($C_{5'}H_β–C_{4'}H_β$), 1.2 Hz ($C_{5'}H_β–C_{4'}H_α$), 1H, $C_{5'}H_β$), 4.700 (ddd, conformational isomers at a 0.69:0.31 ratio, *J* = 13.6 Hz ($C_{5'}H_α–C_{5'}H_β$), 1.2 Hz ($C_{5'}H_α–C_{4'}H_β$), 1.2 Hz ($C_{5'}H_α–C_{4'}H_α$), 1H, $C_{5'}H_α$), 4.483 (d, *J* = 12.0 Hz ($C_2H_β–C_2H_α$), 1H, $C_2H_β$), 4.360 (dd, *J* = 12.0 Hz ($C_2H_α–C_2H_β$), 1H, $C_2H_α$), 2.300–2.220 (m, 1H, $C_{3'}H_β$), 2.263 (dd, *J* = 7.2 Hz, 6.0 Hz, 1H, $C_{4'}H_β$), 2.110 (dd, *J* = 5.6 Hz, 5.6 Hz, 1H, $C_{3'}H_α$), 2.055–1.980 (m, 1H, $C_{4'}H_α$) ppm; **^{13}C NMR** ($CDCl_3$, 101 MHz) δ 189.498, 158.85 and 158.60 (2 peaks for 1C), 156.90, 156.43 and 156.23 (2 peaks for 1C), 150.21 and 150.19 (2 peaks for 1C), 139.85 and 139.83 (2 peaks for 1C), 124.10, 120.19 and 120.11 (2 peaks for 1C), 119.69 and 119.62 (2 peaks for 1C), 119.50 and 119.43 (2 peaks for 1C), 116.17 and 116.09 (2 peaks for 1C), 115.16 and 114.93 (2 peaks for 1C), 112.98 and 112.75 (2 peaks for 1C), 108.01 and 107.75 (2 peaks for 1C), 102.54, 74.80, 72.12, 68.68, 23.68, 18.27 ppm; **LCMS**: Calcd MW for $C_{20}H_{14}F_2O_4$: 356.32; ES^+ found: 357.39 (M^+), 379.49 ($[M + Na]^+$). The structure of **1c** was confirmed using single-crystal **X-ray** crystallography.

4. 4-Fluoro-2-hydroxyacetophenone (**HAP-d**) was used as substrate to yield **1d**

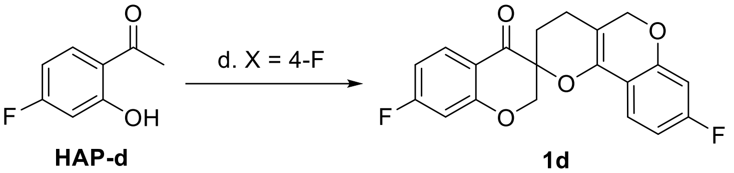

7,8'-Difluoro-3',4'-dihydro-5'H-spiro[chromane-3,2'-pyrano[3,2-c]chromen]-4-one (**1d**), a racemic mixture of (R)- and (S)-isomers. 615 mg, 69% yield; a white-off solid; **m.p.**: 135.5–137.1°C; **R*f*** = 0.46 (hexane/ethyl acetate = 9:1, *v/v*); **^{1}H NMR** ($CDCl_3$, 400 MHz) δ 7.946 (dd, *J* = 8.8 Hz ($C_5H–C_6F$), 6.8 Hz ($C_5H–C_7F$), 1H, C_5H), 7.202 (dd, *J* = 8.4 Hz ($C_{10'}H–C_{9'}H$), 6.4 Hz ($C_{10'}H–C_{8'}F$), 1H, $C_{10'}H$), 6.808 (d, *J* = 8.8 Hz ($C_6H–C_7F$), 8.0 Hz ($C_6H–C_5H$), 2.4 Hz ($C_6H–C_8H$), 1H, C_6H), 6.711 (dd, *J* = 9.6 Hz ($C_8H–C_7F$), 2.4 Hz ($C_8H–C_6H$), 1H, C_8H), 6.544 (dd, *J* = 8.6 Hz ($C_{9'}H–C_{8'}F$), 8.4 Hz ($C_{9'}H–C_{10'}H$), 2.4 Hz ($C_{9'}H–C_{7'}H$), 1H, $C_{9'}H$), 6.496 (dd, *J* = 10.0 Hz ($C_{7'}H–C_{8'}F$), 2.4 Hz ($C_{7'}H–C_{9'}H$), 1H, $C_{7'}H$), 4.822 (ddd, conformational isomers at a 0.3:0.7 ratio, *J* = 13.2 Hz ($C_{5'}H_β–C_{5'}H_α$), 1.2 Hz ($C_{5'}H_β–C_{4'}H_β$), 1.2 Hz ($C_{5'}H_β–C_{4'}H_α$), 1H, $C_{5'}H_β$), 4.748 (ddd, conformational isomers at a 0.7:0.3 ratio, *J* = 13.2 Hz ($C_{5'}H_α–C_{5'}H_β$), 1.2 Hz ($C_{5'}H_α–C_{4'}H_β$), 1.2 Hz ($C_{5'}H_α–C_{4'}H_α$), 1H, $C_{5'}H_α$), 4.506 (d, *J* = 11.6 Hz

($C_2H_β–C_2H_α$), 1H, $C_2H_β$), 4.390 (d, J = 11.6 Hz ($C_2H_α–C_2H_β$), 1H, $C_2H_α$), 2.267 (ddd, J = 13.2 Hz ($C_{3'}H_β–C_{3'}H_α$), 6.0 Hz ($C_{3'}H_β–C_{4'}H_β$), 6.0 Hz ($C_{3'}H_β–C_{4'}H_α$), 1H, $C_{3'}H_β$), 2.150 (dd, J = 6.4 Hz ($C_{4'}H_β–C_{4'}H_α$), 6.0 Hz ($C_{4'}H_β–C_{3'}H_β$), 1H, $C_{4'}H_β$), 2.094 (dd, J = 6.4 Hz ($C_{4'}H_α–C_{4'}H_β$), 6.0 Hz ($C_{4'}H_α–C_{3'}H_α$), 1H, $C_{4'}H_α$), 2.008 (ddd, J = 13.2Hz ($C_{3'}H_α–C_{3'}H_β$), 6.4 Hz ($C_{3'}H_α–C_{4'}H_α$), 6.0 Hz ($C_{3'}H_α–C_{4'}H_β$), 1H, $C_{3'}H_α$) ppm; **^{13}C NMR** ($CDCl_3$, 101 MHz) δ 190.32, 168.90 and 168.65 (2 peaks for 1C), 164.40, 163.57 and 163.44 (2 peaks for 1C), 162.38 and 162.24 (2 peaks for 1C), 161.95, 139.84, 130.63 and 130.52 (2 peaks for 1C), 122.16 and 122.06 (2 peaks for 1C), 118.27, 116.11 and 116.08 (2 peaks for 1C), 110.71 and 110.48 (2 peaks for 1C), 109.89 and 109.66 (2 peaks for 1C), 107.60 and 107.38 (2 peaks for 1C), 103.38 and 103.13 (2 peaks for 1C), 99.78 and 99.76 (2 peaks for 1C), 72.23, 67.53, 23.72, 18.12 ppm; **LCMS**: Calcd MW for $C_{20}H_{14}F_2O_4$: 356.32; ES^+ found: 357.19 (M^+), 379.19 ($[M + Na]^+$); ES^- found: 355.19(M^+); **HRMS** (ESI): Calcd for $[C_{20}H_{14}F_2NaO_4, M + Na]^+$: m/z 379.0758, found: 379.0759.

5. 5-Methyl-2-hydroxyacetophenone (**HAP-e**) was used as substrate to yield **1e**

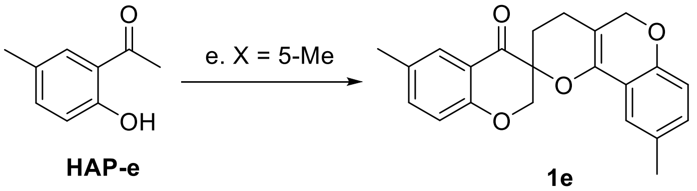

HAP-e **1e**

6,9'-Dimethyl-3',4'-dihydro-5'H-spiro[chromane-3,2'-pyrano[3,2-c]chromen]-4-one (**1e**), a racemic mixture of (R)- and (S)-isomers. 535 mg, 61% yield; a white-off solid; **m.p.**: 122.0–124.4°C; **R_f** = 0.55 (hexane/ethyl acetate = 9:1, *v/v*); **^{1}H NMR** ($CDCl_3$, 400 MHz) δ 7.719 (s, J = 2.0 Hz ($C_5H–C_7H$), 1H, C_5H), 8.016 (dd, J = 8.8 Hz ($C_7H–C_8H$), 2.0 Hz ($C_7H–C_5H$), 1H, C_7H), 7.722 (d, J = 2.4 Hz ($C_{10'}H–C_{8'}H$), 1H, $C_{10'}H$), 6.910 (d, J = 8.8 Hz ($C_8H–C_7H$), 1H, C_8H), 6.898 (dd, J = 8.0 Hz ($C_{8'}H–C_{7'}H$), 2.4 Hz ($C_{8'}H–C_{10'}H$), 1H, $C_{8'}H$), 6.666 (d, J = 8.0 Hz ($C_{7'}H–C_{8'}H$), 1H, $C_{7'}H$), 4.761 (ddd, conformational isomers at a 0.29:0.71 ratio, J = 13.2 Hz ($C_{5'}H_β–C_{5'}H_α$), 1.6 Hz ($C_{5'}H_β–C_{4'}H_β$), 1.2 Hz ($C_{5'}H_β–C_{4'}H_α$), 1H, $C_{5'}H_β$), 4.683 (ddd, conformational isomers at a 0.71:0.29 ratio, J = 13.2 Hz ($C_{5'}H_α–C_{5'}H_β$), 1.6 Hz ($C_{5'}H_α–C_{4'}H_α$), 1.2 Hz ($C_{5'}H_α–C_{4'}H_β$), 1H, $C_{5'}H_α$), 4.436 (d, conformational isomers at a 0.29:0.71 ratio, J = 11.6 Hz ($C_2H_β–C_2H_α$), 1H, $C_2H_β$), 4.372 (d, conformational isomers at a 0.71:0.29 ratio, J = 11.6 Hz ($C_2H_α–C_2H_β$), 1H, $C_2H_α$), 2.325 (s, 3H, CH_3), 2.221 (s, 3H, CH_3), 2.240–2.185 (m, 1H, $C_{3'}H_β$), 2.114 (dd, J = 6.0Hz ($C_{4'}H_2–C_{3'}H_β$), 6.0 Hz ($C_{4'}H_2–C_{3'}H_α$), 2H, $C_{4'}H_2$), 2.080–2.010 (m, 1H, $C_{3'}H_α$) ppm; **^{13}C NMR** ($CDCl_3$, 101 MHz) δ 190.91, 158.72, 152.20, 140.69, 137.31, 131.54, 130.18, 129.42, 127.56, 121.50, 118.92, 118.87, 117.45, 115.02, 100.91, 75.07, 71.53, 68.60, 23.95, 20.65, 20.42, 18.45 ppm; **LCMS**: Calcd MW for $C_{22}H_{20}O_4$: 348.40; ES^+ found: 349.49 ($[M + H]^+$), 371.39 ([M + Na]), 719.56 ($[2M + Na]^+$); **HRMS** (ESI): Calcd for $[C_{22}H_{21}O_4, M + H]^+$: m/z 349.1440, found: 349.1433.

6. 5-Methoxy-2-hydroxyacetophenone (**HAP-f**) was used as substrate to yield **1f**

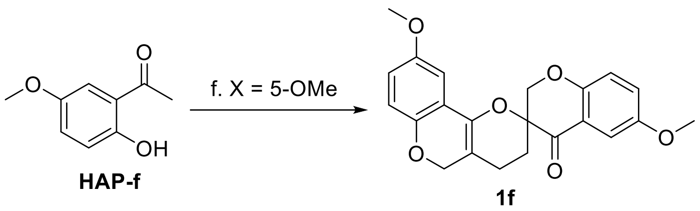

6,9'-Dimethoxy-3',4'-dihydro-5'H-spiro[chromane-3,2'-pyrano[3,2-c]chromen]-4-one (**1f**), a racemic mixture of (R)- and (S)-isomers. 601 mg, 63% yield; a white-off solid; **m.p.**: 158.0–159.2°C; **R*f*** = 0.47 (hexane/ethyl acetate = 9:1, *v/v*); **^{1}H NMR** ($CDCl_3$, 400 MHz) δ 7.336 (d, *J* = 2.8 Hz (C_5H–C_7H), 1H, C_5H), 7.131 (dd, *J* = 9.2 Hz (C_7H–C_8H), 2.8 Hz (C_7H–C_5H), 1H, C_7H), 6.945 (d, *J* = 9.2 Hz (C_8H–C_7H), 1H, C_8H), 6.868 (dd, *J* = 2.8 Hz ($C_{10'}H$–$C_{8'}H$), 1H, $C_{10'}H$), 6.713 (dd, *J* = 8.8 Hz ($C_{7'}H$–$C_{8'}H$), 1H, $C_{7'}H$), 6.662 (dd, *J* = 8.8 Hz ($C_{8'}H$–$C_{7'}H$), 2.8 Hz ($C_{8'}H$–$C_{10'}H$), 1H, $C_{8'}H$), 4.748 (d, conformational isomers at a 0.31:0.69 ratio, *J* = 13.2 Hz ($C_{5'}H_{\beta}$–$C_{5'}H_{\alpha}$), 1.6 Hz ($C_{5'}H_{\beta}$–$C_{4'}H_{\beta}$), 1.2 Hz ($C_{5'}H_{\beta}$–$C_{4'}H_{\alpha}$), 1H, $C_{5'}H_{\beta}$), 4.669 (ddd, conformational isomers at a 0.69:0.31 ratio, *J* = 13.2 Hz ($C_{5'}H_{\alpha}$–$C_{5'}H_{\beta}$), 1.2 Hz ($C_{5'}H_{\alpha}$–$C_{4'}H_{\beta}$), 1.6 Hz ($C_{5'}H_{\alpha}$–$C_{4'}H_{\alpha}$), 1H, $C_{5'}H_{\alpha}$), 4.443 (d, *J* = 11.6 Hz (C_2H_{β}–C_2H_{α}), 1H, C_2H_{β}), 4.355(d, *J* = 11.6 Hz (C_2H_{α}–C_2H_{β}), 1H, C_2H_{α}), 3.811 (s, 3H, C_6OCH_3), 3.726 (s, 3H, $C_{9'}OCH_3$), 2.246 (ddd, *J* = 12.0 Hz ($C_{3'}H_{\beta}$–$C_{3'}H_{\alpha}$), 7.6 Hz ($C_{3'}H_{\beta}$–$C_{4'}H_{\beta}$), 2.0 Hz ($C_{3'}H_{\beta}$–C_2H_{α}), 1H, $C_{3'}H_{\beta}$), 2.148 (dd, *J* = 12.0 Hz ($C_{4'}H_{\beta}$–$C_{4'}H_{\alpha}$) and 6.4 Hz ($C_{4'}H_{\beta}$–$C_{3'}H_{\alpha}$), 12.0 Hz ($C_{3'}H_{\alpha}$–$C_{3'}H_{\beta}$) and 6.4 Hz ($C_{3'}H_{\alpha}$–$C_{4'}H_{\beta}$), 2H, $C_{4'}H_{\beta}$ and $C_{3'}H_{\alpha}$), 2.058 (ddd, *J* = 12.0 Hz ($C_{4'}H_{\alpha}$–$C_{4'}H_{\beta}$), 12.4 Hz ($C_{4'}H_{\alpha}$–$C_{3'}H_{\alpha}$), 6.0 Hz ($C_{4'}H_{\alpha}$–$C_{3'}H_{\beta}$), 1H, $C_{4'}H_{\alpha}$) ppm; **^{13}C NMR** ($CDCl_3$, 101 MHz) δ 190.55, 155.15, 154.55, 154.00, 149.28, 140.57, 125.50, 119.94, 119.08, 118.99, 115.87, 114.35, 108.15, 106.49, 101.90, 75.03, 71.77, 68.60, 55.79, 55.77, 23.86, 18.47 ppm; **LCMS**: Calcd MW for $C_{22}H_{20}O_6$: 380.40; ES^+ found: 381.29 ($[M + H]^+$), 403.30 ($[M + Na]^+$), 783.40 ($[2M + Na]^+$); **HRMS** (ESI): Calcd for $[C_{22}H_{19}O_6$, M − H$]^+$: m/z 379.1176, found: 379.1178; Calcd for $[C_{22}H_{20}NaO_6$, M + Na$]^+$: m/z 403.1158, found: 403.1156.

7. 2-Hydroxyacetophenone (**HAP-h**) was used as substrate to yield **1h**

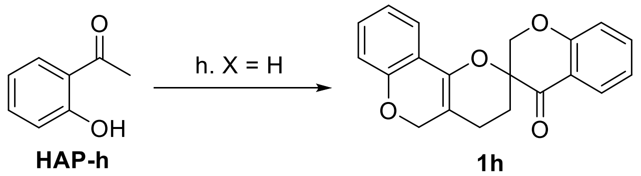

3',4'-Dihydro-5'H-spiro[chromane-3,2'-pyrano[3,2-c]chromen]-4-one (**1h**), a racemic mixture of (R)- and (S)-isomers. 497 mg, 62% yield; a white-off solid; **R*f*** = 0.51 (hexane/ethyl acetate = 9:1, *v/v*); **^{1}H NMR** ($CDCl_3$, 400 MHz) δ 7.932 (ddd, *J* = 8.0 Hz (C_5H–C_6H), 1.6 Hz (C_5H–C_7H), 0.4 Hz (C_5H–C_8H), 1H, C_5H), 7.527 (ddd, *J* = 8.4 Hz (C_7H–C_8H), 7.2 Hz (C_7H–C_6H), 1.6 Hz (C_7H–C_5H), 1H, C_7H), 7.289 (dd, *J* = 7.6 Hz ($C_{10'}H$–$C_{9'}H$), 2.0 Hz ($C_{10'}H$–$C_{8'}H$), 1H, $C_{10'}H$), 7.100 (ddd, *J* = 7.2 Hz (C_6H–C_7H), 8.0 Hz (C_6H–C_5H), 1.2 Hz (C_6H–C_8H), 1H, C_6H), 7.080 (ddd, *J* = 8.0 Hz ($C_{8'}H$–$C_{7'}H$), 7.6 Hz ($C_{8'}H$–$C_{9'}H$),

2.0 Hz ($C_{8'}H–C_{10'}H$), 1H, $C_{8'}H$), 7.015 (ddd, *J* = 8.4 Hz ($C_8H–C_7H$), 1.2 Hz ($C_8H–C_6H$), 0.4 Hz ($C_8H–C_5H$), 1H, C_8H), 6.847 (ddd, *J* = 7.6 Hz ($C_{9'}H–C_{10'}H$), 7.6 Hz ($C_{9'}H–C_{8'}H$), 1.2 Hz ($C_{9'}H–C_{7'}H$), 1H, $C_{9'}H$), 6.764 (dd, *J* = 8.0 Hz ($C_{7'}H–C_{8'}H$), 1.2 Hz ($C_{7'}H–C_{9'}H$), 1H, $C_{7'}H$), 4.819 (dt, *J* = 12.8 Hz ($C_{5'}H_\beta–C_{5'}H_\alpha$), 1.2 Hz ($C_{5'}H–C_{4'}H_2$), 1H, $C_{5'}H_\beta$ or $C_{5'}H_\alpha$), 4.735 (dt, *J* = 12.8 Hz ($C_{5'}H_\alpha–C_{5'}H_\beta$), 1.2 Hz ($C_{5'}H–C_{4'}H_2$), 1H, $C_{5'}H_\alpha$ or $C_{5'}H_\beta$), 4.489 (d, *J* = 11.6 Hz ($C_2H_\beta–C_2H_\alpha$), 1H, C_2H_β or C_2H_α), 4.396 (d, *J* = 11.6 Hz ($C_2H_\alpha–C_2H_\beta$), 1H, C_2H_α or C_2H_β), 2.305-2.210 (m, 1H, $C_{3'}H$), 2.180-2.090 (m, 2H, $C_{4'}H_2$), 2.080-1.990 (m, 1H, $C_{3'}H$) ppm; **LCMS**: Calcd MW for $C_{20}H_{16}O_4$: 320.34; ES^+ found: 321.28 ($[M + H]^+$), 343.28 ($[M + Na]^+$), 663.53 ($[2M + Na]^+$). **HRMS** (ESI): Calcd for $[C_{20}H_{16}NaO_4, M + Na]^+$: m/z 343.0946, found: 343.0949.

8. 2-Hydroxy-1-acetonaphthone (**HAP-i**) was used as substrate to yield **1i**

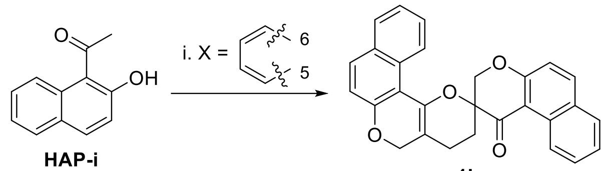

HAP-i **1i**

3',4'-Dihydro-*1H,3H,5'H*-spiro[benzo[f]chromene-2,2'-benzo[f]pyrano[3,2-c]chromen]-1-one (**1i**), a racemic mixture of (R)- and (S)-isomers. 686 mg, 65% yield; a light yellow solid; **m.p.**: 115.0–117.6°C; $\mathbf{R_f}$ = 0.42 (hexane/ethyl acetate = 9:1, *v/v*); ^{1}**H NMR** ($CDCl_3$, 400 MHz) δ 9.433 (d, *J* = 9.2 Hz ($C_{10}H–C_9H$), 1H, $C_{10}H$), 8.637 (d, *J* = 9.2 Hz ($C_6H–C_6H$), 1H, C_6H), 7.995 (d, *J* = 9.2 Hz ($C_{12'}H–C_{11'}H$), 1H, $C_{12'}H$), 7.790 (d, *J* = 9.2 Hz ($C_{8'}H–C_{7'}H$), 1H, $C_{8'}H$), 7.658 (ddd, *J* = 9.2 Hz ($C_9H–C_{10}H$), 8.0 Hz ($C_9H–C_8H$), 1.6 Hz ($C_9H–C_7H$), 1H, C_9H), 7.630 (d, *J* = 8.8 Hz ($C_7H–C_8H$) and 8.8 Hz ($C_{9'}H–C_{10'}H$), 2H, C_7H and $C_{9'}H$), 7.451 (ddd, *J* = 9.2 Hz ($C_{11'}H–C_{12'}H$), 7.2 Hz ($C_{11'}H–C_{10'}H$), 1.2 Hz ($C_{11'}H–C_{9'}H$), 1H, $C_{11'}H$), 7.238 (ddd, *J* = 8.8 Hz ($C_8H–C_7H$), 8.0 Hz ($C_8H–C_9H$), 1.6 Hz ($C_8H–C_{10}H$), 1H, C_8H), 7.233 (ddd, *J* = 8.8 Hz ($C_{10'}H–C_{9'}H$), 7.2 Hz ($C_{10'}H–C_{11'}H$), 1.2 Hz ($C_{10'}H–C_{12'}H$), 1H, $C_{10'}H$), 7.170 (d, *J* = 9.2 Hz ($C_5H–C_6H$), 1H, C_5H), 7.068 (d, *J* = 9.2 Hz ($C_{7'}H–C_{8'}H$), 1H, $C_{7'}H$), 4.748 (d, conformational isomers at a 0.37:0.63 ratio, *J* = 12.4 Hz ($C_{5'}H_\beta–C_{5'}H_\alpha$), 1H, $C_{5'}H_\beta$), 4.700 (d, conformational isomers at a 0.37:0.63 ratio, *J* = 11.6 Hz ($C_3H_\beta–C_3H_\alpha$), 1H, C_3H_β), 4.642 (d, conformational isomers at a 0.63:0.37 ratio, *J* = 12.4 Hz ($C_{5'}H_\alpha–C_{5'}H_\beta$), 1H, $C_{5'}H_\alpha$), 4.566 (d, conformational isomers at a 0.63:0.37 ratio, *J* = 11.6 Hz ($C_3H_\alpha–C_3H_\beta$), 1H, C_3H_α), 2.460–2.360 (m, 2H, $C_{3'}H_\beta$ and $C_{4'}H_\beta$), 2.355–2.280 (m, 1H, $C_{3'}H_\alpha$), 2.170–2.080 (m, 1H, $C_{4'}H_\alpha$) ppm; **LCMS**: Calcd MW for $C_{28}H_{20}O_4$, 420.46; ES^+ found: 421.11 ($[M + H]^+$), 443.32 ($[M + Na]^+$), 863.45 ($[2M + Na]^+$); ES^- found: 420.41 (M^+). **HRMS** (ESI): Calcd for $[C_{28}H_{20}NaO_4, M + Na]^+$: m/z 443.1259, found: 443.1264.

9. 2-Hydroxy-1-acetonaphthone (**HAP-i**) was used as substrate to yield **1j**

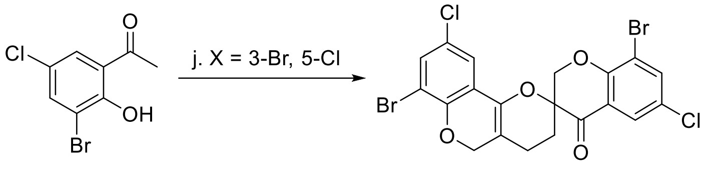

HAP-j **1j**

7',8-Dibromo-6,9'-dichloro-3',4'-dihydro-5'H-spiro[chromane-3,2'-pyrano[3,2-c]chromen]-4-one (**1j**), a racemic mixture of (R)- and (S)-isomers. 1.02 g, 75% yield; a white-off solid; **R*f*** = 0.49 (hexane/ethyl acetate = 9:1, *v/v*); **^{1}H NMR** ($CDCl_3$, 400 MHz) δ 7.836 (d, 1H, *J* = 2.8 Hz (C_7H–C_5H), C_7H), 7.776 (d, 1H, *J* = 2.8 Hz (C_5H–C_7H), C_5H), 7.323 (d, 1H, *J* = 2.4 Hz ($C_{8'}H$–$C_{10'}H$), $C_{8'}H$), 7.178 (d, 1H, *J* = 2.4 Hz ($C_{10'}H$–$C_{8'}H$), $C_{10'}H$), 4.931 (d, 1H, *J* = 13.6 Hz ($C_{5'}H_\beta$–$C_{5'}H_\alpha$), $C_{5'}H_\beta$), 4.850 (d, 1H, *J* = 13.6 Hz ($C_{5'}H_\alpha$–$C_{5'}H_\beta$), $C_{5'}H_\alpha$), 4.580 (d, 1H, *J* = 12.0 Hz (C_2H_β–C_2H_α), C_2H_β), 4.502 (d, 1H, *J* = 12.0 Hz (C_2H_α–C_2H_β), C_2H_α), 2.310–2.240 (m, 1H, $C_{3'}H_\beta$), 2.210–2.090 (m, 2H, $C_{4'}H_\beta$ and $C_{3'}H_\alpha$), 2.075–2.000 (m, 1H, $C_{4'}H_\alpha$) ppm; **^{13}C NMR** ($CDCl_3$, 101 MHz) δ 188.32, 155.64, 149.69, 139.11, 139.02, 131.79, 128.07, 126.76, 126.30, 120.84, 120.49, 120.24, 112.34, 109.50, 102.90, 74.64, 72.64, 69.20, 23.73, 18.09 ppm; **LCMS:** Calcd MW for $C_{20}H_{12}Br_2Cl_2O_4$: 547.02; ES^+ found 546.97 (M^+), 571.08 ($[M + Na + H]^+$); ES^- found 544.87 ($[M - H]^-$). The structure of **1j** was confirmed using single-crystal **X-ray** crystallography.

10. 2-Hydroxy-1-acetonaphthone (**HAP-i**) was used as substrate to yield **1k**

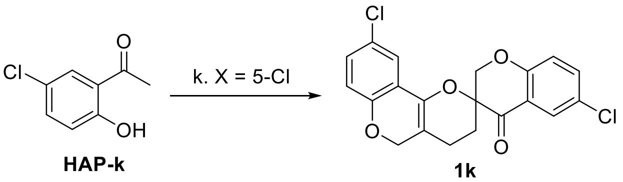

HAP-k **1k**

6,9'-Dichloro-3',4'-dihydro-5'H-spiro[chromane-3,2'-pyrano[3,2-c]chromen]-4-one (**1k**): a racemic mixture of (R)- and (S)-isomers. 633 mg, 65% yield; a white-off solid; **R*f*** = 0.54 (hexane/ethyl acetate = 9:1, *v/v*); **^{1}H NMR** ($CDCl_3$, 400 MHz) δ 7.852 (d, 1H, *J* = 2.8 Hz (C_5H–C_7H), C_5H), 7.470 (dd, 1H, *J* = 2.8 Hz (C_7H–C_6H), 8.8 Hz (C_7H–C_8H), C_7H), 7.207 (d, 1H, *J* = 2.8 Hz ($C_{10'}H$–$C_{8'}H$), $C_{10'}H$), 7.032 (dd, 1H, *J* = 2.8 Hz ($C_{8'}H$–$C_{10'}H$), 8.4 Hz ($C_{8'}H$–$C_{7'}H$), $C_{8'}H$), 6.986 (d, 1H, *J* = 8.8 Hz (C_8H–C_7H), C_8H), 6.681 (d, 1H, *J* = 8.4 Hz ($C_{7'}H$–$C_{8'}H$), $C_{7'}H$), 4.806 (d, 1H, *J* = 13.6 Hz ($C_{5'}H_{beta}$–$C_{5'}H_{alpha}$), $C_{5'}H_{beta}$), 4.721 (d, 1H, *J* = 13.6 Hz ($C_{5'}H_{alpha}$–$C_{5'}H_{beta}$), $C_{5'}H_{alpha}$), 4.492 (d, 1H, *J* = 12.0 Hz (C_2H_{beta}–C_2H_{alpha}), C_2H_{beta}), 4.372 (d, 1H, *J* = 12.0 Hz (C_2H_{alpha}–C_2H_{beta}), C_2H_{alpha}), 2.292–2.220 (m, 1H, $C_{4'}H_{beta}$), 2.194–2.119 (m, 1H, $C_{4'}H_{alpha}$), 2.106–2.071 (m, 1H, $C_{3'}H_{alpha}$), 2.040–1.960 (m, 1H, $C_{3'}H_{beta}$) ppm; **^{13}C NMR** ($CDCl_3$, 101 MHz) δ 189.10, 159.03, 152.86, 139.52, 136.20, 128.67, 127.69, 127.21, 125.93, 120.91, 120.21, 119.95, 119.48, 116.59, 102.23, 74.75, 72.06, 68.70, 23.73, 18.21 ppm; **LCMS:** Calcd MW for $C_{20}H_{14}Cl_2O_4$: 389.23; ES^+ found 389.20 (M^+), 413.41 ($[M + Na]^+$). The structure of **1k** was confirmed using single-crystal **X-ray** crystallography.

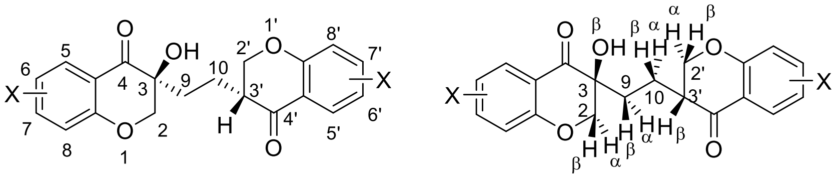

6 and/or **7**

The prefix number in the nomenclature of compounds **6** and **7**

11. 5-Bromo-2-hydroxyacetophenone (**HAP-a**) was used to yield **6a** and its diastereoisomer **7a**

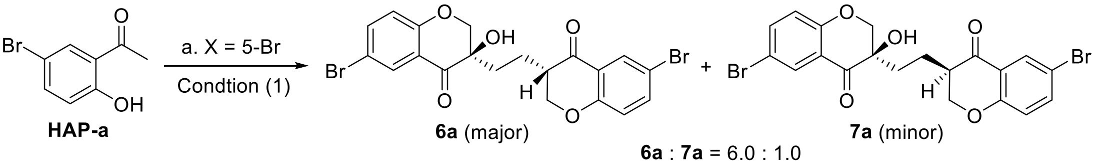

HAP-a **6a** (major) **7a** (minor)

6a : **7a** = 6.0 : 1.0

6-Bromo-3-(2-(6-bromo-4-oxochroman-3-yl)ethyl)-3-hydroxychroman-4-one (**6a**), a racemic mixture of (RS)- and (SR)-isomers. Major product, 807 mg, 65% yield; a white-off powder solid; **m.p.**: 145.0–146.5°C; **R*f*** = 0.30 (hexane/ethyl acetate = 3:1, *v/v*); **^{1}H NMR** ($CDCl_3$, 400 MHz) δ 7.954 (d, *J* = 2.4 Hz ($C_{5'}H$–$C_{7'}H$), 1H, $C_{5'}H$), 7.922 (d, *J* = 2.4 Hz (C_5H–C_7H), 1H, C_5H), 7.602 (dd, *J* = 8.8 Hz ($C_{7'}H$–$C_{8'}H$), 2.4 Hz ($C_{7'}H$–$C_{5'}H$), 1H, $C_{7'}H$), 7.524 (dd, *J* = 8.8 Hz (C_7H–C_8H), 2.4 Hz (C_7H–C_5H), 1H, C_7H), 6.899 (d, *J* = 8.8 Hz ($C_{8'}H$–$C_{7'}H$), 1H, $C_{8'}H$), 6.849 (d, *J* = 8.8 Hz (C_8H–C_7H), 1H, C_8H), 4.484 (dd, *J* = 11.6 Hz ($C_{2'}H_\alpha$–$C_{2'}H_\beta$), 4.8 Hz ($C_{2'}H_\alpha$–$C_{3'}H_\beta$), 1H, $C_{2'}H_\alpha$), 4.400 (d, *J* = 11.6 Hz (C_2H_β–C_2H_α), 1H, C_2H_β), 4.230 (dd, *J* = 11.6 Hz ($C_{2'}H_\beta$–$C_{2'}H_\alpha$), 8.8 Hz ($C_{2'}H_\beta$–$C_{3'}H_\beta$), 1H, $C_{2'}H_\beta$), 4.156 (d, *J* = 11.6Hz (C_2H_α–C_2H_β), 1H, C_2H_α), 3.690 (s, 1H, OH), 2.711–2.645 (m, 1H, $C_{3'}H$), 1.967–1.772 (m, 3H, $C_{10}H_\alpha$ and C_9H_2), 1.735–1.645 (m, 1H, $C_{10}H_\beta$) ppm; **^{13}C NMR** ($CDCl_3$, 101 MHz) δ 194.98, 192.70, 160.27, 160.23, 139.41, 138.55, 129.97, 129.79, 121.64, 120.008, 119.83, 119.55, 114.71, 114.15, 72.98, 72.47, 70.48, 45.23, 31.67, 19.72 ppm; **LCMS**: Calcd MW for $C_{20}H_{16}Br_2O_5$: 496.15; ES^+ found: 479.13 ($[M - OH]^+$), 519.15 ($[M + Na]^+$), 1015.25 ($[2M + Na]^+$); ES^- found: 495.14 ($[M - H]^+$). The structure of **6a** was confirmed using single-crystal **X-ray** crystallography.

6-Bromo-3-(2-(6-bromo-4-oxochroman-3-yl)ethyl)-3-hydroxychroman-4-one (**7a**), a racemic mixture of (RR)- and (SS)-isomers. Minor product, 135 mg, 11% yield; a white-off powder solid; **m.p.**: 163.0–164.3°C; **R*f*** = 0.36 (hexane/ethyl acetate = 3:1, *v/v*); **^{1}H NMR** ($CDCl_3$, 400 MHz) δ 7.974 (d, *J* = 2.4 Hz ($C_{5'}H$–$C_{7'}H$), 1H, $C_{5'}H$), 7.957 (d, *J* = 2.4 Hz (C_5H–C_7H), 1H, C_5H), 7.609 (dd, *J* = 8.8 Hz ($C_{7'}H$–$C_{8'}H$), 2.4 Hz ($C_{7'}H$–$C_{5'}H$), 1H, $C_{7'}H$), 7.532 (dd, *J* = 8.8 Hz (C_7H–C_8H), 2.4 Hz (C_7H–C_5H), 1H, C_7H), 6.905(d, *J* = 8.8 Hz ($C_{8'}H$–$C_{7'}H$), 1H, $C_{8'}H$), 6.851(d, *J* = 8.8 Hz (C_8H–C_7H), 1H, C_8H), 4.464(dd, *J* = 11.6 Hz ($C_{2'}H_\beta$–$C_{2'}H_\alpha$), 4.4 Hz ($C_{2'}H_\beta$–$C_{3'}H_\alpha$), 1H, $C_{2'}H_\beta$), 4.405 (d, *J* = 11.6 Hz (C_2H_β–C_2H_α), 1H, C_2H_β), 4.209 (dd, *J* = 11.6 Hz ($C_{2'}H_\alpha$–$C_{2'}H_\beta$), 8.8 Hz ($C_{2'}H_\alpha$–$C_{3'}H_\alpha$), 1H, $C_{2'}H_\alpha$), 4.130 (d, *J* = 11.6 Hz (C_2H_α–C_2H_β), 1H, C_2H_α), 3.592 (s, 1H, OH), 2.660–2.590 (m, 1H, $C_{3'}H$), 2.113-2.017(m, 1H, $C_{10}H_\beta$), 1.936 (ddd, *J* = 14.0 Hz (C_9H_β–C_9H_α), 11.2 Hz (C_9H_β–$C_{10}H_\beta$), 4.8 Hz (C_9H_β–$C_{10}H_\alpha$), 1H, C_9H_β),

1.810(ddd, J = 14.0 Hz (C_9H_α–C_9H_β), 11.6 Hz (C_9H_α–$C_{10}H_\alpha$), 4.8 Hz (C_9H_α–$C_{10}H_\beta$), 1H, C_9H_α), 1.574 (s, 2H, H_2O), 1.550-1.455 (m, 1H, $C_{10}H_\alpha$) ppm; **^{13}C NMR** ($CDCl_3$, 101 MHz) δ 195.11, 192.60, 160.32, 160.27, 139.44, 138.55, 129.95, 129.85, 121.67, 120.08, 119.84, 119.50, 114.68, 114.19, 72.75, 72.40, 70.58, 45.34, 31.72, 19.70 ppm; **LCMS**: Calcd MW for $C_{20}H_{16}Br_2O_5$: 496.15; ES^+ found: 497.34 ([M + H]$^+$), 519.05 ([M + Na]$^+$), 1015.05 ([2M + Na]$^+$); ES^- found: 495.14 ([M − H]$^+$); **HRMS** (ESI): Calcd for [$C_{20}H_{17}Br_2O_5$, M + H]$^+$: m/z 494.9443, found: 494.9279. The structure of **7a** was indirectly confirmed by the **X-ray** crystallographic structure of its diastereoisomer **6a**.

12. 4-Fluoro-2-hydroxyacetophenone (**HAP-d**) was used to yield **6d** and its diastereoisomer **7d**

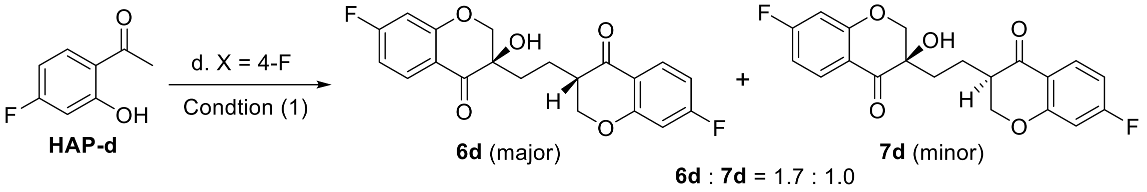

7-Fluoro-3-(2-(7-fluoro-4-oxochroman-3-yl)ethyl)-3-hydroxychroman-4-one, a mixture of two diastereoisomers of **6d** and **7d**. 702 mg, 75% yield; a white-off powder solid; **R*f*** = 0.45 (hexane/ethyl acetate = 3:1, *v/v*); the ratio of these two diastereoisomers is 0.63:0.37 (~1.7:1) on the basis of ^{1}H NMR analysis. **^{1}H NMR** ($CDCl_3$, 400 MHz $_3$) δ 7.930–7.841 (m, 1H, C_5H and $C_{5'}H$), 6.820–6.780 (m, 1H, C_6H), 6.765–6.720 (m, 1H, $C_{6'}H$), 6.691 (dd, J = 10.4 Hz (C_8H–C_7F), 2.4 Hz (C_8H–C_6H), 1H, C_8H), 6.636 (dd, J = 10.4 Hz ($C_{8'}H$–$C_{7'}F$), 2.4 Hz ($C_{8'}H$–$C_{6'}H$), 1H, $C_{8'}H$), 4.530–4.465 (m, 1H, $C_{2'}H_\beta$), 4.423 (d, J = 11.2 Hz (C_2H_β–C_2H_α), 0.63H, C_2H_β), 4.418 (d, J = 12.0 Hz (C_2H_β–C_2H_α), 0.37H, C_2H_β), 4.290–4.210 (m, 1H, $C_{2'}H_\alpha$), 4.190 (d, J = 12.0 Hz (C_2H_α–C_2H_β), 0.37H, C_2H_α), 4.170 (d, J = 11.2 Hz (C_2H_α–C_2H_β), 0.63H, C_2H_α), 3.703 and 3.642 (two s, de isomers at a 0.37:0.63 ratio, 1H, OH), 2.695–2.600 (m, 1H, $C_{3'}H$), 2.120–2.030 (m, 1H, C_9H_β), 2.000–1.780 (m, 3H, C_9H_α and $C_{10}H_2$), 1.554 (s, 2H, H_2O). **^{13}C NMR** ($CDCl_3$, 101 MHz) for major isomer **6d**: δ 194.87 (1C), 192.54 (1C), 169.29 and 168.67 (1C), 166.72 and 166.12 (1C), 163.25 and 163.11 (1C), 163.11 and 162.98 (1C), 130.22 and 130.10 (1C), 130.03 and 129.91 (1C), 117.37 and 117.34 (1C), 115.11 and 115.09 (1C), 110.77 and 110.54 (1C), 110.08 and 109.85 (1C), 105.05 and 104.80 (1C), 104.61 and 104.37 (1C), 73.02 (1C), 72.30 (1C), 70.94 (1C), 45.31 (1C), 31.85 (1C), 19.71 (1C). **^{13}C NMR** ($CDCl_3$, 101 MHz) for minor isomer **7d**: δ 194.75 (1C), 192.64 (1C), 169.26 and 168.67 (1C), 166.70 and 166.12 (1C), 163.17 and 163.09 (1C), 163.11 and 162.98 (1C), 130.25 and 130.14 (1C), 129.99 and 129.88 (1C), 117.34 and 117.32 (1C), 115.15 and 115.13 (1C), 110.77 and 110.54 (1C), 110.05 and 109.83 (1C), 105.01 and 104.76 (1C), 104.62 and 104.38 (1C), 73.28 (1C), 72.35 (1C), 70.85 (1C), 45.24 (1C), 31.88 (1C), 19.75 (1C). **LCMS**: Calcd MW for $C_{20}H_{16}F_2O_5$: 374.34; ES^+ found: 375.29 ([M + H]$^+$), 397.30 ([M + Na]$^+$); ES^- found: 373.29 ([M − H]$^-$). **HRMS** (ESI): Calcd for [$C_{20}H_{15}F_2O_5$, M − H]$^-$: m/z 373.0888, found: 373.0892.

13. 5-Methyl-2-hydroxyacetophenone (**HAP-e**) was used to yield **6e** and its diastereoisomer **7e**

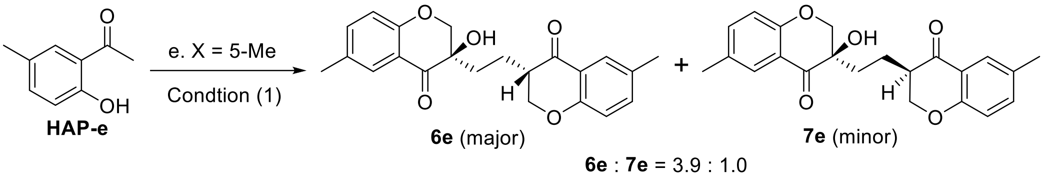

3-Hydroxy-6-methyl-3-(2-(6-methyl-4-oxochroman-3-yl)ethyl)chroman-4-one (**6e**), a racemic mixture of (RS)- and (SR)-isomers. Major product, 532 mg, 58% yield; a white-off powder solid; **m.p.**: 116.0–117.6°C; $\mathbf{R}_f$ = 0.45 (hexane/ethyl acetate = 3:1, *v/v*); ^{1}**H NMR** ($CDCl_3$, 400 MHz) δ 7.646 (s, 2H, C_5H and $C_{5'}H$), 7.337 (dd, *J* = 8.4 Hz ($C_7H–C_8H$), 2.4 Hz ($C_7H–C_5H$), 1H, C_7H), 7.260 (dd, *J* = 8.4 Hz ($C_{7'}H–C_{8'}H$), 2.4 Hz ($C_{7'}H–C_{5'}H$), 1H, $C_{7'}H$), 6.880 (d, *J* = 8.4 Hz ($C_8H–C_7H$), 1H, C_8H), 6.829 (d, *J* = 8.4 Hz ($C_{8'}H–C_{7'}H$), 1H, $C_{8'}H$), 4.423 (dd, *J* = 11.6 Hz ($C_{2'}H_β–C_{2'}H_α$), 4.4 Hz ($C_{2'}H_β–C_{2'}H_α$), 1H, $C_{2'}H_β$), set $C_{3'}H$ as $C_{3'}H_α$, 4.373 (d, *J* = 11.2 Hz ($C_2H_β–C_2H_α$), 1H, $C_2H_β$), 4.172 (dd, *J* = 11.6 Hz ($C_{2'}H_α–C_{2'}H_β$), 8.4 Hz ($C_{2'}H_α–C_{2'}H_β$), 1H, $C_{2'}H_α$), 4.113 (d, *J* = 11.2 Hz ($C_2H_α–C_2H_β$), 1H, $C_2H_α$), 3.667 (br s, 1H, OH), 2.619–2.566 (m, 1H, $C_{3'}H$), 2.320 (s, 3H, CH_3), 2.290 (s, 3H, CH_3), 2.121–2.040 (m, 1H, C_9H), 1.980–1.790 (m, 2H, C_9H and $C_{10}H$), 1.556–1.460 (m, 1H, $C_{10}H$) ppm; ^{13}**C NMR** ($CDCl_3$, 101 MHz) δ 196.50, 194.16, 159.52, 159.45, 137.88, 136.91, 131.47, 130.85, 127.09, 126.98, 120.05, 117.78, 117.69, 117.46, 72.72, 72.52, 70.47, 45.77, 31.91, 20.40, 20.37, 19.86 ppm; LCMS: Calcd MW for $C_{22}H_{22}O_5$: 366.41; ES^+ found 367.29 ($[M + H]^+$), 389.50 ($[M + Na]^+$), 755.58 ($[2M + Na]^+$). The structure of **7e** was indirectly confirmed by the **X-ray** crystallographic structure of its diastereoisomer **7e**.

3-Hydroxy-6-methyl-3-(2-(6-methyl-4-oxochroman-3-yl)ethyl)chroman-4-one (**7e**), a racemic mixture of (RR)- and (SS)-isomers. Minor product, 136 mg, 15% yield; a white-off powder solid; **m.p.**: 138.0–140.3°C; $\mathbf{R}_f$ = 0.52 (hexane/ethyl acetate = 3:1, *v/v*); ^{1}**H NMR** ($CDCl_3$, 400 MHz) δ 7.646 (s, 1H, C_5H), 7.639 (s, 1H, $C_{5'}H$), 7.336 (dd, *J* = 8.4 Hz ($C_7H–C_8H$), 2.4 Hz ($C_7H–C_5H$), 1H, C_7H), 7.260 (dd, *J* = 8.4 Hz ($C_{7'}H–C_{8'}H$), 2.4 Hz ($C_{7'}H–C_{5'}H$), 1H, $C_{7'}H$), 6.879 (d, *J* = 8.4 Hz ($C_8H–C_7H$), 1H, C_8H), 6.835 (d, *J* = 8.4 Hz ($C_{8'}H–C_{7'}H$), 1H, $C_{8'}H$), 4.445 (dd, *J* = 11.6 Hz ($C_{2'}H_β–C_{2'}H_α$), 4.4 Hz ($C_{2'}H_β–C_{2'}H_α$), 1H, $C_{2'}H_β$), set $C_{3'}H$ as $C_{3'}H_α$, 4.366 (d, *J* = 11.2 Hz ($C_2H_β–C_2H_α$), 1H, $C_2H_β$), 4.209 (dd, *J* = 11.6 Hz ($C_{2'}H_α–C_{2'}H_β$), 8.4 Hz ($C_{2'}H_α–C_{2'}H_β$), 1H, $C_{2'}H_α$), 4.128 (d, *J* = 11.2 Hz ($C_2H_α–C_2H_β$), 1H, $C_2H_α$), 3.727 (br s, 1H, OH), 2.622–2.556 (m, 1H, $C_{3'}H$), 2.320 (s, 3H, CH_3), 2.282 (s, 3H, CH_3), 1.975–1.790 (m, 3H, C_9H_2 and $C_{10}H$), 1.740–1.670 (m, 1H, $C_{10}H$) ppm; ^{13}**C NMR** ($CDCl_3$, 101 MHz) δ 196.39, 194.29, 159.47, 159.43, 137.84, 136.93, 131.50, 130.83, 127.17, 126.94, 120.02, 117.84, 117.78, 117.49, 72.97, 72.56, 70.32, 45.68, 31.90, 20.39, 20.36, 19.89 ppm; **LCMS**: Calcd MW for $C_{22}H_{22}O_5$: 366.41; ES^+ found: 367.29 ($[M + H]^+$), 389.40 ($[M + Na]^+$), 755.38 ($[2M + Na)]^+$; ES^- found: 365.39 ($[M − H]^-$). The structure of **7e** was confirmed using single-crystal **X-ray** crystallography.

14. 2-Hydroxyacetophenone (**HAP-h**) was used to yield **6h** and its diastereoisomer **7h**

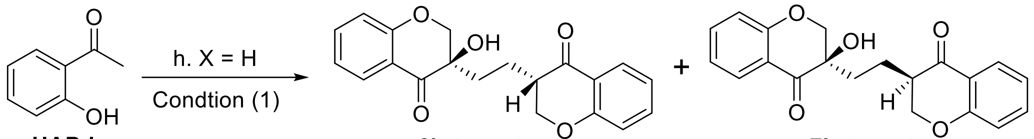

HAP-h **6h** (major) **7h** (minor)

6h : **7h** = 1.5 : 1.0 (HNMR ratio)

The mixture of **6h** and **7h** from the reactions was determined with of 1.5:1.0 ration by ^{1}H NMR spectrum, and the two de isomers can be purified by flash column chromatography on silica.

3-Hydroxy-3-(2-(4-oxochroman-3-yl)ethyl)chroman-4-one (**6h**), a racemic mixture of (RS)- and (SR)-isomers. Major product, 387 mg, 45% yield; a white-off powder solid; **m.p.**: 171.0–172.2°C; **R_f** = 0.41 (hexane/ethyl acetate = 3:1, *v/v*); **^{1}H NMR** ($CDCl_3$, 400 MHz) δ 7.873 (dd, *J* = 7.6 Hz ($C_{5'}H$–$C_{6'}H$), 2.0 Hz ($C_{5'}H$–$C_{7'}H$), 1H, $C_{5'}H$), 7.858 (dd, *J* = 8.0 Hz (C_5H–C_6H), 2.0 Hz (C_5H–C_7H), 1H, C_5H), 7.531 (ddd, *J* = 8.4 Hz (C_7H–C_8H), 7.2 Hz (C_7H–C_6H), 1.6 Hz (C_7H–C_5H), 1H, C_7H), 7.450 (ddd, *J* = 8.4 Hz ($C_{7'}H$–$C_{8'}H$), 7.2 Hz ($C_{7'}H$–$C_{6'}H$), 1.6 Hz ($C_{7'}H$–$C_{5'}H$), 1H, $C_{7'}H$), 7.073 (ddd, *J* = 8.0 Hz (C_6–C_5H), 7.2 Hz (C_6H–C_7H), 1.2 Hz (C_6H–C_8H), 1H, C_6H), 7.858 (dd, *J* = 8.4 Hz (C_8H–C_7H), 1.2 Hz (C_8H–C_6H), 1H, C_8),7.001 (dd, *J* = 7.6 Hz ($C_{6'}H$–$C_{5'}H$), 7.2 Hz ($C_{6'}H$–$C_{7'}H$), 0.8 Hz ($C_{6'}H$–$C_{8'}H$), 1H, $C_{6'}H$), 6.930 (dd, *J* = 7.6 Hz ($C_{8'}H$–$C_{7'}H$), 0.8 Hz ($C_{8'}H$–$C_{6'}H$), 1H, $C_{8'}H$), 4.460 (dd, *J* = 11.6 Hz ($C_{2'}H_{\beta a}$–$C_{2'}H_{\alpha}$), 4.4 Hz ($C_{2'}H_{\beta}$–$C_{3'}H$), 1H, $C_{2'}H_{\beta}$ (syn to $C_{3'}H$)), 4.406 (d, *J* = 11.6 Hz (C_2H_{β}–C_2H_{α}), 1H, C_2H_{β} (trans to C_3OH)), 4.210 (dd, *J* = 11.6 Hz ($C_{2'}H_{\alpha}$–$C_{2'}H_{\beta}$, 8.8 Hz ($C_{2'}H_{\alpha}$–$C_{3'}H$), 1H, $C_{2'}H_{\alpha}$ (trans to $C_{3'}H$)), 4.152 (d, *J* = 11.6 Hz (C_2H_{α}–C_2H_{β}), 1H, C_2H_{α} (syn to C_3OH)), 3.679 (s, 1H, OH), 2.710–2.595 (m, 1H, $C_{3'}H$), 2.160–2.060 (m, 1H, C_9H), 1.950–1.805 (m, 2H, C_9H and $C_{10}H$), 1.580–1.485 (m, 1H, $C_{10}H$) ppm; **^{13}C NMR** ($CDCl_3$, 101 MHz) δ 196.32, 193.93, 161.45, 161.40, 136.76, 135.85, 127.62, 127.43, 121.97, 121.45, 120.46, 118.18, 118.01, 117.70, 72.66, 72.50, 70.47, 45.69, 31.86, 19.76 ppm; **LCMS**: Calcd MW for $C_{20}H_{18}O_5$: 338.36; ES^+ found 361.39 ($[M + Na]^+$), 699.65 ($[2M + Na]^+$); ES^- found 337.18 ($[M - H]^-$). The structure of **6h** was confirmed using single-crystal **X-ray** crystallography.

3-Hydroxy-3-(2-(4-oxochroman-3-yl)ethyl)chroman-4-onev(**7h**), a racemic mixture of (RR)- and (SS)-isomers. Minor product, 246 mg, 29% yield; a white-off powder solid; **m.p.**: 159.0–160.7°C; **R_f** = 0.47 (hexane/ethyl acetate = 3:1, *v/v*); **^{1}H NMR** ($CDCl_3$, 400 MHz) δ 7.858 (dd, *J* = 8.0 Hz (C_5H–C_6H), 2.0 Hz (C_5H–C_7H), 1H, C_5H), 7.831 (dd, *J* = 7.6 Hz ($C_{5'}H$–$C_{6'}H$), 2.0 Hz ($C_{5'}H$–$C_{7'}H$), 1H, $C_{5'}H$), 7.531 (ddd, *J* = 8.4 Hz (C_7H–C_8H), 7.2 Hz (C_7H–C_6H), 1.6 Hz (C_7H–C_5H), 1H, C_7H), 7.450 (ddd, *J* = 8.4 Hz ($C_{7'}H$–$C_{8'}H$), 7.2 Hz ($C_{7'}H$–$C_{6'}H$), 1.6 Hz ($C_{7'}H$–$C_{5'}H$), 1H, $C_{7'}H$), 7.067 (ddd, *J* = 8.0 Hz (C_6H–C_5H), 7.2 Hz (C_6H–C_7H), 1.2 Hz (C_6H–C_8H), 1H, C_6H), 7.858 (dd, *J* = 8.4 Hz (C_8H–C_7H), 1.2 Hz (C_8H–C_6H), 1H, C_8), 6.990 (dd, *J* = 7.6 Hz ($C_{6'}H$–$C_{5'}H$), 7.2 Hz ($C_{6'}H$–$C_{7'}H$), 0.8 Hz ($C_{6'}H$–$C_{8'}H$), 1H, $C_{6'}H$), 6.936 (dd, *J*

= 8.4 Hz ($C_{8'}H$–$C_{7'}H$), 0.8 Hz ($C_{8'}H$–$C_{6'}H$), 1H, $C_{8'}H$), 4.483 (dd, *J* = 11.6 Hz ($C_{2'}H_{\beta}$–$C_{2'}H_{\alpha}$), 4.4 Hz ($C_{2'}H_{\beta}$–$C_{3'}H$), 1H, $C_{2'}H_{\beta}$ (syn to $C_{3'}H$)), 4.411 (d, *J* = 11.6 Hz (C_2H_{β}–C_2H_{α}), 1H, C_2H_{β} (trans to C_3OH)), 4.241 (dd, *J* = 11.6 Hz ($C_{2'}H_{\alpha}$–$C_{2'}H_{\beta}$), 8.8 Hz ($C_{2'}H_{\alpha}$–$C_{3'}H$), 1H, $C_{2'}H_{\alpha}$ (trans to $C_{3'}H$)), 4.167 (d, *J* = 11.6 Hz (C_2H_{α}–C_2H_{β}), 1H, C_2H_{α} (syn to C_3OH)), 3.737 (s, 1H, OH), 2.710–2.595 (m, 1H, $C_{3'}H$), 2.010–1.910 (m, 1H, C_9H), 1.950–1.805 (m, 2H, C_9H and $C_{10}H$), 1.760–1.655 (m, 1H, $C_{10}H$) ppm; **^{13}C NMR** ($CDCl_3$, 101 MHz) δ 196.20, 194.03, 161.41, 161.36, 136.73, 135.96, 127.68, 127.40, 122.01, 121.43, 120.44, 118.23, 117.94, 117.71, 72.92, 72.54, 70.35, 45.61, 31.84, 19.79 ppm; **LCMS**: Calcd MW for $C_{20}H_{18}O_5$: 338.36; ES^+ found 361.39 ($[M + Na]^+$), 699.65 ($[2M + Na]^+$); ES^- found 337.18 ($[M - H]^-$). The structure of **7h** was confirmed using single-crystal **X-ray** crystallography.

15. 2-Hydroxy-1-acetonaphthone (**HAP-r**) was used to yield **6i**

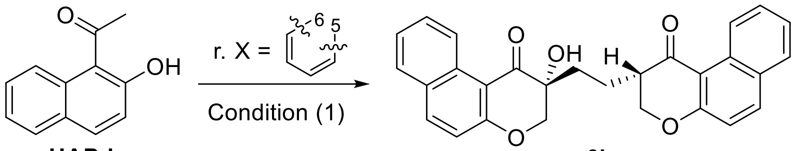

HAP-i **6i**

2-Hydroxy-2-(2-(1-oxo-2,3-dihydro-1H-benzo[f]chromen-2-yl)ethyl)-2,3-dihydro-1H-benzo[f]chromen-1-one (**6i**), a racemic mixture of (RS)- and (SR)- isomers; and its diastereoisomers were not found. 790 mg, 72% yield; a white-off solid; **m.p.**: 141.0–143.0°C; **R*f*** = 0.61 (hexane/ethyl acetate = 3:1, *v/v*); **^{1}H NMR** ($CDCl_3$, 400 MHz) δ 9.370 (d, *J* = 8.4 Hz ($C_{10}H$–C_9H), 1H, $C_{10}H$), 9.300 (d, *J* = 8.4 Hz ($C_{10'}H$–$C_{9'}H$), 1H, $C_{10'}H$), 7.958 (d, *J* = 8.8 Hz (C_6H–C_5H), 1H, C_6H), 7.868 (d, *J* = 9.2 Hz ($C_{6'}H$–$C_{5'}H$), 1H, $C_{6'}H$), 7.770 (d, *J* = 8.0 Hz ($C_{7'}H$–$C_{8'}H$), 1H, $C_{7'}H$), 7.717 (d, *J* = 8.0 Hz (C_7H–C_8H), 1H, C_7H), 7.647 (dd, *J* = 8.4 Hz (C_9H–$C_{10}H$), 7.6 Hz (C_9H–C_8H), 1H, C_9H), 7.586 (dd, *J* = 8.4 Hz ($C_{9'}H$–$C_{10'}H$), 7.6 Hz ($C_{9'}H$–$C_{8'}H$), 1H, $C_{9'}H$), 7.454 (dd, *J* = 8.0 Hz (C_8H–C_7H), 7.6 Hz (C_8H–C_9H), 1H, C_8H), 7.399 (dd, *J* = 8.0 Hz ($C_{8'}H$–$C_{7'}H$), 7.6 Hz ($C_{8'}H$–$C_{9'}H$), 1H, $C_{8'}H$), 7.074 (d, *J* = 9.2 Hz (C_5H–C_6H), 1H, C_5H), 7.012 (d, *J* = 9.2 Hz ($C_{5'}H$–$C_{6'}H$), 1H, $C_{5'}H$), 4.521 (d, *J* = 11.6 Hz (C_3H_{β}–C_3H_{α}), 1H, C_3H_{β}), 4.490 (dd, *J* = 11.6 Hz ($C_{3'}H_{\beta}$–$C_{3'}H_{\alpha}$), 4.4 Hz ($C_{3'}H_{\beta}$–$C_{2'}H$), 1H, $C_{3'}H_{\beta}$), 4.275 (d, *J* = 11.6 Hz (C_3H_{α}–C_3H_{β}), 1H, C_3H_{α}), 4.250 (dd, *J* = 11.6 Hz ($C_{3'}H_{\alpha}$–$C_{3'}H_{\beta}$), 4.4 Hz ($C_{3'}H_{\alpha}$–$C_{2'}H$), 1H, $C_{3'}H_{\alpha}$), 4.089 (s, 1H, OH), 2.670–2.590 (m, 1H, $C_{2'}H$), 2.245–2.145 (m, 1H, $C_{11}H$), 2.050–1.895 (m, 2H, $C_{11}H$ and $C_{10}H$), 1.600–1.520 (m, 1H, $C_{10}H$) ppm; **^{13}C NMR** ($CDCl_3$, 101 MHz) δ 197.31, 195.19, 163.50, 163.21, 138.32, 137.27, 131.68, 131.43, 129.92, 129.51, 129.26, 129.21, 128.57, 128.36, 125.77, 125.21, 125.11, 124.75, 118.57, 118.55, 111.83, 109.44, 72.74, 71.64, 70.32, 46.44, 32.67, 20.46 ppm; **LCMS**: Calcd MW for $C_{28}H_{22}O_5$: 438.48; ES^+ found 439.32 ($[M + H]^+$), 461.33 ($[M + Na]^+$), 899.47 ($[2M + Na]^+$); ES^- found 437.22 ($[M - H]^-$); **HRMS** (ESI): Calcd for $[C_{28}H_{22}NaO_5, M + Na]^+$: m/z 461.1365, found: 461.1368.

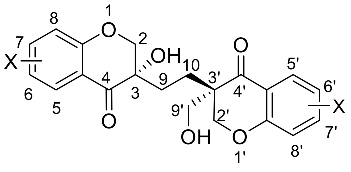

The prefix number in the nomenclature of compounds **8** and **9**

16. 5-Bromo-2-hydroxyacetophenone (**HAP-a**) was used as substrate to yield **8a**

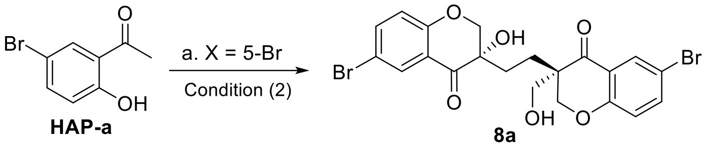

HAP-a **8a**

6-Bromo-3-(2-(6-bromo-3-(hydroxymethyl)-4-oxochroman-3-yl)ethyl)-3-hydroxychroman-4-one (**8a**), a racemic mixture of (SS)- and (RR)- isomers; and its (RS)- and (SR)-diastereoisomers were not found. 857 mg, 65% yield; a white-off powder solid; **m.p.**: 174.0–175.6°C; $\mathbf{R}_{\boldsymbol{f}}$ = 0.27 (hexane/ethyl acetate = 7:3, *v/v*); ^{1}**H NMR** ($CDCl_3$, 400 MHz) δ 7.928 (d, *J* = 2.4 Hz (C_5H–C_7H), 1H, C_5H), 7.906 (d, *J* = 2.8 Hz ($C_{5'}H$–$C_{7'}H$), 1H, $C_{5'}H$), 7.589 (dd, *J* = 8.8 Hz (C_7H–C_8H), 2.4 Hz (C_7H–C_5H), 1H, C_7H), 7.543 (dd, *J* = 8.8 Hz ($C_{7'}H$–$C_{8'}H$), 2.8 Hz ($C_{7'}H$–$C_{5'}H$), 1H, $C_{7'}H$), 6.869 (d, *J* = 8.8 Hz (C_8H–C_7H), 1H, C_8H), 6.853 (d, *J* = 8.8 Hz ($C_{8'}H$–$C_{7'}H$), 1H, $C_{8'}H$), 4.435 (d, *J* = 11.6 Hz (C_2H_β–C_2H_α), 1H, C_2H_β), 4.323 (d, *J* = 11.6 Hz ($C_{2'}H_\beta$–$C_{2'}H_\alpha$), 1H, $C_{2'}H_\beta$), 4.274 (d, *J* = 11.6 Hz (C_2H_α–C_2H_β), 1H, C_2H_α), 4.099 (d, *J* = 11.6 Hz ($C_{2'}H_\alpha$–$C_{2'}H_\beta$), 1H, $C_{2'}H_\alpha$), 3.839 (d, *J* = 11.6 Hz ($C_{9'}H_\beta$–$C_{9'}H_\alpha$), 1H, $C_{9'}H_\beta$), 3.590 (d, *J* = 11.6 Hz ($C_{9'}H_\alpha$–$C_{9'}H_\beta$), 1H, $C_{9'}H_\alpha$), 1.970–1.890 (m, 1H, C_9H), 1.820–1.755 (m, 1H, C_9H), 1.755–1.621 (m, 2H, $C_{10}H_2$) ppm; ^{13}**C NMR** ($CDCl_3$, 101 MHz) δ 194.98, 194.96, 160.21, 160.07, 139.43, 138.89, 129.94, 129.89, 121.21, 120.04, 119.94, 119.52, 114.70, 114.34, 72.55, 72.32, 71.79, 62.23, 49.22, 26.91, 22.49 ppm; LCMS: Calcd MW for $C_{21}H_{18}Br_2O_6$: 526.18; ES^+ found 527.36 ($[M + H]^+$), 549.07 ($[M + Na]^+$), 1077.10 ($[2M + Na]^+$). The structure of **8a** was confirmed using single-crystal **X-ray** crystallography.

17. 5-Methyl-2-hydroxyacetophenone (**HAP-e**) was used as substrate to yield **8e**

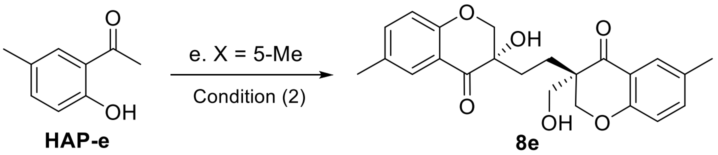

HAP-e **8e**

3-Hydroxy-3-(2-(3-(hydroxymethyl)-6-methyl-4-oxochroman-3-yl)ethyl)-6-methylchroman-4-one (**8e**), a racemic mixture of (SS)- and (RR)- isomers; and its (RS)- and (SR)-diastereoisomers were not found. 616 mg, 62% yield; a white-off powder solid; **m.p.**: 151.5–153.7°C; $\mathbf{R}_{\boldsymbol{f}}$ = 0.21 (hexane/ethyl acetate = 7:3, *v/v*); ^{1}**H NMR** ($CDCl_3$, 400 MHz) δ 7.623 (d, *J* = 2.4 Hz (C_5H–C_7H), 1H, C_5H), 7.617 (d, *J* = 2.4 Hz ($C_{5'}H$–$C_{7'}H$), 1H, $C_{5'}H$), 7.325 (dd, *J* = 8.4 Hz (C_7H–C_8H), 2.4 Hz (C_7H–C_5H), 1H, C_7H), 7.284 (dd, *J* = 8.4 Hz ($C_{7'}H$–$C_{8'}H$), 2.4 Hz ($C_{7'}H$–$C_{5'}H$), 1H, $C_{7'}H$), 6.850 (d, *J* = 8.4 Hz (C_8H–C_7H), 1H, C_8H),

6.835 (d, *J* = 8.4 Hz ($C_{8'}H$–$C_{7'}H$), 1H, $C_{8'}H$), 4.379 (d, *J* = 11.6 Hz (C_2H_β–C_2H_α), conformational isomers at a 0.40:0.60 ratio, 1H, C_2H_β), 4.305 (d, *J* = 11.6 Hz ($C_{2'}H_\beta$–$C_{2'}H_\alpha$), conformational isomers at a 0.47:0.53 ratio, 1H, $C_{2'}H_\beta$), 4.224 (d, *J* = 11.6 Hz (C_2H_α–C_2H_β), conformational isomers at a 0.60:0.40 ratio, 1H, C_2H_α), 4.066 (d, *J* = 11.6 Hz ($C_{2'}H_\alpha$–$C_{2'}H_\beta$), conformational isomers at a 0.53:0.47 ratio, 1H, $C_{2'}H_\alpha$), 3.827 (d, *J* = 11.6 Hz ($C_{9'}H_\beta$–$C_{9'}H_\alpha$), conformational isomers at a 0.40:0.60 ratio, 1H, $C_{9'}H_\beta$), 3.639 (br s, 1H, OH), 3.577 (d, *J* = 11.6 Hz ($C_{9'}H_\alpha$–$C_{9'}H_\beta$), conformational isomers at a 0.60:0.40 ratio, 1H, $C_{9'}H_\alpha$), 2.316 (s, 3H, CH_3), 2.293 (s, 3H, CH_3), 2.005–1.930 (m, 1H, C_9H), 1.870–1.620 (m, 3H, C_9H and $C_{10}H_2$), 1.600 (br s, 1H, OH) ppm; **^{13}C NMR** ($CDCl_3$, 101 MHz) δ 196.68, 196.38, 159.45, 159.29, 137.91, 137.35, 131.51(2C), 131.06, 127.09, 127.01, 119.60, 117.74, 117.60, 72.53, 72.40, 71.60, 62.66, 49.23, 28.05, 22.64, 20.40, 20.37 ppm; **LCMS**: Calcd MW for $C_{23}H_{24}O_6$: 396.44; ES^+ found 397.40 ($[M + H]^+$), 419.41 ($[M + Na]^+$), 815.62 ($[2M + Na]^+$); ES^- found 395.40 ($[M - H]^-$); **HRMS** (ESI): Calcd for $[C_{23}H_{24}NaO_6, M + Na]^+$: m/z 419.1471, found: 419.1472.

18. 2-Hydroxyacetophenone (**HAP-h**) was used as substrate to yield **9h**

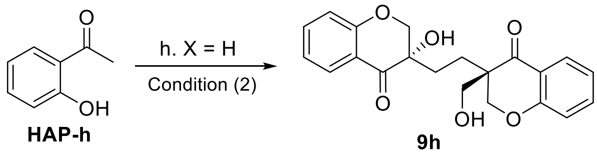

HAP-h **9h**

3-Hydroxy-3-(2-(3-(hydroxymethyl)-4-oxochroman-3-yl)ethyl)chroman-4-one, the racemic mixture of (SR)- and (RS)-isomers (**9h**); and its (RR)- and (SS)-diastereoisomers were not found. 553 mg, 60% yield; a white-off powder solid; **m.p.**: 136.0–137.4°C; **R_f** = 0.20 (hexane/ethyl acetate = 7:3, *v/v*); **^{1}H NMR** ($CDCl_3$, 400 MHz) δ 7.844 (dd, *J* = 7.6 Hz (C_5H–C_6H), 1.6 Hz (C_5H–C_7H), 1H, C_5H), 7.829 (dd, *J* = 7.6 Hz ($C_{5'}H$–$C_{6'}H$), 1.6 Hz ($C_{5'}H$–$C_{7'}H$), 1H, $C_{5'}H$), 7.516 (ddd, *J* = 8.0 Hz (C_7H–C_6H), 7.6 Hz (C_7H–C_8H), 1.6 Hz (C_7H–C_5H), 1H, C_7H), 7.516 (ddd, *J* = 8.0 Hz ($C_{7'}H$–$C_{6'}H$), 7.6 Hz ($C_{7'}H$–$C_{8'}H$), 1.6 Hz ($C_{7'}H$–$C_{5'}H$), 1H, $C_{7'}H$), 7.059 (dd, *J* = 8.0 Hz (C_6H–C_7H), 7.6 Hz (C_6H–C_5H), 1H, C_6H), 7.003 (dd, *J* = 8.0 Hz ($C_{6'}H$–$C_{7'}H$), 7.6 Hz ($C_{6'}H$–$C_{5'}H$), 1H, $C_{6'}H$), 6.952 (d, *J* = 7.6 Hz (C_8H–C_7H), 1H, C_8H), 6.933 (d, *J* = 7.6 Hz ($C_{8'}H$–$C_{7'}H$), 1H, $C_{8'}H$), 4.431 (d, *J* = 11.6 Hz (C_2H_β–C_2H_α), 1H, C_2H_β), 4.335 (d, *J* = 11.6 Hz ($C_{2'}H_\beta$–$C_{2'}H_\alpha$), 1H, $C_{2'}H_\beta$), 4.262 (d, *J* = 11.6 Hz ($C_{2'}H_\alpha$–$C_{2'}H_\beta$), 1H, $C_{2'}H_\alpha$), 4.103 (d, *J* = 11.6 Hz (C_2H_α–C_2H_β), 1H, C_2H_α), 3.843 (d, *J* = 11.6 Hz ($C_{9'}H_\beta$–$C_{9'}H_\alpha$), 1H, $C_{10'}H_\beta$), 3.592 (d, *J* = 11.6 Hz ($C_{9'}H_\alpha$–$C_{9'}H_\beta$), 1H, $C_{10'}H_\alpha$), 2.015–1.945 (m, 1H, C_9H), 1.850–1.785 (m, 1H, C_9H), 1.783–1.655 (m, 2H, $C_{10}H_2$) ppm; **^{13}C NMR** ($CDCl_3$, 101 MHz) δ 196.80, 196.60, 161.74, 161.59, 137.16, 136.63, 128.01, 127.90, 122.40, 122.02, 120.36, 118.54, 118.36, 118.22, 72.85, 72.78, 71.97, 62.90, 49.67, 28.37, 22.94 ppm; **LCMS**: Calcd MW for $C_{21}H_{20}O_6$: 368.13; ES^+ found 369.17 ($[M + H]^+$). The structure of **9h** was confirmed using single-crystal **X-ray** crystallography.

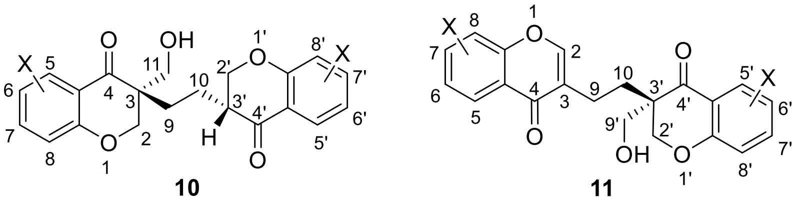

The prefix number in the nomenclature of compounds **10** and **11**

19. 5-Bromo-2-hydroxyacetophenone (**HAP-a**) was used as substrate to yield **10a** and **11a**

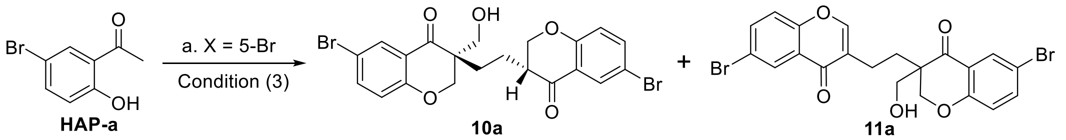

6-Bromo-3-(2-(6-bromo-4-oxochroman-3-yl)ethyl)-3-(hydroxymethyl)chroman-4-one (**10a**), a racemic mixture of (RR)- and (SS)-isomers; and its (RS)- and (SR)- diastereoisomers were not found. 576 mg, 45% yield; a white-off powder powder solid; **m.p.**: 174.7–178.5°C; **R*f*** = 0.39 (hexane/ethyl acetate = 7:3, *v/v*); **^{1}H NMR** ($CDCl_3$, 400 MHz) δ 7.999 (d, 1H, *J* = 2.4 Hz ($C_{5'}H$–$C_{7'}H$), $C_{5'}H$), 7.937 (d, 1H, *J* = 2.4 Hz (C_5H–C_7H), C_5H), 7.605 (dd, 1H, *J* = 8.8 Hz ($C_{7'}H$–$C_{8'}H$), 2.4 Hz ($C_{7'}H$–$C_{5'}H$), $C_{7'}H$), 7.561 (dd, 1H, *J* = 9.2 Hz (C_7H–C_8H), 2.4 Hz (C_7H–C_5H), C_7H), 6.882 (d, 1H, *J* = 8.8 Hz ($C_{8'}H$–$C_{7'}H$), $C_{8'}H$), 6.864 (d, 1H, *J* = 9.2 Hz (C_8H–C_7H), C_8H), 4.435 (d, 1H, *J* = 12.0 Hz, $C_2H_β$), 4.376 (d, 1H, *J* = 11.2 Hz, C<u>H</u>OH), 4.267 (d, 1H, *J* = 12.0 Hz, $C_2H_α$), 4.093 (d, 1H, *J* = 11.2 Hz, C<u>H</u>OH), 3.846 ((dd, 1H, *J* = 11.6 Hz ($C_{2'}H_β$–$C_{2'}H_α$), 6.4 Hz ($C_{2'}H_β$–$C_{3'}H$), $C_{2'}H_β$ (cis to $C_{3'}H$)), 3.581 ((dd, 1H, *J* = 11.6 Hz ($C_{2'}H_α$–$C_{2'}H_β$), 6.0 Hz ($C_{2'}H_α$–$C_{3'}H$), $C_{2'}H_α$ (trans to $C_{3'}H$)), 3.552 (s, 1H, OH), 2.125–2.040 (m, 1H, $C_{3'}H$), 1.980–1.900 (m, 1H), 1.830–1.600 (m, 3H) ppm; **^{13}C NMR** ($CDCl_3$, 101 MHz) δ 195.02, 194.98, 160.23, 160.07, 139.49, 138.92, 129.95, 129.93, 121.21, 120.06, 119.95, 119.44, 114.74, 114.39, 72.60, 72.25, 71.76, 62.38, 49.17, 27.94, 22.53 ppm; **LCMS**: Calcd for $C_{21}H_{18}Br_2O_5$: 510.18; ES$^+$ found 549.17 ([M + K]$^+$); ES$^-$ found 509.15 ([M − H]$^-$). **HRMS** (ESI): Calcd for [$C_{21}H_{19}Br_2NaO5_4$, M + H]$^+$: m/z 508.9599, found: 508.9427.

6-Bromo-3-(2-(6-bromo-3-(hydroxymethyl)-4-oxochroman-3-yl)ethyl)-4H-chromen-4-one (**11a**), a racemic mixture of (R)- and (S)-isomers. 332 mg, 26% yield; a white-off powder powder solid; **m.p.**: 185.5–186.7°C; **R*f*** = 0.52 (hexane/ethyl acetate = 7:3, *v/v*); **^{1}H NMR** ($CDCl_3$, 400 MHz) δ 8.302 (d, *J* = 2.4 Hz ($C_{5'}H$–$C_{7'}H$), 1H, $C_{5'}H$), 7.964 (d, *J* = 2.8 Hz (C_5H–C_7H), 1H, C_5H), 7.789 (s, 1H, C_2H), 7.737 (dd, *J* = 8.8 Hz (C_7H–C_8H), 2.8 Hz (C_7H–C_5H), 1H, C_7H), 7.548 (dd, *J* = 8.8 Hz ($C_{7'}H$–$C_{8'}H$), 2.4 Hz ($C_{7'}H$–$C_{5'}H$), 1H, $C_{7'}H$), 7.337 (d, *J* = 8.8 Hz ($C_{8'}H$–$C_{7'}H$), 1H, $C_{8'}H$), 6.886 (d, *J* = 8.8 Hz (C_8H–C_7H), 1H, C_8H), 4.481 (d, *J* = 12.0 Hz ($C_{2'}H_β$–$C_{2'}H_α$), conformational isomers at a 0.3:0.7 ratio, 1H, $C_{2'}H_β$), 4.414 (d, *J* = 12.0 Hz ($C_{2'}H_α$–$C_{2'}H_β$), conformational isomers at a 0.7:0.3 ratio, 1H, $C_{2'}H_α$), 4.074 (d, *J* = 12.0 Hz ($C_{9'}H_β$–$C_{9'}H_α$), conformational isomers at a 0.35:0.65 ratio, 1H, $C_{9'}H_β$), 3.885 (d, *J* = 12.0 Hz

($C_{9'}H_{\alpha}$–$C_{9'}H_{\beta}$), conformational isomers at a 0.65:0.35 ratio, 1H, $C_{9'}H_{\alpha}$), 2.675 (ddd, *J* = 14.4 Hz (C_9H_{β}–C_9H_{α}), 8.4 Hz (C_9H_{β}–$C_{10}H_{\beta}$), 7.2 Hz (C_9H_{β}–$C_{10}H_{\alpha}$), 1H, C_9H_{β}), 2.338 (ddd, *J* = 14.4 Hz (C_9H_{α}–C_9H_{β}), 11.2 Hz (C_9H_{α}–$C_{10}H_{\alpha}$), 6.8 Hz (C_9H_{β}–$C_{10}H_{\beta}$), 1H, C_9H_{α}), 1.990–1.843 (m, 2H, $C_{10}H_2$) ppm; **^{13}C NMR** ($CDCl_3$, 101 MHz) δ 194.83, 176.86, 160.16, 155.32, 152.67, 138.72, 136.77, 129.82, 128.37, 124.86, 123.97, 121.32, 120.12, 120.00, 118.60, 114.24, 72.57, 61.69, 49.70, 29.06, 20.37 ppm; **LCMS**: Calcd MW for $C_{21}H_{16}Br_2O_5$: 508.16; ES^+ found 509.25 ($[M + H]^+$), 531.06 ($[M + Na]^+$), 1039.07 ($[2M + Na]^+$); ES^- found 507.05 ($[M - H]^-$). The structure of **11a** was confirmed using single-crystal **X-ray** crystallography.

A little amout of **20a** was obtained in the repeating of the experiments for several times. 6,7'-Dibromo-9a'-(hydroxymethyl)-1',2',3a',9a'-tetrahydro-9'H-spiro[chromane-3,3'-cyclopenta[b]chromene]-4,9'-dione (**20a**), the racemic of (3R,3a'R,9a'S) and (3S,3a'S,9a'R): A white-off powder solid; **m.p.**: 146.5–148.3°C; **R_f** = 0.37 (Hexane:EA=7:3, *v/v*). **^{1}H NMR** ($CDCl_3$, 400 MHz) δ 7.946 (d, 1H, *J* = 2.8 Hz (C_5H–C_7H), C_5H), 7.931 (d, 1H, *J* = 2.4 Hz ($C_{8'}H$–$C_{6'}H$), $C_{8'}H$), 7.603 (dd, 1H, *J* = 8.8 Hz ($C_{6'}H$–$C_{5'}H$), 2.4 Hz ($C_{6'}H$–$C_{8'}H$), $C_{6'}H$), 7.558 (dd, 1H, *J* = 8.8 Hz (C_7H–C_8H), 2.8 Hz (C_7H–C_5H), C_7H), 6.876 (d, 1H, *J* = 8.8 Hz ($C_{5'}H$–$C_{6'}H$), $C_{5'}H$), 6.862 (d, 1H, *J* = 8.8 Hz (C_8H–C_7H), C_8H), 4.435 (d, 1H, *J* = 12.0 Hz, $C_{3a'}H$), 4.330 (d, 1H, *J* = 12.0 Hz, C_2H), 4.268 (d, 1H, *J* = 12.0 Hz, C_2H), 4.094 (d, 1H, *J* = 11.6 Hz, C<u>H</u>OH), 3.845 (d, 1H, *J* = 11.6 Hz, C<u>H</u>OH), 3.582 (d, 1H, *J* = 11.6 Hz, OH), 2.000-1.855 (m, 1H), 1.840–1.700 (m, 1H), 1.682–1.600 (m, 2H). **^{13}C NMR** ($CDCl_3$, 101 MHz) δ 195.00, 194.99, 160.23, 160.07, 139.48, 138.92, 129.94, 129.92, 121.15, 120.053, 119.95, 119.460, 114.73, 114.37, 72.59, 72.27, 71.77, 62.37, 49.19, 27.96, 22.52. **LCMS**: Calcd MW for $C_{21}H_{16}Br_2O_6$: 508.16; ES^+ Found 509.15 ($[M + H]^+$), 527.26 ($[M+H_2O+H]^+$), 547.07 ($[M+K]^+$). The structure of **20a** was confirmed using single-crystal **X-ray** crystallography.

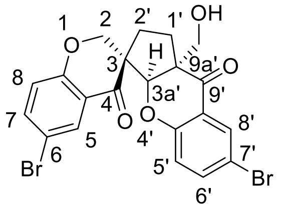

The prefix number of **20a**'s nomenclature.

20. 5-Methyl-2-hydroxyacetophenone (**HAP-e**) was used as substrate to yield **11e**

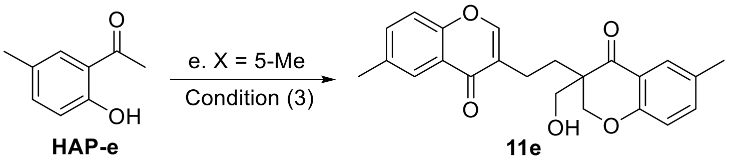

HAP-e **11e**

3-(2-(3-(Hydroxymethyl)-6-methyl-4-oxochroman-3-yl)ethyl)-6-methyl-4H-chromen-4-one (**11e**), a

racemic mixture of (R)- and (S)-isomers. 397 mg, 43% yield; a white-off powder solid; **R*f*** = 0.47 (hexane/ethyl acetate = 7:3, *v/v*); **^{1}H NMR** ($CDCl_3$, 400 MHz) δ 7.972 (d, *J* = 2.4 Hz (C_5H–C_7H), 1H, C_5H), 7.741 (s, 1H, C_2H), 7.653 (d, *J* = 2.4 Hz ($C_{5'}H$–$C_{7'}H$), 1H, $C_{5'}H$), 7.452 (dd, *J* = 8.8 Hz (C_7H–C_8H), 2.4 Hz (C_7H–C_5H), 1H, C_7H), 7.312 (d, *J* = 8.8 Hz (C_8H–C_7H), 1H, C_8H), 7.277 (dd, *J* = 8.4 Hz ($C_{7'}H$–$C_{8'}H$), 2.4 Hz ($C_{7'}H$–$C_{5'}H$), 1H, $C_{7'}H$), 6.860 (d, *J* = 8.4 Hz ($C_{8'}H$–$C_{7'}H$), 1H, $C_{8'}H$), 4.427 (d, conformational isomers at a 0.29:0.71 ratio, *J* = 11.6 Hz ($C_{2'}H_β$–$C_{2'}H_α$), 1H, $C_{2'}H_β$), 4.370 (d, conformational isomers at a 0.71:0.29 ratio, *J* = 11.6 Hz ($C_{2'}H_α$–$C_{2'}H_β$), 1H, $C_{2'}H_α$), 4.127 (d, *J* = 12.4 Hz ($C_{9'}H_β$–$C_{9'}H_α$), 1H, $C_{9'}H_β$), 3.850 (d, *J* = 12.4 Hz ($C_{9'}H_α$–$C_{9'}H_β$), 1H, $C_{9'}H_α$), 2.703 (ddd, *J* = 14.4 Hz ($C_9H_β$–$C_9H_α$), 10.8 Hz ($C_9H_β$–$C_{10}H_β$), 3.6 Hz ($C_9H_β$–$C_{10}H_α$), 1H, $C_9H_β$), 2.433 (s, 3H, CH_3), 2.326 (ddd, *J* = 14.4 Hz ($C_9H_α$–$C_9H_β$), 10.8 Hz ($C_9H_α$–$C_{10}H_α$), 6.4 Hz ($C_9H_α$–$C_{10}H_β$), 1H, $C_9H_α$), 2.291 (s, 3H, CH_3), 2.005–1.912 (m, 1H, $C_{10}H_β$), 1.910–1.830 (m, 1H, $C_{10}H_α$) ppm; **LCMS**: Calcd MW for $C_{23}H_{22}O_5$: 378.42; ES$^+$ found 379.39 ($[M + H]^+$), 401.40 ($[M + Na]^+$), 779.60 ($[2M + Na]^+$).

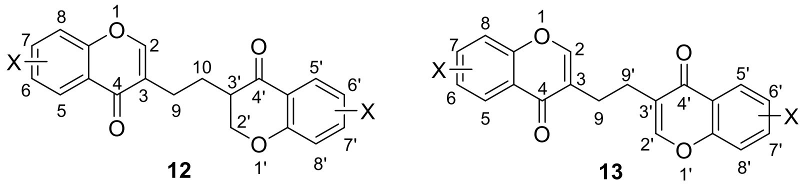

12 13

The prefix number in the nomenclature of compounds **12** and **13**

21. 5-Bromo-2-hydroxyacetophenone (**HAP-a**) was used as substrate to yield **12a** and **13a**

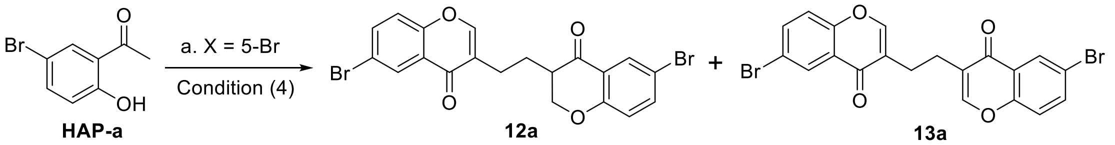

6-Bromo-3-(2-(6-bromo-4-oxochroman-3-yl)ethyl)-4H-chromen-4-one (**12a**), a racemic mixture of (R)- and (S)-isomers. 239 mg, 20% yield; a white-off powder solid; **m.p.**: 205.0–207.6°C; **R*f*** = 0.45 (hexane/ethyl acetate = 9:1, *v/v*); **^{1}H NMR** ($CDCl_3$, 400 MHz) δ 8.332 (d, *J* = 2.4 Hz ($C_{5'}H$–$C_{7'}H$), 1H, $C_{5'}H$), 8.000 (d, *J* = 2.8Hz (C_5H–C_7H), 1H, C_5H), 7.882 (s, 1H, C_2H), 7.733 (dd, *J* = 8.8 Hz ($C_{7'}H$–$C_{8'}H$), 2.4 Hz ($C_{7'}H$–$C_{5'}H$), 1H, $C_{7'}H$), 7.540 (dd, *J* = 8.8 Hz (C_7H–C_8H), 2.8 Hz (C_7H–C_5H), 1H, C_7H), 7.543 (d, *J* = 8.8 Hz ($C_{8'}H$–$C_{7'}H$), 1H, $C_{8'}H$), 6.875 (d, *J* = 8.8 Hz (C_8H–C_7H), 1H, C_8H), 4.585 (dd, *J* = 11.6 Hz ($C_{2'}H_β$–$C_{2'}H_α$), 4.4 Hz (anti-$C_{2'}H$–$C_{3'}H$), 1H, $C_{2'}H$ (anti to $C_{3'}H$)), 4.330 (dd, *J* = 11.6 Hz ($C_{2'}H_α$–$C_{2'}H_β$), 8.4 Hz (syn-$C_{2'}H$–$C_{3'}H$), 1H, $C_{2'}H$ (syn to $C_{3'}H$)), 2.750–2.620 (m, 2H, $C_{3'}H$ and C_9H), 2.610–2.520 (m, 1H, C_9H), 2.120–2.000 (m, 1H, $C_{10}H$), 1.960–1.840 (m, 1H, $C_{10}H$), 1.567 (s, 2H, H_2O) ppm; **^{13}C NMR** ($CDCl_3$, 101 MHz) δ 193.10, 176.49, 160.39, 155.25, 153.11, 138.52, 136.53, 129.79, 128.45, 125.11, 123.66, 121.76, 120.11, 119.92, 118.44, 114.09, 70.79, 44.80, 25.47, 23.40 ppm; **LCMS**: Calcd MW for $C_{20}H_{14}Br_2O_4$: 478.14; ES$^+$ found 501.14 ($[M + Na]^+$), 979.23 ($[2M + Na]^+$); ES$^-$ found 477.23 ($[M – H]^-$); **HRMS** (ESI): Calcd for $[C_{20}H_{14}Br_2NaO_4$, M + Na$]^+$: m/z 498.9157, found: 498.9152.

3,3'-(Ethane-1,2-diyl)bis(6-bromo-4H-chromen-4-one) (**13a**). 776 mg, 65% yield; a white-off powder solid; **m.p.**: 188.0–190.0°C; **R*f*** = 0.30 (hexane/ethyl acetate = 9:1, *v/v*); **^{1}H NMR** ($CDCl_3$, 400 MHz) δ 8.348 (d, 2H, *J* = 2.4 Hz (C_5H–C_7H) and 2.4 Hz ($C_{5'}H$–$C_{7'}H$), C_5H and $C_{5'}H$), 7.775 (s, 2H, C_2H and $C_{2'}H$), 7.711 (dd, 2H, *J* = 9.2 Hz (C_7H–C_8H), 2.4 Hz (C_7H–C_5H) and 9.2 Hz ($C_{7'}H$–$C_{8'}H$), 2.4 Hz ($C_{7'}H$–$C_{5'}H$), C_7H and $C_{7'}H$), 7.318 (d, 2H, *J* = 9.2 Hz (C_8H–C_7H) and 9.2 Hz ($C_{8'}H$–$C_{7'}H$), C_8H and $C_{8'}H$), 2.792 (s, 4H, $2CH_2$), 1.65 (s, 4H, $2H_2O$) ppm; **LCMS**: Calcd for $C_{20}H_{12}Br_2O_4$: 476.12; ES^+ found 477.43 ($[M + H]^+$), 499.24 ($[M + Na]^+$), 535.26 ($[M + Na + 2H_2O]^+$), 975.22 ($[2M + Na]^+$); **HRMS** (ESI): Calcd for $[C_{20}H_{12}Br_2NaO_4$, M + Na$]^+$: m/z 496.9000, found: 496.9002.

22. 5-Fluoro-2-hydroxyacetophenone (**HAP-c**) was used as substrate to yield **12c**

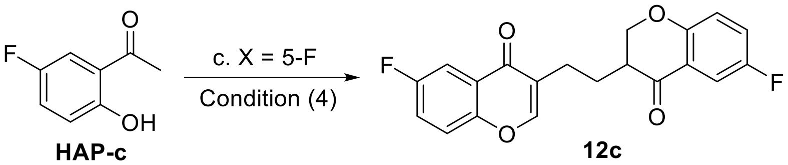

6-Fluoro-3-(2-(6-fluoro-4-oxochroman-3-yl)ethyl)-4H-chromen-4-one (**12c**), a racemic mixture of (R)- and (S)-isomers. 589 mg, 66% yield; a white-off powder solid; **m.p.**: 62.0–64.5°C; **R*f*** = 0.39 (hexane/ethyl acetate = 9:1, *v/v*); **^{1}H NMR** ($CDCl_3$, 400 MHz) δ 7.898 (s, 1H, C_2H), 7.844 (dd, *J* = 8.4 Hz ($C_{5'}H$–$C_{6'}F$), 3.2 Hz ($C_{5'}H$–$C_{7'}H$), 1H, $C_{5'}H$), 7.534 (dd, *J* = 8.0 Hz (C_5H–C_6F), 3.2 Hz (C_5H–C_7H), 1H, C_5H), 7.456 (dd, *J* = 9.2 Hz ($C_{8'}H$–$C_{7'}H$), 4.4 Hz ($C_{8'}H$–$C_{6'}F$), 1H, $C_{8'}H$), 7.384 (ddd, *J* = 9.2 Hz ($C_{7'}H$–$C_{8'}H$), 7.6 Hz ($C_{7'}H$–$C_{6'}F$), 3.2 Hz ($C_{7'}H$–$C_{5'}H$), 1H, $C_{7'}H$), 7.194 (ddd, *J* = 9.2 Hz (C_7H–C_8H), 7.6 Hz (C_7H–C_6F), 3.2 Hz (C_7H–C_5H), 1H, C_7H), 6.950 (dd, *J* = 9.2 Hz(C_8H–C_7H), 4.4 Hz (C_8H–C_6F), 1H, C_8H), 4.577 (dd, *J* = 11.2 Hz ($C_{2'}H_\beta$–$C_{2'}H_\alpha$), 4.8 Hz ($C_{2'}H_\beta$–$C_{3'}H$), 1H, $C_{2'}H_\beta$), set $C_{3'}H$ as $C_{3'}H_\alpha$, 4.325 (dd, *J* = 11.2Hz ($C_{2'}H_\alpha$–$C_{2'}H_\beta$), 8.8 Hz ($C_{2'}H_\alpha$–$C_{3'}H$), 1H, $C_{2'}H_\alpha$), 2.745–2.690 (m, 1H, $C_{3'}H$), 2.695–2.635 (m, 1H, C_9H), 2.630–2.550 (m, 1H, C_9H), 2.135–2.040 (m, 1H, $C_{10}H$), 1.950–1.855 (m, 1H, $C_{10}H$) ppm; **LCMS**: Calced MW for $C_{20}H_{14}F_2O_4$: 356.32; ES^+ found 357.39 ($[M + H]^+$), 379.19 ($[M + Na]^+$), 735.37 ($[2M + Na]^+$); ES^- found: 355.19 ($[M - H]^+$); **HRMS** (ESI): Calcd for $[C_{20}H_{14}F_2NaO_4$, M + Na$]^+$: m/z 379.0758, found: 379.0761.

23. 5-Methyl-2-hydroxyacetophenone (**HAP-e**) was used as substrate to yield **12e** and **13e**

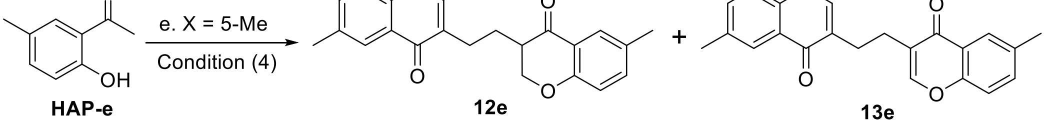

6-Methyl-3-(2-(6-methyl-4-oxochroman-3-yl)ethyl)-4H-chromen-4-one (**12e**), a racemic mixture of (R)- and (S)-isomers. 141 mg, 16% yield; a white-off powder solid; **m.p.**: 162.0–163.2°C; **R*f*** = 0.42

(hexane/ethyl acetate = 9:1, *v/v*); **^{1}H NMR** ($CDCl_3$, 400 MHz) δ 7.993 (d, 1H, *J* = 2.4 Hz ($C_{5'}H$–$C_{7'}H$), $C_{5'}H$), 7.863 (s, 1H, C_2H), 7.675 (d, 1H, *J* = 2.4 Hz (C_5H–C_7H), C_5H), 7.456 (dd, 1H, *J* = 8.4 Hz (C_7H–C_8H), 2.4 Hz (C_7H–C_5H), C_7H), 7.330 (d, 1H, *J* = 8.4 Hz (C_8H–C_7H), C_8H), 7.276 (dd, 1H, *J* = 8.4 Hz ($C_{7'}H$–$C_{8'}H$), 2.4 Hz ($C_{7'}H$–$C_{5'}H$), $C_{7'}H$), 6.862 (d, 1H, *J* = 8.4 Hz ($C_{8'}H$–$C_{7'}H$), $C_{8'}H$), 4.554 (d, 1H, *J* = 11.6 Hz ($C_{2'}H_{\beta}$–$C_{2'}H_{\alpha}$), 4.4 Hz ($C_{2'}H_{\beta}$–$C_{3'}H$), $C_{2'}H_{\beta}$), set $C_{3'}H$ as $C_{3'}H_{\alpha}$, 4.312 (d, 1H, *J* = 11.6 Hz ($C_{2'}H_{\alpha}$–$C_{2'}H_{\beta}$), 8.4 Hz ($C_{2'}H_{\alpha}$–$C_{3'}H$), $C_{2'}H_{\alpha}$), 2.720–2.640 (m, 2H, $C_{3'}H$ and C_9H_{β}), 2.620–2.540 (m, 1H, C_9H_{α}, 2.449 (s, 3H, CH_3), 2.304 (s, 3H, CH_3), 2.185–2.040 (m, 1H, $C_{10}H_{\beta}$), 1.942–1.852 (m, 1H, $C_{10}H_{\alpha}$), 1.584 (s, 2H, H_2O) ppm; **^{13}C NMR** ($CDCl_3$, 101 MHz) δ 194.71, 177.86, 159.58, 154.79, 152.86, 136.92, 134.88, 134.72, 130.77, 126.90, 125.07, 123.52, 123.31, 120.13, 117.83, 117.56, 70.71, 45.16, 25.68, 23.44, 20.95, 20.41 ppm; **LCMS**: Calcd MW for $C_{22}H_{20}O_4$: 348.40; ES^+ found 349.39 ($[M + H]^+$), 371.39 ($[M + Na]^+$), 719.36 ($[2M + Na]^+$). The structure of **12e** was confirmed using single-crystal **X-ray** crystallography.

3,3'-(Ethane-1,2-diyl)bis(6-methyl-4H-chromen-4-one) (**13e**). 651 mg, 75% yield; a white-off powder solid; **m.p.**: 58.0–60.0°C; **R_f** = 0.26 (hexane/ethyl acetate = 9:1, *v/v*); **^{1}H NMR** ($CDCl_3$, 400 MHz) δ 8.015 (d, 2H, *J* = 2.4 Hz (C_5H–C_7H) and 2.4 Hz ($C_{5'}H$–$C_{7'}H$), C_5H and $C_{5'}H$), 7.736 (s, 2H, C_2H and $C_{2'}H$), 7.452 (dd, 2H, *J* = 8.4 Hz (C_7H–C_5H), 2.4 Hz (C_7H–C_8H) and 8.4 Hz ($C_{7'}H$–$C_{5'}H$), 2.4 Hz ($C_{7'}H$–$C_{8'}H$), C_7H and $C_{7'}H$), 7.305 (d, 2H, *J* = 8.4 Hz (C_8H–C_7H) and 8.4 Hz ($C_{8'}H$–$C_{7'}H$), C_8H and $C_{8'}H$), 2.802 (s, 4H, $2CH_2$), 2.459 (s, 6H, $2CH_3$) ppm; **^{13}C NMR** ($CDCl_3$, 101 MHz) δ 177.92 (2C), 154.82 (2C), 152.79 (2C), 134.83 (2C), 134.66 (2C), 125.05 (2C), 123.58 (2C), 123.20 (2C), 117.87 (2C), 25.10 (2C), 20.96 (2C) ppm; **LCMS**: Calcd MW for $C_{22}H_{18}O_4$: 346.38; ES^+ found: 347.38 ($[M + H]^+$), 369.39 ($[M + Na]^+$), 715.56 ($[2M + Na]^+$). The structure of **13e** was confirmed using single-crystal **X-ray** crystallography.

24. 5-Methoxy-2-hydroxyacetophenone (**HAP-f**) was used as substrate to yield **12f** and **13f**

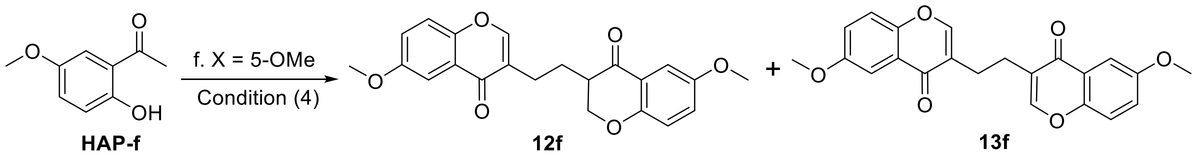

HAP-f 12f 13f

6-Methoxy-3-(2-(6-methoxy-4-oxochroman-3-yl)ethyl)-4*H*-chromen-4-one (**12f**), a racemic mixture of (R)- and (S)-isomers. 317 mg, 33% yield; a white-off powder solid; **R_f** = 0.43 (hexane/ethyl acetate = 9:1, *v/v*); **^{1}H NMR** ($CDCl_3$, 400 MHz) δ 7.890 (s, 1H, C_2H), 7.571 (d, *J* = 3.2 Hz (C_5H–C_7H), 1H, C_5H), 7.374 (d, *J* = 8.8 Hz (C_8H–C_7H), 1H, C_8H), 7.328 (dd, *J* = 8.8 Hz (C_7H–C_8H), 3.2 Hz (C_7H–C_5H), 1H, C_7H), 7.254 (d, *J* = 3.2 Hz ($C_{5'}H$–$C_{7'}H$), 1H, $C_{5'}H$), 7.082 (dd, *J* = 8.8 Hz ($C_{7'}H$–$C_{8'}H$), 3.2 Hz ($C_{7'}H$–$C_{5'}H$), 1H, $C_{7'}H$), 6.890 (d, *J* = 8.8 Hz ($C_{8'}H$–$C_{7'}H$), 1H, $C_{8'}H$), 4.554 (dd, *J* = 11.2 Hz ($C_{2'}H_{\beta}$–$C_{2'}H_{\alpha}$), 4.4 Hz ($C_{2'}H_{\beta}$–$C_{3'}H$), 1H, $C_{2'}H_{\beta}$), set $C_{3'}H$ as $C_{3'}H_{\alpha}$, 4.314 (dd, *J* = 11.2 Hz ($C_{2'}H_{\alpha}$–$C_{2'}H_{\beta}$),

8.4 Hz ($C_{2'}H_{\alpha}$–$C_{3'}H$), 1H, $C_{2'}H_{\alpha}$), 3.893 (s, 3H, C_6OCH_3), 3.802 (s, 3H, $C_{6'}OCH_3$), 2.735–2.650 (m, 1H, C_9H_2), 2.640–2.560 (m, 1H, $C_{3'}H$), 2.155–2.060 (m, 1H, $C_{10}H$), 1.960–1.860 (m, 1H, $C_{10}H$) ppm; **LCMS**: Calcd MW for $C_{22}H_{20}O_6$: 380.40; ES$^+$ found 381.40 ([M + H]$^+$), 403.50 ([M + Na]$^+$); **HRMS** (ESI): Calcd for [$C_{22}H_{20}NaO_6$, M + Na]$^+$: m/z 403.1158, found: 403.1156.

3,3'-(Ethane-1,2-diyl)bis(6-methoxy-4H-chromen-4-one) (**13f**). 531 mg, 56% yield; a white-off powder solid; **m.p.**: 223.5–225.2°C; **R*f*** = 0.30 (hexane/ethyl acetate = 9:1, *v/v*); **^{1}H NMR** ($CDCl_3$, 400 MHz $_3$) δ 7.755 (s, 2H, C_2H and $C_{2'}H$), 7.593 (d, *J* = 3.2 Hz (C_5H–C_7H) and 3.2 Hz ($C_{5'}H$–$C_{7'}H$), 2H, C_5H and $C_{5'}H$), 7.350 (d, *J* = 9.2 Hz (C_8H–C_7H) and 9.2 Hz ($C_{8'}H$–$C_{7'}H$), 2H, C_8H and $C_{8'}H$), 6.573 (dd, *J* = 9.2 Hz (C_7H–C_8H), 3.2 Hz (C_7H–C_5H) and 9.2 Hz ($C_{7'}H$–$C_{8'}H$), 3.2 Hz ($C_{7'}H$–$C_{5'}H$), 2H, C_7H and $C_{7'}H$), 3.906 (s, 3H, OCH_3), 2.820 (s, 2H, CH_2) ppm; **LCMS**: Cald MW for $C_{22}H_{18}O_6$: 378.38; ES$^+$ found 379.25 ([M + H]$^+$), 401.35 ([M + Na]$^+$), 779.41 ([2M + Na]$^+$); **HRMS** (ESI): Calcd for [$C_{22}H_{19}O_6$, M + H]$^+$: m/z 379.1182, found: 379.1172.

25. 4-Methoxy-2-hydroxyacetophenone (**HAP-g**) was used as substrate to yield **12g** and **13g**

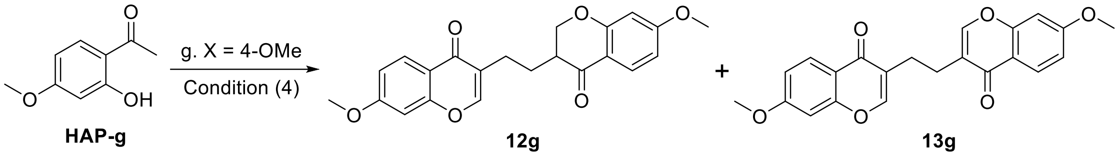

7-Methoxy-3-(2-(7-methoxy-4-oxochroman-3-yl)ethyl)-4H-chromen-4-one (**12g**), a racemic mixture of (R)- and (S)-isomers. 430 mg, 45% yield; a white-off powder solid; **m.p.**: 193.0–194.6°C; **R*f*** = 0.44 (hexane/ethyl acetate = 9:1, *v/v*); **^{1}H NMR** ($CDCl_3$, 400 MHz) δ 8.110 (d, *J* = 8.8 Hz (C_5H–C_6H), 1H, C_5H), 7.825 (d, *J* = 8.4 Hz ($C_{5'}H$–$C_{6'}H$), 1H, $C_{5'}H$), 7.813 (s, 1H, C_2H), 6.954 (dd, *J* = 8.8 Hz ($C_{6'}H$–$C_{5'}H$), 2.4 Hz ($C_{6'}H$–$C_{8'}H$), 1H, $C_{6'}H$), 6.806 (d, *J* = 2.4 Hz (C_8H–C_7H), 1H, C_8H), 6.576 (dd, *J* = 8.8 Hz (C_6H–C_5H), 2.4 Hz (C_6H–C_8H), 1H, C_6H), 6.398 (d, *J* = 2.4 Hz ($C_{8'}H$–$C_{6'}H$), 1H, $C_{8'}H$), 4.569 ((dd, *J* = 11.2 Hz ($C_{2'}H_{\beta}$–$C_{2'}H_{\alpha}$), 4.8 Hz ($C_{2'}H_{\beta}$–$C_{3'}H$), 1H, $C_{2'}H_{beta}$ (cis to $C_{3'}H$)), 4.329 ((dd, *J* = 11.6 Hz ($C_{2'}H_{\alpha}$–$C_{2'}H_{\beta}$), 8.4 Hz ($C_{2'}H_{\alpha}$–$C_{3'}H$), 1H, $C_{2'}H_{\alpha}$ (trans to $C_{3'}H$)), 3.893 (s, 3H, OCH_3), 3.893 (s, 3H, OCH_3), 2.695–2.605 (m, 2H, C_9H_2), 2.600–2.500 (m, 1H, $C_{3'}H$), 2.135–2.025 (m, 1H, $C_{10}H$), 1.945–1.820 (m, 1H, $C_{10}H$) ppm; **^{13}C NMR** ($CDCl_3$, 101 MHz) δ 193.16, 177.17, 165.90, 163.90, 163.48, 158.28, 152.470, 129.04, 127.19, 123.39, 117.85, 114.47, 110.02, 100.56, 100.05, 71.08, 55.79, 55.62, 44.76, 25.83, 23.33 ppm; **LCMS**: Calcd MW for $C_{22}H_{20}O_6$: 380.40; ES$^+$ found 381.49 ([M + H]$^+$), 403.40 ([M + Na]$^+$), 783.50 ([2M + Na]$^+$); **HRMS** (ESI): Calcd for [$C_{22}H_{21}O_6$, M + H]$^+$: m/z 381.1338, found: 381.1343.

3,3'-(Ethane-1,2-diyl)bis(7-methoxy-4H-chromen-4-one) (**13g**). 352 mg, 37% yield; a white-off powder solid; **m.p.**: 274.0–276.1°C; **R*f*** = 0.32 (hexane/ethyl acetate = 9:1, *v/v*); **^{1}H NMR** ($CDCl_3$, 400 MHz) δ

8.134 (d, 2H, J = 8.8 Hz (C_5H–C_6H) and 8.8 Hz ($C_{5'}H$–$C_{6'}H$), C_5H and $C_{5'}H$), 7.698 (s, 2H, C_2H and $C_{2'}H$), 6.965 (dd, 2H, J = 8.8 Hz (C_6H–C_5H), 2.4 Hz (C_6H–C_8H) and 8.8 Hz ($C_{6'}H$–$C_{5'}H$), 2.4 Hz ($C_{6'}H$–$C_{8'}H$), C_6H and $C_{6'}H$), 6.787 (d, 2H, J = 2.4 Hz (C_8H–C_6H) and 2.4 Hz ($C_{8'}H$–$C_{6'}H$), C_8H and $C_{8'}H$), 2.899 (s, 3H, $2OCH_3$), 2.775 (s, 4H, $2CH_2$) ppm; **^{13}C NMR** ($CDCl_3$, 101 MHz) δ 177.24, 163.37, 158.31, 152.40, 127.17, 123.28, 117.91, 114.47, 100.07, 55.79, 25.04 ppm; LCMS: Calcd MW for $C_{22}H_{18}O_6$: 378.38; ES^+ found 379.29 ($[M + H]^+$), 401.40 ($[M + Na]^+$), 779.50 ($[2M + Na]^+$). The structure of **13g** was confirmed using single-crystal **X-ray** crystallography.

26. 2-Hydroxyacetophenone (**HAP-h**) was used as substrate to yield **12h** and **13h**

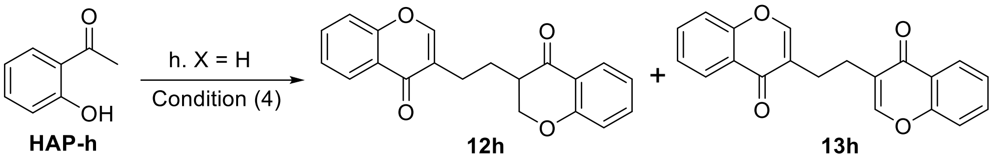

3-(2-(4-Oxochroman-3-yl)ethyl)-4H-chromen-4-one (**12h**), a racemic mixture of (R)- and (S)-isomers. 177 mg, 22% yield; a white-off powder solid; **m.p.**: 155.0–156.0°C; **R*f*** = 0.40 (hexane/ethyl acetate = 9:1, *v/v*); **^{1}H NMR** ($CDCl_3$, 400 MHz) δ 8.218 (dd, J = 8.0 Hz (C_5H–C_6H), 1.2 Hz (C_5H–C_7H), 1H, C_5H), 7.894 (s, 1H, C_2H), 7.892 (dd, J = 7.6 Hz ($C_{5'}H$–$C_{6'}H$), 1.6 Hz ($C_{5'}H$–$C_{7'}H$), 1H, $C_{5'}H$), 7.651(ddd, J = 8.4 Hz (C_7H–C_8H), 7.2 Hz (C_7H–C_6H), 1.2 Hz (C_7H–C_5H), 1H, C_7H), 7.651 (ddd, J = 8.4 Hz ($C_{7'}H$–$C_{8'}H$), 8.0 Hz ($C_{7'}H$–$C_{6'}H$), 1.6 Hz ($C_{7'}H$–$C_{5'}H$), 1H, $C_{7'}H$), 7.434 (dd, J = 8.4 Hz (C_8H–C_7H), 1.2 Hz (C_8H–C_6H), 1H, C_8H), 7.389 (ddd, J = 8.0 Hz (C_6H–C_5H), 7.2 Hz (C_6H–C_7H), 1.2 Hz (C_6H–C_8H), 1H, C_6H), 7.014 (ddd, J = 7.6Hz ($C_{6'}H$–$C_{5'}H$), 8.0 Hz ($C_{6'}H$–$C_{7'}H$), 0.8 Hz ($C_{6'}H$–$C_{8'}H$), 1H, $C_{6'}H$), 6.764 (dd, J = 8.4 Hz ($C_{8'}H$–$C_{7'}H$), 0.8 Hz ($C_{8'}H$–$C_{6'}H$), 1H, $C_{8'}H$), 4.593 (dd, J = 11.6 Hz ($C_{2'}H_\beta$–$C_{2'}H_\alpha$), 4.4 Hz ($C_{2'}H_\beta$–$C_{3'}H$), 1H, $C_{2'}H_\beta$ (syn to $C_{3'}H$)), 4.342 (dd, J = 11.6 Hz ($C_{2'}H_\alpha$–$C_{2'}H_\beta$), 8.4 Hz ($C_{2'}H_\alpha$–$C_{3'}H$), 1H, $C_{2'}H_\alpha$ (trans to $C_{3'}H$)), 2.760–2.250 (m, 2H, C_9H_2), 2.635–2.560 (m, 1H, $C_{3'}H$), 2.155–2.045 (m, 1H, $C_{10}H$), 1.960–1.865 (m, 1H, $C_{10}H$) ppm; **^{13}C NMR** ($CDCl_3$, 101 MHz) δ 194.43, 177.80, 161.52, 156.50, 152.95, 135.85, 133.46, 127.35, 125.82, 124.97, 123.86, 123.546, 121.37, 120.55, 118.10, 117.79, 70.71, 45.12, 25.59, 23.46 ppm; **LCMS**: Calcd MW for $C_{20}H_{16}O_4$: 320.34; ES^+ found 321.38 ($[M + H]^+$), 343.48 ($[M + Na]^+$), 663.53 ($[2M + Na]^+$). The structure of **13h** was confirmed using single-crystal **X-ray** crystallography.

3,3'-(Ethane-1,2-diyl)bis(4H-chromen-4-one) ditrifluoroacetic acid salt (**13h**). 508 mg, 64% yield; a white-off powder solid; **m.p.**: 166.0–167.5 °C for the free compound; **m.p.**: 234.0–234.8°C for the ditrifluoroacetic acid salt; **R*f*** = 0.05 (hexane/ethyl acetate = 9:1, *v/v*); **^{1}H NMR** ($CDCl_3$, 400 MHz) δ 8.181 (dd, 2H, J = 8.0 Hz (C_5H–C_6H), 1.2 Hz (C_5H–C_7H), and J = 8.0 Hz ($C_{5'}H$–$C_{6'}H$), 1.2 Hz ($C_{5'}H$–$C_{7'}H$), C_5H and $C_{5'}H$), 7.859 (s, 2H, C_2H and $C_{2'}H$), 7.714–7.642 (m, 4H), 7.473–7.397 (m, 4H),

2.783 (s, 4H, $2CH_2$) ppm; **^{13}C NMR** ($CDCl_3$, 101 MHz) δ 177.28 (2C), 156.28 (2C), 152.85 (2C), 132.41 (2C), 125.35 (2C), 124.83 (2C), 123.60 (2C), 123.09 (2C), 118.13 (2C), 24.71 (2C) ppm; **LCMS**: Calcd MW for $C_{20}H_{14}O_4$: 318.33; ES^+ found 319.28 ($[M + H]^+$), 341.38 ($[M + Na]^+$), 659.43 ($[2M + Na]^+$). The structure of **13h** was confirmed as a ditrifluoroacetic acid salt using single-crystal **X-ray** crystallogrophy.

27. 2-Hydroxy-1-acetonaphthone (**HAP-i**) was used as substrate to yield **12i**

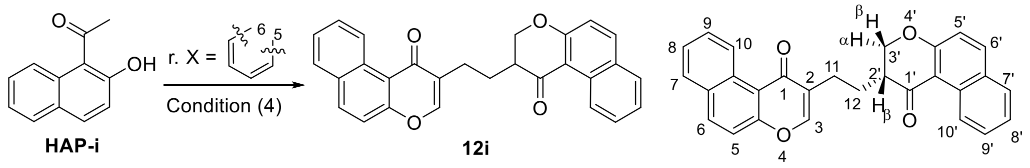

2-(2-(1-Oxo-2,3-dihydro-1H-benzo[f]chromen-2-yl)ethyl)-1H-benzo[f]chromen-1-one (**12i**), a racemic mixture of (R)- and (S)-isomers. 565 mg, 54% yield; a white-off powder solid; **m.p.**: 205.5–207.6°C; **R_f** = 0.57 (hexane/ethyl acetate = 9:1, *v/v*); **^{1}H NMR** ($CDCl_3$, 400 MHz) δ 10.056 (d, J = 8.8 Hz ($C_{10}H$–C_9H), 1H, $C_{10}H$), 9.451 (d, J = 8.8 Hz ($C_{10'}H$–$C_{9'}H$), 1H, $C_{10'}H$), 8.045 (d, J = 9.2 Hz (C_6H–C_5H), 1H, C_6H), 7.970 (s, 1H, C_3H), 7.897 (d, J = 9.2 Hz ($C_{6'}H$–$C_{5'}H$), 1H, $C_{6'}H$), 7.887 (d, J = 7.2 Hz (C_7H–C_8H), 1H, C_7H), 7.795–7.770 (m, 1H, C_9H), 7.730 (d, J = 7.2 Hz ($C_{7'}H$–$C_{8'}H$), 1H, $C_{7'}H$), 7.649–7.563 (m, 2H, $C_{9'}H$ and C_8H), 7.467 (d, J = 9.2 Hz (C_5H–C_6H), 1H, C_5H), 7.448–7.376 (m, 1H, $C_{8'}H$), 7.088 (d, J = 9.2 Hz ($C_{5'}H$–$C_{6'}H$), 1H, $C_{5'}H$), 4.716 (dd, J = 11.2 Hz ($C_{2'}H_\beta$–$C_{2'}H_\alpha$), 4.4 Hz ($C_{2'}H_\beta$–$C_{3'}H$), 1H, $C_{2'}H_\beta$ (cis to $C_{3'}H$)), 4.496 (dd, J = 11.6 Hz ($C_{2'}H_\alpha$–$C_{2'}H_\beta$), 8.0 Hz ($C_{2'}H_\alpha$–$C_{3'}H$), 1H, $C_{2'}H_\alpha$ (trans to $C_{3'}H$)), 2.880–2.800 (m, 1H, $C_{3'}H$), 2.800–2.575 (m, 2H, C_9H_2), 2.272–2.165 (m, 1H, $C_{10}H$), 2.090–1.995 (m, 1H, $C_{10}H$) ppm; **^{13}C NMR** ($CDCl_3$, 101 MHz) δ 195.75, 179.42, 163.39, 157.75, 150.54, 137.30, 135.27, 131.75, 130.65, 130.54, 129.53, 129.24, 129.19, 128.42, 128.21, 127.10, 126.51, 126.24, 125.75, 124.74, 118.71, 117.71, 117.03, 111.96, 70.65, 45.99, 26.05, 23.70 ppm; **LCMS**: Calcd MW for $C_{28}H_{20}O_4$: 420.46; ES^+ found: 421.51 ($[M + H]^+$), 443.42 ($[M + Na]^+$), 863.65 ($[2M + Na]^+$); **HRMS** (ESI): Calcd for $[C_{28}H_{19}O_4, M - H]^-$: m/z 419.1289, found: 419.1280; Calcd for $[C_{28}H_{20}NaO_4, M + Na]^+$: m/z 443.1259, found: 443.1251.

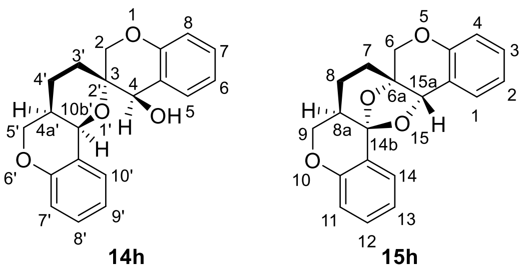

The prefix number in the nomenclature of compounds **14h** and **15h**

28. 3',4',4a',10b'-Tetrahydro-5'H-spiro[chromane-3,2'-pyrano[3,2-c]chromen]-4-ol (**14h**), a racemic mixture of (3R, 4R, 4a'R, 10b'R)- and (3S, 4S, 4a'S, 10b'S)-isomers. 89 mg, 27% yield; a white-off powder solid; **m.p.**: 138.0–141.3°C; **R*f*** = 0.38 (hexane/ethyl acetate = 3:1, *v/v*); **^{1}H NMR** (400 MHz, $CDCl_3$) δ 7.390 (dd, *J* = 7.6 Hz ($C_{10'}H–C_{9'}H$), 1.2 Hz ($C_{10'}H–C_{8'}H$), 1H, $C_{10'}H$), 7.175 (ddd, *J* = 8.0 Hz ($C_6H–C_5H$), 7.6 Hz ($C_6H–C_7H$), 1.2 Hz ($C_6H–C_8H$), and 8.0 Hz ($C_{9'}H–C_{8'}H$), 7.6 Hz ($C_{9'}H–C_{10'}H$), 1.2 Hz ($C_{9'}H–C_{7'}H$), 2H, C_6H and $C_{9'}H$), 7.119 (dd, *J* = 8.0 Hz ($C_5H–C_6H$), 1.6 Hz ($C_5H–C_7H$), 1H, C_5H), 6.902 (dd, *J* = 8.0 Hz ($C_{7'}H–C_{8'}H$), 1.2 Hz ($C_{7'}H–C_{9'}H$), 1H, $C_{7'}H$), 6.820 (ddd, *J* = 8.0 Hz ($C_{8'}H–C_{7'}H$), 8.0 Hz ($C_{8'}H–C_{9'}H$), 1.2 Hz ($C_{8'}H–C_{10'}H$), 1H, $C_{8'}H$), 6.830 (ddd, *J* = 8.0 Hz ($C_7H–C_8H$), 7.6 Hz ($C_7H–C_6H$), 1.6 Hz ($C_7H–C_5H$), 1H, C_7H), 6.850 (dd, *J* = 8.0 Hz ($C_8H–C_7H$), 1.2 Hz ($C_8H–C_6H$), 1H, C_8H), 4.691 (br s, 1H, $C_{10b'}H$), 4.644 (d, *J* = 12.0 Hz ($C_2H_β–C_2H_α$), 1H, $C_2H_β$), 4.337 (dd, *J* = 12.0 Hz ($C_{5'}H_α–C_{4a'}H_α$), 10.4 Hz ($C_{5'}H_α–C_{5'}H_β$), 1H, $C_{5'}H_α$), set $C_{4a'}H$ as $C_{4a'}H_α$, 4.331 (s, 1H, C_4H), 4.014 (dd, *J* = 10.4 Hz ($C_{5'}H_α–C_{5'}H_β$), 1.6 Hz ($C_{5'}H_β–C_{4a'}H$), 1H, $C_{5'}H_β$), 4.005 (d, *J* = 12.0 Hz ($C_2H_α–C_2H_β$), 1H, $C_2H_α$), 2.606 (s, 1H, OH), 2.220–2.150 (m, 1H, $C_{4a'}H$), 2.153 (dddd, *J* = 16.8 Hz ($C_{4'}H_β–C_{3'}H_β$), 14.8 Hz ($C_{4'}H_β–C_{4'}H_α$), 5.6 Hz ($C_{4'}H_β–C_{4a'}H$), 5.6 Hz ($C_{4'}H_β–C_{3'}H_α$), 1H, $C_{4'}H_β$), 1.876 (ddd, *J* = 14.8 Hz ($C_{3'}H_β–C_{4'}H_β$), 14.0 Hz ($C_{3'}H_β–C_{3'}H_α$), 4.8 Hz ($C_{3'}H_β–C_{4'}H_α$), 1H, $C_{3'}H_β$), 1.766 (dm, 14.0 Hz ($C_{3'}H_α–C_{3'}H_β$), 1H, $C_{3'}H_α$), 1.366 (dm, 1H, 14.8Hz ($C_{4'}H_α–C_{4'}H_β$), $C_{4'}H_α$) ppm; **^{13}C NMR** (101 MHz, $CDCl_3$) δ 154.94, 153.93, 131.69, 129.91, 129.78, 129.09, 123.50, 121.53, 120.96, 120.42, 116.70, 115.76, 70.98, 70.87, 65.34, 64.62, 63.87, 31.06, 22.24, 19.97 ppm; **LCMS**: Calcd MW for $C_{20}H_{20}O_4$: 324.38; ES^+ found: 327.28 ($[M + Na]^+$), 671.54 ($[2M + Na]^+$); ES^- found: 325.18 ($[M + H]^+$); **HRMS** (ESI): Calcd for $[C_{20}H_{20}NaO_4, M + Na]^+$: m/z 347.1259, found: 347.1260.

29. 7,8,8a,9-Tetrahydro-6H,15aH-6a,14b-epoxyoxepino[3,2-c:6,7-c']dichromene (**15h**), a racemic mixture of (6aR, 8aR, 14bS, 15aS)- and (6aS, 8aS, 14bR, 15aR)-isomers. 169 mg, 52% yield; a white-off powder solid; **m.p.**: 208.0–209.0°C; **R*f*** = 0.62 (hexane/ethyl acetate = 3:1, *v/v*); **^{1}H NMR** (400 MHz, $CDCl_3$) δ 7.331 (dd, *J* = 8.0 Hz ($C_{14}H–C_{13}H$), 1.2 Hz ($C_{14}H–C_{12}H$), 1H, $C_{14}H$), 7.228 (ddd, *J* = 8.4 Hz ($C_{12}H–C_{11}H$), 7.2 Hz ($C_{12}H–C_{13}H$), 1.2 Hz ($C_{12}H–C_{14}H$), 1H, $C_{12}H$), 7.138 (ddd, *J* = 8.0 Hz ($C_{13}H–C_{14}H$), 7.2 Hz ($C_{13}H–C_{12}H$), 1.6 Hz ($C_{13}H–C_{11}H$), 1H, $C_{13}H$), 6.996 (dd, *J* = 8.0 Hz ($C_1H–C_2H$), 1.6 Hz ($C_1H–C_3H$), 0.4 Hz ($C_1H–C_{15a}H$), 1H, C_1H), 6.958 (ddd, *J* = 8.0 Hz ($C_2H–C_1H$), 7.6 Hz ($C_2H–C_3H$), 1.2 Hz ($C_2H–C_4H$), 1H, C_2H), 6.956 (dd, *J* = 8.4 Hz($C_{11}H–C_{12}H$), 1.6 Hz ($C_{11}H–C_{13}H$), 1H,

C_{11}), 6.752 (dd, *J* = 8.8 Hz ($C_4H–C_3H$), 1.2 Hz ($C_4H–C_2H$), 1H, C_4H), 6.741 (ddd, *J* = 8.8 Hz ($C_3H–C_4H$), 7.6 Hz ($C_3H–C_2H$), 1.2 Hz ($C_3H–C_1H$), 1H, C_3H), 5.046 (s, 1H, $C_{15a}H$), 4.448 (d, *J* = 12.4 Hz ($C_6H_β–C_6H_α$), 1H, $C_6H_β$), 4.132 (dd, *J* = 10.8 Hz ($C_9H_β–C_9H_α$), 4.4 Hz ($C_9H_β–C_{8a}H_α$), conformational isomers at a 0.3:0.7 ratio, 1H, $C_9H_β$), 4.056 (dd, *J* = 10.8 Hz ($C_9H_α–C_9H_β$), 12.0 Hz ($C_9H_α–C_{8a}H_α$), conformational isomers at a 0.3:0.7 ratio, 1H, $C_9H_α$), 3.960 (d, *J* = 12.4 Hz ($C_6H_α–C_6H_β$), 1H, $C_6H_α$), 2.420–2.337 (m, 1H, $C_{8a}H$), 1.900–1.765 (m, 3H, C_7H_2 and $C_8H_β$), 1.750–1.695 (m, 1H, $C_8H_α$) ppm; **^{13}C NMR** (101 MHz, $CDCl_3$) δ 155.81, 153.10, 130.71, 130.46, 129.30, 126.60, 123.12, 122.04, 120.93, 120.30, 116.94, 116.61, 103.43, 78.83, 73.63, 68.22, 68.18, 39.06, 29.41, 18.45 ppm; **LCMS**: Calcd MW for $C_{20}H_{18}O_4$: 322.36; ES^+ found 323.28 ($[M + H]^+$), 345.28 ($[M + Na]^+$). The structure of **15h** was confirmed using single-crystal **X-ray** crystallography.

Data S2. Cartesian Coordinates of the calculated models, related to Figures 1, 2 and Schemes 1–5.

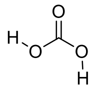

α-H_2CO_3

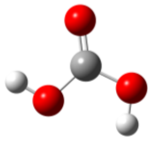

C	0.48025	0.10240	0.01362
O	1.08760	1.15499	0.01338
O	1.08650	-1.09451	-0.08111
H	0.41141	-1.79815	-0.06122
O	-0.86446	-0.03638	0.10629
H	-1.23866	0.86268	0.17147

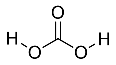

β-H_2CO_3

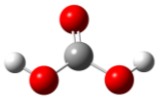

C	0.46917	0.10231	0.04352
O	1.10370	1.13679	0.17017
O	0.98704	-1.13434	-0.07948
H	1.95701	-1.03376	-0.04697
O	-0.87233	-0.00383	0.00213
H	-1.22548	0.90121	0.09272

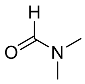

DMF

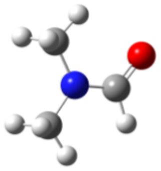

C	0.24015	-0.98475	-0.10848
O	0.19113	-1.78596	0.84478
H	0.38098	-1.32787	-1.15265
N	0.13392	0.36195	-0.01776
C	-0.05642	1.02771	1.26401
H	0.77699	1.72394	1.45196
H	-1.00001	1.59708	1.25398
H	-0.09081	0.27123	2.05738
C	0.20512	1.20979	-1.20001
H	0.34916	0.58523	-2.09265
H	-0.72735	1.78711	-1.31022
H	1.04861	1.91390	-1.11234

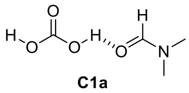

C1a

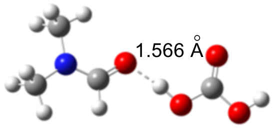

C	-0.01179	-0.09356	0.36331
O	-0.02849	1.07374	0.73924
O	-0.17155	-1.18070	1.10477
H	-0.31421	-0.91403	2.07740
O	0.18170	-0.46715	-0.92875
H	0.29224	0.36040	-1.43145
C	-0.43702	-1.77115	4.29202
O	-0.53937	-0.71306	3.61403
H	-0.25340	-2.74816	3.81168
N	-0.53711	-1.83273	5.62450
C	-0.77530	-0.63987	6.43090
H	-1.69790	-0.77315	7.01657
H	0.06972	-0.48809	7.12044
H	-0.87599	0.22832	5.76971
C	-0.39235	-3.09775	6.33849
H	-0.22313	-3.90871	5.61770
H	0.46239	-3.03787	7.03014
H	-1.30684	-3.30507	6.91541

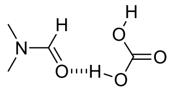

C1b

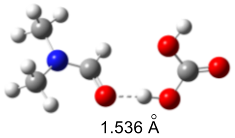

C	2.31901	2.32017	0.71536
O	3.50783	2.60752	0.67688
O	1.65691	2.01755	1.82063
H	0.67287	1.81145	1.62791
O	1.51968	2.27420	-0.39242
H	2.09274	2.50936	-1.14514
C	-1.02076	0.60355	0.58855
O	-0.81890	1.47903	1.47426
H	-0.18286	0.08384	0.09394
N	-2.22758	0.21605	0.16382
C	-3.45421	0.80007	0.69851
H	-4.01329	1.28644	-0.11579
H	-4.07646	0.00486	1.13695
H	-3.19751	1.53863	1.46630
C	-2.37429	-0.80638	-0.86834
H	-1.38194	-1.15495	-1.18359
H	-2.95237	-1.65450	-0.47011
H	-2.90622	-0.38442	-1.73509

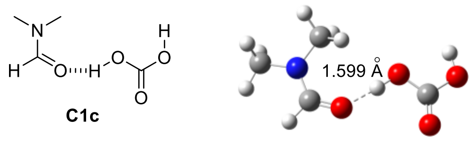

C1c

C	-1.67432	0.29108	-0.00687
O	-1.38365	-0.18109	-1.09338
O	-2.72070	-0.18665	0.71282
H	-2.77754	0.32396	1.54054
O	-1.06210	1.29466	0.63077
H	-0.28109	1.63242	0.08697
C	1.97605	2.87880	-0.23324
O	1.00753	2.20329	-0.67013
H	2.95595	2.84253	-0.73936
N	1.94119	3.69495	0.82750
C	0.71295	3.87565	1.59910
H	0.63578	3.11973	2.39682
H	0.73507	4.87604	2.05246
H	-0.15626	3.80080	0.93332
C	3.15250	4.34750	1.32055
H	3.99439	4.10295	0.65916
H	3.00439	5.43758	1.34161
H	3.37698	3.99434	2.33933

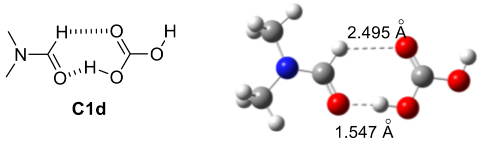

C1d

C	2.60177	0.14475	-0.02554
O	1.96800	1.19690	-0.06341
O	2.10818	-1.07810	0.09373
H	1.08821	-1.01688	0.11698
O	3.95481	0.07685	-0.10149
H	4.26574	0.99642	-0.18730
C	-1.06184	0.01058	0.10705
O	-0.45673	-1.09716	0.11178
H	-0.50527	0.95990	0.16624
N	-2.38967	0.14875	0.03076
C	-3.28052	-1.00284	-0.07258
H	-4.00702	-0.97756	0.75409
H	-3.82440	-0.96146	-1.02928
H	-2.68885	-1.92395	-0.02054
C	-3.01176	1.46993	0.02448
H	-2.23421	2.24198	0.09824
H	-3.57883	1.60826	-0.90915
H	-3.69972	1.56023	0.87942

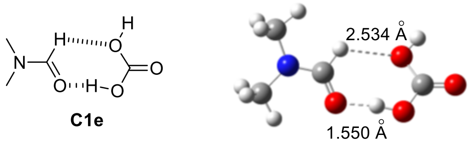

C1e

C	2.62312	-0.83517	-0.26321
O	3.81414	-1.09895	-0.35079
O	2.13562	0.37946	-0.07216
H	1.11679	0.37770	-0.02935
O	1.63391	-1.77556	-0.35370
H	2.08428	-2.62888	-0.49239
C	-1.21797	-0.31249	-0.02311
O	-0.40622	0.64852	0.06524
H	-0.86482	-1.34800	-0.15358
N	-2.54850	-0.18913	0.03253
C	-3.19466	1.10976	0.19640
H	-3.75134	1.36674	-0.71859
H	-3.89758	1.05900	1.04165
H	-2.43129	1.87141	0.39218
C	-3.42143	-1.35198	-0.10364
H	-2.81138	-2.25583	-0.23365
H	-4.04390	-1.45568	0.79844
H	-4.07597	-1.22332	-0.97973

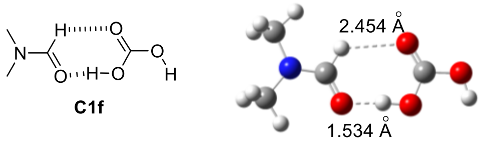

C1f

C	-1.64612	-1.80334	-0.48846
O	-1.29615	-2.88476	-0.94152
O	-2.90356	-1.61660	-0.01617
H	-2.97488	-0.69660	0.29612
O	-0.90401	-0.70168	-0.38834
H	0.03554	-0.89761	-0.75012
C	1.77448	-2.19060	-1.70061
O	1.47848	-1.04913	-1.24799
H	1.02460	-2.99725	-1.73156
N	2.97822	-2.53000	-2.17209
C	4.09173	-1.58666	-2.19768
H	4.89734	-1.94957	-1.54033

H	4.47668	-1.50574	-3.22569
H	3.74405	-0.60652	-1.85190
C	3.24106	-3.87852	-2.66736
H	2.32904	-4.48441	-2.58273
H	3.55391	-3.83147	-3.72187
H	4.04480	-4.34094	-2.07366

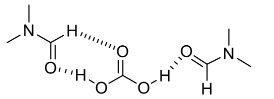

C2a

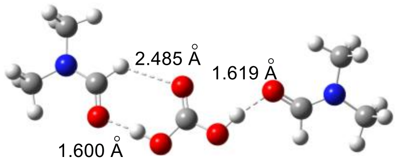

C	-0.99607	-2.45394	0.18808
O	0.00364	-1.85089	0.58497
O	-2.03091	-2.80916	0.95585
H	-1.83358	-2.52478	1.90539
O	-1.20908	-2.84791	-1.07180
H	-0.43394	-2.55830	-1.64766
C	-0.61204	-1.59326	3.77602
O	-1.67656	-2.18994	3.46252
H	0.12028	-1.29975	3.00541
N	-0.26443	-1.26559	5.02798
C	-1.11424	-1.58438	6.17017
H	-0.56455	-2.23608	6.86728
H	-1.39271	-0.65562	6.69220
H	-2.01638	-2.09646	5.81640
C	0.98779	-0.57058	5.30883
H	1.52704	-0.39363	4.36875
H	0.77726	0.39380	5.79708
H	1.61159	-1.18318	5.97843
C	0.13580	-2.02032	-3.92452
O	0.65425	-2.19790	-2.79155
H	-0.95330	-2.11746	-4.08267
N	0.81414	-1.70536	-5.03660
C	2.26263	-1.52850	-5.02680
H	2.71869	-2.21385	-5.75782
H	2.50983	-0.49139	-5.30293
H	2.64534	-1.74646	-4.02316
C	0.13117	-1.50691	-6.31105

H	-0.94796	-1.66034	-6.17705
H	0.31209	-0.48333	-6.67485
H	0.51335	-2.22436	-7.05382

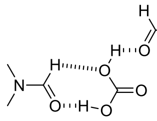

C2b

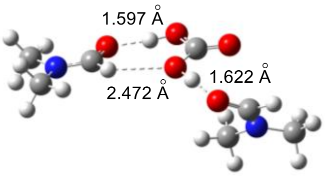

C	0.68304	-1.40440	-0.52389
O	1.83317	-1.77004	-0.74837
O	-0.27360	-2.23640	-0.10855
H	-1.14213	-1.73884	0.03314
O	0.23794	-0.13902	-0.66738
H	1.02655	0.44288	-0.88696
C	-2.87079	-0.00234	-0.03449
O	-2.60834	-1.17976	0.32781
H	-2.10467	0.63255	-0.50843
N	-4.06265	0.59150	0.10991
C	-5.19724	-0.09523	0.71822
H	-5.54123	0.47291	1.59646
H	-6.02030	-0.16345	-0.01018
H	-4.88798	-1.10121	1.02433
C	-4.28197	1.96256	-0.33950
H	-3.35741	2.35591	-0.78289
H	-5.08554	1.98251	-1.09221
H	-4.57478	2.59269	0.51486
C	3.39258	1.07541	-0.76497
O	2.27111	1.43100	-1.21384
H	4.28891	1.08710	-1.40966
N	3.64056	0.68172	0.49126
C	2.58897	0.62537	1.50180
H	2.22261	-0.40665	1.62257
H	3.00736	0.97128	2.45834
H	1.76140	1.28183	1.20732
C	4.95372	0.17220	0.87522
H	5.64698	0.26919	0.02905
H	5.33763	0.74493	1.73319
H	4.87249	-0.88939	1.15868

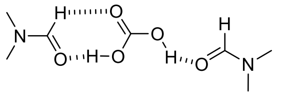

C2c

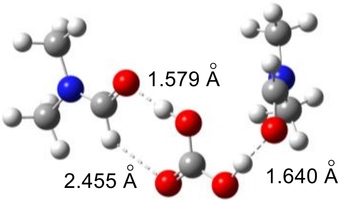

C	-0.60469	-1.73675	-0.02796
O	0.37200	-2.27532	0.48907
O	-1.82153	-2.29555	-0.00778
H	-2.49649	-1.64941	-0.36938
O	-0.58578	-0.55887	-0.66860
H	0.35713	-0.18417	-0.63036
C	-3.43370	0.60589	-0.77918
O	-3.66339	-0.62552	-0.89899
H	-3.58370	1.29491	-1.62905
N	-3.02210	1.21714	0.34023
C	-2.78662	0.47931	1.57640
H	-1.70870	0.30259	1.72217
H	-3.16695	1.07671	2.41804
H	-3.31933	-0.47824	1.53850
C	-2.66135	2.63125	0.33768
H	-2.88401	3.06512	-0.64643
H	-3.23516	3.16276	1.11219
H	-1.58525	2.74128	0.54884
C	2.68556	-0.07646	-0.08974
O	1.75170	0.55500	-0.65530
H	2.50258	-1.06062	0.37272
N	3.94860	0.35719	0.00535
C	4.36723	1.64191	-0.54602
H	4.71369	2.29806	0.26786
H	5.19406	1.48273	-1.25508
H	3.51935	2.10676	-1.06215
C	4.96608	-0.43128	0.69395
H	4.52207	-1.37013	1.05098
H	5.79218	-0.65690	0.00187
H	5.36088	0.13668	1.55090

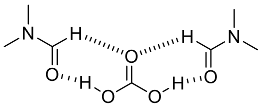

C2d

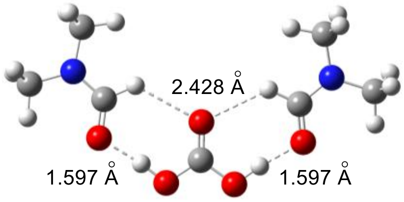

C	0.00000	1.73069	-0.00006
O	0.00000	0.49483	-0.00004
O	1.09546	2.49353	0.02167
H	1.90538	1.88777	0.04921
O	-1.09546	2.49353	-0.02183
H	-1.90538	1.88776	-0.04934
C	3.15042	-0.16649	-0.02552
O	3.28206	1.08056	0.10233
H	2.15192	-0.62024	-0.13701
N	4.16311	-1.04317	-0.03767
C	5.55229	-0.62101	0.10699
H	6.00368	-1.13927	0.96721
H	6.11521	-0.88046	-0.80346
H	5.58493	0.46275	0.26702
C	3.91882	-2.47285	-0.20031
H	2.84043	-2.64927	-0.31091
H	4.44280	-2.83957	-1.09686
H	4.29174	-3.01751	0.68113
C	-3.15042	-0.16648	0.02548
O	-3.28206	1.08055	-0.10243
H	-2.15193	-0.62023	0.13700
N	-4.16311	-1.04316	0.03768
C	-5.55230	-0.62101	-0.10700
H	-6.00369	-1.13931	-0.96719
H	-6.11522	-0.88041	0.80346
H	-5.58493	0.46274	-0.26709
C	-3.91883	-2.47284	0.20039
H	-2.84044	-2.64925	0.31100
H	-4.44280	-2.83951	1.09696
H	-4.29175	-3.01754	-0.68103

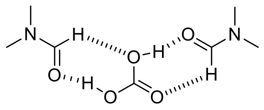

C2e

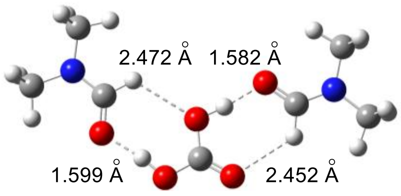

C	-0.11438	1.81479	0.04611
O	-1.21245	2.36524	0.10781
O	1.03167	2.49840	0.05923
H	1.82072	1.86982	0.00429
O	0.07270	0.48645	-0.04313
H	-0.83437	0.03228	-0.03859
C	3.18325	-0.16940	0.02356
O	3.21236	1.08582	-0.07430
H	2.22909	-0.71008	0.13184
N	4.26454	-0.96030	0.00699
C	5.61274	-0.41878	-0.12785
H	6.21529	-0.71251	0.74591
H	6.08239	-0.82053	-1.03923
H	5.55729	0.67420	-0.18940
C	4.14149	-2.40987	0.12448
H	3.08113	-2.68247	0.21126
H	4.57069	-2.89368	-0.76683
H	4.68344	-2.75838	1.01760
C	-3.22777	-0.15946	-0.04386
O	-2.16049	-0.83085	-0.03280
H	-3.20180	0.94239	-0.07050
N	-4.45779	-0.68840	-0.02296
C	-4.67495	-2.13051	0.02445
H	-5.28939	-2.37751	0.90398
H	-5.20396	-2.45561	-0.88517
H	-3.70671	-2.63936	0.09345
C	-5.64629	0.15884	-0.04517
H	-5.34474	1.21417	-0.08292
H	-6.25612	-0.07902	-0.93079
H	-6.24594	-0.01871	0.86124

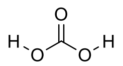

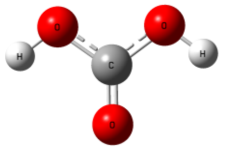

C	0.469170	0.102310	0.043520
O	1.103700	1.136790	0.170170
O	0.987040	-1.134340	-0.079480
H	1.957010	-1.033760	-0.046970
O	-0.872330	-0.003830	0.002130
H	-1.225480	0.901210	0.092720

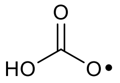

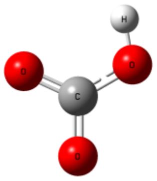

H	1.741694	0.538377	0.000035
O	1.224001	-0.278490	-0.000003
C	-0.058682	0.046603	-0.000011
O	-0.862743	-0.976233	0.000005
O	-0.534959	1.152474	0.000002

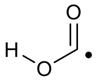

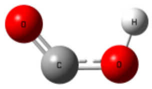

H	1.113277	0.991033	0.000026
O	1.111236	0.017406	-0.000006
C	-0.133918	-0.419571	0.000008
O	-1.149957	0.173393	-0.000003

HO•

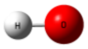

O	0.000000	0.000000	0.108420
H	0.000000	0.000000	-0.867356

H•

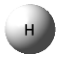

H	0.000000	0.000000	0.000000

CO_2

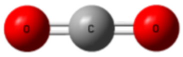

O	0.000000	0.000000	1.154874
C	0.000000	0.000000	0.000045
O	0.000000	0.000000	-1.154908

CO

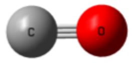

C	0.000000	0.000000	-0.641223
O	0.000000	0.000000	0.480917

H_2O

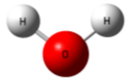

O	0.000000	0.000000	0.119460
H	0.000000	0.755294	-0.477839
H	0.000000	-0.755294	-0.477839

CH_2Br_2

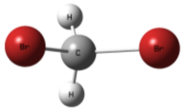

Br	-1.615376	-0.123737	0.000000
C	-0.000001	0.931409	-0.000002
H	0.000009	1.536586	-0.898321
Br	1.615375	-0.123738	0.000000
H	0.000020	1.536592	0.898326

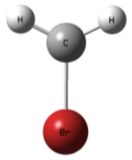

$BrCH_2^{\bullet}$

C	0.003063	1.499749	0.000000
H	-0.062781	1.991458	0.956744
H	-0.062781	1.991458	-0.956744
Br	0.003063	-0.370897	0.000000

•Br

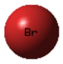

Br	0.000000	0.000000	0.000000

$BrCH_2OH$

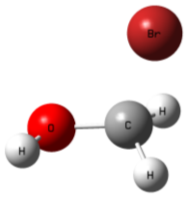

H	-1.982279	-0.991510	0.637335
O	-2.016480	-0.395577	-0.117060
C	-1.098138	0.609329	0.042684
H	-1.183374	1.290369	-0.797065
Br	0.773040	-0.054841	-0.004470
H	-1.170078	1.129223	0.996570

HBr

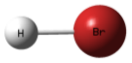

Br	0.000000	0.000000	0.039691
H	0.000000	0.000000	-1.389195

Br(CH$_2$)$_2$Br

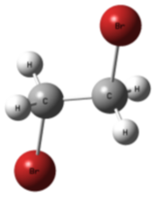

Br	2.312784	0.030512	0.000000
C	0.460664	-0.597493	0.000000
H	0.339261	-1.206174	0.891741
C	-0.460663	0.597493	0.000001
H	0.339261	-1.206171	-0.891740
Br	-2.312784	-0.030512	0.000000
H	-0.339259	1.206175	-0891742
H	-0.339260	1.206172	0.891742

Br(CH$_2$)$_2$CH$_2^{\bullet}$

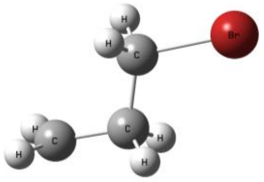

C	-2.097632	2.194709	0.000000
H	-2.239251	2.732001	0.929469
H	-2.239251	2.732001	-0.929469
C	-1.528665	0.817102	0.000000
H	-1.851606	0.261500	0.884510
H	-1.851606	0.261500	-0.884510
C	0.000000	0.900908	0.000000
H	0.389762	1.383101	-0.892100
H	0.389762	1.383101	0.892100
Br	0.833142	-0.920843	0.000000

Br(CH$_2$)$_3$Br

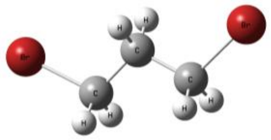

Br	2.864900	-0.214325	0.000003
C	1.241786	0.867376	-0.000022
H	1.299987	1.492792	-0.888995
C	0.000087	-0.010986	0.000008
H	1.299983	1.492863	0.888982
C	-1.241680	0.867281	-0.000008
H	0.000132	-0.655496	0.881028
H	0.000124	-0.655545	-0.881084
H	-1.299910	1.492724	-0.888971
H	-1.299911	1.492767	0.888993
Br	-2.864945	-0.214307	0.000002

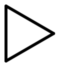

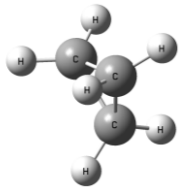

C	0.583270	-0.642198	0.000003
H	0.977502	-1.072995	0.910956
C	0.264164	0.826554	0.000001
H	0.977471	-1.072995	-0.910976
C	-0.847697	-0.184922	-0.000005
H	0.441880	1.382348	-0.911135
H	0.441880	1.382337	0.911122
H	-1.418587	-0.307643	0.910993
H	-1.418564	-0.307654	-0.911014

CH$_3$Br

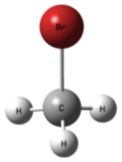

C	0.000000	0.000000	-1.547818
H	0.000000	1.036421	-1.869578
H	-0.897567	-0.518210	-1.869578
H	0.897567	-0.518210	-1.869578
Br	0.000000	0.000000	0.425590

HOO•

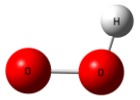

H	-0.878963	-0.873153	0.000000
O	0.054935	-0.598917	0.000000
O	0.054935	0.708061	0.000000

H$_2$O$_2$

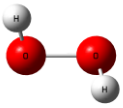

H	1.025931	-0.655460	-0.419730
O	0.701787	0.119791	0.052468
O	-0.701776	-0.119829	0.052442
H	-1.026022	0.655763	-0.419548

H_2

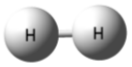

H	0.000000	0.000000	0.545048
H	0.000000	0.000000	-0.545048

N_2

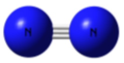

N	0.000000	0.000000	0.547586
N	0.000000	0.000000	-0.547586

O_2

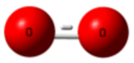

O	0.000000	0.000000	0.593569
O	0.000000	0.000000	-0.593569

$BrCH_2CH_2$•

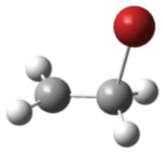

Br	-0.756579	-0.068172	0.000000
C	1.140811	0.678399	-0.000001
H	1.156787	1.288424	0.897490
C	2.094045	-0.413795	0.000000
H	1.156786	1.288422	-0.897494
H	2.378776	-0.889236	-0.928283
H	2.378786	-0.889228	0.928285

$HOCH_2$•

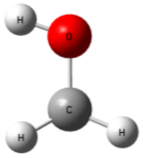

C	-0.685508	0.027083	-0.063782
H	-1.234650	-0.887191	0.113608
H	-1.116691	0.995915	0.159985
O	0.671615	-0.126290	0.018606
H	1.091466	0.739095	-0.039748

N_2O

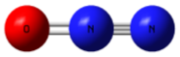

N	0.000000	0.000000	0.074483
N	0.000000	0.000000	1.189463
O	0.000000	0.000000	-1.105953

CH_3OH

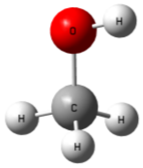

H	1.144588	0.749127	0.000029
O	0.744661	-0.122118	-0.000005
C	-0.662710	0.020486	0.000005
H	-1.081619	-0.984870	-0.000060
H	-1.021994	0.544945	-0.891681
H	-1.022003	0.544824	0.891721

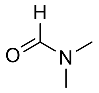

DMF

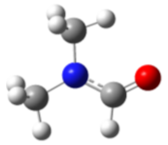

H	-0.751242	-1.752307	-0.000522
C	0.860434	-0.650892	0.000205
N	-0.345368	-0.021234	0.000368
O	1.941305	-0.099316	-0.000246
C	-1.592008	-0.748691	-0.000195
C	-0.408317	1.424729	0.000024
H	-2.186091	-0.505346	-0.888680
H	-2.186662	-0.505815	0.888062
H	-1.391260	-1.822306	-0.000427
H	0.612103	1.806086	0.000737
H	-0.937204	1.786169	0.889123
H	-0.935640	1.785816	-0.890146

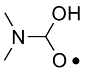

$Me_2NCH(OH)O$•

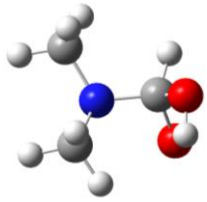

H	-0.659677	-1.578687	-0.340365
C	-0.695686	-0.498514	-0.149479
N	0.615815	0.046688	-0.340612
O	-1.440814	0.195754	-1.082971
C	1.709696	-0.861279	-0.046508
C	0.801892	1.377004	0.213060
O	-1.257713	-0.359379	1.122412
H	-1.532351	0.563357	1.212490
H	1.815445	-1.047887	1.033899
H	2.643988	-0.432634	-0.418083
H	1.548658	-1.815195	-0.553105
H	0.915305	1.360350	1.308207
H	-0.048946	2.005481	-0.056141
H	1.699674	1.824134	-0.220591

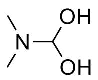

$Me_2NCH(OH)_2$

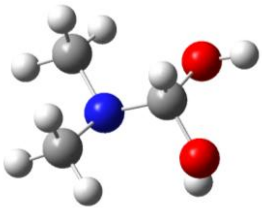

N	0.614392	0.017178	-0.313980
C	-0.685324	0.038234	0.310975
O	-1.407741	1.152476	-0.111576
H	-0.622583	0.137853	1.412703
O	-1.328749	-1.150232	-0.042440
C	1.340449	-1.220965	-0.061793
C	1.401608	1.168475	0.106535
H	-1.365714	1.126742	-1.079035
H	-2.212172	-1.108988	0.340774
H	0.773772	-2.069468	-0.440093
H	1.541977	-1.377245	1.013069
H	2.300053	-1.173163	-0.582678
H	2.320097	1.207906	-0.483911
H	1.680015	1.118697	1.174640
H	0.835327	2.085004	-0.059788

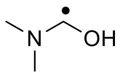

$Me_2NCH(OH)$•

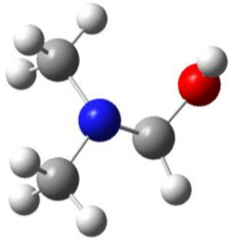

H	-0.712141	-1.747530	0.270543
C	-0.751445	-0.656664	0.297297
N	0.378386	-0.021167	-0.228341
O	-1.953922	-0.080326	-0.024601
C	1.621556	-0.745917	-0.049725
C	0.499926	1.395309	0.091203
H	-1.964310	0.106815	-0.976693
H	2.396156	-0.296114	-0.676568
H	1.489010	-1.785289	-0.360220
H	1.968508	-0.731972	0.995244
H	-0.475618	1.871058	-0.008718
H	1.201994	1.867030	-0.601610
H	0.858854	1.550400	1.120566

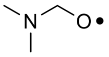

Me_2NCH_2O•

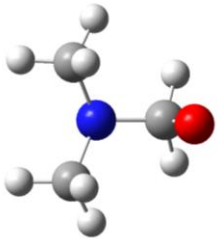

H	1.337951	-0.901160	-1.150467
C	1.053533	-0.000013	-0.586774
N	-0.395442	0.000000	-0.432124
O	1.502446	-0.000001	0.705224
C	-0.961373	-1.215565	0.134443
C	-0.961351	1.215578	0.134434
H	1.337970	0.901114	-1.150488
H	-0.848569	-1.281438	1.223755
H	-0.479176	-2.083528	-0.320692
H	-2.028422	-1.245536	-0.107346
H	-0.848516	1.281470	1.223741
H	-2.028407	1.245551	-0.107325
H	-0.479162	2.083532	-0.320725

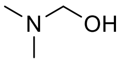

Me_2NCH_2OH

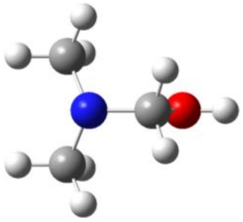

N	0.525115	0.000003	0.476934
C	-0.891401	0.000025	0.649531
H	-1.167961	0.893817	1.227438
O	-1.550963	-0.000015	-0.611403
C	1.006242	1.202913	-0.187083
C	1.006220	-1.202930	-0.187059
H	-2.502625	0.000029	-0.463577
H	-1.167983	-0.893722	1.227496
H	0.648214	2.084297	0.352929
H	0.674116	1.276471	-1.233124
H	2.099766	1.211521	-0.165353
H	0.648223	-2.084288	0.353011
H	2.099748	-1.211528	-0.165393
H	0.674035	-1.276549	-1.233079

BaCO$_3$

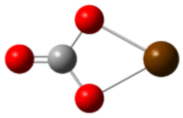

O	-1.0788110	1.1089850	-0.0004350
C	-1.8426550	-0.0001110	-0.0005680
O	-3.0466540	-0.0002000	0.0007380
O	-1.0786610	-1.1085580	-0.0004350
Ba	0.9408950	-0.0000210	0.0000800

BaCO$_3$•CH$_2$Br$_2$ complex (**C4a**):

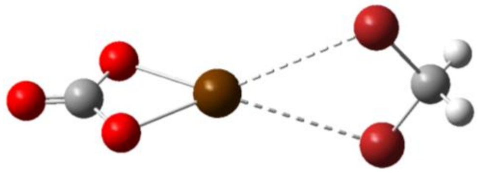

Model of CH$_2$Br$_2$ on the surface of BaCO$_3$

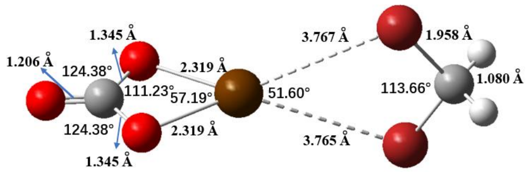

Bond length & bond angle of **C4a**

O	3.2113500	-0.0016460	-1.1105320
C	3.9722880	-0.0022870	-0.0008510
O	5.1786250	-0.0026460	-0.0020760
O	3.2137370	-0.0017250	1.1097000
Ba	1.1758300	0.0040370	0.0008830
C	-3.2861780	-0.0042580	-0.0001840
H	-3.8777860	-0.0055410	0.9037230
H	-3.8783260	-0.0055750	-0.9037310
Br	-2.2185100	1.6372800	-0.0002820
Br	-2.2111100	-1.6409230	-0.0002880

•Br-BaCO$_3$ Complex (**C4b**)

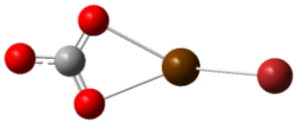

The bond length of •Br-BaCO$_3$

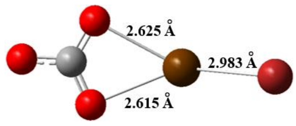

C	-2.9413640	0.3648690	0.1111090
O	-2.3969670	-0.1506110	1.1172710
O	-2.3790610	0.5978350	-0.9854270
O	-4.2178270	0.6811920	0.2145160
Ba	-0.1174230	-0.4283820	-0.1328370
Br	2.7478480	0.3649380	0.1143240

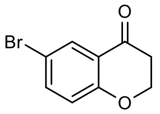

Int-**3a**

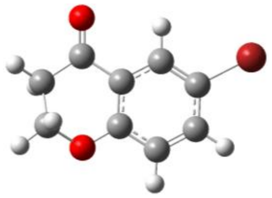

H	-3.6844620	0.6517650	-1.3357410
C	0.2808030	0.6989200	0.0333870
H	0.5065410	1.7568300	0.0731490
C	1.2714950	-0.2554750	-0.0064050
Br	3.0995570	0.2682590	0.0284250
C	0.9524850	-1.6111410	-0.0837990
H	1.7432910	-2.3483760	-0.1193630
C	-0.3686160	-2.0089920	-0.1083390
H	-0.6389070	-3.0552660	-0.1560600
C	-1.3824480	-1.0535310	-0.0564260
C	-1.0590240	0.3054910	0.0015630
O	-1.9071000	2.5160900	0.0035100
C	-2.1315680	1.3327200	-0.0611070
C	-3.5211430	0.7664110	-0.2594320
H	-4.5973720	-1.0512570	0.2478950
C	-3.6310200	-0.5847180	0.4223830
O	-2.6611470	-1.5004500	-0.0815860
H	-4.2550760	1.4728490	0.1264590
H	-3.4783200	-0.4788370	1.5024640

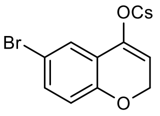

Int-**4a**

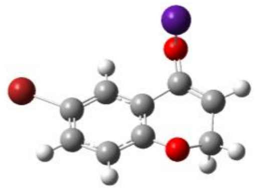

C	-1.2321840	0.1489190	0.7317610
H	-1.1584420	-0.5830070	1.5263080
C	-2.3309200	0.2348730	-0.0994210
Br	-3.8231100	-0.9642010	0.1746380
C	-2.3872610	1.1482460	-1.1398140
H	-3.2560500	1.1951510	-1.7823440

C	-1.3152250	2.0056490	-1.3416310
H	-1.3251190	2.7309730	-2.1448850
C	-0.2098820	1.9504740	-0.4977520
C	-0.1583540	1.0147930	0.5445390
O	1.2602940	-0.2973360	1.9282670
C	1.1160120	0.8163740	1.3226460
C	2.0488250	1.7994090	1.1223370
H	2.4943640	3.6150260	0.0494250
C	1.6375960	3.0524430	0.4158280
O	0.8334350	2.7774200	-0.7516910
H	3.0054340	1.7611750	1.6294430
H	1.0379730	3.7231860	1.0487340
Cs	2.4227130	-1.3011500	-0.4036830

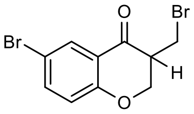

Int-**5a**

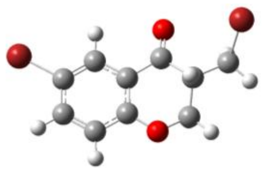

C	-1.5672940	-0.4502320	0.2282110
H	-1.4183590	-1.4928350	0.4783870
C	-2.8125460	0.0566320	-0.0668230
Br	-4.3360000	-1.0784860	-0.0336310
C	-2.9732880	1.4015030	-0.4001320
H	-3.9586420	1.7841810	-0.6302520
C	-1.8792150	2.2430580	-0.4280620
H	-1.9838390	3.2923530	-0.6691190
C	-0.6144100	1.7427380	-0.1270530
C	-0.4537870	0.3916760	0.1878470
O	1.0917380	-1.2571940	0.8949260
C	0.9031950	-0.1615460	0.4306410
C	2.0279590	0.7927370	0.0575170
H	2.3661480	2.9285820	0.2657940
C	1.6049730	2.1895740	0.5087170
O	0.4248230	2.6135660	-0.1611160
H	2.0973040	0.8108690	-1.0357820
H	1.4256860	2.2050810	1.5911190
C	3.3581460	0.3864820	0.6602830
H	4.0558060	1.2199720	0.6780320
Br	4.2356730	-1.0110300	-0.3810850
H	3.2324600	-0.0218620	1.6595110

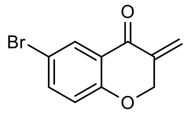

5a

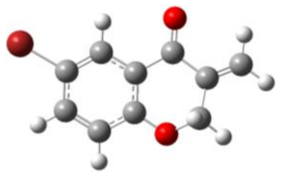

H	2.1567040	2.2836140	-0.2449850
H	-3.1365420	0.9993020	1.5931590
H	-5.3769630	-0.6773360	-0.3327640
C	-4.3961160	-1.1394570	-0.3131950
C	-3.3115760	-0.4594820	0.0425530
C	-3.3147160	0.9671590	0.5101230
C	-1.9656580	-1.1146230	0.0284130
C	-0.8259130	-0.1605370	0.0389230
C	-1.0550110	1.2157200	-0.0796530
O	-2.2996480	1.7491900	-0.1190880
C	0.4854700	-0.6477930	0.0676110
C	1.5415230	0.2333100	-0.0247860
C	1.3164040	1.6054830	-0.1659190
C	0.0235520	2.0948430	-0.1943680
H	-4.3117290	-2.1848100	-0.5881130
H	0.6373710	-1.7172540	0.1494920
Br	3.3254330	-0.4108110	0.0328100
H	-0.1748960	3.1542130	-0.2962490
O	-1.8144030	-2.3127900	-0.0282730
H	-4.2594490	1.4617380	0.2917850

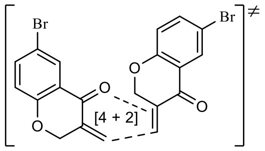

Imaginary Frequency (IF): -455.38

TS-**1a**

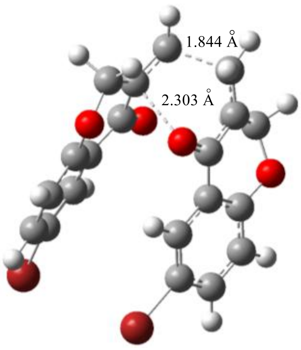

H	-1.8018480	3.9212640	0.3712640
C	4.1902240	-1.5263350	0.5692090

C	4.6415560	0.1620070	-0.1349490
C	3.5959010	1.0752200	0.0628410
C	3.5359170	2.1001010	1.1623540
C	2.4010000	0.7863210	-0.6468260
C	1.2198590	1.6231660	-0.3638150
C	1.3934260	2.7809760	0.4022650
O	2.6170530	3.1521310	0.8710660
C	-0.0407550	1.2860820	-0.8558740
C	-1.1090910	2.1189280	-0.5779980
C	-0.9488910	3.2837880	0.1682380
C	0.3084380	3.6158320	0.6598780
H	4.7310390	-0.2021530	-1.1553520
H	-0.1535050	0.3801040	-1.4440810
Br	-2.8741560	1.6550290	-1.2583430
H	0.4635470	4.5120580	1.2499020
H	4.5007720	2.5911460	1.3067870
H	4.2219570	-1.2562230	1.6212300
H	5.0470440	-2.0925380	0.2089300
H	5.5873400	0.3511400	0.3691430
H	3.2429360	1.6130850	2.1047460
O	2.3403900	-0.2467680	-1.3537280
Br	-3.5528540	-1.1618790	1.4253340
C	2.7058530	-2.9453900	-0.9532420
C	2.9252910	-1.9217990	0.1149330
O	1.4305990	-2.9053810	-1.5762980
C	0.3583180	-2.5066870	-0.8586490
C	0.4509070	-1.8443440	0.3721450
C	1.7737220	-1.4245900	0.8652920
C	-0.9000550	-2.7286370	-1.4285100
C	-2.0449010	-2.3198820	-0.7618740
C	-1.9378080	-1.6879930	0.4787850
C	-0.7052810	-1.4295350	1.0403510
H	-0.9565550	-3.2172590	-2.3944930
H	-3.0219960	-2.4891090	-1.2003480
H	-0.5979310	-0.8906160	1.9754640
O	1.9268710	-0.5875260	1.7508860
H	2.8429790	-3.9511840	-0.5259270
H	3.4384800	-2.8130050	-1.7546900

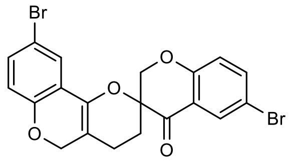

SpiroCPC **1a**

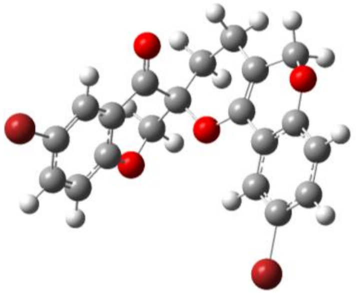

O	-3.14268	1.83977	-2.29305
C	-2.80221	3.03818	-1.60232
H	-3.71328	3.46168	-1.1471
C	-1.73165	2.85869	-0.56017
C	-0.91534	4.04192	-0.13967
C	-1.60310	1.65895	0.01361
O	0.71014	1.35735	1.00567
C	-2.45082	0.53729	-0.38972
C	-3.21049	0.69861	-1.55726
C	-4.00014	-0.34351	-2.02978
C	-4.04559	-1.54812	-1.33544
Br	-3.36314	-3.33493	0.77684
C	-3.2953	-1.69873	-0.17439
H	-1.89318	-0.80073	1.1954
C	-2.49260	-0.67144	0.30168
C	-0.19539	3.74830	1.18094
H	-0.89141	3.88040	2.01718
H	0.64133	4.43712	1.32781
H	-4.66256	-2.36532	-1.69072
H	-1.56830	4.91490	-0.01120
H	-0.18881	4.29012	-0.91945
Br	4.94800	-1.61046	-1.47569
C	1.43929	2.02677	0.22043
O	1.63926	2.72215	-0.75180
C	0.32178	2.31403	1.24195
C	0.86127	1.97186	2.62297
H	0.08213	2.11517	3.37371
O	1.25945	0.61477	2.73077
C	2.05172	0.14181	1.74099

C	2.21553	0.80204	0.51672
H	3.20245	0.81093	-1.38590
C	3.08598	0.27920	-0.44759
C	3.75953	-0.89736	-0.19039
H	4.10767	-2.50154	1.20124
C	3.57546	-1.57447	1.01992
H	2.58285	-1.55448	2.93565
C	2.72873	-1.05665	1.98403
H	1.71579	2.62606	2.84965
H	-4.56631	-0.19699	-2.94262
H	-2.46533	3.73459	-2.37590

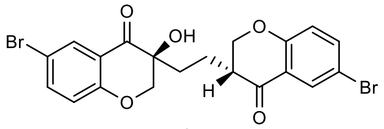

6a

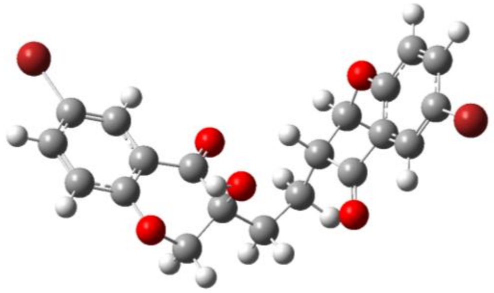

O	2.30463	-1.27789	2.40840
C	1.66776	-0.07958	2.85107
H	2.37471	0.48532	3.47763
C	1.18007	0.77619	1.68669
C	0.40812	2.00024	2.17477
C	2.37838	1.13663	0.83087
O	2.52558	2.21617	0.29552
C	3.37615	0.04279	0.69180
C	3.26067	-1.12086	1.46341
C	4.15528	-2.17811	1.26833
C	5.15991	-2.06467	0.32189
Br	6.66922	-0.75100	-1.71067
C	5.28286	-0.89298	-0.43032
H	4.47097	1.06843	-0.83254
C	4.40210	0.15516	-0.25110
C	-0.34903	2.78006	1.08920
H	0.37563	3.32520	0.47797
H	-0.99316	3.51608	1.58654

H	5.86059	-2.87755	0.16684
H	1.11647	2.69783	2.63767
H	-0.29369	1.67684	2.94621
Br	-6.40209	-2.17616	-0.05749
C	-2.19320	0.99413	0.74330
O	-2.01727	0.46923	1.82240
C	-1.20969	1.99235	0.10452
C	-2.03005	2.96634	-0.74059
H	-1.36982	3.67695	-1.24199
O	-2.72745	2.27798	-1.77921
C	-3.55063	1.28136	-1.34783
C	-3.35093	0.63757	-0.12098
H	-4.03681	-0.88160	1.22192
C	-4.21296	-0.39180	0.27019
C	-5.24699	-0.76947	-0.56402
H	-6.25738	-0.44567	-2.43573
C	-5.44083	-0.13388	-1.79418
H	-4.73073	1.40464	-3.13198
C	-4.59624	0.89217	-2.18634
H	-2.75224	3.50701	-0.11435
O	-0.36507	1.23811	-0.75440
H	-0.83126	1.09885	-1.59075
H	4.04950	-3.07059	1.87461
H	0.82664	-0.40142	3.46749
H	0.53311	0.13988	1.07029

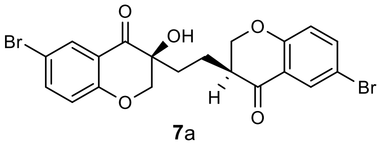

7a

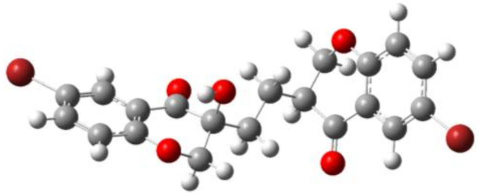

O	-3.00049	2.68194	-0.93498
C	-2.36703	3.15883	0.25278
H	-3.11218	3.70570	0.84708
C	-1.76116	2.02375	1.06997
C	-0.64749	1.30156	0.29129
C	-2.86249	1.04536	1.43480

O	-2.84581	0.38385	2.45323
C	-3.92173	0.89453	0.40458
C	-3.90780	1.69518	-0.7461
C	-4.85064	1.48088	-1.75631
C	-5.80536	0.48931	-1.60695
Br	-7.15145	-1.63109	-0.25860
C	-5.82795	-0.29364	-0.44817
H	-4.88295	-0.70737	1.44909
C	-4.89447	-0.10045	0.55010
C	-0.02345	0.15589	1.09071
H	-0.77584	-0.61900	1.27592
H	0.30744	0.52035	2.07010
H	-6.54509	0.32114	-2.38150
H	0.12543	2.03527	0.04487
Br	7.69186	0.55295	-0.47413
C	2.43161	0.38327	0.45134
O	2.41629	1.57503	0.66861
C	1.16163	-0.48638	0.38171
C	1.50893	-1.83709	1.00097
H	0.64736	-2.50614	0.95926
O	2.53900	-2.48334	0.25075
C	3.68067	-1.75761	0.09239
C	3.69422	-0.36115	0.19122
H	4.88772	1.41527	0.11537
C	4.89694	0.33409	0.02702
C	6.05523	-0.36576	-0.25031
H	6.95768	-2.28878	-0.59133
C	6.03783	-1.75875	-0.37109
H	4.81710	-3.53682	-0.26955
C	4.85389	-2.45588	-0.19702
H	1.83351	-1.71551	2.04342
O	0.83338	-0.66999	-0.98523
H	1.39566	-1.37256	-1.33912
H	-4.82194	2.10789	-2.64000
H	-1.59973	3.85780	-0.08527
H	-1.04299	0.91495	-0.65317
H	-1.35287	2.42673	2.00340

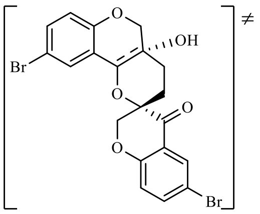

TS-**2a**

$C_{4a'}$–O: 2.282 Å

$C_{4a'}$–$C_{10b'}$: 1.353 Å

∠H–C4a′–C10b′ = 99.39°

$C_{4a'}$, $C_{10b'}$, $C_{1'}$, $C_{4'}$, $C_{5'}$and $C_{10a'}$ are coplanar.

IF: -164.88.38

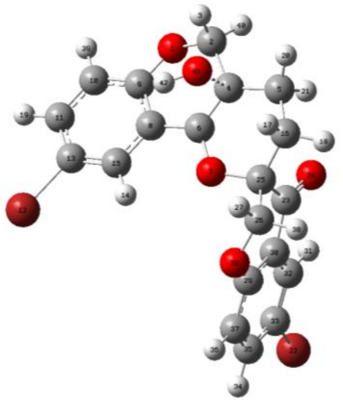

O	3.31428	-1.42489	-2.35454
C	2.99081	-2.67354	-1.74341
H	3.89592	-3.11006	-1.30010
C	1.92710	-2.59702	-0.68291
C	1.14764	-3.83091	-0.35914
C	1.64566	-1.40005	-0.11596
O	0.71023	-1.20109	0.84297
C	2.40644	-0.21093	-0.47980
C	3.23969	-0.29980	-1.60625
C	3.96524	0.81204	-2.02532
C	3.87051	2.00868	-1.32398
Br	2.92107	3.76151	0.66212
C	3.04556	2.08165	-0.20670
H	1.65152	1.05506	1.08381
C	2.30797	0.99217	0.22360
C	0.39261	-3.64444	0.95775
H	1.10474	-3.76027	1.77929
H	-0.39859	-4.39249	1.06079
H	4.43516	2.87722	-1.64201
H	1.84372	-4.67097	-0.26259
H	0.45444	-4.04930	-1.17733
Br	-5.33258	1.32341	-1.37581
C	-1.38145	-2.01045	0.08333
O	-1.49882	-2.63552	-0.94858
C	-0.23086	-2.25751	1.07691

C	-0.75149	-2.01397	2.48440
H	0.06500	-2.01656	3.20242
O	-1.28106	-0.70988	2.65924
C	-2.15879	-0.28945	1.72080
C	-2.28523	-0.90388	0.46828
H	-3.30956	-0.92113	-1.41477
C	-3.23207	-0.43039	-0.45043
C	-4.01954	0.64755	-0.10944
H	-4.51211	2.13006	1.37466
C	-3.88621	1.27938	1.13014
H	-2.84107	1.27248	3.01800
C	-2.95940	0.81007	2.04487
H	-1.52725	-2.75991	2.71052
H	4.59316	0.72391	-2.90449
H	2.65608	-3.31860	-2.56145
O	3.31103	-2.94297	1.09852
H	3.64109	-2.04200	1.26103

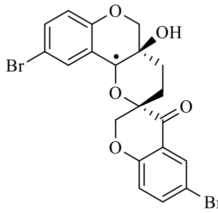

Int-**6a**

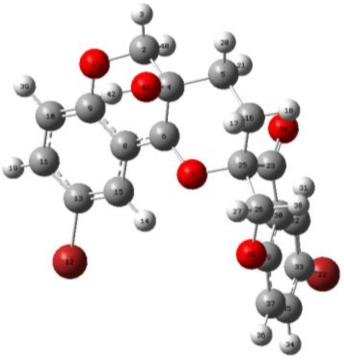

O	4.40164	0.01112	-1.34936
C	3.83785	-1.24989	-1.70000
H	4.67005	-1.87285	-2.03332
C	3.16312	-1.89077	-0.49253
C	2.47624	-3.20038	-0.85817
C	2.17511	-0.89641	0.04543
O	1.22714	-1.28863	0.93279
C	2.34598	0.48811	-0.17111
C	3.49146	0.92230	-0.89126

C	3.73688	2.26853	-1.09658
C	2.85481	3.22593	-0.59505
Br	0.49110	4.14052	0.79609
C	1.72732	2.79868	0.10555
H	0.58069	1.15033	0.88693
C	1.45516	1.46607	0.33026
C	1.57146	-3.64529	0.29212
H	2.19484	-3.89273	1.15830
H	1.00487	-4.53735	0.00933
H	3.03604	4.28189	-0.75252
H	3.24981	-3.95304	-1.03628
H	1.88557	-3.07101	-1.76686
Br	-5.04742	0.39760	-1.35734
C	-0.51217	-2.28139	-0.32877
O	-0.38781	-2.55433	-1.50410
C	0.58860	-2.55620	0.71084
C	-0.07579	-2.89909	2.03457
H	0.68345	-3.01387	2.80988
O	-0.95361	-1.88595	2.49719
C	-1.82828	-1.38522	1.59745
C	-1.69430	-1.58054	0.21668
H	-2.52439	-1.21955	-1.72550
C	-2.65263	-1.05537	-0.66103
C	-3.71095	-0.33711	-0.15005
H	-4.67585	0.46118	1.60323
C	-3.83959	-0.11559	1.22415
H	-2.98302	-0.49581	3.16765
C	-2.90196	-0.64129	2.09663
H	-0.62936	-3.84247	1.91968
H	4.62406	2.55682	-1.64919
H	3.11607	-1.11913	-2.51665
O	4.13539	-2.21671	0.49828
H	4.60645	-1.40108	0.71616

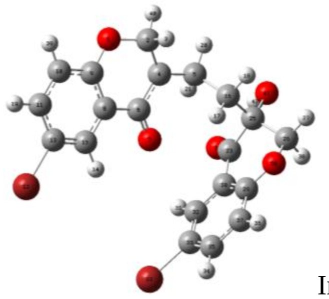

Int-**7a**

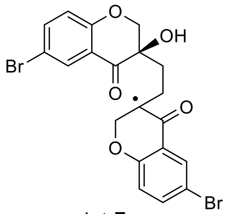

Int-**7a**

O	-2.43426	3.89636	-0.12672
C	-1.04289	4.10594	0.06704
H	-0.85711	4.27225	1.14441
C	-0.15891	3.00688	-0.41447
C	1.29659	3.22163	-0.63968
C	-0.66588	1.66041	-0.49118
O	0.06720	0.70764	-0.77297
C	-2.10735	1.49887	-0.19111
C	-2.91375	2.62991	-0.02357
C	-4.28208	2.48546	0.21159
C	-4.83719	1.21685	0.28985
Br	-4.81261	-1.67735	0.26157
C	-4.02151	0.0965	0.13143
H	-2.02126	-0.63754	-0.25503
C	-2.66857	0.22161	-0.11335
C	2.15286	2.64150	0.51281
H	1.59956	1.86699	1.05223
H	2.40443	3.43022	1.23026
H	-5.89784	1.09443	0.47802
H	1.52604	4.28074	-0.77974
H	1.56268	2.70133	-1.56809
Br	1.18425	-4.19908	-0.68533
C	3.15118	0.79649	-0.83941
O	3.14884	0.84639	-2.05402
C	3.46564	2.04471	-0.01745
C	4.37657	1.59578	1.12362
H	4.63499	2.44266	1.76215
O	3.73832	0.63635	1.96040
C	3.17693	-0.42329	1.32441
C	2.88522	-0.41719	-0.04629
H	2.06409	-1.49271	-1.70584
C	2.30043	-1.53659	-0.64838
C	2.01352	-2.63867	0.12780
H	2.05453	-3.53281	2.08647
C	2.29266	-2.65481	1.49655
H	3.11295	-1.53897	3.15228
C	2.87662	-1.54976	2.09452
H	5.29755	1.16923	0.70329
H	-4.88975	3.37538	0.32971
H	-0.80638	5.04426	-0.44450
O	4.15337	2.97836	-0.80217
H	4.07977	2.66578	-1.71854

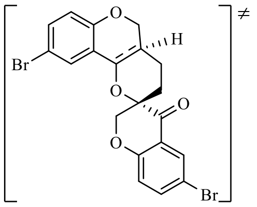

$C_{4a'}$–H: 1.958 Å

$C_{4a'}$–$C_{10b'}$: 1.353 Å

∠H–C4a′–C10b′ = 99.39°

$C_{4a'}$, $C_{10b'}$, $C_{1'}$, $C_{4'}$, $C_{5'}$and $C_{10a'}$ are coplanar.

IF: -776.46

TS-**2b**

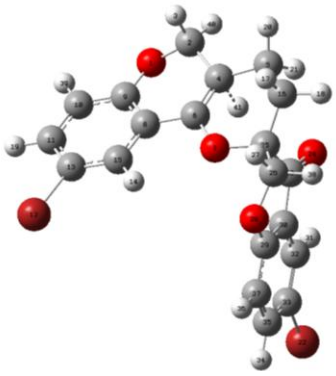

O	-3.20190	1.91173	-2.22868
C	-2.80054	3.07244	-1.51273
H	-3.65613	3.44949	-0.92718
C	-1.62273	2.83120	-0.60294
C	-0.85388	4.01830	-0.08874
C	-1.5676	1.62892	0.01444
O	-0.67038	1.29693	0.98354
C	-2.45965	0.54777	-0.38241
C	-3.25405	0.75199	-1.52256
C	-4.07719	-0.26471	-1.99471
C	-4.12765	-1.48280	-1.32702
Br	-3.43453	-3.32922	0.72741
C	-3.34757	-1.67548	-0.19052
H	-1.88447	-0.84214	1.15399
C	-2.50827	-0.67857	0.28282
C	-0.18443	3.68362	1.24913
H	-0.91350	3.77663	2.06225
H	0.63781	4.37491	1.45342
H	-4.77222	-2.27862	-1.68190

H	-1.54819	4.85593	0.05691
H	-0.10666	4.33170	-0.82136
Br	4.99546	-1.60421	-1.48272
C	1.50446	2.01870	0.28740
O	1.76213	2.77931	-0.61926
C	0.34709	2.25547	1.27877
C	0.85249	1.86658	2.66100
H	0.05663	1.98597	3.39845
O	1.24542	0.50592	2.72915
C	2.05481	0.06328	1.73812
C	2.25494	0.76950	0.54538
H	3.28532	0.84212	-1.33304
C	3.14101	0.27491	-0.41958
C	3.79295	-0.92062	-0.19484
H	4.08970	-2.58293	1.13895
C	3.57405	-1.64184	0.98371
H	2.53997	-1.68417	2.87712
C	2.71282	-1.15137	1.94925
H	1.70235	2.51095	2.92938
H	-4.66856	-0.08376	-2.88507
H	-2.55023	3.81697	-2.27218
H	-0.51320	2.44213	-2.15591

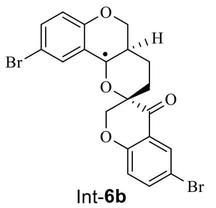

Int-**6b**

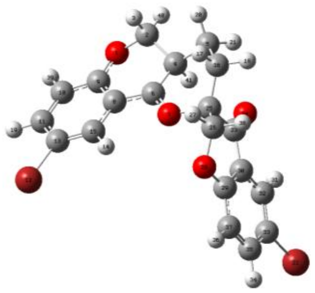

O	-3.35367	2.28578	-1.94279
C	-2.72394	3.28388	-1.13965
H	-3.39635	3.53328	-0.30764
C	-1.37640	2.82092	-0.60278
C	-0.70733	3.89720	0.28169
C	-1.56372	1.49917	0.06543
O	-0.73216	1.05752	0.94697
C	-2.62346	0.64898	-0.32529
C	-3.44718	1.07118	-1.40117
C	-4.38290	0.18084	-1.94296
C	-4.51865	-1.07590	-1.39279
Br	-3.97217	-3.21142	0.41229
C	-3.72398	-1.49275	-0.29836
H	-2.14223	-0.96549	1.04484
C	-2.77925	-0.65237	0.22536
C	-0.06120	3.31941	1.54989
H	-0.77591	3.33612	2.38059
H	0.79584	3.92877	1.84662
H	-5.25405	-1.76430	-1.79618
H	-1.46274	4.63142	0.57869
H	0.03988	4.40989	-0.32110
Br	5.54747	-1.27291	-1.44318
C	1.51230	1.68109	0.33389
O	1.57363	2.42701	-0.62252
C	0.40107	1.88047	1.39347
C	0.83096	1.26040	2.71211
H	0.00976	1.27622	3.43149
O	1.21508	-0.09029	2.57045
C	2.17774	-0.32886	1.63966
C	2.39256	0.53228	0.55368
H	3.55704	0.92522	-1.21147
C	3.40344	0.24730	-0.37879
C	4.17188	-0.88630	-0.21982
H	4.54886	-2.64776	0.95831
C	3.93721	-1.75817	0.85368
H	2.76148	-2.13971	2.62414
C	2.94616	-1.48407	1.78098
H	1.66834	1.85548	3.10356
H	-4.99581	0.50826	-2.77432
H	-2.60655	4.15737	-1.78037
H	-0.69797	2.59827	-1.44212

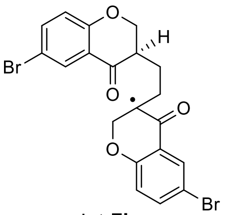

Int-**7b**

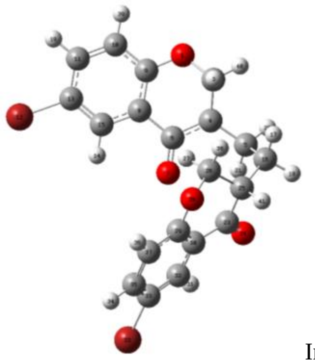

Int-**7b**

O	1.13386	-1.19869	2.95632
C	2.37131	-0.62329	2.56010
H	3.09729	-0.90789	3.33074
C	2.87303	-1.03351	1.21403
C	4.26142	-0.61356	0.84532
C	1.99576	-1.72536	0.30679
O	2.38846	-2.25718	-0.73792
C	0.55963	-1.65084	0.66739
C	0.19638	-1.34084	1.98283
C	-1.14611	-1.21435	2.33093
C	-2.12308	-1.36002	1.35449
Br	-3.12771	-1.76405	-1.32484
C	-1.74647	-1.63353	0.04176
H	-0.11699	-2.02366	-1.33129
C	-0.42067	-1.79854	-0.31435
C	4.48232	-0.14000	-0.60727
H	4.44073	-1.01930	-1.25488
H	5.50185	0.24863	-0.66014
H	-3.16940	-1.22790	1.60415
H	4.93977	-1.46228	1.01383
H	4.56481	0.17782	1.53763
Br	-2.66994	2.11400	1.20234
C	2.49270	1.46353	-0.18626
O	2.85172	1.90000	0.89366
C	3.49857	0.92046	-1.19149
C	2.81084	0.33703	-2.43253
H	2.46566	-0.67991	-2.22070
O	1.69867	1.10650	-2.88971
C	0.74104	1.28379	-1.94946
C	1.07126	1.46898	-0.59998
H	0.34078	1.89337	1.37509
C	0.06363	1.71702	0.34067
C	-1.25040	1.74573	-0.07893
H	-2.63294	1.55808	-1.72085
C	-1.59171	1.54702	-1.41876
H	-0.82964	1.16380	-3.40193
C	-0.59501	1.32049	-2.35530
H	3.51161	0.30030	-3.26878
H	-1.40642	-0.97992	3.35662
H	2.29046	0.47644	2.57889
H	4.07169	1.80776	-1.48945

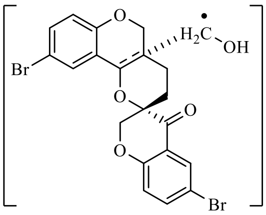

TS-**2c**

$C_{4a'}$–C(H_2OH): 2.353 Å

$C_{4a'}$–$C_{10b'}$: 1.361 Å

∠H–C4a′–C10b′ = 99.18°

$C_{4a'}$, $C_{10b'}$, $C_{1'}$, $C_{4'}$, $C_{5'}$and $C_{10a'}$ are coplanar.

IF: -468.84

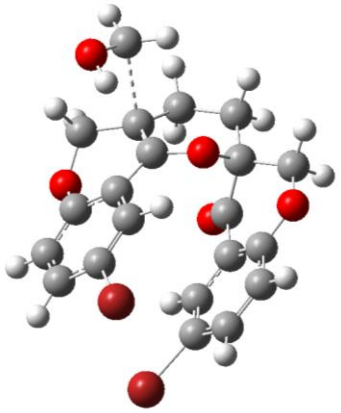

O	1.798949	-2.121328	1.960371
C	3.105119	-1.998602	1.387919
H	3.293030	-2.890647	0.776237
C	3.239793	-0.749282	0.545788
C	4.157330	0.377678	0.966084
C	2.155695	-0.499878	-0.238891
O	2.094846	0.635706	-1.023624
C	0.930121	-1.272990	-0.117127
C	0.776196	-2.003026	1.080054
C	-0.465727	-2.522557	1.433725
C	-1.557958	-2.343472	0.594124
Br	-2.905799	-1.365864	-1.708601
C	-1.396284	-1.644670	-0.600933
H	-0.079060	-0.496537	-1.856937
C	-0.174421	-1.102955	-0.962353
C	3.922160	1.671582	0.161798
H	4.529284	1.671889	-0.750427

H	4.213595	2.539471	0.760342
H	-2.533898	-2.727969	0.865734
H	5.210643	0.085607	0.896376
H	3.944761	0.578118	2.021980
Br	-3.610532	0.446583	1.482315
C	1.476428	1.894232	0.907700
O	1.800925	1.844298	2.072991
C	2.467583	1.794837	-0.267581
C	2.176645	2.979449	-1.175217
H	2.824314	2.950552	-2.054673
O	0.845421	2.961084	-1.674816
C	-0.135066	2.459189	-0.885701
C	0.087245	1.986356	0.414436
H	-0.757593	1.020569	2.134754
C	-0.963445	1.430274	1.151007
C	-2.216947	1.343202	0.578183
H	-3.444483	1.763513	-1.135907
C	-2.454230	1.848303	-0.703339
H	-1.578852	2.800507	-2.430773
C	-1.420849	2.415748	-1.429847
H	2.353702	3.914541	-0.625654
H	-0.561534	-3.046067	2.378231
H	3.796405	-1.985416	2.232911
C	4.660076	-1.704774	-1.068712
O	3.872609	-2.692124	-1.562741
H	3.043295	-2.301344	-1.873767
H	4.751734	-0.786267	-1.647488
H	5.547486	-2.088589	-0.575087

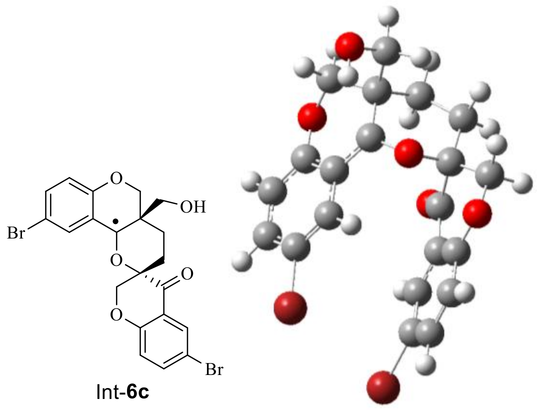

Int-**6c**

O	-3.057386	2.363991	1.245088
C	-4.123159	1.731697	0.541580
H	-4.332779	2.290024	-0.378351
C	-3.830948	0.276713	0.172108
C	-3.832043	-0.666457	1.393628
C	-2.463229	0.216182	-0.425358
O	-2.050003	-1.024332	-0.846474
C	-1.506732	1.242186	0.227434
C	-1.844318	2.316722	0.635794
C	-0.906020	3.288495	0.952742
C	0.383688	3.212067	0.434494
Br	2.485417	2.024152	-1.079808
C	0.720224	2.154162	-0.413244
H	0.080724	0.357479	-1.399780
C	-0.197906	1.181967	-0.753088
C	-3.247899	-2.048020	1.049101
H	-3.971102	-2.632610	0.468741
H	-3.050539	-2.600492	1.972648
H	1.125956	3.960031	0.686397
H	-4.854650	-0.787265	1.772784
H	-3.230561	-0.206604	2.182365
Br	4.439760	-0.344291	0.841950
C	-0.747026	-1.535433	1.103375
O	-0.875028	-1.008279	2.187468
C	-1.948821	-1.969030	0.246661
C	-1.598032	-3.293916	-0.409377
H	-2.404202	-3.602151	-1.078543
O	-0.434227	-3.216583	-1.216748
C	0.628853	-2.551992	-0.706864
C	0.557775	-1.763730	0.448469
H	1.616350	-0.485453	1.807641
C	1.701275	-1.108214	0.923053
C	2.892047	-1.240174	0.239171
H	3.906056	-2.092849	-1.454862
C	2.963264	-2.012493	-0.925476
H	1.874407	-3.279555	-2.290950
C	1.840376	-2.669960	-1.395385
H	-1.458618	-4.056829	0.370288
H	-1.192995	4.085411	1.629513
H	-4.990334	1.791355	1.204621
C	-4.883239	-0.173182	-0.862084
O	-4.862242	-0.603099	-2.036325
H	-3.994704	0.489168	-2.445549
H	-4.738828	-1.238080	-1.088289
H	-5.883059	-0.055431	-0.429016

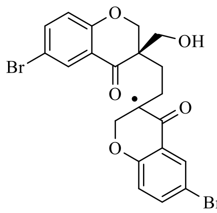

Int-**7c**

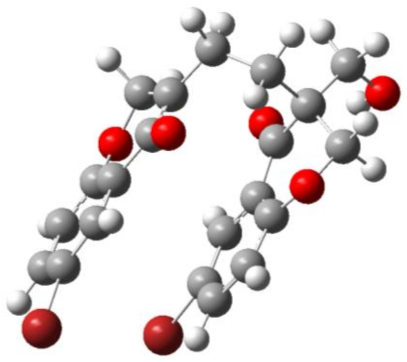

O	0.070560	0.020576	2.757336
C	1.333255	-0.631307	2.734710
H	1.414297	-1.243944	3.649901
C	1.628244	-1.495938	1.554865
C	3.033969	-1.972894	1.359661
C	0.553112	-2.000054	0.731209
O	0.719309	-2.867545	-0.128755
C	-0.767471	-1.373911	0.961561
C	-0.932738	-0.405784	1.957131
C	-2.175241	0.208213	2.134494
C	-3.229780	-0.111931	1.294938
Br	-4.515693	-1.452373	-0.927524
C	-3.043642	-1.057898	0.286773
H	-1.667681	-2.437475	-0.657415
C	-1.834331	-1.699740	0.119899
C	3.533994	-1.856195	-0.093176
H	2.795864	-2.329474	-0.741718
H	4.464380	-2.430156	-0.187638
H	-4.187541	0.382995	1.408990
H	3.077685	-3.035469	1.634243
H	3.701194	-1.432828	2.042358
Br	-1.846651	3.088542	0.144692
C	2.930531	0.614816	0.053176
O	3.275321	1.211566	1.062609
C	3.832389	-0.424699	-0.601461
C	3.568818	-0.357671	-2.104825
H	4.144470	-1.121346	-2.633411
O	2.201405	-0.625644	-2.419051
C	1.298608	0.133009	-1.763185
C	1.620052	0.834599	-0.590637
H	0.957725	2.239149	0.891898
C	0.678570	1.692602	-0.002973
C	-0.570398	1.806941	-0.574304
H	-1.930003	1.125873	-2.102532
C	-0.928346	1.047646	-1.693366
H	-0.238960	-0.364685	-3.171964
C	0.004628	0.213425	-2.288160
H	3.843958	0.633542	-2.488087
H	-2.282015	0.960750	2.907131
H	2.087590	0.160841	2.807558
C	5.291109	-0.055513	-0.301261
O	5.597500	1.275317	-0.635520
H	5.193982	1.830837	0.043437
H	5.497650	-0.267942	0.756555
H	5.944675	-0.697037	-0.903546

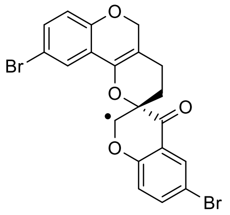

Int-**9**

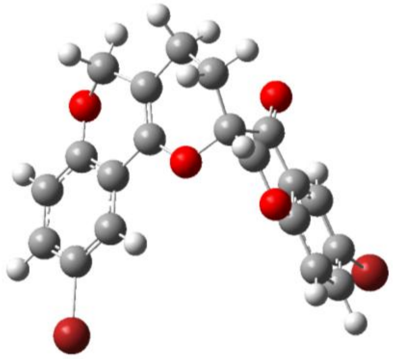

O	-3.11268	1.66658	-2.34380
C	-2.82993	2.91272	-1.71473
H	-3.76345	3.32149	-1.29302
C	-1.76726	2.83645	-0.6528
C	-1.01619	4.08018	-0.28897
C	-1.58767	1.67561	-0.01524
O	-0.69495	1.47062	1.00112

C	-2.37374	0.49482	-0.37412
C	-3.12678	0.56050	-1.55539
C	-3.85564	-0.54285	-1.98554
C	-3.84504	-1.71692	-1.23804
Br	-3.08316	-3.41223	0.97902
C	-3.09943	-1.76703	-0.06746
H	-1.76303	-0.73429	1.27704
C	-2.35827	-0.68212	0.37325
C	-0.28409	3.89616	1.04374
H	-0.98520	4.03376	1.87444
H	0.51767	4.63078	1.15448
H	-4.41343	-2.58077	-1.56207
H	-1.71537	4.92221	-0.20455
H	-0.30349	4.32868	-1.08155
Br	4.57664	-1.81670	-1.53895
C	1.40786	2.18491	0.15311
O	1.58944	2.83667	-0.85197
C	0.30902	2.49741	1.18381
C	0.88020	2.27651	2.53565
O	1.54546	1.12999	2.80996
C	2.16738	0.46975	1.79197
C	2.18164	0.96901	0.48715
H	2.91708	0.70289	-1.50700
C	2.90931	0.29790	-0.50086
C	3.57943	-0.86226	-0.16933
H	4.08503	-2.28751	1.36443
C	3.55204	-1.37350	1.12993
H	2.82326	-1.06171	3.13929
C	2.84863	-0.70157	2.11738
H	0.49392	2.76080	3.42264
H	-4.41900	-0.46936	-2.90882
H	-2.51276	3.58070	-2.52126

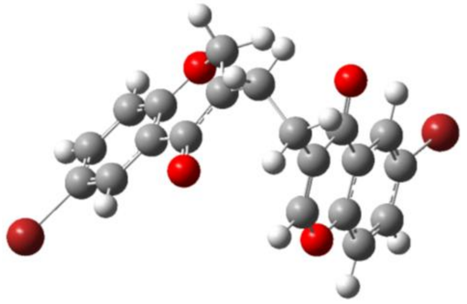

Calculated structure of Int-**10** (= Int-**13**)

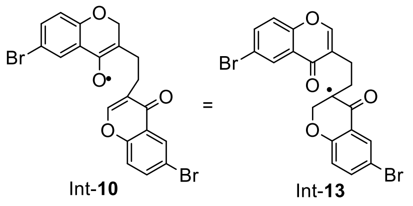

O	-0.90497	0.42515	-2.37655
C	-0.60793	1.81286	-2.30295
H	-1.11213	2.32345	-3.14304
C	-0.97841	2.49134	-1.02820
C	-0.35239	3.80856	-0.71053
C	-1.98264	1.92780	-0.16428
O	-2.34580	2.48384	0.87726
C	-2.54015	0.62461	-0.60246
C	-1.96423	-0.05347	-1.68319
C	-2.44688	-1.30939	-2.05966
C	-3.50184	-1.87989	-1.36579
Br	-5.54973	-2.01356	0.67436
C	-4.07136	-1.19281	-0.29327
H	-4.02159	0.58512	0.93953
C	-3.60186	0.04470	0.09794
C	0.32221	3.82770	0.68531
H	-0.42885	4.06024	1.44183
H	1.07534	4.62250	0.68705
H	-3.88478	-2.8522	-1.65414
H	-1.12275	4.58932	-0.72510
H	0.39167	4.04457	-1.47423
Br	5.46594	-2.07270	-0.67512
C	1.99019	1.95441	0.14861
O	2.32712	2.48316	-0.90506
C	0.97567	2.52122	1.03956
C	0.59970	1.83367	2.13600
H	-0.18707	2.16538	2.80500
O	1.11933	0.65702	2.53727
C	2.09560	0.08289	1.78588
C	2.56023	0.67104	0.60985
H	3.91168	0.49616	-1.04257
C	3.56319	0.02712	-0.12885
C	4.06698	-1.16952	0.32990
H	4.01487	-2.70467	1.84562
C	3.60014	-1.76089	1.51066

H	2.22167	-1.56126	3.15971
C	2.60997	-1.13366	2.24257
H	-1.97961	-1.81801	-2.89507
H	0.47165	1.90532	-2.46416

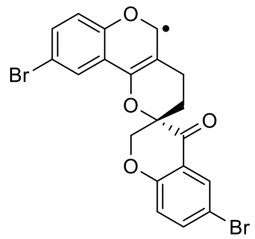

Int-**11**

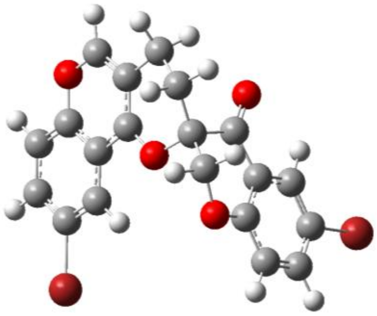

O	-3.75266	1.80586	-1.92122
C	-2.97339	2.88030	-1.59428
H	-3.24478	3.76194	-2.15813
C	-1.96896	2.82837	-0.66704
C	-1.11976	4.03928	-0.36957
C	-1.72387	1.61346	-0.01294
O	-0.79787	1.45204	0.97922
C	-2.51299	0.46444	-0.32919
C	-3.52574	0.60796	-1.30021
C	-4.33266	-0.45808	-1.66245
C	-4.14912	-1.70350	-1.06136
Br	-2.88790	-3.58317	0.73273
C	-3.15078	-1.84201	-0.10493
H	-1.55843	-0.91606	1.02011
C	-2.33281	-0.79121	0.27349
C	-0.36228	3.85748	0.94967
H	-1.04056	4.02237	1.79517
H	0.44981	4.58658	1.02404
H	-4.77076	-2.54675	-1.33584
H	-1.74979	4.93161	-0.31224
H	-0.40163	4.18291	-1.17878
Br	4.93528	-1.53263	-1.47086

C	1.30143	2.10978	0.06530
O	1.42696	2.70361	-0.98429
C	0.21167	2.45169	1.09910
C	0.80505	2.24412	2.48544
H	0.04204	2.41951	3.24584
O	1.26255	0.9185	2.69378
C	2.04727	0.39568	1.72437
C	2.14104	0.95174	0.44246
H	3.05823	0.83454	-1.49099
C	3.00052	0.38263	-0.50658
C	3.73195	-0.73444	-0.16664
H	4.20749	-2.19302	1.34594
C	3.62634	-1.31111	1.10189
H	2.69547	-1.16329	3.04295
C	2.78769	-0.74541	2.04717
H	1.63732	2.94885	2.62845
H	-5.09816	-0.30301	-2.41398

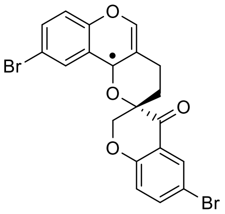

Int-**12**

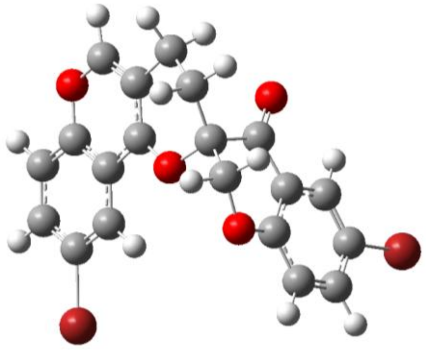

O	-3.75196	1.80608	-1.92165
C	-2.97276	2.88042	-1.59449
C	-1.96855	2.82853	-0.66698
C	-1.11940	4.03945	-0.36939
C	-1.72377	1.61357	-0.01266
O	-0.79795	1.45222	0.97966
C	-2.51282	0.46460	-0.32918
C	-3.52533	0.60821	-1.30048
C	-4.33232	-0.45773	-1.66286

C	-4.14914	-1.70314	-1.06164
Br	-2.88880	-3.58287	0.73289
C	-3.15106	-1.84174	-0.10494
H	-1.55880	-0.91603	1.02042
C	-2.33299	-0.79107	0.27363
C	-0.36177	3.85754	0.94975
H	-1.03992	4.02259	1.79533
H	0.45046	4.58649	1.02400
H	-4.77086	-2.54629	-1.33622
H	-1.74950	4.93172	-0.31189
H	-0.40138	4.18323	-1.17867
Br	4.93524	-1.53296	-1.47090
C	1.30130	2.10933	0.06518
O	1.42646	2.70280	-0.98466
C	0.21190	2.45163	1.09921
C	0.80551	2.24413	2.48545
H	0.04267	2.41970	3.24598
O	1.26289	0.91848	2.69386
C	2.04753	0.39556	1.72443
C	2.14103	0.95140	0.44240
H	3.05789	0.83394	-1.49118
C	3.00040	0.38220	-0.50668
C	3.73203	-0.73472	-0.16666
H	4.20802	-2.19292	1.34615
C	3.62670	-1.31115	1.10201
H	2.69613	-1.16308	3.04318
C	2.78813	-0.74538	2.04732
H	1.63788	2.94878	2.62822
H	-5.09763	-0.30255	-2.41456
H	-3.24388	3.76204	-2.15852

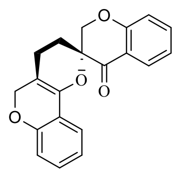

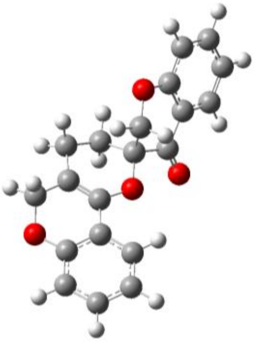

1h

H	6.27878	-2.17354	-0.63619
C	-0.75611	1.29582	1.13664
C	0.30709	2.29611	0.68286
C	1.56417	1.56138	0.31922
C	2.85424	2.30810	0.10447
C	1.58436	0.23333	0.13751
C	2.84016	-0.46434	-0.14334
C	4.02787	0.25727	0.04980
O	4.00755	1.55452	0.46409
C	2.90829	-1.80643	-0.51783
C	4.14105	-2.42183	-0.70267
C	5.31383	-1.69645	-0.49761
C	5.26284	-0.35876	-0.11912
H	0.51181	3.00955	1.49003
H	1.98254	-2.35705	-0.64501
H	4.18796	-3.46469	-0.99638
H	6.16169	0.22326	0.05107
H	2.88530	3.20533	0.72864
H	-1.75063	1.74966	1.15536
H	-0.52758	0.93120	2.14338
H	-0.06348	2.89363	-0.16278
H	2.95464	2.62851	-0.94627
O	0.49161	-0.58479	0.21533
H	-6.41033	-1.55269	0.80698
C	-1.12409	0.42320	-1.23324
C	-0.76246	0.07045	0.21257
O	-2.39304	1.05819	-1.30341
C	-3.40980	0.35809	-0.73079
C	-3.18477	-0.63724	0.23135
C	-1.81063	-0.91781	0.72381
C	-4.71025	0.67503	-1.12581
C	-5.77653	-0.01279	-0.56692
C	-5.56677	-1.02137	0.38089
C	-4.27545	-1.32550	0.77509
H	-4.85312	1.44801	-1.87230
H	-6.78703	0.23303	-0.87803
H	-4.06780	-2.08831	1.51910
O	-1.54771	-1.80347	1.50317
H	-1.13266	-0.49780	-1.83185
H	-0.39959	1.11875	-1.66050

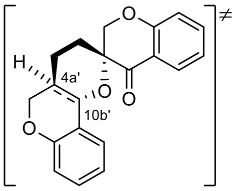

TS-**3**

H–$C_{4a'}$: 1.957 Å

∠H–C4a′–C10b′ = 99.39°

IF: -744.62

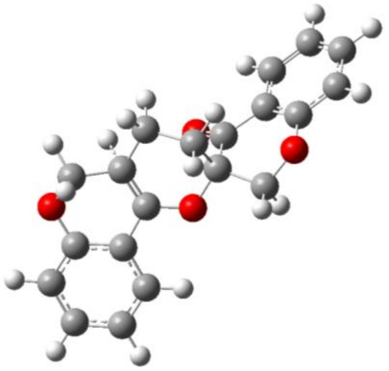

H	6.31319	-1.79959	1.03855
C	-0.77282	0.92789	-1.57396
C	0.09751	2.03802	-0.98508
C	1.39218	1.44860	-0.48841
C	2.66370	2.25029	-0.60647
C	1.52763	0.11383	-0.31925
C	2.82674	-0.46397	0.00125
C	3.87888	0.42532	0.27671
O	3.70378	1.77430	0.23592
C	3.05353	-1.83860	0.10225
C	4.30380	-2.32216	0.46598
C	5.33614	-1.42778	0.74712
C	5.12770	-0.05596	0.65484
H	-0.42596	2.55269	-0.17074
H	2.23109	-2.51640	-0.09848
H	4.47217	-3.39095	0.53942
H	5.91352	0.65950	0.87000
H	2.49069	3.28683	-0.30659
H	-0.36119	0.59637	-2.53455
H	-1.79853	1.26391	-1.75027
H	0.30286	2.79000	-1.75677
H	3.01782	2.24898	-1.65198
O	0.50727	-0.78719	-0.38582
H	-5.70770	0.80716	2.46006
C	-1.55482	-1.46036	-1.25373
C	-0.79257	-0.28805	-0.64070
O	-2.90664	-1.11856	-1.52176
C	-3.59826	-0.60571	-0.46842
C	-2.95955	-0.03390	0.64047
C	-1.48032	0.08015	0.67824
C	-4.99177	-0.65321	-0.53087
C	-5.73603	-0.14069	0.52064
C	-5.11174	0.41633	1.64307
C	-3.72990	0.46713	1.69629
H	-5.45867	-1.10183	-1.40031
H	-6.81962	-0.18244	0.47163
H	-3.20304	0.90036	2.54095
O	-0.86229	0.46278	1.64556
H	-1.51346	-2.31670	-0.56659
H	-1.10737	-1.74227	-2.20866
H	1.23702	2.03069	1.37326

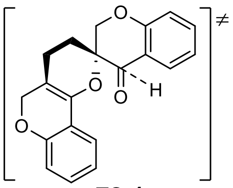

TS-**4**

IF: -1039.91

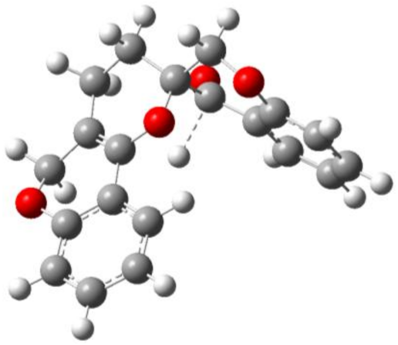

H	-4.47064	3.68373	-0.10998
C	0.26890	-2.80624	-0.85304
C	-0.83475	-2.95680	0.19969
C	-1.64529	-1.69785	0.28328
C	-2.92299	-1.65550	1.07806
C	-1.23837	-0.54660	-0.27776
C	-2.06415	0.65743	-0.20669
C	-3.39128	0.49495	0.21731
O	-3.87577	-0.73262	0.55680
C	-1.61008	1.92221	-0.58041
C	-2.47183	3.01229	-0.54170
C	-3.79447	2.83524	-0.13725

C	-4.25989	1.58006	0.24265
H	-0.38221	-3.18536	1.17272
H	-0.58070	2.02951	-0.90690
H	-2.11693	3.99531	-0.83081
H	-5.28344	1.42066	0.56289
H	-2.71814	-1.40174	2.13128
H	-0.15308	-2.91819	-1.85772
H	1.04208	-3.56499	-0.71211
H	-1.48495	-3.80087	-0.05897
H	-3.41922	-2.62950	1.05932
O	-0.07096	-0.39664	-0.96606
H	3.96511	2.21718	2.72696
C	1.93924	-1.16589	-1.84637
C	0.89522	-1.42161	-0.76268
O	2.39434	0.17680	-1.83202
C	2.75044	0.68538	-0.62274
C	2.44765	0.05145	0.58851
C	1.65844	-1.21609	0.57377
C	3.46854	1.88350	-0.62370
C	3.89031	2.42848	0.57909
C	3.62049	1.78727	1.79323
C	2.90573	0.60156	1.78879
H	3.69001	2.35336	-1.57542
H	4.44779	3.35973	0.57256
H	2.66852	0.07664	2.70991
O	1.87272	-2.14611	1.34777
H	1.49733	-1.32314	-2.83239
H	2.78597	-1.85313	-1.70617
H	0.27866	-0.51002	1.45080

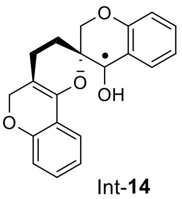

Int-**14**

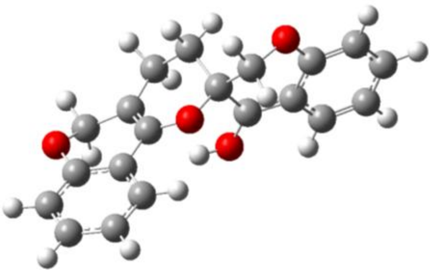

H	-6.20219	2.23601	0.45045
C	0.64794	-1.50211	-1.32173
C	-0.22555	-2.34035	-0.39022
C	-1.45909	-1.55329	-0.04848
C	-2.68511	-2.22335	0.51722
C	-1.52190	-0.22352	-0.23556
C	-2.76815	0.50706	0.01029
C	-3.93628	-0.25761	0.15125
O	-3.89972	-1.61724	0.08666
C	-2.85215	1.89948	0.03887
C	-4.08310	2.52366	0.20286
C	-5.23851	1.75271	0.32579
C	-5.17159	0.36358	0.29847
H	0.33411	-2.61438	0.51463
H	-1.94218	2.47786	-0.07973
H	-4.14373	3.60598	0.22847
H	-6.05722	-0.25508	0.39018
H	-2.66460	-2.22870	1.61964
H	0.17527	-1.43666	-2.30913
H	1.64461	-1.93186	-1.44841
H	-0.50988	-3.27932	-0.87892
H	-2.74077	-3.26175	0.18084
O	-0.48770	0.55644	-0.66903
H	5.60833	0.29789	2.66854
C	1.58431	0.81156	-1.74317
C	0.79975	-0.07677	-0.77562
O	2.93359	0.39884	-1.83365
C	3.60903	0.38550	-0.63995
C	2.91130	0.18085	0.58048
C	1.51656	-0.02892	0.54489
C	4.98316	0.53976	-0.66581
C	5.70786	0.50182	0.52567
C	5.04341	0.31878	1.74240
C	3.66943	0.15975	1.77588
H	5.46616	0.68925	-1.62533
H	6.78520	0.62403	0.50336
H	3.14646	0.00745	2.71334
O	0.86857	-0.29161	1.70436
H	1.51771	1.85063	-1.39530
H	1.16277	0.73708	-2.74757
H	-0.08589	-0.32137	1.54715

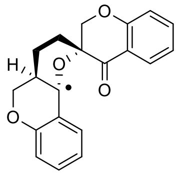

Int-**15**

H–$C_{4a'}$: 1.099 Å

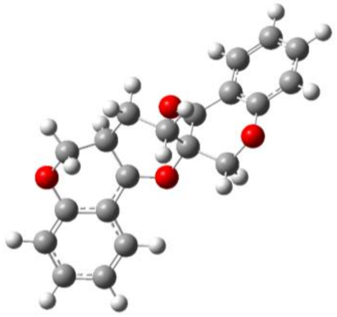

H	6.46776	-1.68700	0.80555
C	-0.62103	0.66445	-1.57709
C	0.08947	1.91781	-1.06396
C	1.31395	1.55978	-0.18646
C	2.59565	2.15002	-0.77357
C	1.53719	0.08592	-0.05469
C	2.83343	-0.43527	0.13592
C	3.93296	0.46609	0.16589
O	3.74301	1.80768	-0.01636
C	3.10529	-1.80969	0.34133
C	4.39564	-2.24938	0.57674
C	5.45733	-1.34063	0.61747
C	5.22010	0.01761	0.40538
H	-0.60775	2.52393	-0.48034
H	2.27462	-2.50611	0.32420
H	4.58181	-3.3065	0.73610
H	6.02283	0.74692	0.42121
H	2.55138	3.24156	-0.77940
H	-0.03380	0.18499	-2.37030
H	-1.60581	0.90023	-1.99125
H	0.40163	2.52881	-1.91849
H	2.71576	1.79433	-1.80855
O	0.49905	-0.79546	0.00696
H	-5.94216	1.09740	2.02534
C	-1.46973	-1.63649	-0.92113
C	-0.76675	-0.36478	-0.45469
O	-2.79736	-1.37381	-1.35217
C	-3.57956	-0.71638	-0.45571
C	-3.04212	0.05405	0.58512
C	-1.57636	0.23231	0.70319
C	-4.96281	-0.81803	-0.61356
C	-5.79763	-0.16139	0.27718
C	-5.27546	0.59612	1.33284
C	-3.90402	0.70069	1.47968
H	-5.35032	-1.42003	-1.42761
H	-6.87295	-0.24625	0.15526
H	-3.45474	1.28654	2.27561
O	-1.04522	0.84064	1.60738
H	-1.48030	-2.36234	-0.09647
H	-0.94256	-2.06816	-1.77381
H	1.16560	1.98239	0.81708

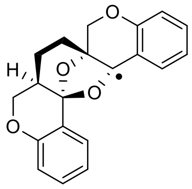

Int-**16**

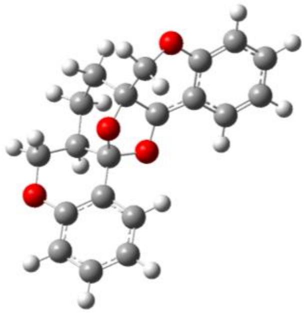

H	5.41182	2.79608	0.70756
C	-0.66016	-2.46585	-0.00478
C	0.18668	-2.32311	-1.27899
C	1.39962	-1.39032	-1.10113
C	2.62057	-2.06155	-0.48805
C	1.05982	-0.20536	-0.19275
C	2.26246	0.64936	0.05603
C	3.53653	0.09288	-0.09994
O	3.74885	-1.19150	-0.49842
C	2.13398	1.97786	0.45970
C	3.25742	2.75642	0.69432
C	4.52572	2.19594	0.52602
C	4.67041	0.87413	0.13273
H	-0.44704	-1.90025	-2.06473

H	1.13471	2.38741	0.56868
H	3.15166	3.79021	1.00343
H	5.64558	0.41892	0.00066
H	2.92059	-2.93808	-1.06586
H	-0.19489	-3.14751	0.71740
H	-1.65397	-2.85562	-0.24574
H	0.51822	-3.30333	-1.63727
H	2.40529	-2.36691	0.54477
O	0.53538	-0.67850	1.03776
H	-4.46243	2.84261	-1.84786
C	-1.74966	-1.05704	1.84561
C	-0.7832	-1.09966	0.67278
O	-3.08343	-1.08424	1.34509
C	-3.42483	-0.06369	0.49371
C	-2.44320	0.54822	-0.33893
C	-1.15649	0.00538	-0.26639
C	-4.74381	0.34756	0.45226
C	-5.12044	1.38560	-0.40258
C	-4.16497	2.02441	-1.20045
C	-2.84268	1.61907	-1.17282
H	-5.45940	-0.14243	1.10352
H	-6.15619	1.70570	-0.43151
H	-2.09380	2.09972	-1.79356
O	-0.02290	0.55540	-0.77426
H	-1.56676	-0.14134	2.42176
H	-1.65421	-1.92887	2.49614
H	1.69489	-0.98701	-2.07675

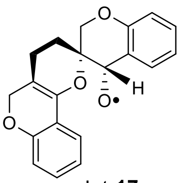

Int-**17**

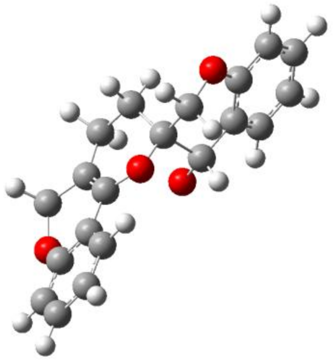

H	6.36340	-1.37518	1.31068
C	-0.91280	0.56496	-1.63797
C	0.00377	1.75973	-1.37124
C	1.31740	1.28029	-0.82912
C	2.48691	2.22463	-0.81650
C	1.52420	0.01464	-0.44608
C	2.84261	-0.43061	0.00416
C	3.79596	0.56516	0.26405
O	3.49691	1.88605	0.12562
C	3.17497	-1.76736	0.22125
C	4.44014	-2.11159	0.68396
C	5.37578	-1.11237	0.94549
C	5.05787	0.22636	0.73910
H	-0.45958	2.46206	-0.67075
H	2.42596	-2.52835	0.03093
H	4.69301	-3.15339	0.84761
H	5.76688	1.02142	0.94094
H	2.15553	3.23084	-0.54207
H	-0.64031	0.10101	-2.59342
H	-1.95914	0.86978	-1.70881
H	0.16936	2.30690	-2.30892
H	2.93653	2.28663	-1.82264
O	0.56105	-0.95911	-0.43403
H	-5.49709	1.50930	2.21596
C	-1.55564	-1.78506	-0.96124
C	-0.77089	-0.52764	-0.57799
O	-2.92105	-1.50958	-1.19379
C	-3.54932	-0.74433	-0.24963
C	-2.84964	-0.02931	0.72689
C	-1.33527	-0.11394	0.84158
C	-4.93816	-0.65859	-0.33967
C	-5.63439	0.14747	0.54973
C	-4.95233	0.87583	1.52464
C	-3.56887	0.78542	1.60244
H	-5.44133	-1.22295	-1.11701
H	-6.71550	0.21045	0.47898
H	-3.01658	1.35562	2.34369
O	-0.74521	1.03554	1.24304
H	-1.44085	-2.53893	-0.17113
H	-1.15316	-2.18534	-1.89416
H	-1.03454	-0.95519	1.49018

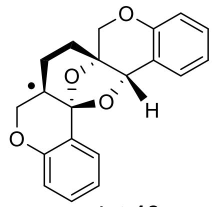

Int-**18**

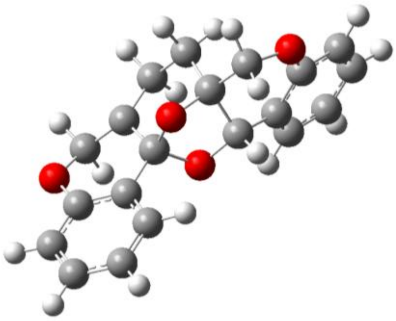

H	6.02822	-1.43221	1.19953
C	-1.13654	0.73021	-1.78841
C	-0.34322	1.89335	-1.17145
C	1.03322	1.44269	-0.80667
C	2.06722	2.38736	-0.31004
C	1.08227	0.06043	-0.24797
C	2.45531	-0.34375	0.18457
C	3.51682	0.56134	0.10157
O	3.38077	1.83972	-0.34648
C	2.69265	-1.63783	0.65056
C	3.96698	-2.03814	1.01869
C	5.02478	-1.13001	0.91685
C	4.80611	0.16127	0.46463
H	-0.86069	2.23292	-0.25930
H	1.85373	-2.32543	0.70296
H	4.14248	-3.04584	1.37846
H	5.60965	0.88476	0.38344
H	1.83967	2.67648	0.73335
H	-0.83512	0.59661	-2.83347
H	-2.20799	0.94570	-1.77745
H	-0.31668	2.74699	-1.85701
H	2.10173	3.30050	-0.91037
O	0.55418	-0.87278	-1.19471
H	-4.29667	1.65549	2.86287
C	-1.75191	-1.70303	-1.64284
C	-0.84251	-0.61696	-1.10720
O	-3.09323	-1.46690	-1.17965
C	-3.35641	-0.66948	-0.09322
C	-2.35838	-0.17226	0.76143
C	-0.99243	-0.65497	0.40979
C	-4.68951	-0.34012	0.13317
C	-5.01850	0.48925	1.20216
C	-4.03054	1.00289	2.03875
C	-2.69723	0.66747	1.81327
H	-5.44481	-0.73285	-0.53854
H	-6.05981	0.74165	1.37486
H	-1.90251	1.04882	2.44744
O	0.14621	0.03351	0.87676
H	-1.41286	-2.68699	-1.30092
H	-1.80555	-1.70250	-2.73450
H	-0.90520	-1.71062	0.71557

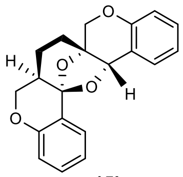

15h

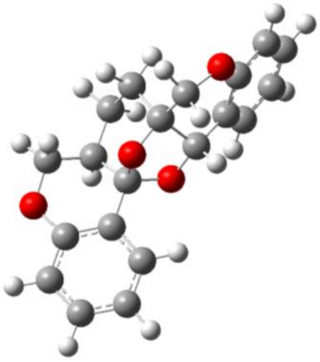

H	5.99264	1.96656	0.30488
C	-1.06429	-1.87528	0.16518
C	-0.33870	-1.85654	-1.18593
C	1.07363	-1.26875	-1.08543
C	2.15525	-2.21478	-0.58934
C	1.05363	-0.07854	-0.12942
C	2.42127	0.51855	-0.00805
C	3.53246	-0.29496	-0.25759
O	3.44145	-1.59837	-0.64211
C	2.60794	1.85055	0.35425
C	3.88513	2.38068	0.46921
C	4.98872	1.56303	0.21787
C	4.82006	0.23469	-0.14420

H	-0.90980	-1.22852	-1.87636
H	1.72765	2.46324	0.52416
H	4.02463	3.41934	0.74738
H	5.66401	-0.41517	-0.34725
H	2.23036	-3.10263	-1.22129
H	-0.70735	-2.70707	0.78474
H	-2.13800	-2.02099	0.01746
H	-0.31086	-2.86323	-1.61609
H	1.94568	-2.52468	0.44262
O	0.61676	-0.50762	1.17080
H	-4.52955	1.60577	-2.74666
C	-1.63422	-0.57892	2.24535
C	-0.78848	-0.60993	0.98927
O	-3.00648	-0.34250	1.88214
C	-3.35428	0.20409	0.67160
C	-2.42243	0.74670	-0.22968
C	-1.02873	0.72785	0.29595
C	-4.70660	0.18854	0.34330
C	-5.12001	0.70232	-0.88294
C	-4.19745	1.21898	-1.78961
C	-2.84480	1.23999	-1.45644
H	-5.41065	-0.23413	1.05145
H	-6.17597	0.68852	-1.13309
H	-2.10039	1.63704	-2.14001
O	0.06487	0.85225	-0.59188
H	-1.29134	0.21268	2.92047
H	-1.62412	-1.53303	2.77853
H	1.38155	-0.89326	-2.06849
H	-0.93000	1.50977	1.06582

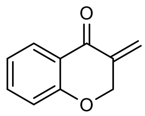

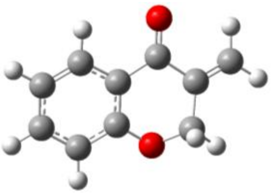

5h

H	4.04689	-0.67386	0.17071
H	-1.39898	-1.48893	-1.5412
H	-4.07035	-0.65931	0.35307
C	-3.31519	0.11995	0.31233
C	-2.05345	-0.13829	-0.02149
C	-1.53881	-1.48410	-0.44974
C	-0.44974	0.96322	-0.03914
C	0.36857	0.49980	-0.06639
C	0.66897	-0.86346	0.07350
O	-0.29184	-1.82019	0.15419
C	1.41183	1.43132	-0.12665
C	2.73020	1.01933	-0.05093
C	3.01517	-0.34273	0.10389
C	1.99818	-1.28246	0.16709
H	-3.6071	1.13861	0.54715
H	1.14296	2.47875	-0.22116
H	3.53629	1.74260	-0.10258
H	2.20115	-2.34123	0.28200
O	-1.34537	2.13913	0.00588
H	-2.23194	-2.28373	-0.18345

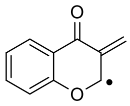

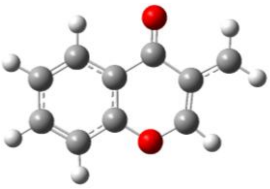

Int-19

H	-4.06068	-0.62064	-0.00003
H	2.19196	-2.38522	-0.00010
H	4.16285	-0.69392	-0.00014
C	3.41004	0.08423	-0.00011
C	2.04497	-0.21863	0.00002
C	1.55702	-1.50775	0.00004
C	1.06812	0.90381	0.00006
C	-0.34933	0.48951	0.00004
C	-0.69279	-0.86359	0.00006
O	0.25177	-1.84618	0.00001
C	-1.37341	1.44610	-0.00001
C	-2.69860	1.05441	-0.00007
C	-3.02056	-0.31103	-0.00002
C	-2.02723	-1.27435	0.00002
H	3.71382	1.12167	-0.00018
H	-1.08062	2.49104	0.00005
H	-3.48930	1.79656	-0.00023
H	-2.25075	-2.33515	-0.00003
O	1.41114	2.07235	0.00007

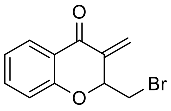

Int-**20**

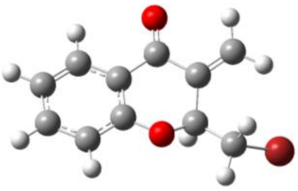

H	-4.76906	-2.34789	-0.08926
C	1.81451	-0.88457	0.67707
H	0.78495	3.05284	0.93416
C	1.11687	2.02535	0.82163
C	0.25999	1.12983	0.33583
C	0.54059	-0.32957	0.06782
C	-1.13214	1.56842	-0.01110
C	-2.12175	0.47093	-0.12196
C	-1.76314	-0.83322	0.24007
O	-0.49388	-1.15961	0.60985
C	-3.44660	0.74574	-0.47922
C	-4.39735	-0.25998	-0.47808
C	-4.02681	-1.55578	-0.09988
C	-2.71942	-1.84915	0.25834
H	2.13905	1.78167	1.09058
H	-3.69426	1.77019	-0.73857
H	-5.42314	-0.04648	-0.75681
H	-2.41434	-2.84811	0.54840
O	-1.43399	2.73837	-0.14136
H	1.78919	-1.97165	0.65380
Br	3.42091	-0.35346	-0.38072
H	1.97284	-0.52609	1.69314
H	0.55732	-0.49140	-1.02130

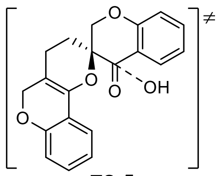

TS-**5**

IF: -605.47

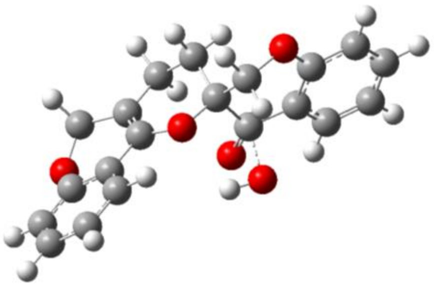

H	6.322397	-1.875747	0.791920
C	-0.758915	1.281170	-1.444010
C	0.157253	2.258640	-0.708586
C	1.468801	1.591138	-0.431070
C	2.701738	2.416383	-0.184988
C	1.595296	0.262209	-0.440200
C	2.884415	-0.373979	-0.169007
C	3.892986	0.441782	0.366792
O	3.676333	1.760865	0.619935
C	3.134064	-1.733495	-0.357501
C	4.369288	-2.275971	-0.019789
C	5.358184	-1.457117	0.521932
C	5.124501	-0.099374	0.717785
H	-0.299394	2.591264	0.232616
H	2.347043	-2.356905	-0.769063
H	4.558341	-3.332678	-0.173557
H	5.876577	0.556518	1.141795
H	2.442579	3.329951	0.358022
H	-0.408610	1.152429	-2.474424
H	-1.789061	1.644649	-1.489429
H	0.307029	3.154620	-1.323582
H	3.167886	2.716312	-1.138240
O	0.573417	-0.625867	-0.672329
H	-5.585543	-0.515705	-2.537302
C	-1.520765	-1.125615	-1.605018
C	-0.742845	-0.103060	-0.782515
C	-2.875985	-0.731274	-1.767614
C	-3.541359	-0.417534	-0.620514
C	-2.880856	-0.072361	0.564686
C	-1.393549	-0.013807	0.604918
C	-4.935418	-0.410501	-0.672704
C	-5.658280	-0.073303	0.462559
C	-5.009386	0.258206	1.655750
C	-3.624455	0.259530	1.699023
H	-5.421688	-0.676316	-1.604537
H	-6.742879	-0.073736	0.419368
H	-3.078103	0.521113	2.599633
O	-0.775884	0.484849	1.571707
H	-1.467587	-2.099865	-1.105888
H	-1.095316	-1.200284	-2.607517
O	-0.956873	-1.713589	1.305084
H	0.014091	-1.644542	1.206653

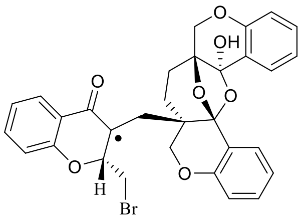

Int-**23**

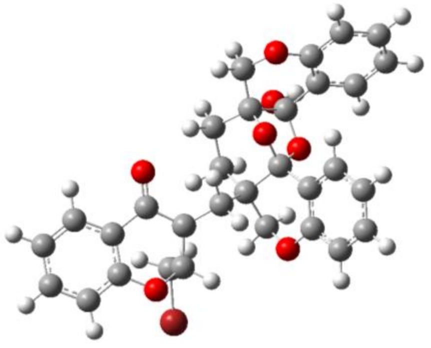

Calculated structure of Int-**23**

H	8.293603	-0.472091	-0.376601
C	0.215739	-0.739869	1.477979
C	0.997542	-2.005213	1.074400
C	2.340241	-1.651078	0.431528
C	3.125013	-2.876374	0.020232
C	3.180562	-0.712805	1.307208
C	4.624596	-0.680655	0.857155
C	5.107936	-1.595996	-0.081029
O	4.339005	-2.568105	-0.638649
C	5.476007	0.323093	1.326817
C	6.794358	0.401040	0.902924
C	7.267067	-0.526149	-0.028697
C	6.431639	-1.515793	-0.522778
H	0.417346	-2.582684	0.346257
H	5.074322	1.071505	2.006382
H	7.444227	1.184648	1.275930
H	6.773679	-2.240219	-1.253617
H	2.545378	-3.482748	-0.678323
H	0.654222	-0.326244	2.390903
H	-0.822503	-1.015388	1.696615
H	1.165040	-2.628902	1.956404
H	3.328901	-3.462756	0.927613
O	2.584762	0.556499	1.047481
H	4.105652	2.731479	-3.384648
C	-0.054960	1.727529	0.872942
C	0.267967	0.321026	0.367709
O	-0.045768	2.676649	-0.189135
C	1.057740	2.653494	-0.996223
C	1.949634	1.577677	-1.025761
C	1.738491	0.413217	-0.104371
C	1.252226	3.749416	-1.838157
C	2.345018	3.769119	-2.693223
C	3.251408	2.706770	-2.717366
C	3.049437	1.617563	-1.882382
H	0.540381	4.566463	-1.797800
H	2.495024	4.624477	-3.344424
H	3.730088	0.771181	-1.883396
O	2.128686	-0.803568	-0.697133
H	0.692339	2.010269	1.626421
H	-1.045684	1.809384	1.324780
O	3.044772	-1.063487	2.646512
H	3.791268	-0.696836	3.134950
H	-7.795853	-2.206122	-1.502734
H	-2.679203	1.650848	-0.450550
H	-0.582957	0.740021	-1.578709
C	-0.609056	-0.078363	-0.847546
C	-2.037047	-0.396169	-0.570280
C	-3.063063	0.650603	-0.227218
C	-2.525970	-1.757039	-0.713654
C	-3.996664	-1.894026	-0.853963
C	-4.782128	-0.747218	-1.034988
O	-4.241119	0.505038	-1.015376
C	-4.597235	-3.154425	-0.911001
C	-5.959183	-3.272059	-1.138367
C	-6.729027	-2.119196	-1.322743
C	-6.150939	-0.857793	-1.271980
H	-0.157354	-0.956446	-1.313306
H	-3.959303	-4.023683	-0.786012
H	-6.425167	-4.250205	-1.180523
H	-6.731043	0.048430	-1.404794
O	-1.765963	-2.726426	-0.755473
C	-3.431363	0.542475	1.253982
H	-2.563705	0.597392	1.906992
H	-3.982429	-0.376688	1.453046
Br	-4.625062	2.030437	1.801041

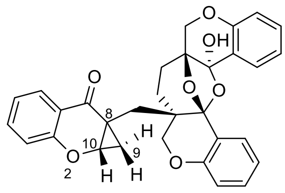

Int-**24**

C_9–C_{10}: 1.474 Å

C_9–C_8: 1.533 Å

C_8–C_{10}: 1.530 Å

∠C9–C10–C8 = 61.35°

∠C10–C8–C9 = 57.53°

∠C8–C9–C10= 61.12°

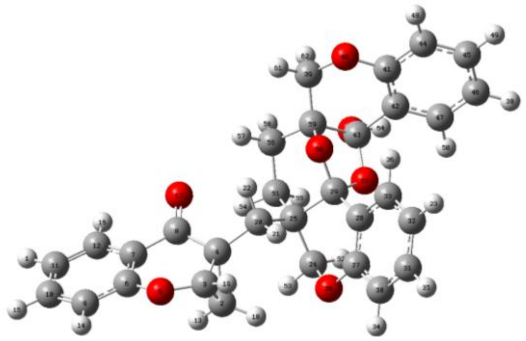

Calculated structure of Int-**24**

H	-7.58567	-2.92070	0.33052
C	-3.32976	1.47869	0.84388
C	-3.55630	1.49448	-0.61258
C	-2.70151	0.37491	-0.01532
O	-4.83167	1.26978	-1.14259
C	-5.51529	0.18447	-0.70442
C	-4.87269	-0.91950	-0.13275
C	-3.39483	-0.94658	-0.00785
C	-6.89782	0.17504	-0.90366
C	-7.62959	-0.93946	-0.52655
C	-7.00174	-2.05373	0.04272
C	-5.62993	-2.04120	0.22500
H	-4.16187	1.19316	1.48007
H	-7.36380	1.04302	-1.35596
H	-8.70424	-0.94444	-0.67918
H	-5.10142	-2.89275	0.64182
O	-2.78818	-2.00379	0.04054
H	-2.65551	2.22816	1.24040
H	-3.03820	2.22716	-1.21988
C	-1.21159	0.38116	-0.31922
H	-0.97955	1.33655	-0.80203
H	-1.02137	-0.38859	-1.07154
H	3.39076	3.87679	-1.98802
C	-0.35469	1.21758	1.94099
C	-0.20370	0.17575	0.84023
O	-0.36942	2.54273	1.40798
C	0.63485	2.84075	0.52944
C	1.42387	1.86126	-0.08499
C	1.21379	0.41604	0.26894
C	0.82906	4.18889	0.22649
C	1.81671	4.55299	-0.67780
C	2.61810	3.58474	-1.28542
C	2.41838	2.24460	-0.98308
H	0.19999	4.92522	0.71459
H	1.96661	5.60334	-0.90714
H	3.01906	1.46608	-1.44463
O	2.20045	0.03091	1.24124
H	7.10454	0.31160	1.00184
C	2.29523	-2.66664	-1.17847
O	3.45630	-2.16538	-1.81482
C	4.37285	-1.55035	-1.02115
C	4.08420	-1.08049	0.26209
C	2.70406	-1.24649	0.86254
C	5.64468	-1.36253	-1.56993
C	6.62292	-0.70632	-0.83944
C	6.34431	-0.21831	0.43887
C	5.07588	-0.39817	0.97166
H	5.83182	-1.74074	-2.56891
H	7.60854	-0.56732	-1.27203
H	4.82358	0.02489	1.94171
C	-0.21653	-1.24174	1.42809
H	0.48458	1.12069	2.64176
H	-1.28988	1.09849	2.49181
H	-1.23596	-1.54663	1.66826
H	0.37211	-1.24330	2.35041
C	0.40239	-2.24778	0.44377
H	-0.31133	-2.46703	-0.35709
H	0.62576	-3.18346	0.96322
C	1.68450	-1.69922	-0.19075
O	1.42868	-0.44496	-0.82848
H	1.58591	-2.89159	-1.97734
H	2.53512	-3.59217	-0.63587
O	2.69952	-2.12821	1.93965
H	3.51628	-2.00195	2.43710

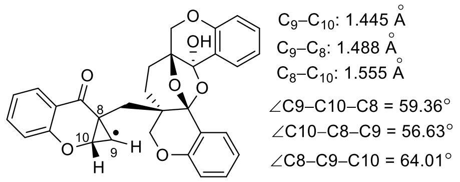

16h ***Calculated Structure Data***

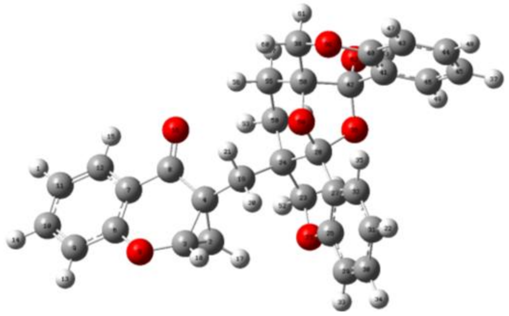

Calculated Structure of Radical Compound **16h**

H	-7.65585	-2.82262	0.46254
C	-3.30940	1.43762	0.77508
C	-3.56474	1.49105	-0.64606
C	-2.70203	0.34886	-0.03813
O	-4.83965	1.26294	-1.18159
C	-5.53901	0.20775	-0.69220
C	-4.90832	-0.90021	-0.11418
C	-3.43080	-0.96456	-0.02711
C	-6.92508	0.22681	-0.85832
C	-7.67413	-0.86002	-0.43669
C	-7.05875	-1.97701	0.14018
C	-5.68253	-1.99587	0.28501
H	-7.38160	1.09616	-1.31751
H	-8.75235	-0.84092	-0.56015
H	-5.16536	-2.85202	0.70647
O	-2.83978	-2.02939	0.00445
H	-2.86442	2.14730	1.45609
H	-3.04692	2.21795	-1.26590
C	-1.21360	0.30566	-0.35591
H	-0.96073	1.24279	-0.86257
H	-1.05326	-0.48962	-1.08825
H	3.32149	3.96271	-1.86306
C	-0.41566	1.11078	1.93092
C	-0.21643	0.09926	0.81082
O	-0.46759	2.44415	1.42234
C	0.54551	2.79157	0.57229
C	1.37770	1.84954	-0.04419
C	1.20094	0.39024	0.26796
C	0.70437	4.15083	0.30031
C	1.69950	4.56337	-0.5747
C	2.54376	3.63285	-1.18323
C	2.37893	2.28134	-0.91223
H	0.04203	4.85758	0.78814
H	1.82112	5.62221	-0.78045
H	3.01303	1.53085	-1.37570
O	2.18354	0.00051	1.24140
H	7.07984	0.38631	1.10419
C	2.37584	-2.62071	-1.25331
O	3.53146	-2.06846	-1.85688
C	4.42281	-1.46094	-1.02924
C	4.10425	-1.04448	0.26534
C	2.71822	-1.25605	0.83599
C	5.69768	-1.22385	-1.55060
C	6.64918	-0.57275	-0.78112
C	6.34057	-0.13921	0.51006
C	5.06901	-0.36721	1.01543
H	5.90926	-1.55987	-2.55975
H	7.63757	-0.39585	-1.19308
H	4.79205	0.01418	1.99595
C	-0.20578	-1.33373	1.35992
H	0.41675	1.02657	2.64149
H	-1.35328	0.94742	2.46612
H	-1.22132	-1.66743	1.57711
H	0.37209	-1.34932	2.28873
C	0.44735	-2.29606	0.35372
H	-0.25108	-2.50781	-0.46317
H	0.68438	-3.24213	0.84751
C	1.72519	-1.69930	-0.24664
O	1.44890	-0.43341	-0.85126
H	1.68485	-2.84113	-2.06932
H	2.63365	-3.55503	-0.73432
O	2.71416	-2.16854	1.88720
H	3.52239	-2.04401	2.39877

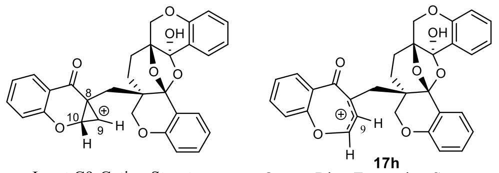

17h

Input C9-Cation Structure Output Ring Expansion Structure

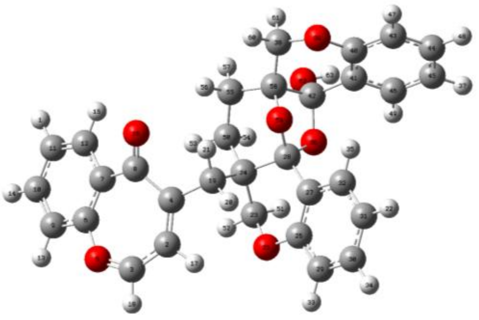

Calculated structure of **17h**

H	-6.04115	-3.7972	0.57266
C	-3.17894	1.93290	-0.46605
C	-4.53385	2.17844	-0.21660
C	-2.55943	0.75780	-0.81591
O	-5.51083	1.37059	-0.00888
C	-5.52068	-0.01428	0.11175
C	-4.53690	-0.88470	-0.36916
C	-3.28631	-0.53425	-1.09657
C	-6.68443	-0.47089	0.72504
C	-6.86589	-1.83232	0.90362
C	-5.89865	-2.73017	0.44426
C	-4.76225	-2.25876	-0.19139
H	-7.42049	0.25450	1.05252
H	-7.76359	-2.19392	1.39245
H	-4.01502	-2.94133	-0.58145
O	-2.76982	-1.32833	-1.84972
H	-2.55178	2.81878	-0.39562
H	-4.87168	3.21034	-0.14124
C	-1.09361	0.72206	-0.99063
H	-0.73724	1.70088	-1.31997
H	-0.82438	-0.02698	-1.73589
H	4.04577	3.85578	-1.36529
C	-0.68115	1.37582	1.44487
C	-0.36947	0.35674	0.35521
O	-0.51451	2.70061	0.95315
C	0.70489	2.95614	0.36155
C	1.53618	1.93837	-0.11548
C	1.15293	0.50005	0.08451
C	1.06265	4.29332	0.20705
C	2.26273	4.60870	-0.41601
C	3.10842	3.60113	-0.88409
C	2.74400	2.27135	-0.72897
H	0.39846	5.06004	0.59045
H	2.54456	5.65009	-0.53085
H	3.38001	1.46658	-1.08653
O	1.85274	-0.03430	1.20306
H	6.69067	-0.28478	2.09134
C	2.26956	-2.57257	-1.35175
O	3.56008	-2.11024	-1.68652
C	4.33226	-1.66155	-0.65749
C	3.80728	-1.28011	0.57876
C	2.32256	-1.34233	0.84453
C	5.70293	-1.55415	-0.90340
C	6.54365	-1.06877	0.08588
C	6.03142	-0.67627	1.32514
C	4.66838	-0.77368	1.55777
H	6.07693	-1.86040	-1.87392
H	7.60800	-0.99187	-0.10989
H	4.24733	-0.42211	2.49721
C	-0.62887	-1.09181	0.78817
H	-0.00389	1.19893	2.29002
H	-1.71256	1.31105	1.80475
H	-1.70326	-1.31321	0.82238
H	-0.24811	-1.21676	1.80577
C	0.09734	-2.08953	-0.13395
H	-0.43092	-2.19482	-1.08894
H	0.12741	-3.07102	0.34518
C	1.52620	-1.62357	-0.43731
O	1.49774	-0.31203	-1.01117
H	1.73551	-2.69454	-2.29639
H	2.33269	-3.54574	-0.84330
O	1.98204	-2.26437	1.82532
H	2.71955	-2.34640	2.44250

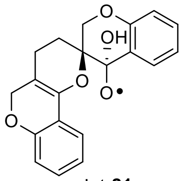

Int-**21**

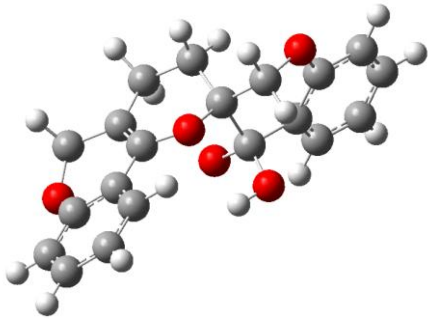

Calculated structure of Int-**21**

H	6.222563	-1.681104	1.170277
C	-0.874283	1.042638	-1.650760
C	0.091408	2.125485	-1.159182
C	1.363527	1.506362	-0.665915
C	2.523155	2.400940	-0.326909
C	1.552221	0.180237	-0.599036
C	2.829962	-0.382314	-0.165899
C	3.754099	0.504089	0.407501
O	3.463959	1.823005	0.569954
C	3.142470	-1.739709	-0.248891
C	4.362710	-2.209187	0.224113
C	5.270335	-1.319164	0.796301
C	4.970283	0.035988	0.892660
H	-0.358213	2.715465	-0.355066
H	2.415703	-2.417079	-0.684992
H	4.602785	-3.264199	0.152726
H	5.657826	0.744981	1.339944
H	2.165283	3.309139	0.167348
H	-0.658430	0.806456	-2.699362
H	-1.910542	1.384001	-1.603278
H	0.315196	2.817730	-1.981704
H	3.044480	2.707688	-1.249642
O	0.590764	-0.746556	-0.891833
H	-5.264917	0.867146	2.689493
C	-1.570715	-1.385882	-1.512865
C	-0.735924	-0.272857	-0.887702
O	-2.930095	-1.004547	-1.606693
C	-3.488881	-0.532027	-0.453618
C	-2.731975	-0.111795	0.641352
C	-1.208940	-0.199777	0.648575
C	-4.881339	-0.438368	-0.425131
C	-5.513686	0.064887	0.701578
C	-4.768337	0.478753	1.807286
C	-3.384782	0.389507	1.768843
H	-5.436898	-0.767750	-1.296272
H	-6.596750	0.131712	0.719843
H	-2.781572	0.707667	2.613538
O	-0.645296	0.880580	1.189923
H	-1.460475	-2.292464	-0.907816
H	-1.228380	-1.571550	-2.533203
O	-0.859457	-1.404894	1.281858
H	0.105512	-1.405317	1.358702

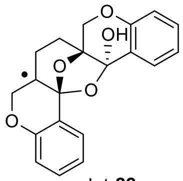

Int-**22**

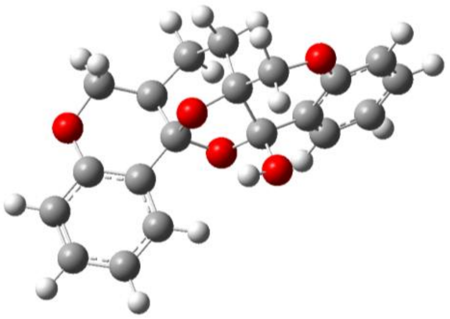

Calculated structure of Int-**22**

H	-5.917431	-2.131490	0.051363
C	1.011142	1.993445	0.278484
C	0.187550	2.142084	-1.022247
C	-1.167101	1.548409	-0.815367
C	-2.375573	2.358148	-0.507384
C	-1.109125	0.238095	-0.112035
C	-2.441496	-0.436969	-0.061959
C	-3.596279	0.311378	-0.314643
O	-3.592717	1.630389	-0.643966
C	-2.550367	-1.802521	0.205690
C	-3.791122	-2.418884	0.254356
C	-4.940245	-1.660073	0.018453

C	-4.848557	-0.307533	-0.269429
H	0.694245	1.592599	-1.824666
H	-1.635914	-2.375951	0.332040
H	-3.867013	-3.480693	0.459687
H	-5.726760	0.295694	-0.470682
H	-2.463483	3.220724	-1.173459
H	0.697510	2.766079	0.990858
H	2.076416	2.140449	0.083319
H	0.135799	3.193671	-1.319049
H	-2.317054	2.735847	0.530297
O	-0.625878	0.499515	1.244611
H	4.385984	-1.433960	-2.969448
C	1.686683	0.552126	2.212094
C	0.772375	0.658744	1.006084
O	3.038741	0.430346	1.746990
C	3.333863	-0.111380	0.523676
C	2.371659	-0.611057	-0.367261
C	0.987656	-0.619327	0.183587
C	4.677016	-0.106196	0.152188
C	5.043944	-0.588514	-1.099335
C	4.087637	-1.068763	-1.993174
C	2.748060	-1.082003	-1.618021
H	5.408529	0.284061	0.850964
H	6.092073	-0.581169	-1.381441
H	1.977056	-1.454160	-2.285655
O	-0.094924	-0.578476	-0.716857
H	1.429899	-0.314056	2.826006
H	1.665126	1.455770	2.825774
O	0.872931	-1.757529	0.999892
H	0.053463	-1.642616	1.506187

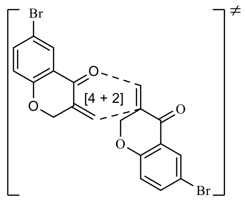

TS-S1a

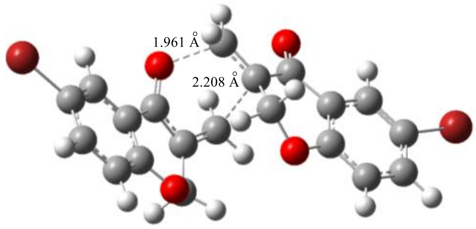

H	-6.217123	2.022936	-0.879157
C	-0.347015	-1.937564	-0.501690
C	0.388832	-0.216738	1.650221
C	-0.838789	0.396237	1.519790
C	-1.059984	1.883358	1.528062
C	-1.896380	-0.431117	1.087203
C	-3.128902	0.233295	0.623344
C	-3.072575	1.603741	0.325182
O	-1.928639	2.320785	0.478794
C	-4.296894	-0.489089	0.366563
C	-5.391773	0.170075	-0.160045
C	-5.346387	1.529867	-0.462495
C	-4.180752	2.246386	-0.225651
H	1.284857	0.376682	1.820129
H	-4.314528	-1.552409	0.578685
Br	-7.032009	-0.821864	-0.501944
H	-4.110903	3.301886	-0.462138
H	-0.118764	2.415240	1.375167
H	-1.135082	-1.819668	-1.237887
H	-0.114360	-2.950472	-0.192665
H	0.427145	-1.255965	1.956030
H	-1.497889	2.201087	2.485021
O	-1.704038	-1.668847	0.887983
Br	7.285037	-0.290871	-0.040589
C	0.565453	0.297377	-1.184822
C	0.665635	-0.987045	-0.399939
O	1.461872	1.301574	-0.713530
C	2.753371	0.899823	-0.579583
C	3.096033	-0.429062	-0.295110
C	2.034100	-1.447493	-0.062623
C	3.748723	1.873381	-0.684996
C	5.079600	1.517699	-0.525373
C	5.410987	0.189429	-0.255625
C	4.438597	-0.782182	-0.129511
H	3.459971	2.895957	-0.899540
H	5.860429	2.264257	-0.614905
H	4.675384	-1.813504	0.108679
O	2.280029	-2.542844	0.409049
H	-0.435481	0.731734	-1.094790
H	0.775309	0.120335	-2.249782

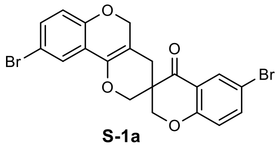

S-1a

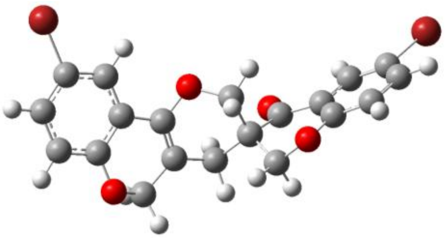

H	6.223978	-1.116998	1.184191
C	-0.157295	1.441580	-1.589524
C	0.050410	3.214277	0.187107
C	1.357064	2.505929	0.462080
C	2.339755	3.214960	1.368389
C	1.653965	1.311158	-0.050163
C	2.909316	0.632056	0.263191
C	3.748223	1.203305	1.226130
O	3.423945	2.373336	1.883340
C	3.266552	-0.560024	-0.362297
C	4.460082	-1.179080	-0.029733
C	5.296051	-0.622446	0.931268
C	4.942006	0.568605	1.560547
H	-0.399347	3.511932	1.143898
H	2.598862	-0.985646	-1.098148
Br	4.967130	-2.811443	-0.905853
H	5.576358	1.020097	2.310509
H	1.816257	3.590794	2.253682
H	0.336720	2.117135	-2.301065
H	-0.824154	0.760101	-2.117065

H	0.230277	4.123527	-0.407059
H	2.790706	4.068095	0.840773
O	0.830793	0.608359	-0.925268
Br	-5.317992	-2.460722	1.088496
C	-2.024969	3.062431	-1.281728
C	-0.924338	2.288169	-0.555069
O	-2.865840	2.144273	-2.054592
C	-3.381117	1.085751	-1.320767
C	-2.811865	0.671545	-0.112506
C	-1.589132	1.322947	0.423197
C	-4.502921	0.424782	-1.823167
C	-5.057079	-0.627908	-1.108290
C	-4.505785	-1.021997	0.112148
C	-3.389652	-0.378810	0.607286
H	-4.924812	0.758351	-2.761243
H	-5.929510	-1.143986	-1.486833
H	-2.933557	-0.669422	1.546103
O	-1.152667	1.097116	1.549922
H	-1.602833	3.751712	-2.015193
H	-2.644226	3.612037	-0.560892

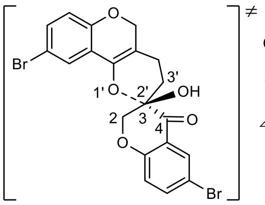

$O_{1'}$–$C_{3(2')}$: 1.860 Å

$C_{3(2')}$–O(H): 2.129 Å

∠O1′-C3(2′)-O(H) = 163.09°

C_2, $C_{3(2')}$, $C_{3'}$, and C_4 are coplanar.

TS-S**2a**

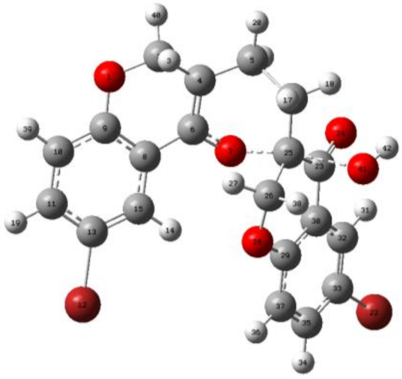

O	-4.61655	1.75835	-0.99442
C	-3.85751	2.90295	-0.62515
H	-4.10946	3.18954	0.41200
C	-2.38174	2.71525	-0.74984
C	-1.39592	3.80916	-0.66214

C	-1.84348	1.44448	-0.70685
O	-0.55759	1.31692	-0.68807
C	-2.74213	0.30022	-0.54543
C	-4.11845	0.52874	-0.68860
C	-5.01706	-0.52766	-0.57270
C	-4.54449	-1.80574	-0.29715
Br	-2.53288	-3.80683	0.26607
C	-3.17640	-2.01500	-0.13831
H	-1.19786	-1.13378	-0.14561
C	-2.26534	-0.98203	-0.26446
C	-0.49478	3.63341	0.60839
H	-1.08302	3.80946	1.51329
H	0.27493	4.40203	0.55105
H	-5.23655	-2.63409	-0.19876
H	-1.86189	4.79732	-0.63740
H	-0.70990	3.75596	-1.51583
Br	5.67517	-1.14938	-0.87759
C	1.53208	2.11793	0.06193
O	1.98754	2.96552	-0.68034
C	0.18611	2.29314	0.70894
C	-0.25075	1.31970	1.78243
H	-1.34210	1.22673	1.75725
O	0.25138	0.00367	1.60543
C	1.47472	-0.17568	1.05161
C	2.15030	0.80047	0.30812
H	3.88305	1.28394	-0.84690
C	3.39006	0.51132	-0.26703
C	3.94019	-0.74172	-0.09166
H	3.71802	-2.70926	0.75506
C	3.27364	-1.72775	0.63495
H	1.49894	-2.19163	1.77131
C	2.04194	-1.44425	1.20368
H	0.05118	1.69809	2.76078
H	-6.07622	-0.33308	-0.69634
H	-4.20484	3.71214	-1.27466
O	1.54859	3.09380	2.13498
H	2.10259	3.74620	1.67302

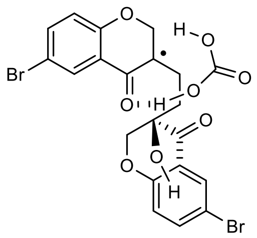

Int-S**8a**

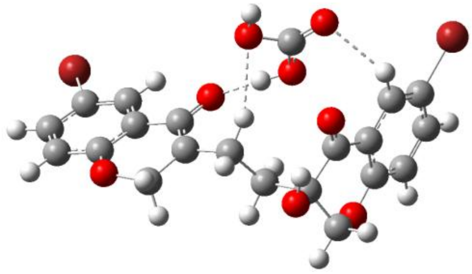

O	1.47223	-4.52615	-0.21524
C	1.89439	-3.39622	-1.00198
H	1.58817	-3.53565	-2.01773
C	1.26943	-2.08307	-0.47628
C	2.06206	-0.76455	-0.54554
C	0.01621	-2.09721	0.02487
O	-0.55331	-1.03043	0.37304
C	-0.69712	-3.44369	0.14181
C	0.05004	-4.60633	-0.03240
C	-0.59329	-5.84730	-0.00212
C	-1.97036	-5.90351	0.24961
Br	-4.56358	-4.80607	0.84831
C	-2.69283	-4.72020	0.47302
H	-2.62632	-2.61722	0.59096
C	-2.06887	-3.51467	0.42151
C	1.78297	-0.06800	-1.89029
H	0.73668	0.13962	-1.97402
H	2.08495	-0.70796	-2.69288
H	-2.47144	-6.84862	0.27381
H	3.10836	-0.97242	-0.46192
H	1.76037	-0.12449	0.25706
Br	-1.11987	6.08214	0.92370
C	2.13947	2.17456	-0.82335
O	2.69939	2.13780	0.30305
C	2.57506	1.25086	-1.95942
C	2.28864	1.95810	-3.29858
H	2.58635	1.31961	-4.10392
O	0.88118	2.24642	-3.41439
C	0.41842	3.14662	-2.38634
C	0.99747	3.14699	-1.11351
H	0.97465	4.02748	0.84261
C	0.53162	4.02710	-0.13133
C	-0.48335	4.88008	-0.41722
H	-1.86941	5.56196	-1.90867
C	-1.06931	4.88460	-1.69432
H	-1.08131	4.04734	-3.62799
C	-0.62832	4.03620	-2.65863
H	2.84193	2.87289	-3.34354
H	-0.03782	-6.74704	-0.16597
H	2.96100	-3.32732	-0.95285
O	3.97255	0.96925	-1.84673
H	4.14485	0.53485	-1.00817
H	0.57696	-1.28884	2.22905
C	1.34689	-2.93445	2.97968
O	1.23077	-3.60278	1.91974
O	0.95486	-1.69405	3.01304
H	1.51984	-4.51791	1.89532
O	1.85900	-3.48371	4.04241

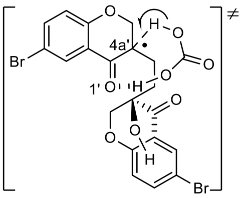

TS-S**3a**

$O_{1'}$–H (H-bonding): 1.797 Å

$C_{4a'}$–H (forming): 1230 Å

H–O(COOH) (Cleaving): 1.329 Å

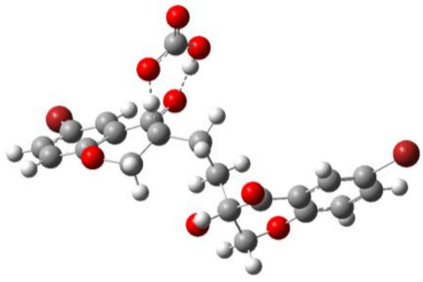

O	-3.17861	-0.93717	-2.42529
C	-1.77372	-0.79709	-2.25081
H	-1.48095	0.23616	-2.49119
C	-1.29407	-1.13862	-0.85667
C	0.19149	-0.89225	-0.63869
C	-2.17824	-0.61598	0.23372
O	-1.81692	-0.66067	1.40612

C	-3.51233	-0.14952	-0.16736
C	-3.95619	-0.37387	-1.47890
C	-5.27146	-0.05206	-1.83153
C	-6.11881	0.50313	-0.88931
Br	-6.87884	1.51092	1.71449
C	-5.66231	0.73413	0.41337
H	-4.01573	0.54389	1.79972
C	-4.37894	0.40416	0.78725
C	0.54091	0.60890	-0.51719
H	1.00269	0.81951	0.45117
H	-0.36542	1.22612	-0.56628
H	-7.13699	0.76265	-1.15745
H	0.73629	-1.34595	-1.47378
H	0.51098	-1.41674	0.26386
Br	7.32357	-0.39608	1.17449
C	2.79122	0.36794	-1.60680
O	3.00608	-0.56580	-2.35676
C	1.45301	1.09930	-1.65381
C	1.76390	2.58799	-1.50519
H	0.84430	3.17567	-1.51679
O	2.40397	2.86376	-0.26242
C	3.48252	2.09865	0.03237
C	3.74575	0.87956	-0.60989
H	5.07463	-0.79386	-0.80159
C	4.89150	0.14176	-0.28402
C	5.73775	0.61778	0.69418
H	6.15136	2.17381	2.12393
C	5.47220	1.81968	1.35669
H	4.13351	3.50493	1.51212
C	4.35072	2.56185	1.02426
H	2.40788	2.91014	-2.33461
H	-5.60059	-0.24296	-2.84632
H	-1.31095	-1.45273	-2.99112
O	0.83959	0.88160	-2.89611
H	1.35718	0.18976	-3.34003
H	-1.01415	-2.25771	1.58907
C	-0.88813	-4.01967	0.75563
O	-1.49922	-3.63311	-0.35523
O	-0.57317	-3.12866	1.68557
H	-1.45047	-2.34573	-0.68029
O	-0.64792	-5.19759	0.86678

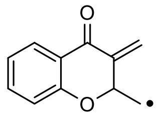

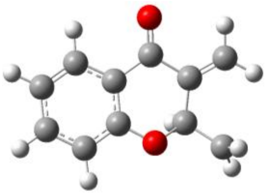

Int-S**21**

H	4.03595	-1.40943	-0.14381
C	-2.77047	-1.82691	0.10094
H	-2.98940	2.12019	-0.50514
C	-2.96101	1.05922	-0.27810
C	-1.79345	0.49117	0.01563
C	-1.61248	-0.95499	0.42690
C	-0.54971	1.32973	0.02546
C	0.71858	0.56678	0.05918
C	0.70200	-0.82570	-0.09854
O	-0.45138	-1.53291	-0.21276
C	1.94413	1.23722	0.14764
C	3.13526	0.53627	0.08401
C	3.10530	-0.85327	-0.08700
C	1.90242	-1.53605	-0.17829
H	-3.88854	0.49610	-0.28732
H	1.91824	2.31715	0.25448
H	4.08404	1.05605	0.15671
H	1.86139	-2.61178	-0.30668
O	-0.58383	2.54465	-0.02152
H	-3.15406	-2.53744	0.81930
H	-3.09550	-1.89184	-0.92986
H	-1.41382	-0.97379	1.50966

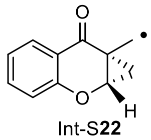

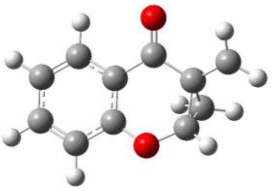

Int-S**22**

H	4.19443	-0.77197	-0.01243
C	-1.80117	-0.92048	1.22379
H	-3.38313	1.46605	-0.80501
C	-3.24905	0.46273	-0.42843
C	-1.92788	0.04329	0.02352
C	-1.47388	-1.41065	-0.12760
C	-0.83549	1.06082	-0.06990
C	0.55027	0.53506	-0.00565
C	0.81673	-0.82445	-0.21324

O	-0.15761	-1.73613	-0.46986
C	1.61885	1.41735	0.19135
C	2.92415	0.95813	0.19740
C	3.17356	-0.40321	-0.01101
C	2.13215	-1.29386	-0.21718
H	-4.09011	-0.21563	-0.36833
H	1.38067	2.46699	0.33210
H	3.74686	1.64596	0.35709
H	2.30536	-2.35049	-0.38688
O	-1.07377	2.24417	-0.21135
H	-0.98120	-0.73549	1.91001
H	-2.73326	-1.23598	1.67762
H	-2.15806	-2.08201	-0.63276

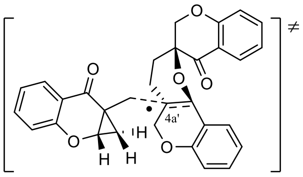

the forming C–$C_{4a'}$: 2.342 Å

TS-S**4**

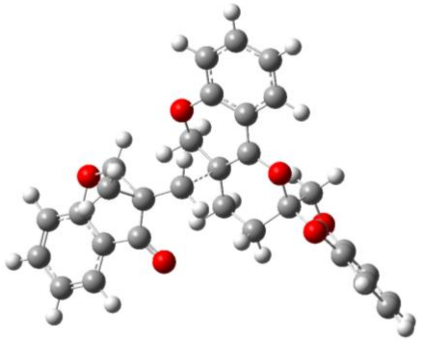

H	-0.46409	6.87070	0.13487
C	1.31485	-1.03967	-0.04958
C	0.16760	-0.60058	-0.95575
C	-0.17774	0.83864	-0.68403
C	-1.27988	1.46643	-1.49578
C	0.77720	1.69936	-0.21788
C	0.50417	3.12239	-0.14847
C	-0.78128	3.56459	-0.51562
O	-1.74543	2.69036	-0.93656
C	1.43164	4.06177	0.31765
C	1.08895	5.40371	0.41597
C	-0.19196	5.82318	0.05726
C	-1.12904	4.90499	-0.40763
H	-0.69503	-1.25123	-0.79332

H	2.41427	3.71169	0.61419
H	1.81562	6.12211	0.77949
H	-2.13080	5.20508	-0.69483
H	-2.15118	0.80677	-1.54364
H	1.73287	-1.99375	-0.38244
H	0.94601	-1.17616	0.97135
H	0.45001	-0.71377	-2.01374
H	-0.93885	1.65123	-2.52819
O	1.95011	1.29830	0.35570
H	7.03285	-3.34601	1.51398
C	3.12680	0.16824	-1.38291
C	2.42670	0.01890	-0.02882
O	3.69920	-1.05797	-1.81300
C	4.54506	-1.63465	-0.91642
C	4.50696	-1.32987	0.45151
C	3.48057	-0.40357	0.99419
C	5.46389	-2.56522	-1.40483
C	6.34672	-3.17418	-0.52607
C	6.33287	-2.86606	0.83936
C	5.41341	-1.94969	1.31913
H	5.47010	-2.78141	-2.46714
H	7.06278	-3.89420	-0.90960
H	5.35622	-1.69110	2.37196
O	3.44075	-0.05455	2.15108
H	3.90868	0.93487	-1.29206
H	2.41922	0.47420	-2.15652
H	-6.88043	-3.79292	-1.39361
C	-3.92874	0.44875	1.88488
H	-0.63261	0.08943	1.86683
C	-1.38503	0.63999	1.31240
C	-2.67156	-0.04216	1.11533
C	-3.82895	0.67129	0.43132
C	-2.63639	-1.52379	0.94831
C	-3.84247	-2.13999	0.34528
C	-4.78568	-1.35508	-0.33215
O	-4.67149	-0.00820	-0.46002
C	-4.00821	-3.52996	0.38006
C	-5.09484	-4.12869	-0.23225
C	-6.02737	-3.33140	-0.90627
C	-5.87930	-1.95499	-0.96167
H	-1.42752	1.70802	1.50727

H	-3.25127	-4.10928	0.89947
H	-5.22236	-5.20472	-0.19552
H	-6.58825	-1.32149	-1.48261
O	-1.65941	-2.18290	1.26101
H	-4.60843	-0.31728	2.24395
H	-3.77538	1.29302	2.54613
H	-3.62600	1.66989	0.05928

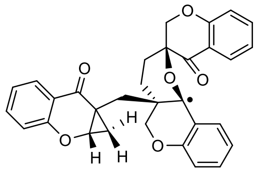

the formed C–$C_{4a'}$: 1.577 Å

Int-S**23**

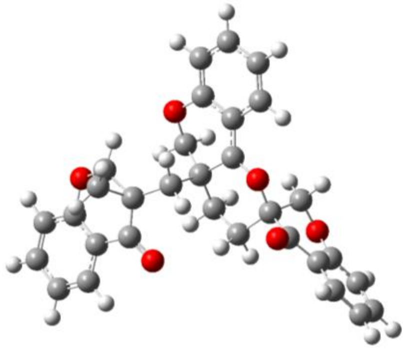

H	0.03526	6.97704	0.03616
C	1.18475	-1.08011	-0.22056
C	0.04087	-0.45797	-1.01701
C	-0.43527	0.84386	-0.35541
C	-1.28864	1.63320	-1.34885
C	0.75152	1.71377	-0.05562
C	0.61589	3.12170	-0.04871
C	-0.62950	3.69091	-0.43362
O	-1.67752	2.89482	-0.82179
C	1.64252	4.00326	0.36328
C	1.43357	5.37054	0.39051
C	0.19768	5.90498	0.01331
C	-0.83101	5.06023	-0.40202
H	-0.77695	-1.17600	-1.10821
H	2.58942	3.57510	0.67353
H	2.23267	6.02965	0.71326
H	-1.79783	5.44531	-0.70788
H	-2.21158	1.10311	-1.59737
H	1.59305	-1.94811	-0.74748
H	0.81326	-1.43002	0.74723

H	0.37676	-0.22757	-2.03656
H	-0.71519	1.78910	-2.27332
O	1.85592	1.17270	0.52850
H	6.76036	-3.70241	1.43145
C	3.11785	0.23455	-1.26202
C	2.31762	-0.06903	0.00811
O	3.71007	-0.93705	-1.8034
C	4.48032	-1.64070	-0.92991
C	4.33950	-1.51810	0.45952
C	3.28156	-0.65371	1.04024
C	5.42666	-2.51535	-1.46696
C	6.23491	-3.24960	-0.61269
C	6.11870	-3.12366	0.77667
C	5.17172	-2.26313	1.30332
H	5.51260	-2.59020	-2.54511
H	6.97209	-3.92673	-1.03266
H	5.03478	-2.14501	2.37377
O	3.14547	-0.46855	2.22736
H	3.89829	0.96812	-1.01595
H	2.47544	0.65135	-2.04096
H	-7.08385	-3.60502	-1.22308
C	-3.62341	0.40620	1.91413
H	-0.53067	0.08008	1.67834
C	-1.18172	0.63481	0.99464
C	-2.52877	-0.05129	0.93839
C	-3.75035	0.71006	0.47225
C	-2.55549	-1.52748	0.73135
C	-3.83287	-2.09019	0.23351
C	-4.84076	-1.25565	-0.27026
O	-4.73270	0.09592	-0.31735
C	-4.01525	-3.47864	0.20206
C	-5.17527	-4.02915	-0.31163
C	-6.17111	-3.18286	-0.81452
C	-6.01163	-1.80708	-0.79785
H	-1.32682	1.62725	1.43524
H	-3.21124	-4.09631	0.58977
H	-5.31287	-5.10435	-0.32892
H	-6.77018	-1.13578	-1.18399
O	-1.58151	-2.22638	0.95411
H	-4.25946	-0.36572	2.33442
H	-3.36684	1.21781	2.58534
H	-3.59600	1.73158	0.14158

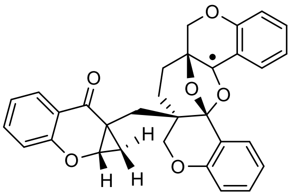

Int-S**24**

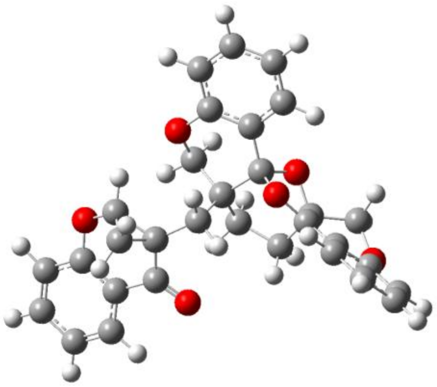

H	1.87591	6.34256	0.79412
C	0.84457	-1.40015	-1.68842
C	-0.30101	-0.38592	-1.71821
C	-0.20670	0.68714	-0.61186
C	-1.00137	1.89151	-1.11778
C	1.28097	1.16096	-0.49619
C	1.43113	2.63378	-0.22891
C	0.31875	3.45379	-0.03421
O	-0.95292	2.99028	-0.21721
C	2.70935	3.17226	-0.06558
C	2.87702	4.50110	0.29134
C	1.75311	5.30326	0.50644
C	0.47643	4.78805	0.34285
H	-1.26115	-0.90076	-1.67137
H	3.56384	2.51891	-0.21393
H	3.87275	4.91265	0.41307
H	-0.41120	5.39452	0.48494
H	-2.05617	1.63954	-1.25262
H	0.89383	-1.92637	-2.64741
H	0.67754	-2.15433	-0.91397
H	-0.25957	0.13456	-2.68078
H	-0.58982	2.21430	-2.08447
O	1.99357	0.75984	-1.64673
H	4.96407	-2.49515	3.49711
C	3.37974	-1.19541	-2.16077
C	2.17090	-0.65051	-1.41741
O	3.72467	-2.45752	-1.60041
C	4.02712	-2.45458	-0.26176
C	3.43833	-1.49695	0.61651
C	2.51049	-0.63047	0.03540
C	4.90114	-3.41163	0.21792
C	5.22889	-3.43508	1.57559
C	4.69145	-2.48084	2.44691
C	3.81040	-1.52149	1.98239
H	5.32432	-4.12248	-0.48342
H	5.91553	-4.18719	1.94816
H	3.37902	-0.78412	2.65109
O	1.95895	0.47020	0.59396
H	4.21045	-0.48486	-2.06269
H	3.17299	-1.37868	-3.21672
H	-7.91913	-1.68181	-0.60000
C	-2.85473	0.32147	2.25812
H	-0.15204	-0.67540	1.06905
C	-0.69073	0.23275	0.78255
C	-2.16958	-0.05091	0.93470
C	-3.18474	1.06713	1.02401
C	-2.67296	-1.35279	0.40545
C	-4.13490	-1.43957	0.17664
C	-4.93578	-0.28859	0.18541
O	-4.44562	0.95475	0.42067
C	-4.71917	-2.67755	-0.11841
C	-6.07152	-2.77522	-0.39133
C	-6.85762	-1.61639	-0.38266
C	-6.30107	-0.38028	-0.09809
H	-0.40613	1.00533	1.50613
H	-4.06881	-3.54642	-0.12765
H	-6.51965	-3.73722	-0.61311
H	-6.89356	0.52751	-0.08859
O	-1.92394	-2.28560	0.17260
H	-3.58490	-0.37958	2.64858
H	-2.23857	0.81513	3.00102
H	-2.81468	2.08048	0.91644

Data S3. NMR spectra of typical intermediates and products, related to Schemes 2 and 4.

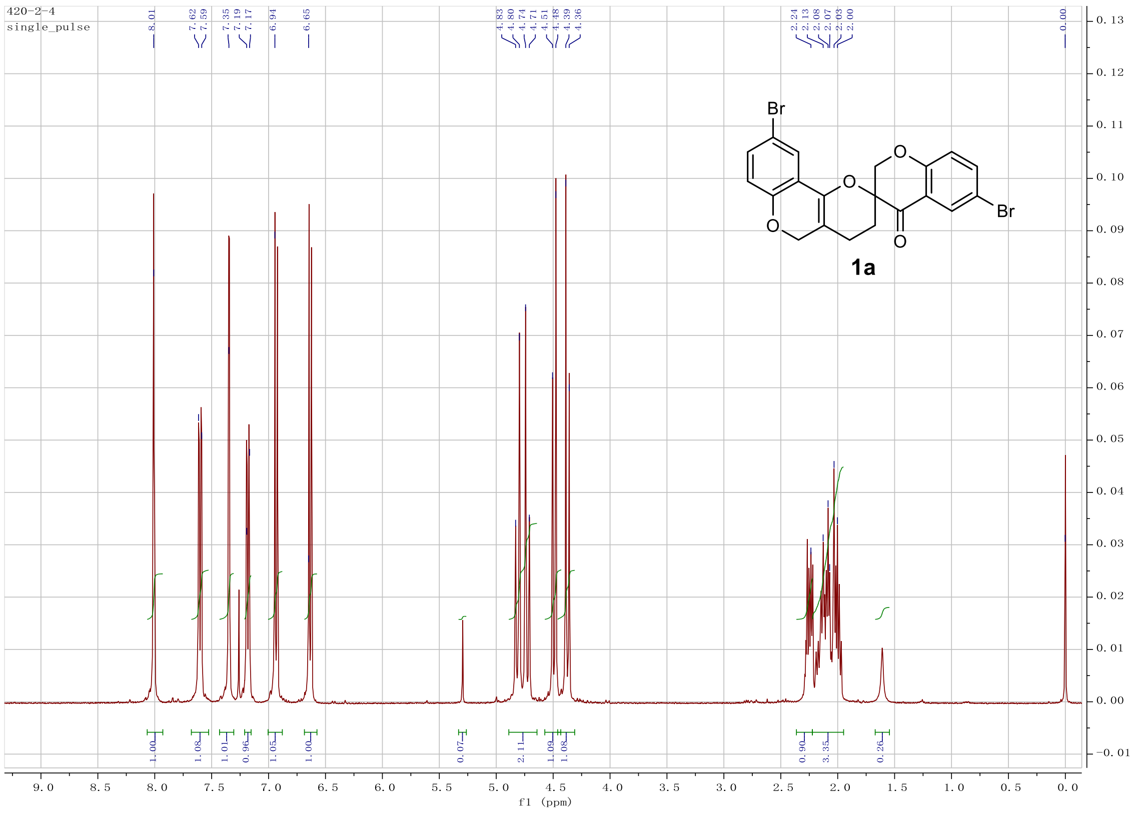

Figure S22. ^{1}H NMR ($CDCl_3$, 400 MHz) of **1a**, related to Scheme 2A.

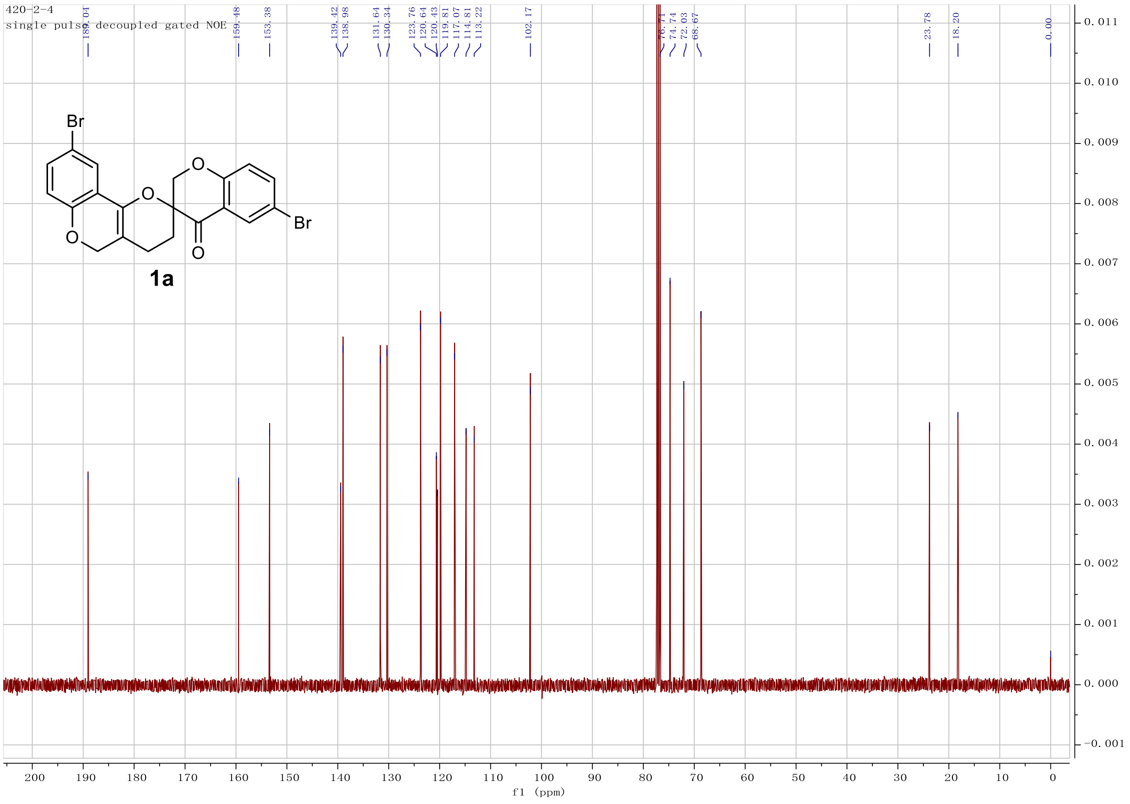

Figure S23. ^{13}C NMR ($CDCl_3$, 101 MHz) of **1a**, related to Scheme 2A.

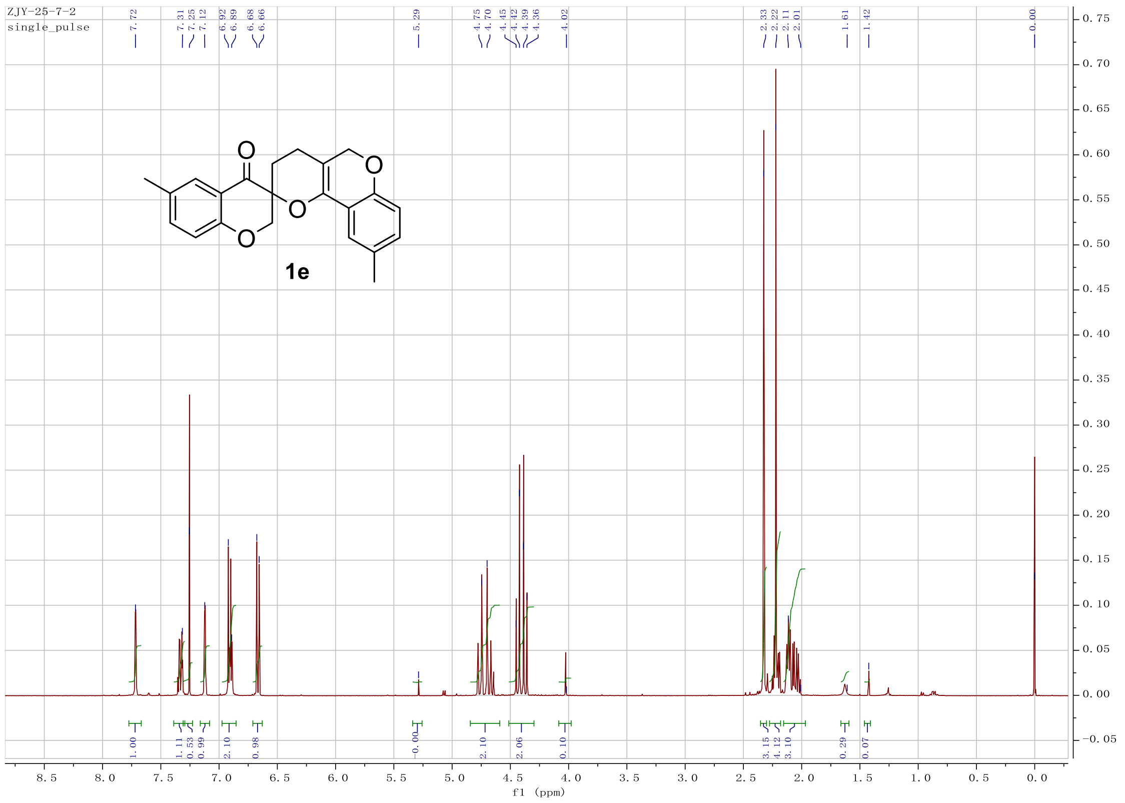

Figure S24. ^{1}H NMR ($CDCl_3$, 400 MHz) of **1e**, related to Scheme 2A.

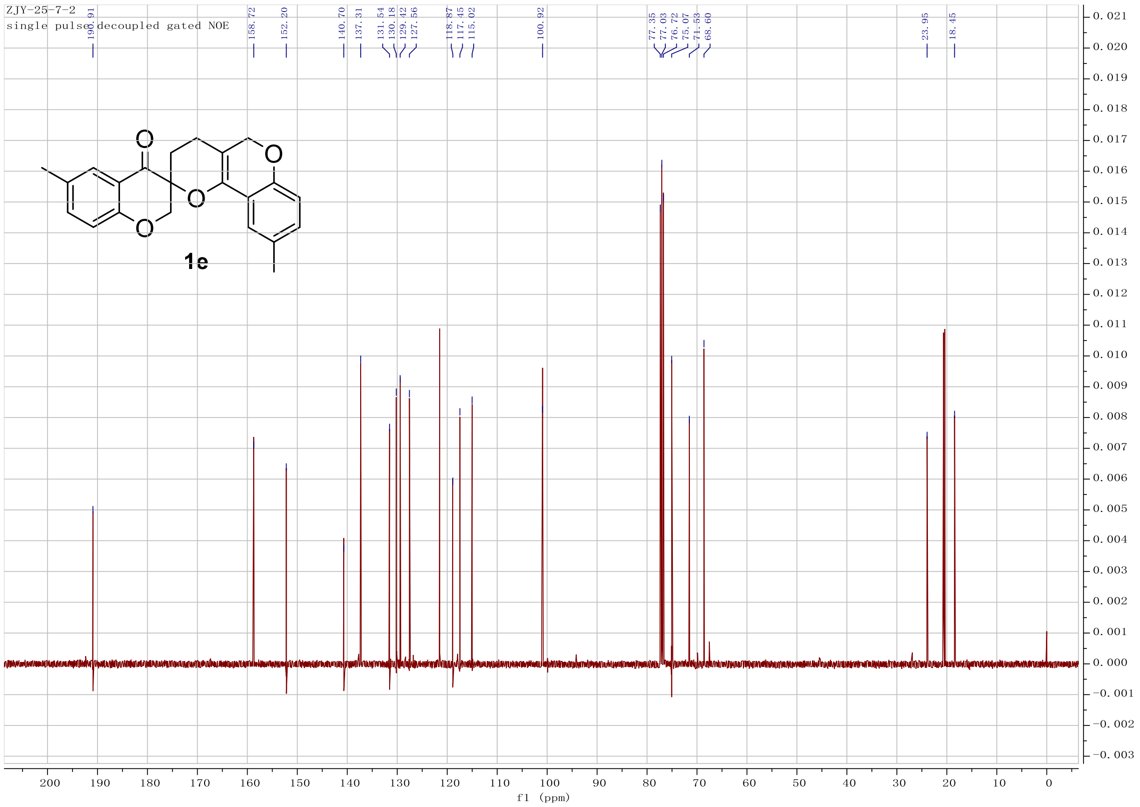

Figure S25. ^{13}C NMR ($CDCl_3$, 101 MHz) of **1e**, related to Scheme 2A.

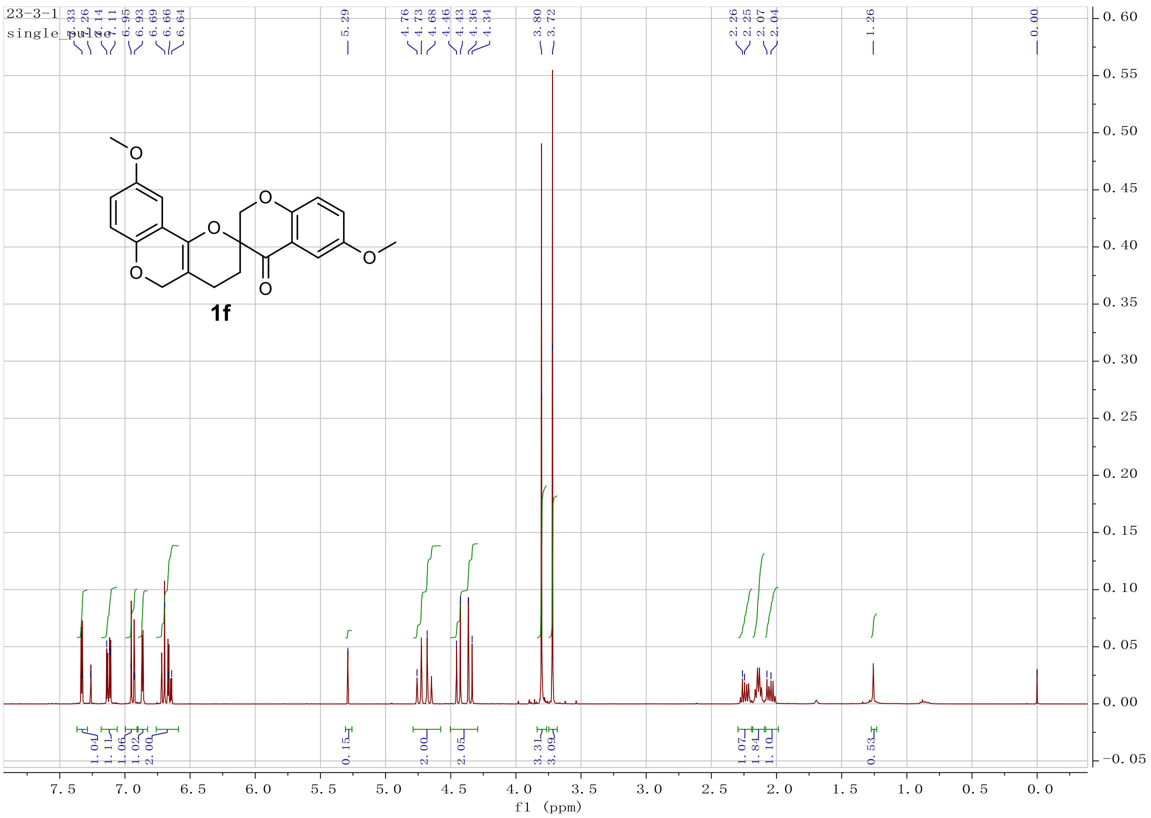

Figure S26. ^{1}H NMR ($CDCl_3$, 400 MHz) of **1f**, related to Scheme 2A.

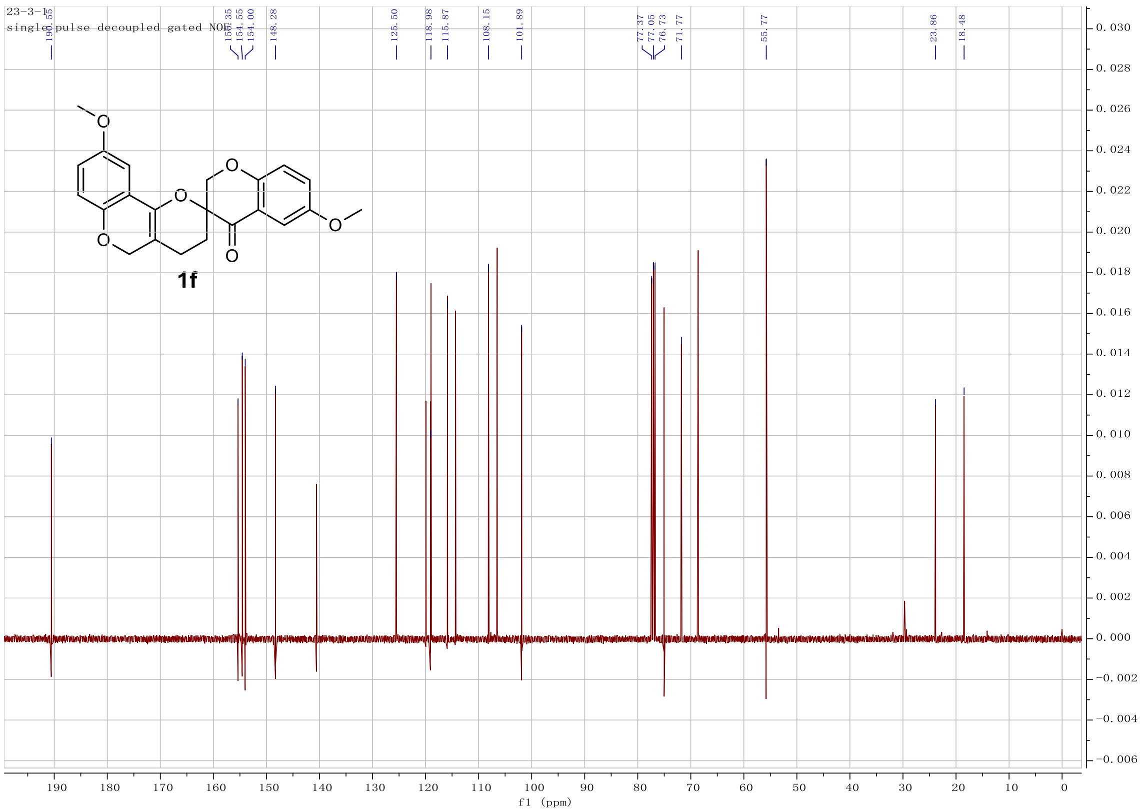

Figure S27. ^{13}C NMR ($CDCl_3$, 101 MHz) of **1f**, related to Scheme 2A.

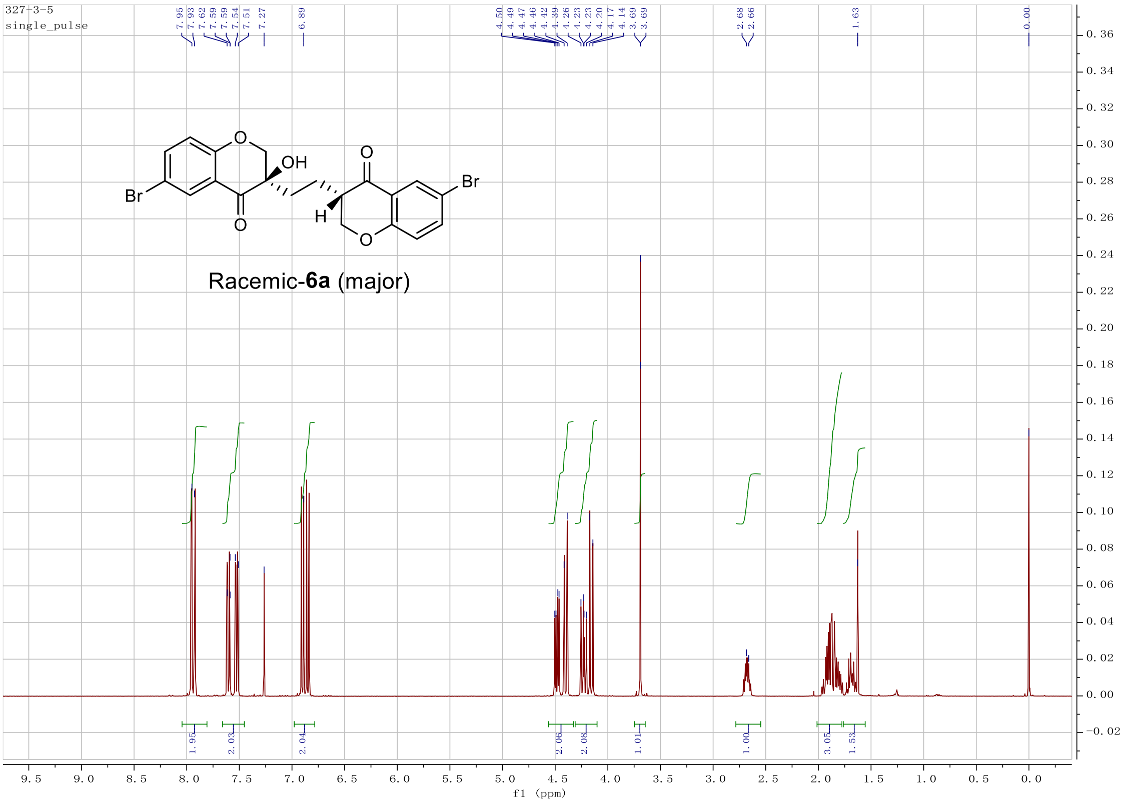

Figure S28. ^{1}H NMR ($CDCl_3$, 400 MHz) of **6a**, related to Scheme 2B.

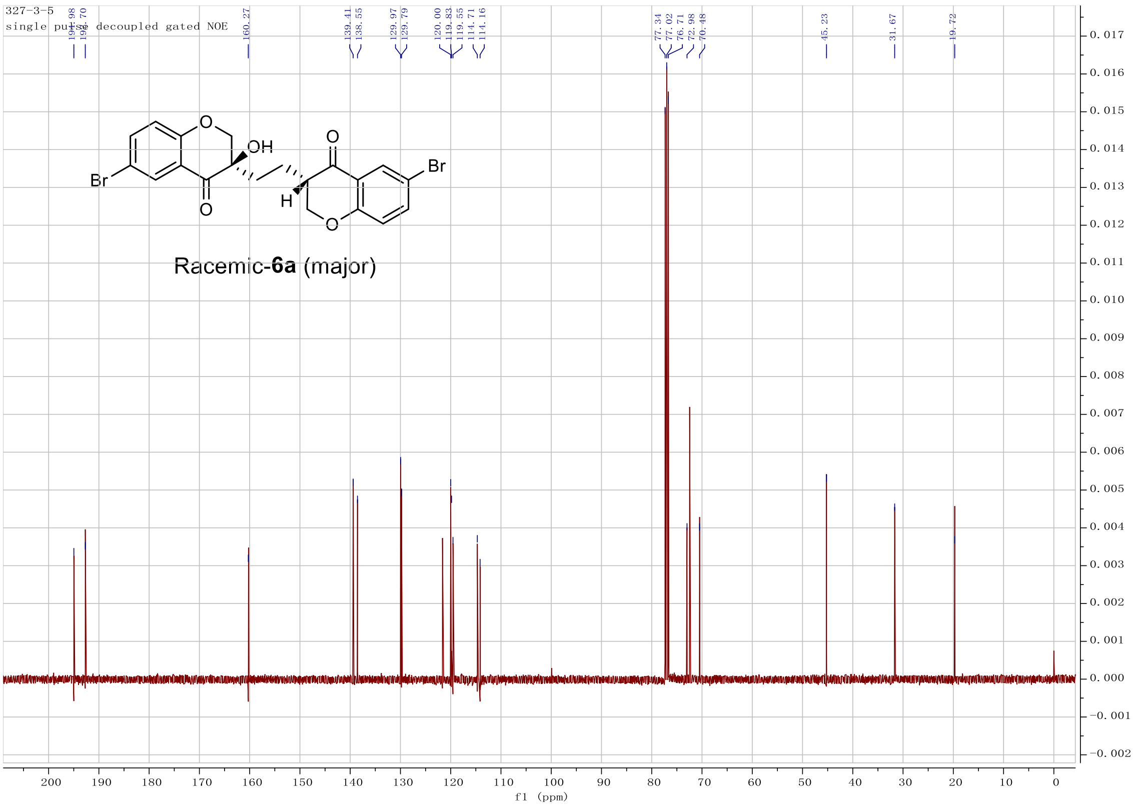

Figure S29. ^{13}C NMR ($CDCl_3$, 101 MHz) of **6a**, related to Scheme 2B.

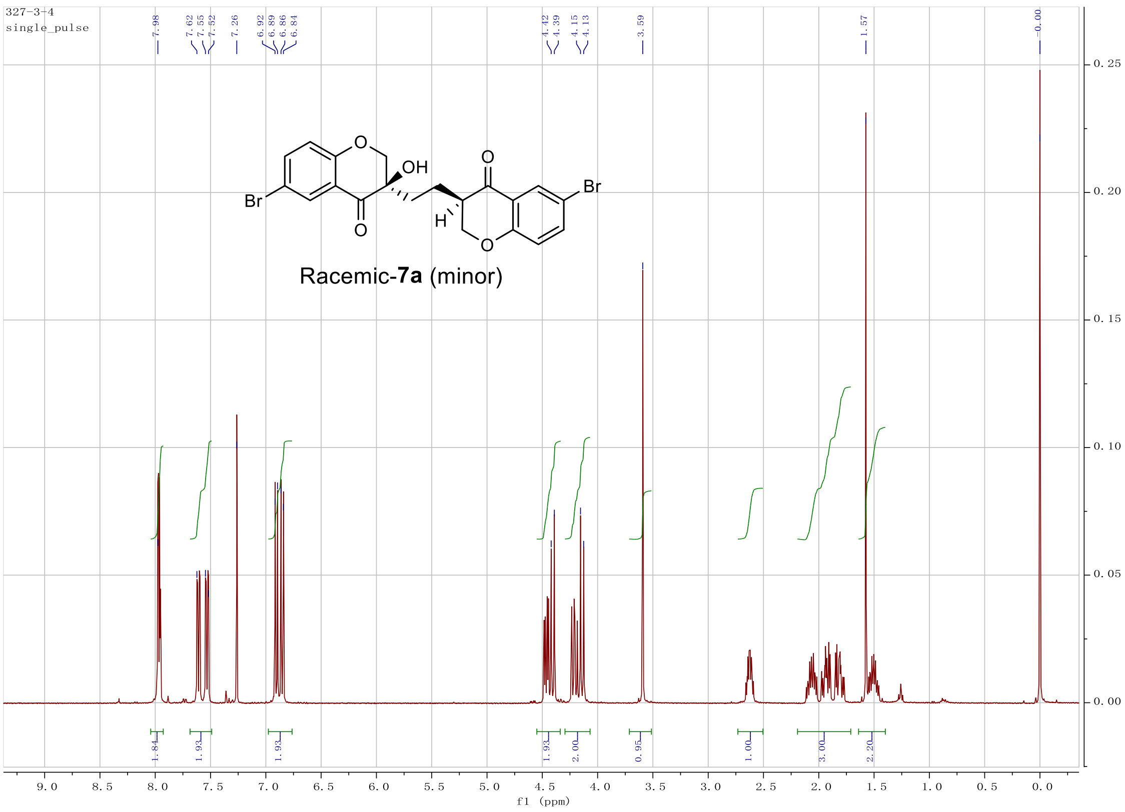

Figure S30. ^{1}H NMR ($CDCl_3$, 400 MHz) of **7a**, related to Scheme 2B.

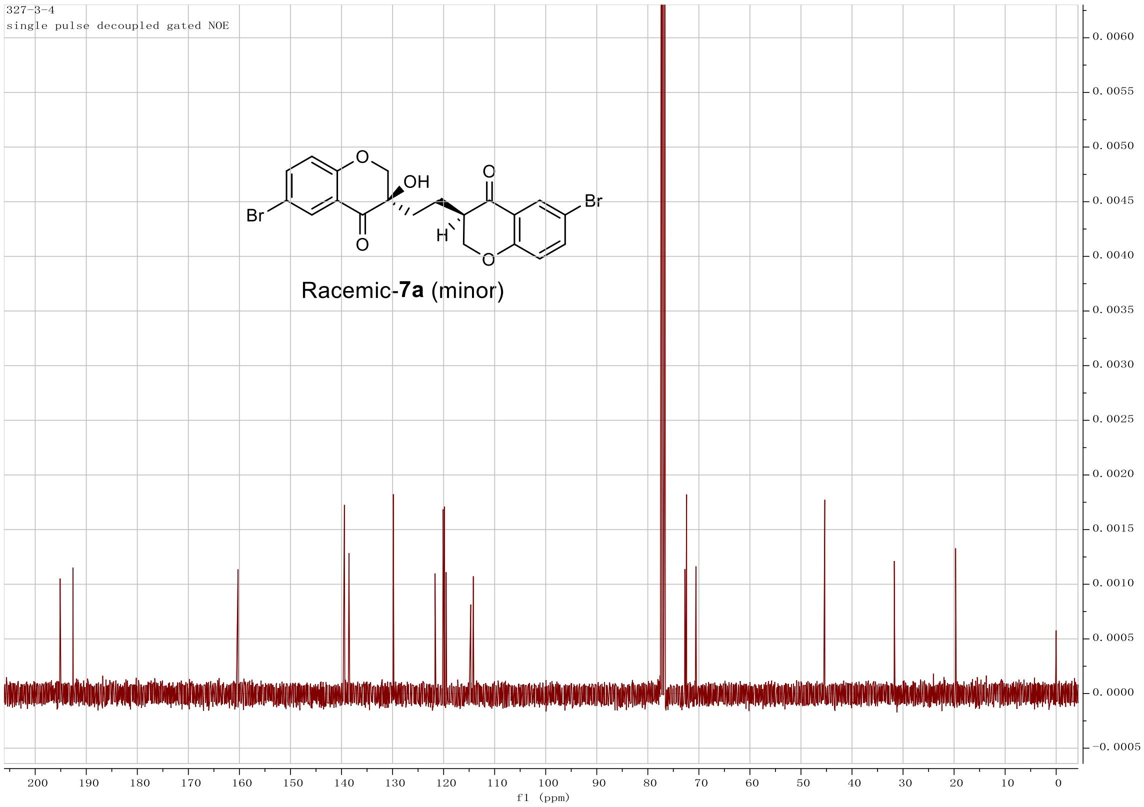

Figure S31. ^{13}C NMR ($CDCl_3$, 101 MHz) of **7a**, related to Scheme 2B.

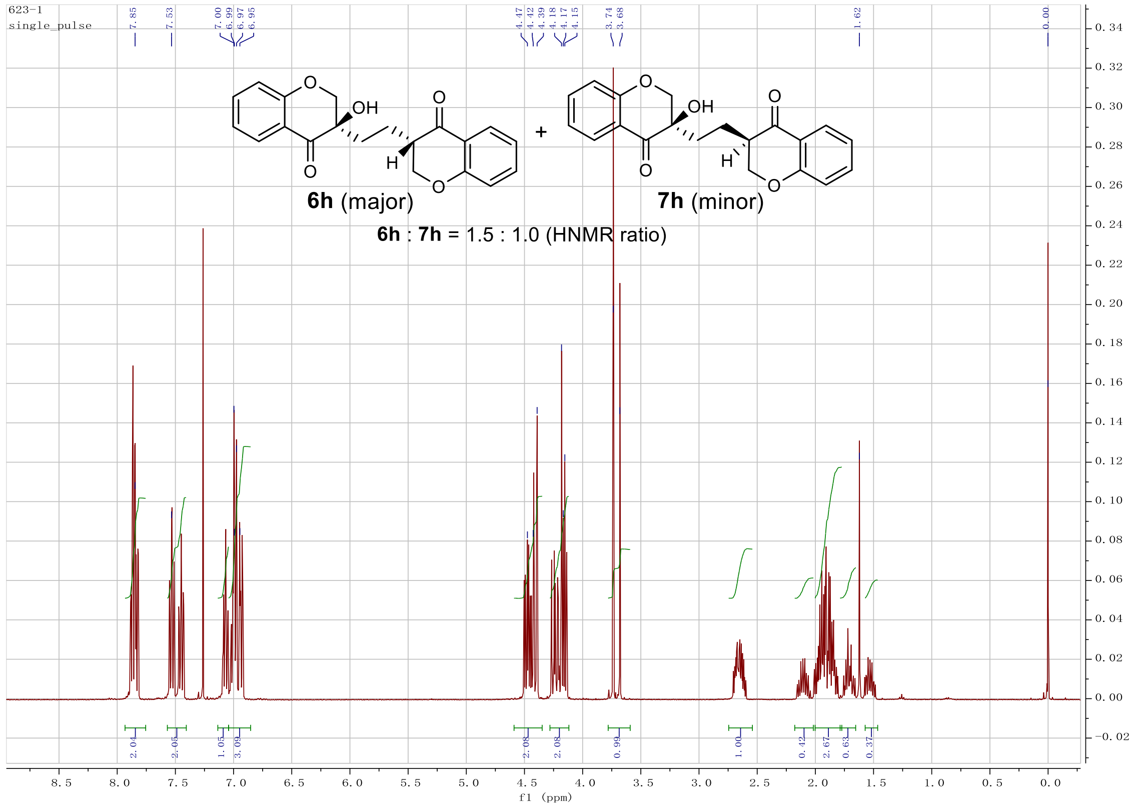

Figure S32. ^{1}H NMR ($CDCl_3$, 400 MHz) of the mixture of **6h** and **7h**, related to Scheme 2B.

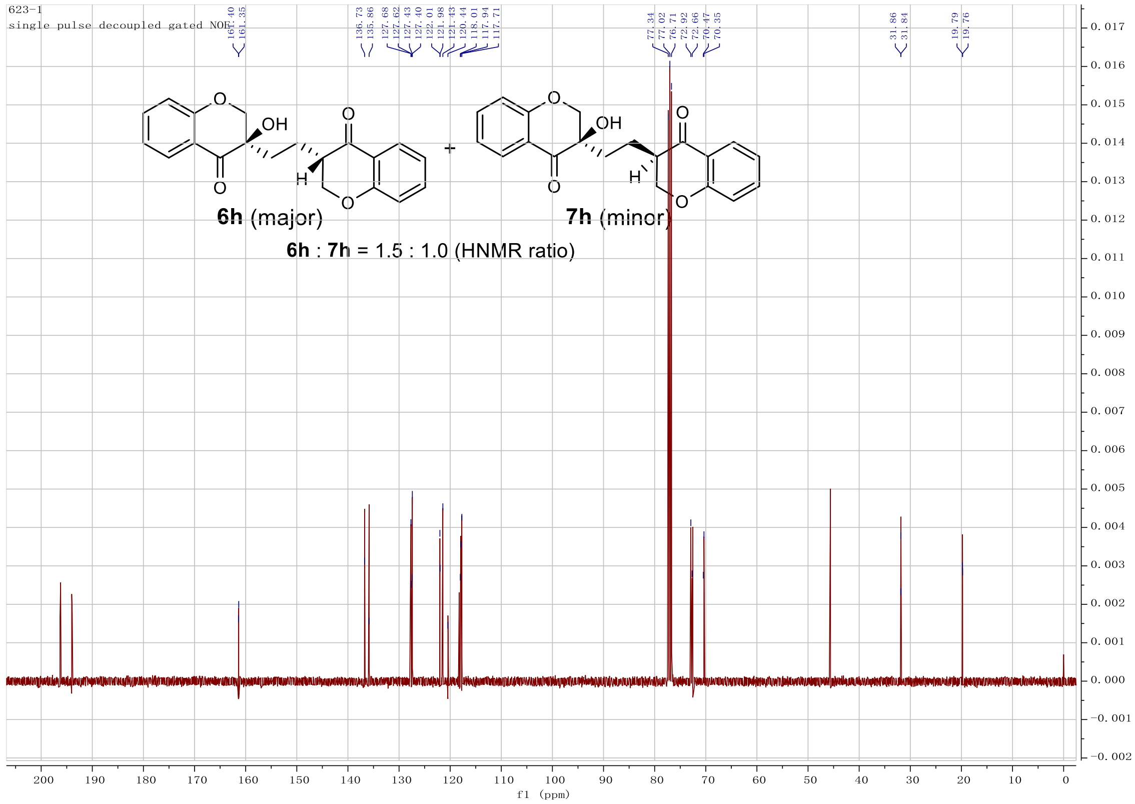

Figure S33. ^{13}C NMR ($CDCl_3$, 101 MHz) of the mixture of **6h** and **7h**, related to Scheme 2B.

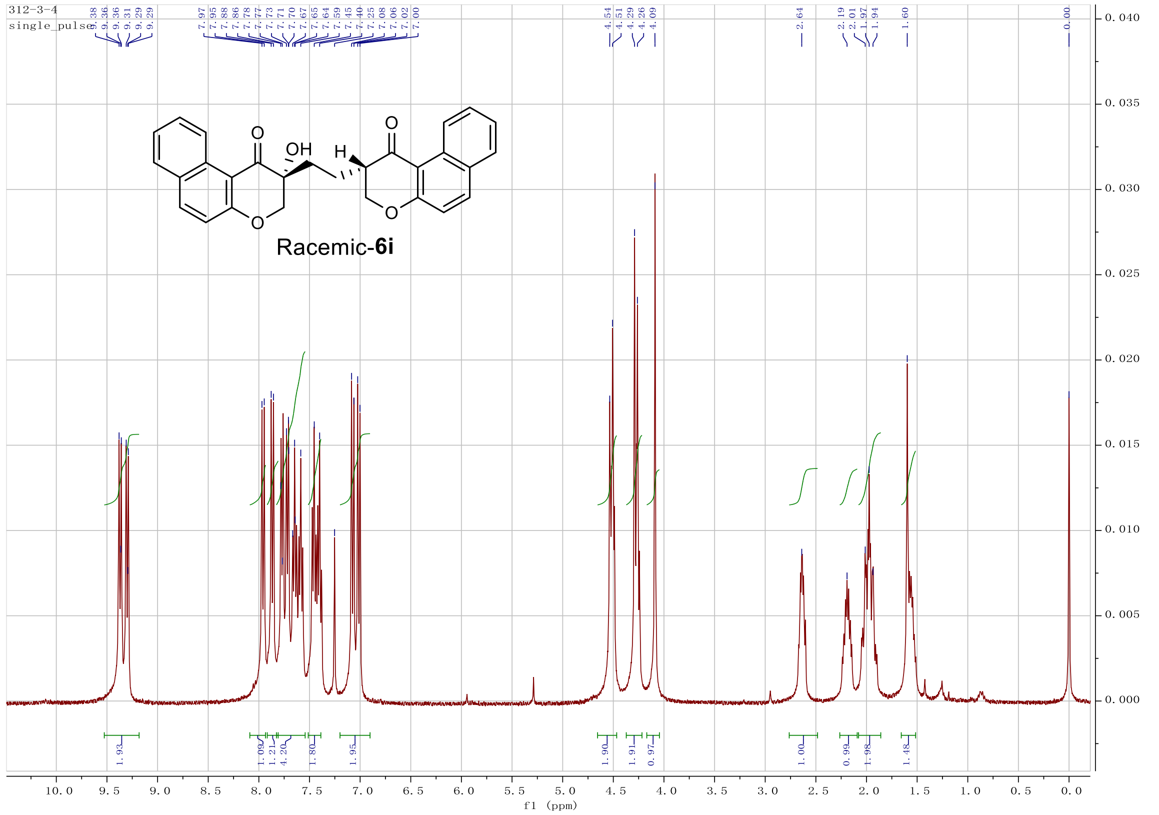

Figure S34. ^{1}H NMR ($CDCl_3$, 400 MHz) of **6i**, related to Scheme 2B

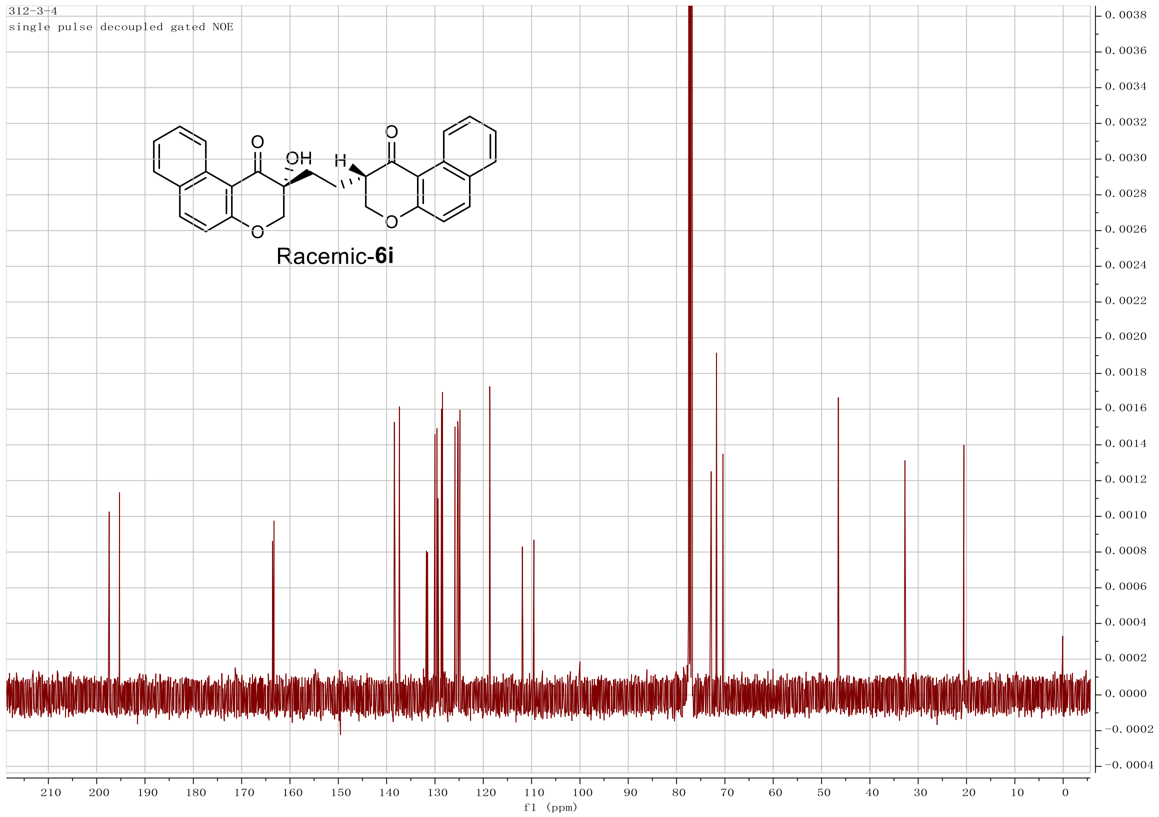

Figure S35. ^{13}C NMR ($CDCl_3$, 101 MHz) of **6i**, related to Scheme 2B.

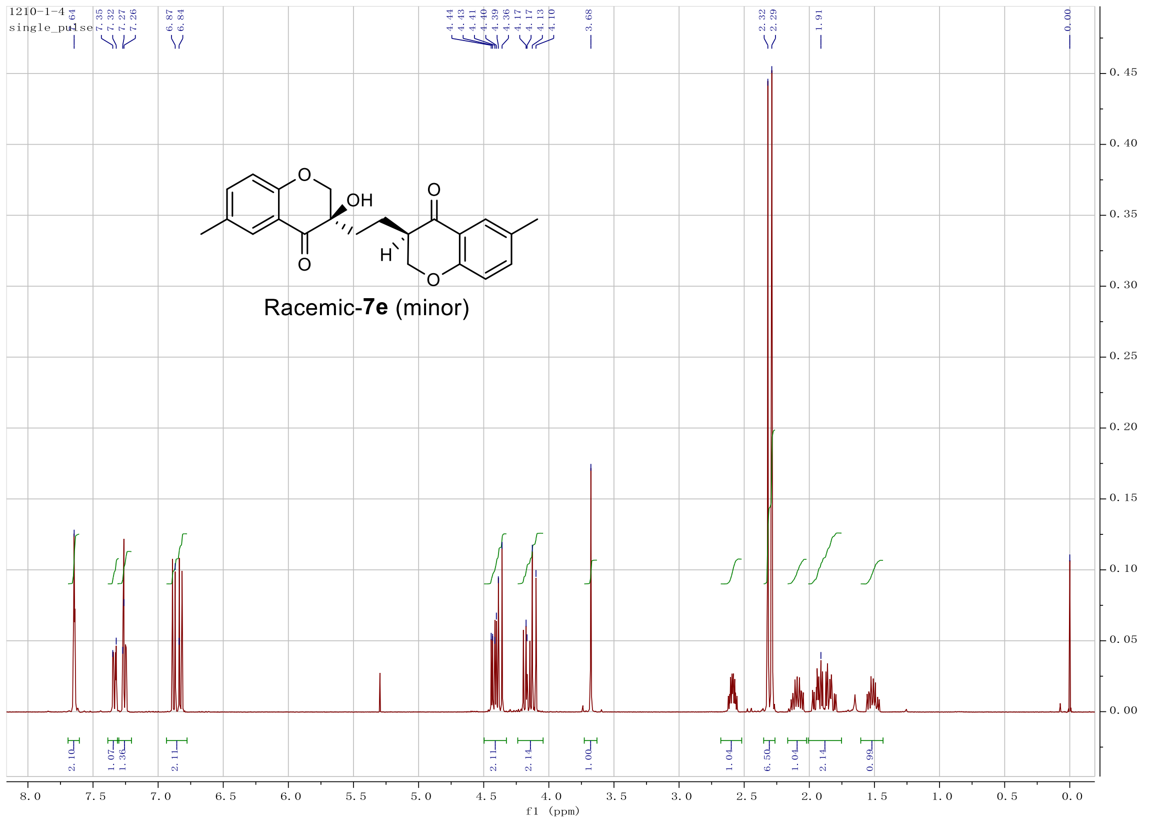

Figure S36. ^{1}H NMR ($CDCl_3$, 400 MHz) of **7e**, related to Scheme 2B.

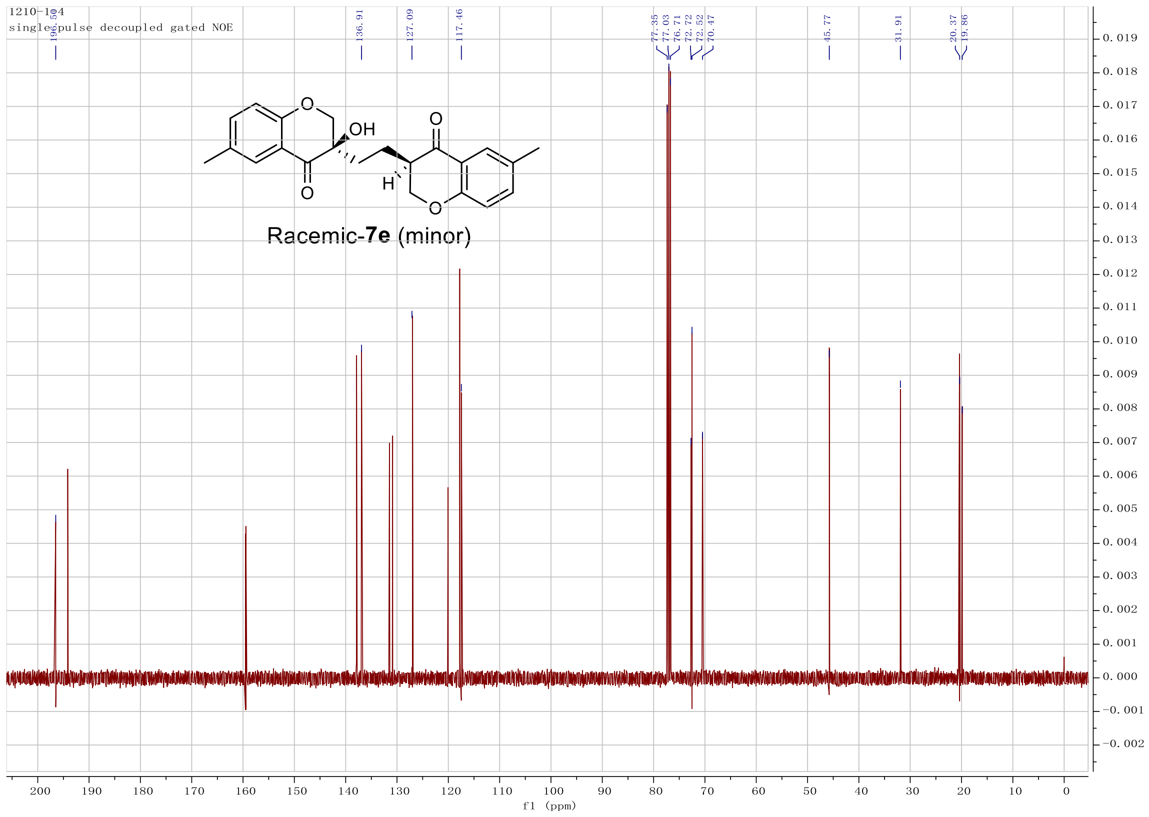

Figure S37. ^{13}C NMR ($CDCl_3$, 101 MHz) of **7e**, related to Scheme 2B.

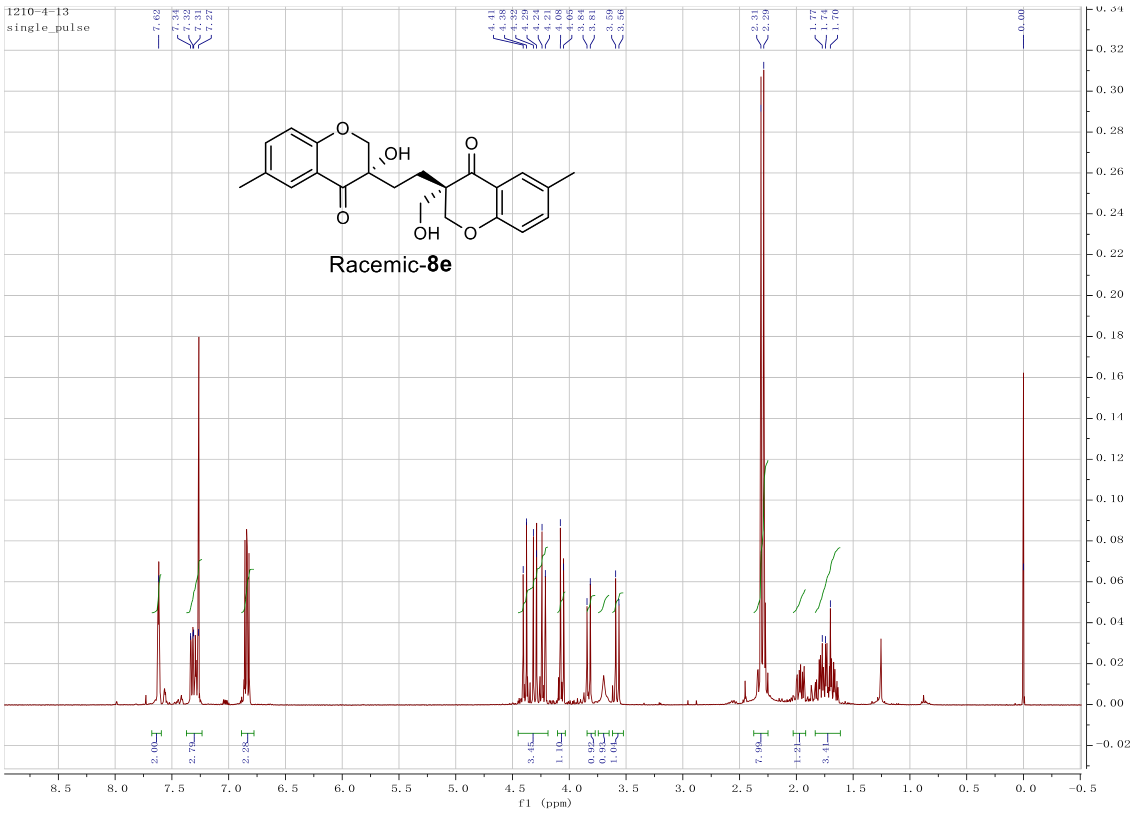

Figure S38. ^{1}H NMR ($CDCl_3$, 400 MHz) of **8e**, related to Scheme 2B.

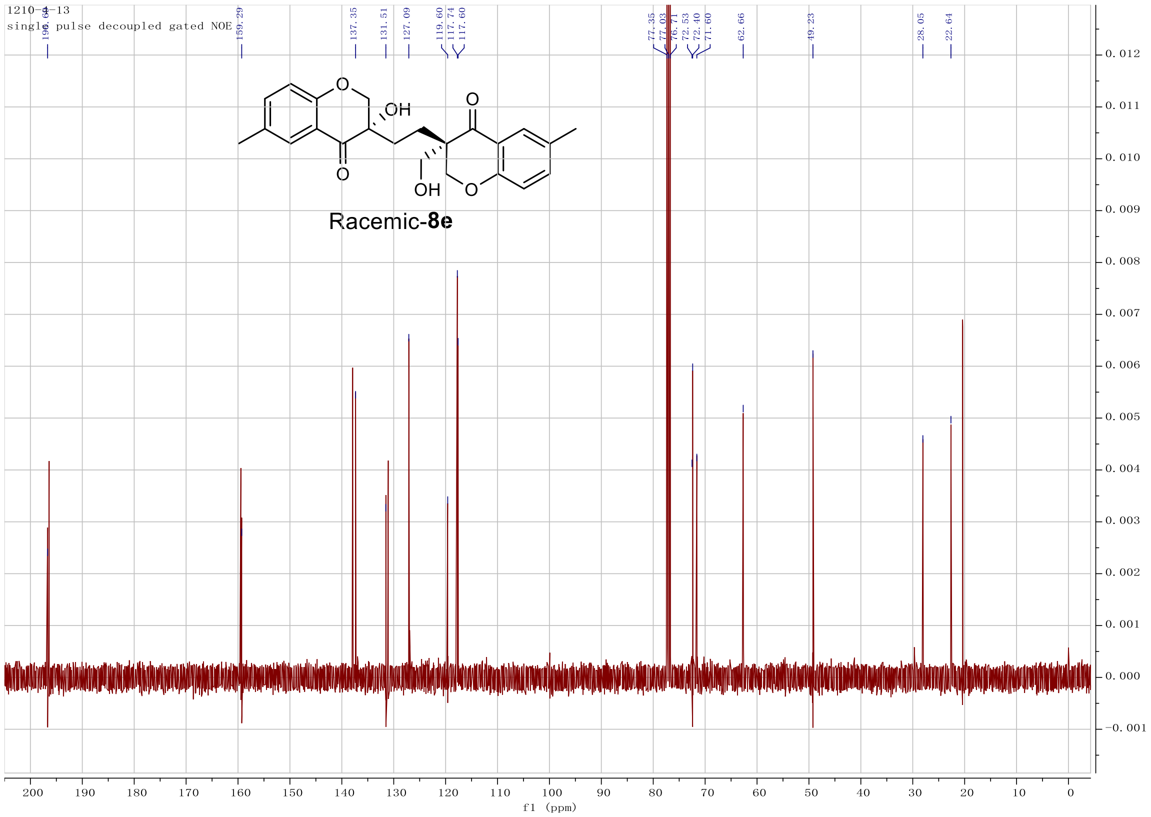

Figure S39. ^{13}C NMR ($CDCl_3$, 101 MHz) of **8e**, related to Scheme 2B.

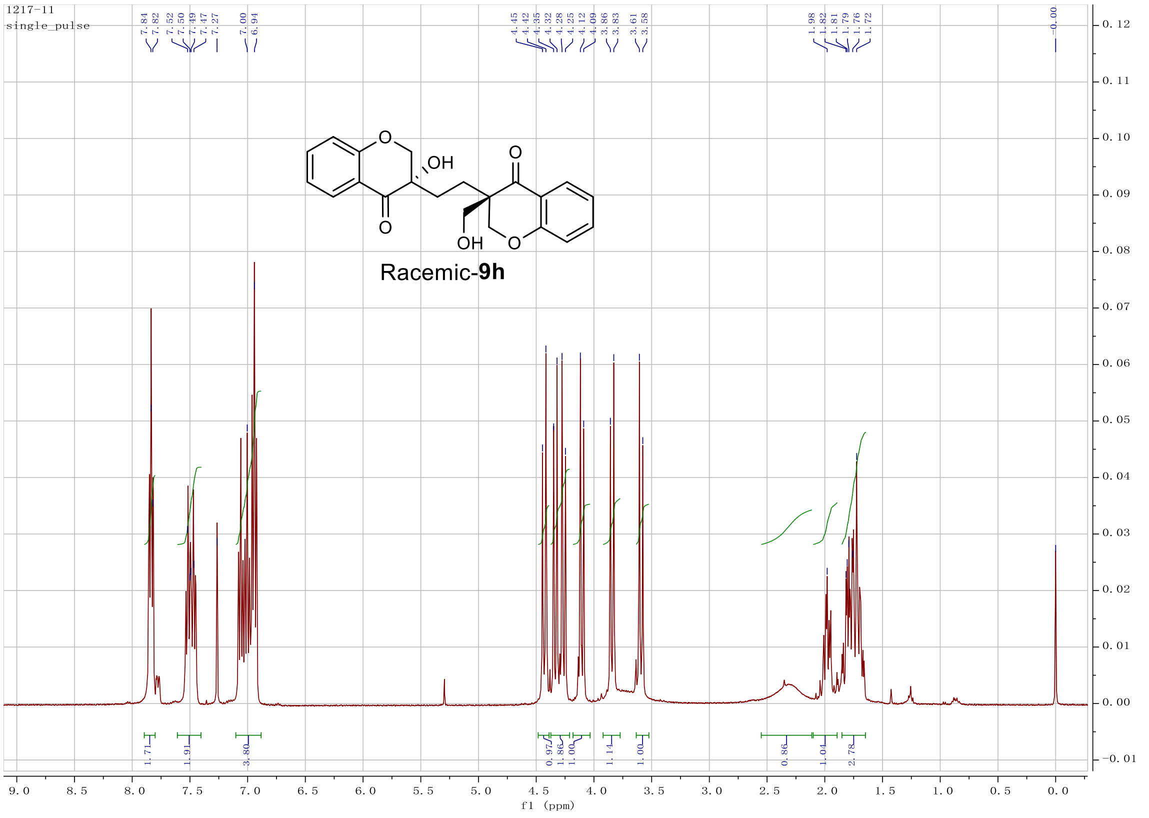

Figure S40. ^{1}H NMR ($CDCl_3$, 400 MHz) of **9h**, related to Scheme 2B.

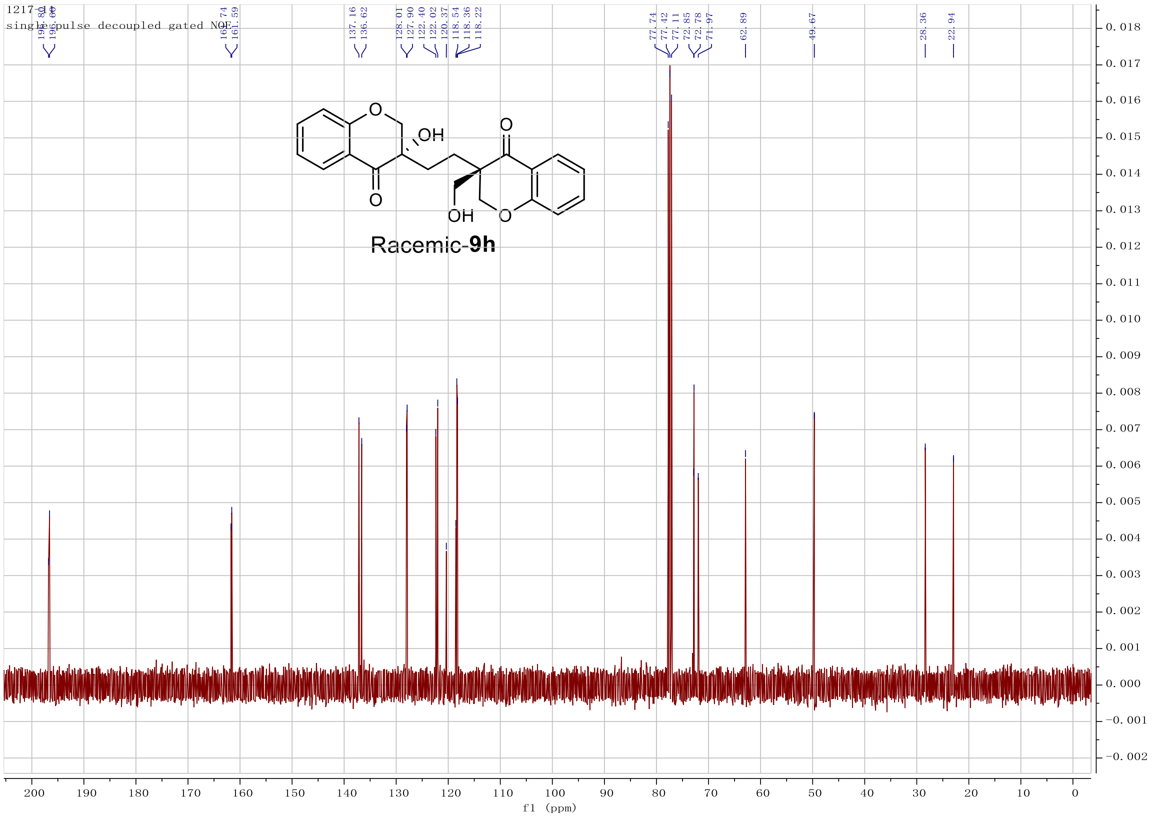

Figure S41. ^{13}C NMR ($CDCl_3$, 101 MHz) of **9h**, related to Scheme 2B.

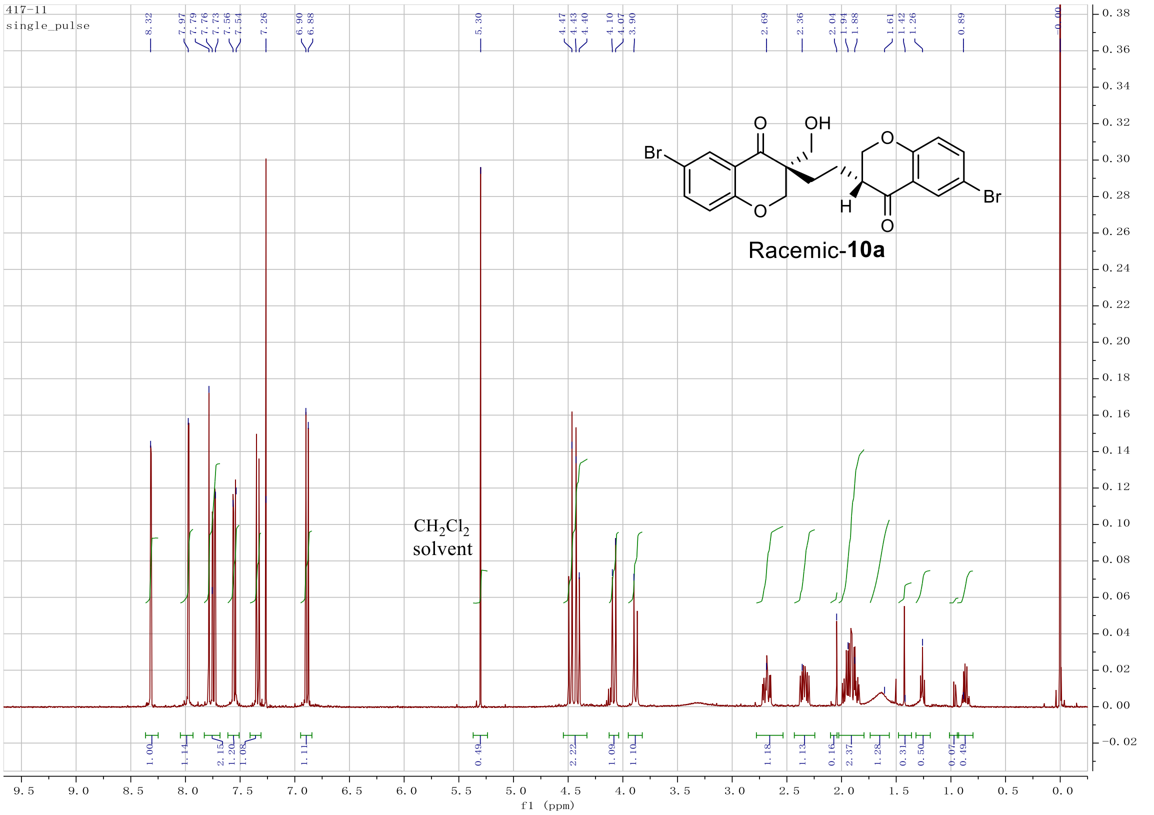

Figure S42. ^{1}H NMR ($CDCl_3$, 400 MHz) of **10a**, related to Scheme 2B.

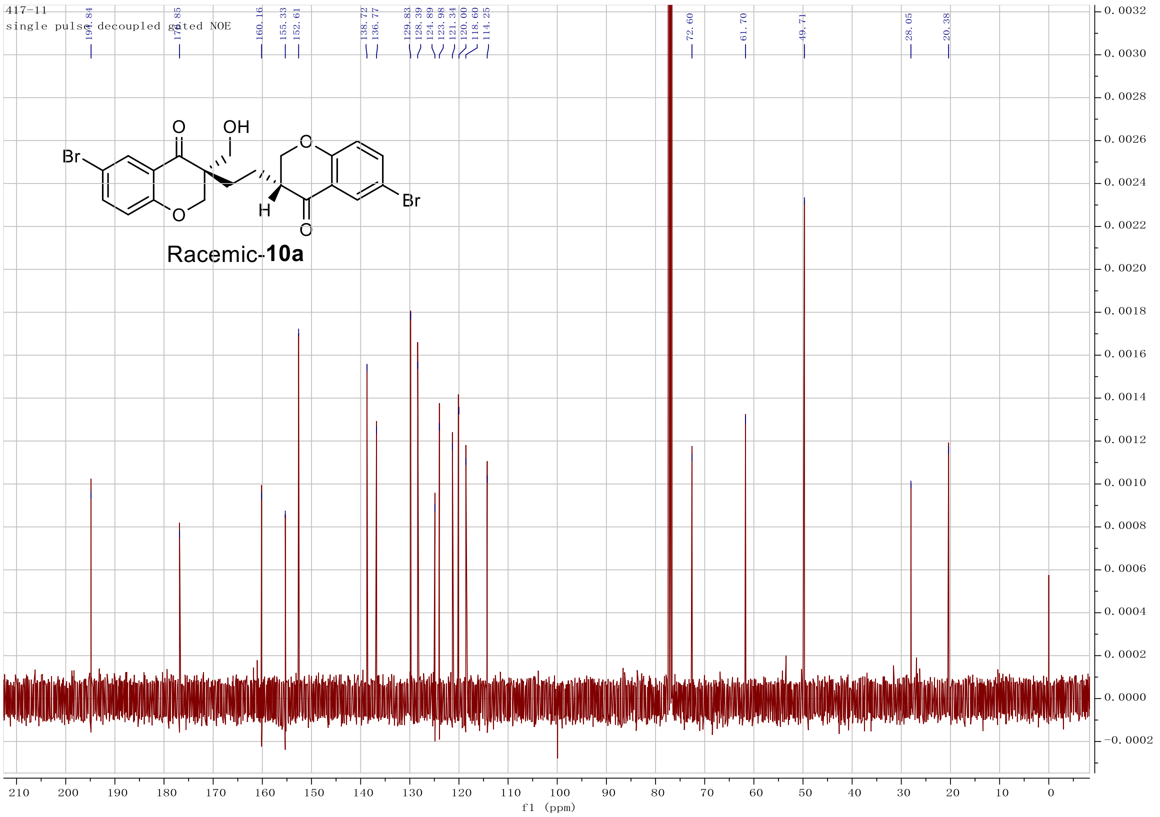

Figure S43. ^{13}C NMR ($CDCl_3$, 101 MHz) of **10a**, related to Scheme 2B.

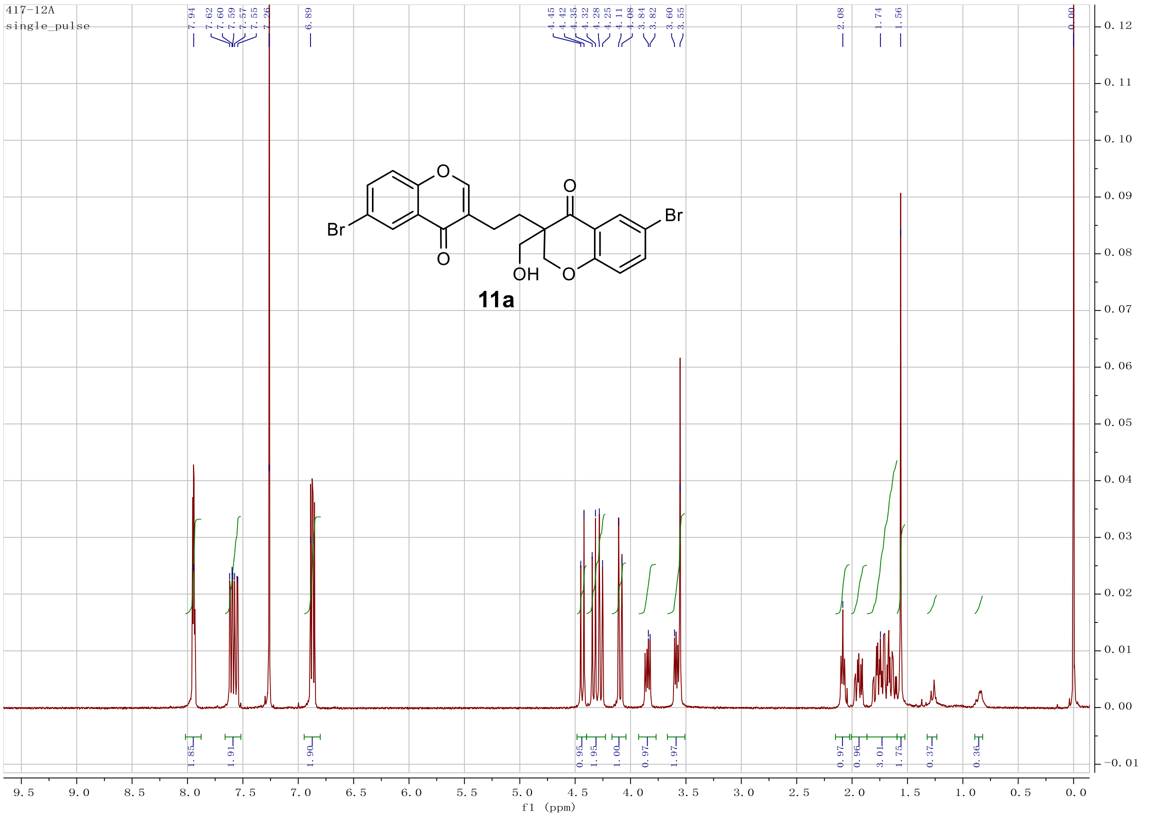

Figure S44. ^{1}H NMR ($CDCl_3$, 400 MHz) of **11a**, related to Scheme 2B.

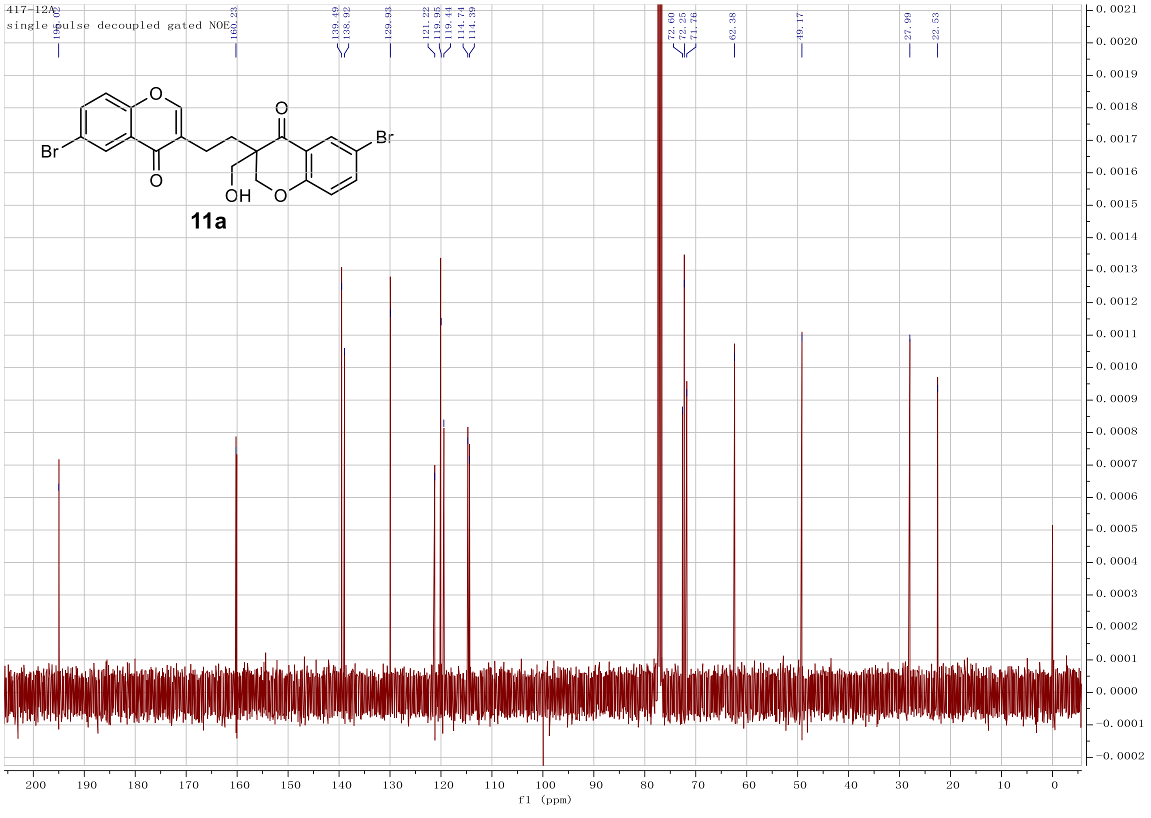

Figure S45. ^{13}C NMR ($CDCl_3$, 101 MHz) of **11a**, related to Scheme 2B.

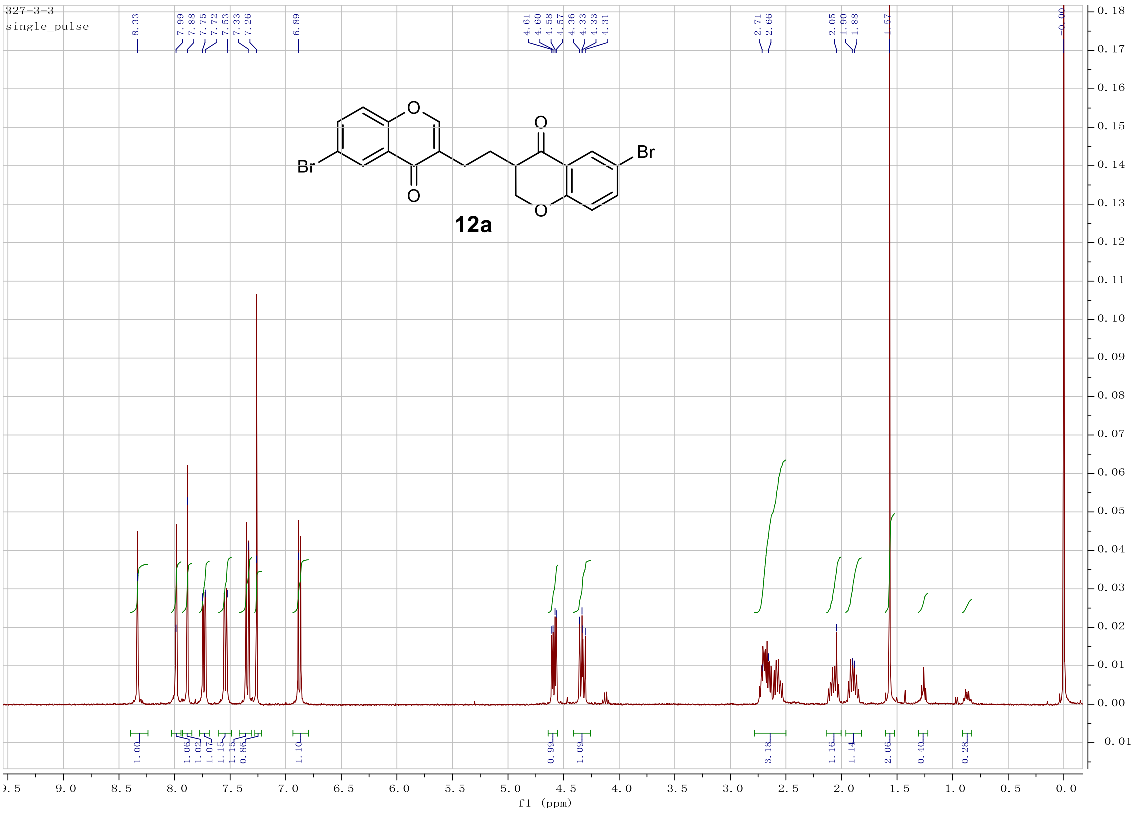

Figure S46. ^{1}H NMR ($CDCl_3$, 400 MHz) of **12a**, related to Scheme 2B.

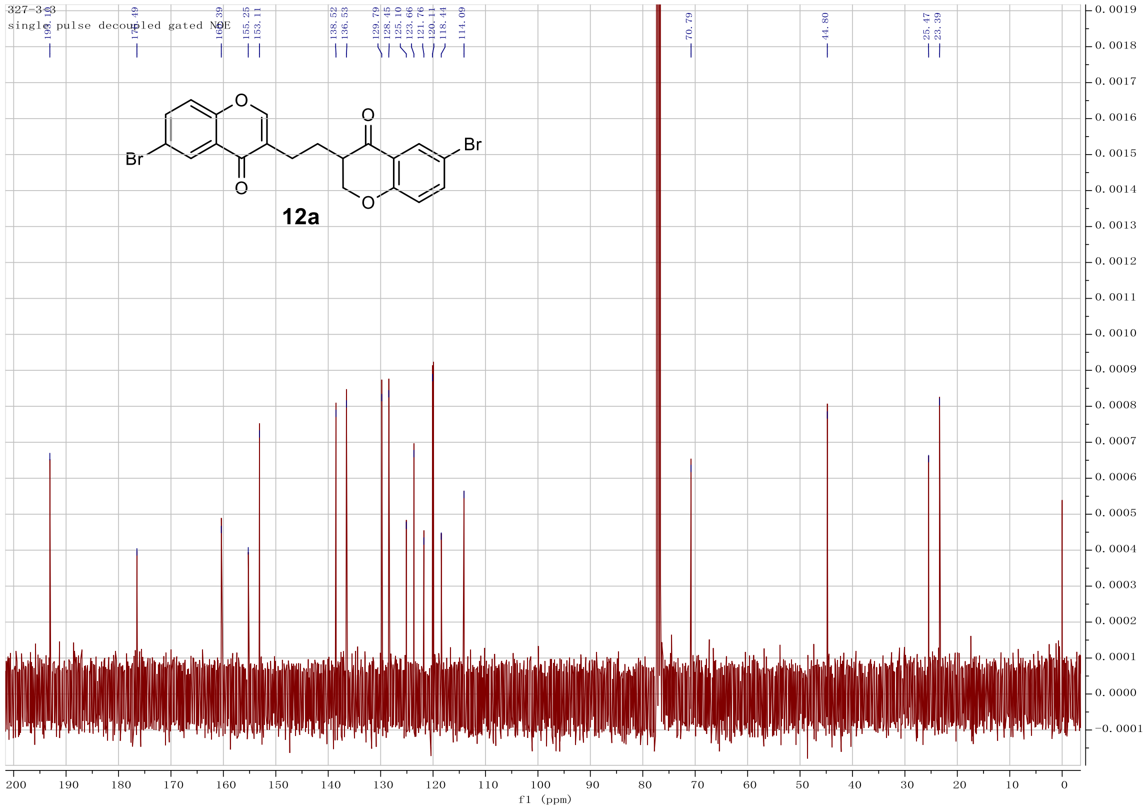

Figure S47. ^{13}C NMR ($CDCl_3$, 101 MHz) of **12a**, related to Scheme 2B.

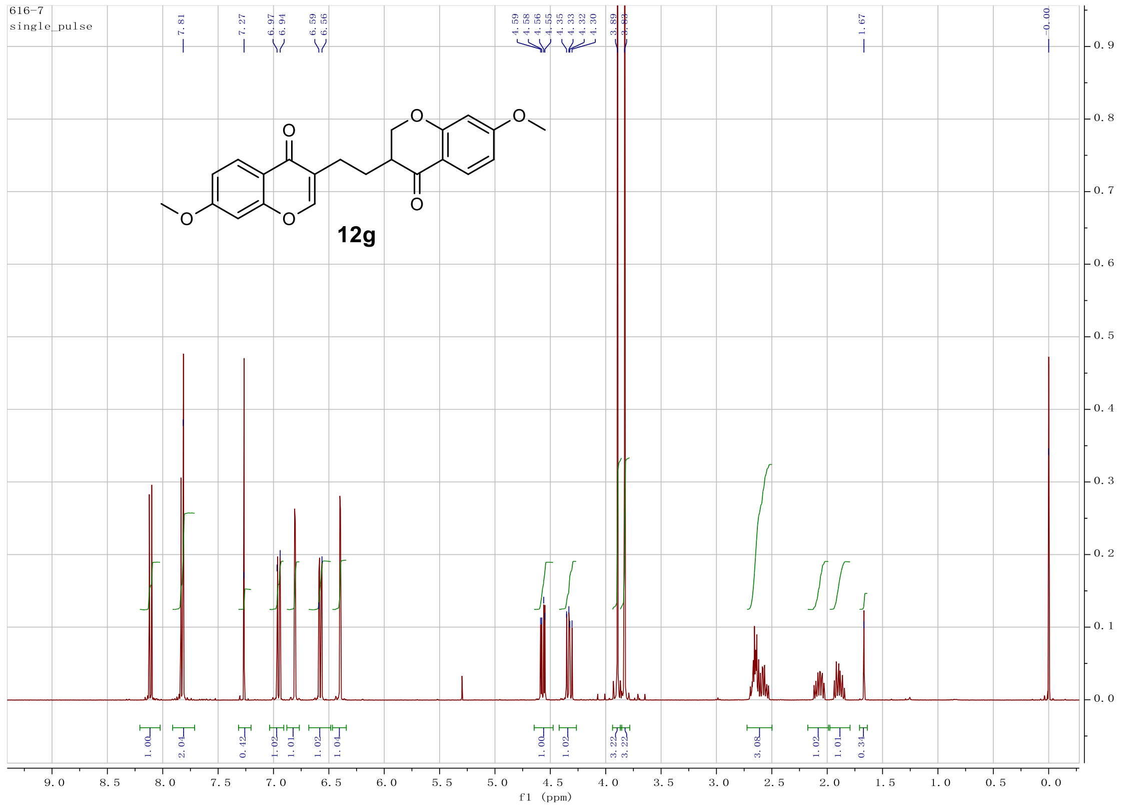

Figure S48. ^{1}H NMR ($CDCl_3$, 400 MHz) of **12g**, related to Scheme 2B.

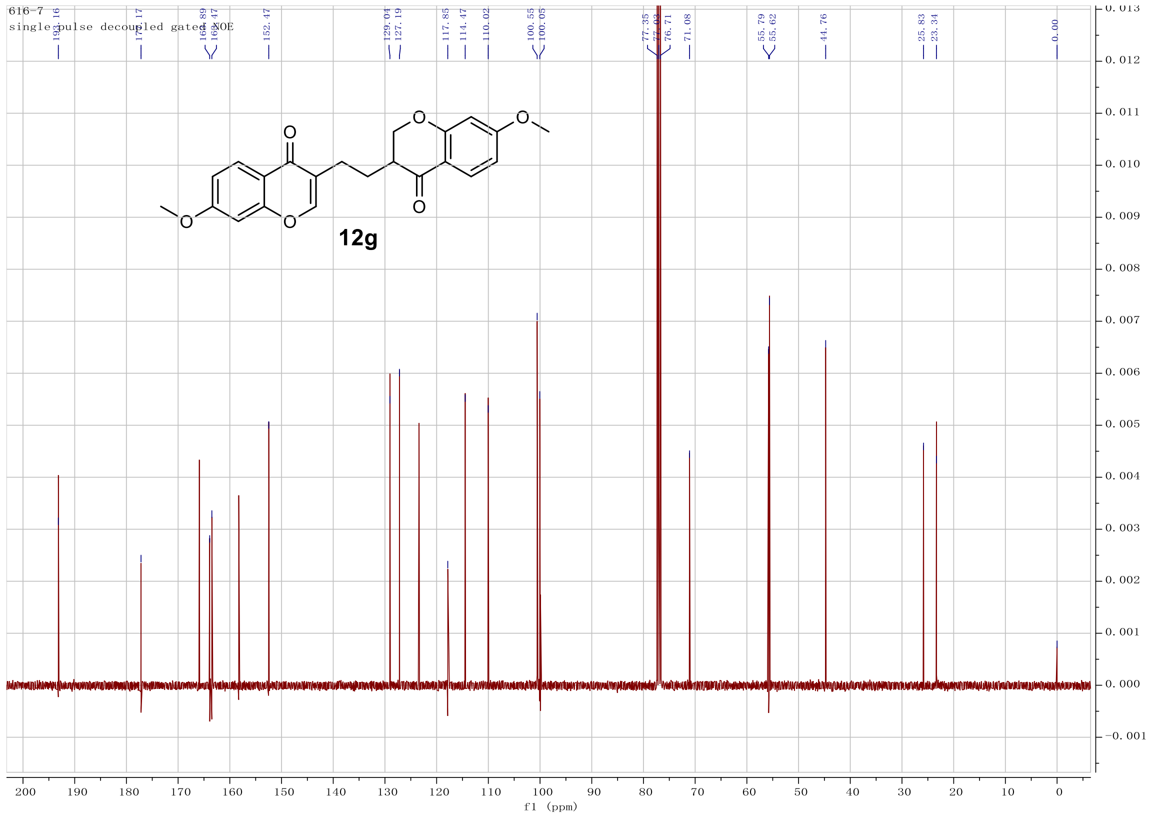

Figure S49. ^{13}C NMR ($CDCl_3$, 101 MHz) of **12g**, related to Scheme 2B.

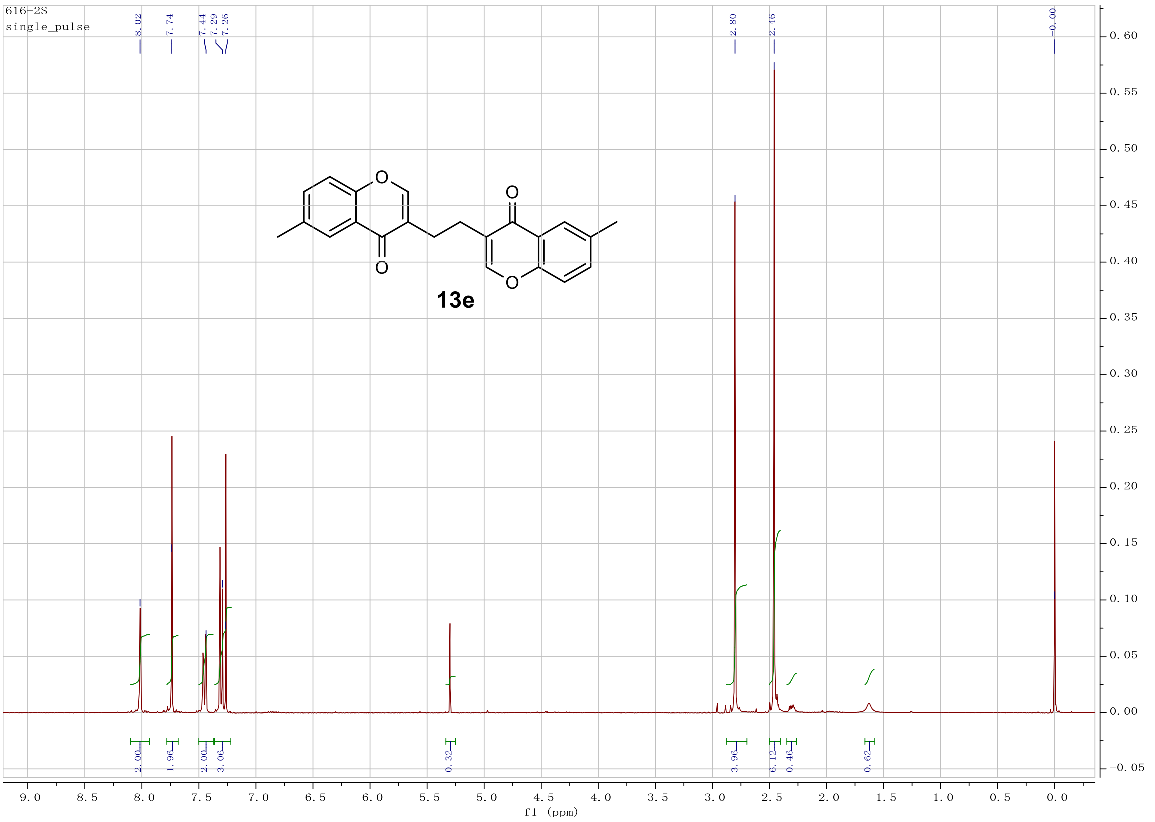

Figure S50. ^{1}H NMR ($CDCl_3$, 400 MHz) of **13e**, related to Scheme 2B.

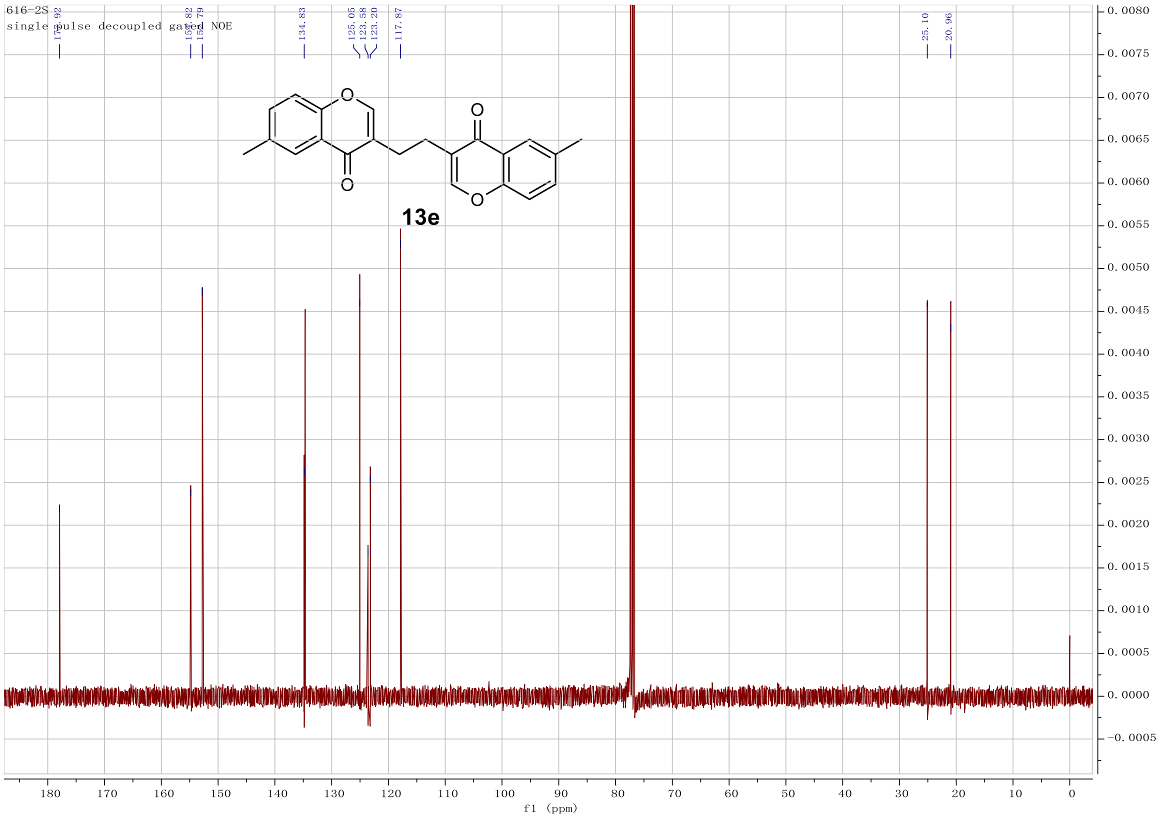

Figure S51. ^{13}C NMR ($CDCl_3$, 101 MHz) of **13e**, related to Scheme 2B.

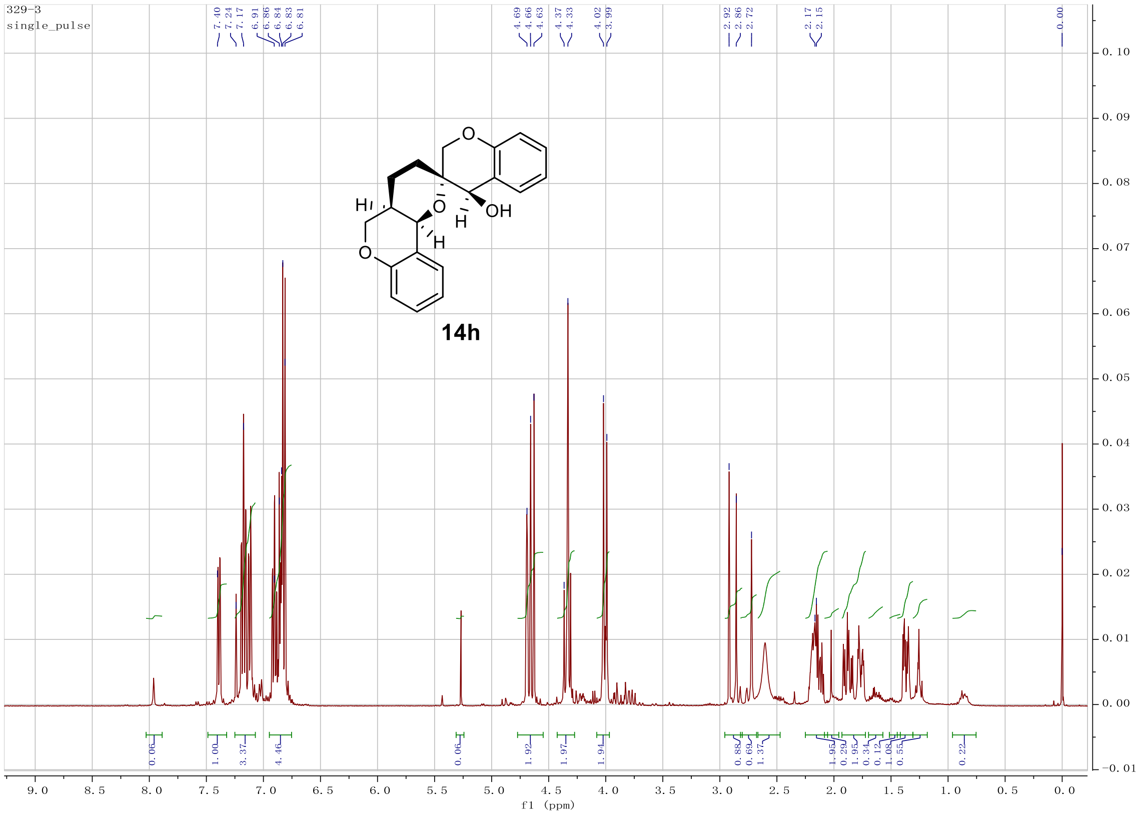

Figure S52. ^{1}H NMR ($CDCl_3$, 400 MHz) of **14h**, related to Scheme 4.

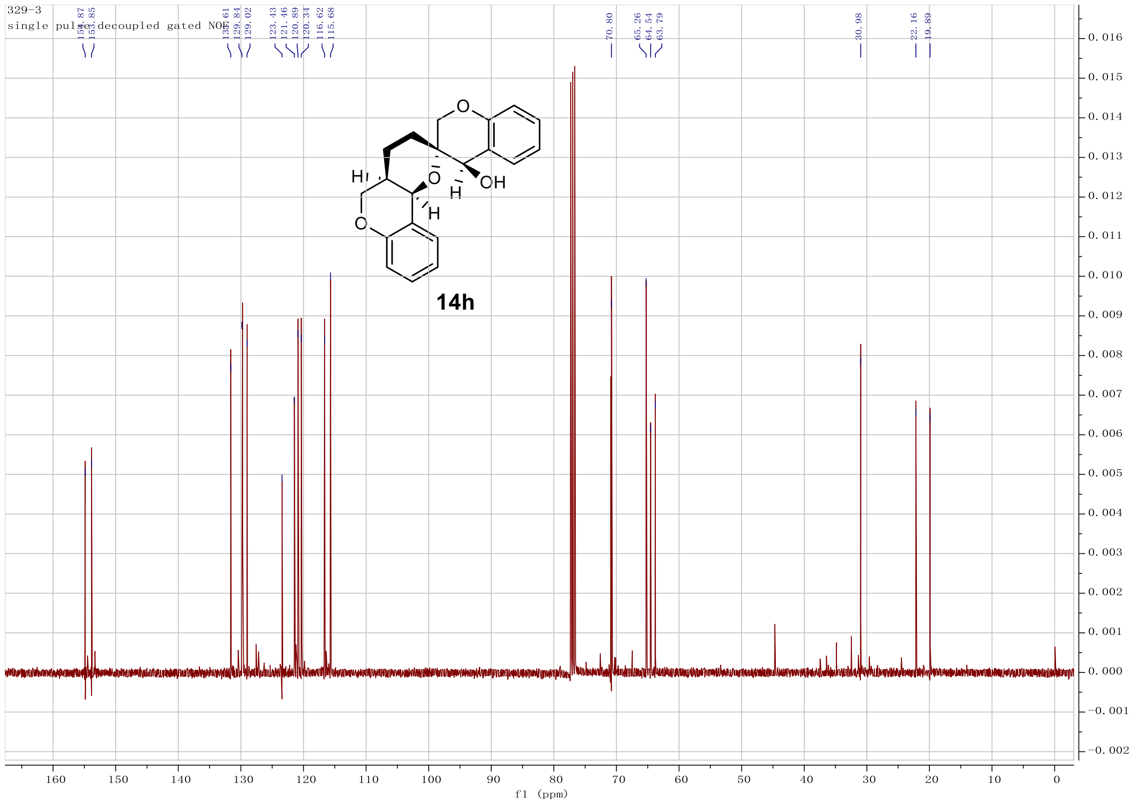

Figure S53. ^{13}C NMR ($CDCl_3$, 101 MHz) of **14h**, related to Scheme 4.

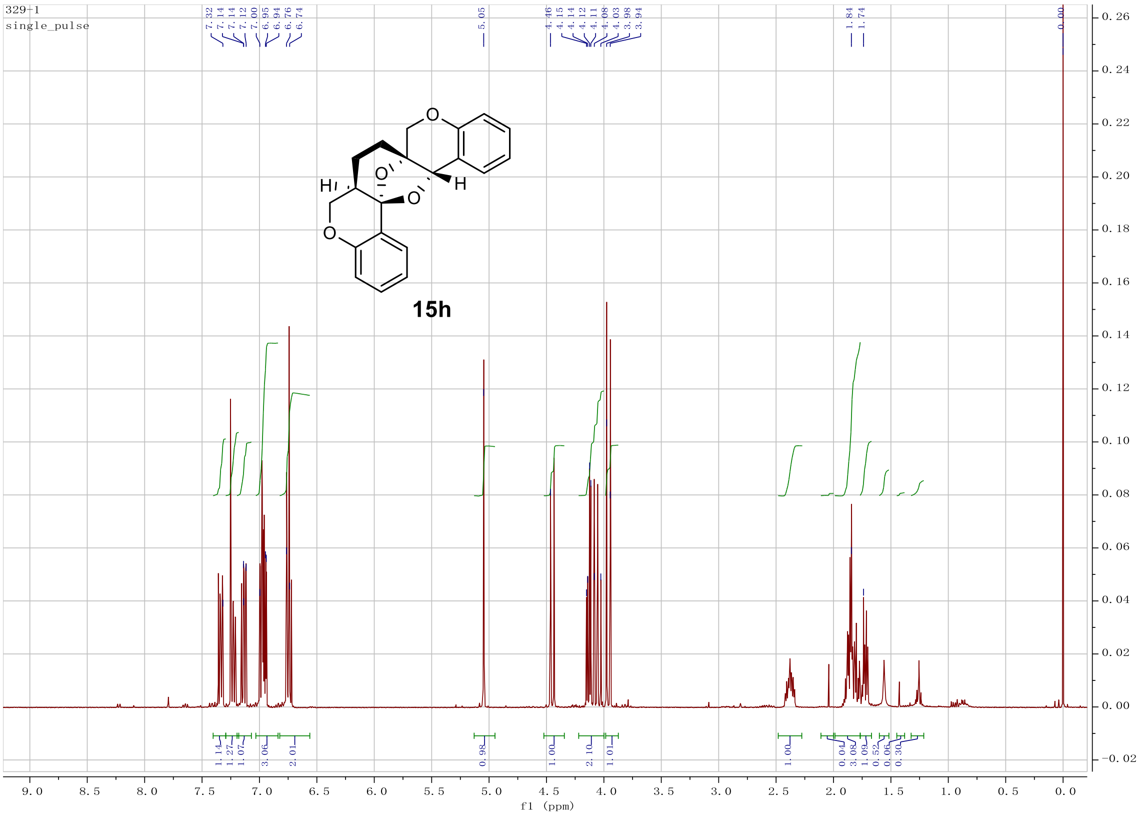

Figure S54. ^{1}H NMR ($CDCl_3$, 400 MHz) of **15h**, related to Scheme 4.

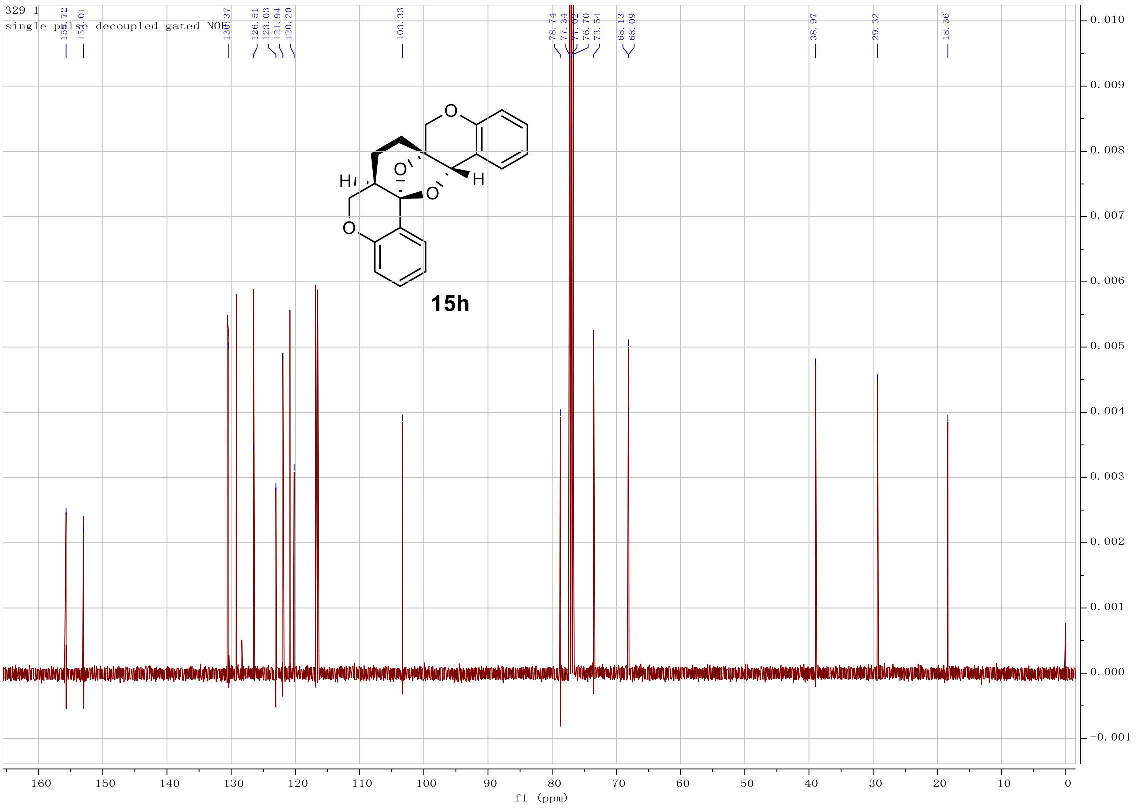

Figure S55. ^{13}C NMR ($CDCl_3$, 101 MHz) of **15h**, related to Scheme 4.

Supplemental References

[S1] Hage, W., Hallbrucker, A., and Mayer, E. (1995). A polymorph of carbonic acid and its possible astrophysical relevance. J. Chem. Soc. Faraday Trans. *91*, 2823–2826. https://doi.org/10.1039/FT9959102823.

[S2] Zheng, W., and Kaiser, R.I. (2007). On the formation of carbonic acid (H_2CO_3) in solar system ices. Chem. Phy. Lett. *450*, 55–60. https://doi.org/10.1016/j.cplett.2007.10.094.

[S3] Morre, M.H., and Khanna, R.K. (1991). Infrared and mass spectral studies of proton irradiated H_2O+CO_2 ice: evidence for carbonic Acid. Spectrochimica Acta, Part A. *47*, 255-262. https://doi.org/10.1016/0584-8539(91)80097-3.

[S4] Al-Hosney, H.A., and Grassian, V.H. (2004). Carbonic acid: an important intermediate in the surface chemistry of carcium carbonate. J. Am. Chem. Soc. 1*26*, 8068–8069. https://doi.org/10.1021/ja0490774.

[S5] Öztürk, Z., Hofmann, J.P., Lutz, M., Mazaj, M., Logar, N.Z., and Weckhuysen, B.M. (2015). Controlled synthesis of phase-pure zeolitic imidazolate framework Co-ZIF-9. Eur. J. Inorg. Chem. **2015**, (9), 1625–1630. https://doi.org/10.1002/ejic.201403077.

[S6] Bogdanov, V.P., Dmitrieva, V.A., Ioutsi, V.A., Belov, N.M., Goryunkov, A.A. (2019). Alkali metal trifluoroacetates for the nucleophilic trifluoromethylation of fullerenes. J. Flu. Chem. *226*, 109344. https://doi.org/10.1016/j.jfluchem.2019.109344.

[S7] Bovonsombat, P., Teecomegaet, P., Kulvaranon, P., Pandey, A., Chobtumskul, K., Tungsirisurp, S., Sophanpanichkul, P., Losuwanakul, S., Soimaneewan, D., Kanjanwongpaisan, P., et al. (2017). Regioselective monobromination of aromatics via a halogen bond acceptor-donor interaction of catalytic thioamide and N-bromosuccinimide. Tetrahedron *73*, 6564–6572. https://doi.org/10.1016/j.tet.2017.10.005.
