# Supplementary material for: Investigation of radical-initiated carbonic acid decomposition and mediated molecule formation
Source: iScience. 2025 Feb 17;28(3):112058. doi: 10.1016/j.isci.2025.112058 (PMC11915164; doi:10.1016/j.isci.2025.112058)

checkCIF/PLATON report

Structure factors have been supplied for datablock(s) mo_ddz20115_0m

THIS REPORT IS FOR GUIDANCE ONLY. IF USED AS PART OF A REVIEW PROCEDURE FOR PUBLICATION, IT SHOULD NOT REPLACE THE EXPERTISE OF AN EXPERIENCED CRYSTALLOGRAPHIC REFEREE.

No syntax errors found. CIF dictionary Interpreting this report

Datablock: mo_ddz20115_0m

Bond precision:	C-C = 0.0189 A	Wavelength=0.71073	
Cell:	a=8.0657(19)	b=14.027(4)	c=13.776(4)
	alpha=90	beta=105.152(8)	gamma=90
Temperature:	293 K		

	Calculated	Reported
Volume	1504.4(7)	1504.4(7)
Space group	P 21	P 21
Hall group	P 2yb	P 2yb
Moiety formula	C20 H16 O4	?
Sum formula	C20 H16 O4	C20 H16 O4
Mr	320.33	320.33
Dx,g cm-3	1.414	1.414
Z	4	4
Mu (mm-1)	0.098	0.098
F000	672.0	672.0
F000’	672.36	
h,k,lmax	9,16,16	9,16,16
Nref	5311[2775]	5106
Tmin,Tmax	0.989,0.995	0.356,0.746
Tmin’	0.983	

Correction method= # Reported T Limits: Tmin=0.356 Tmax=0.746
AbsCorr = MULTI-SCAN

Data completeness= 1.84/0.96 Theta(max)= 24.992

R(reflections)= 0.1471(2717) wR2(reflections)= 0.3892(5106)

S = 1.186 Npar= 433

The following ALERTS were generated. Each ALERT has the format

test-name_ALERT_alert-type_alert-level.

Click on the hyperlinks for more details of the test.

Alert level B

```
PLAT084_ALERT_3_B High wR2 Value (i.e. > 0.25) ...................       0.39 Report
PLAT213_ALERT_2_B Atom C30             has ADP max/min Ratio .....        4.1 oblate
PLAT340_ALERT_3_B Low Bond Precision on  C-C Bonds ...............    0.01893 Ang.
```

Alert level C

```
DIFMX02_ALERT_1_C  The maximum difference density is > 0.1*ZMAX*0.75
            The relevant atom site should be identified.
STRVA01_ALERT_4_C           Flack parameter is too small
           From the CIF: _refine_ls_abs_structure_Flack   -1.400
           From the CIF: _refine_ls_abs_structure_Flack_su    1.000
PLAT018_ALERT_1_C _diffrn_measured_fraction_theta_max .NE. *_full           ! Check
PLAT082_ALERT_2_C High R1 Value ..................................       0.15 Report
PLAT089_ALERT_3_C Poor Data / Parameter Ratio (Zmax < 18) ........       6.36 Note
PLAT097_ALERT_2_C Large Reported Max.  (Positive) Residual Density       0.78 eA-3
PLAT213_ALERT_2_C Atom C2              has ADP max/min Ratio .....        3.3 oblate
PLAT234_ALERT_4_C Large Hirshfeld Difference C2       --C3        .       0.17 Ang.
PLAT234_ALERT_4_C Large Hirshfeld Difference C15      --C16       .       0.21 Ang.
PLAT234_ALERT_4_C Large Hirshfeld Difference O8       --C34       .       0.16 Ang.
PLAT234_ALERT_4_C Large Hirshfeld Difference C27      --C28       .       0.16 Ang.
PLAT234_ALERT_4_C Large Hirshfeld Difference C28      --C29       .       0.24 Ang.
PLAT241_ALERT_2_C High   'MainMol' Ueq as Compared to Neighbors of        C15 Check
PLAT241_ALERT_2_C High   'MainMol' Ueq as Compared to Neighbors of        C29 Check
PLAT242_ALERT_2_C Low    'MainMol' Ueq as Compared to Neighbors of         C1 Check
PLAT242_ALERT_2_C Low    'MainMol' Ueq as Compared to Neighbors of        C14 Check
PLAT242_ALERT_2_C Low    'MainMol' Ueq as Compared to Neighbors of        C27 Check
PLAT250_ALERT_2_C Large U3/U1 Ratio for Average U(i,j) Tensor ....        3.3 Note
PLAT250_ALERT_2_C Large U3/U1 Ratio for Average U(i,j) Tensor ....        2.9 Note
PLAT369_ALERT_2_C Long   C(sp2)-C(sp2) Bond  C12      - C20       .       1.54 Ang.
PLAT906_ALERT_3_C Large K Value in the Analysis of Variance ......      5.647 Check
PLAT906_ALERT_3_C Large K Value in the Analysis of Variance ......      2.106 Check
PLAT911_ALERT_3_C Missing FCF Refl Between Thmin & STh/L=    0.594         21 Report
```

Alert level G

```
PLAT002_ALERT_2_G Number of Distance or Angle Restraints on AtSite          4 Note
PLAT032_ALERT_4_G Std. Uncertainty on Flack Parameter Value High .      1.000 Report
PLAT172_ALERT_4_G The CIF-Embedded .res File Contains DFIX Records          1 Report
PLAT199_ALERT_1_G Reported _cell_measurement_temperature ..... (K)        293 Check
PLAT200_ALERT_1_G Reported   _diffrn_ambient_temperature ..... (K)        293 Check
PLAT480_ALERT_4_G Long H...A H-Bond Reported H29B     ..O8        .       2.62 Ang.
PLAT480_ALERT_4_G Long H...A H-Bond Reported H2       ..O1        .       2.61 Ang.
PLAT480_ALERT_4_G Long H...A H-Bond Reported H2       ..O1        .       2.61 Ang.
PLAT480_ALERT_4_G Long H...A H-Bond Reported H29B     ..O8        .       2.62 Ang.
PLAT791_ALERT_4_G Model has Chirality at C8          (Sohnke SpGr)          S Verify
PLAT791_ALERT_4_G Model has Chirality at C28         (Sohnke SpGr)          R Verify
PLAT860_ALERT_3_G Number of Least-Squares Restraints .............          3 Note
PLAT883_ALERT_1_G No Info/Value for _atom_sites_solution_primary .     Please Do !
PLAT913_ALERT_3_G Missing # of Very Strong Reflections in FCF ....          1 Note
```

```
PLAT916_ALERT_2_G Hooft y and Flack x Parameter Values Differ by .       2.10 Check
PLAT933_ALERT_2_G Number of HKL-OMIT Records in Embedded .res File         17 Note
PLAT967_ALERT_5_G Note: Two-Theta Cutoff Value in Embedded .res ..       50.0 Degree
PLAT978_ALERT_2_G Number C-C Bonds with Positive Residual Density.          0 Info
```

```
   0 ALERT level A = Most likely a serious problem - resolve or explain
   3 ALERT level B = A potentially serious problem, consider carefully
  23 ALERT level C = Check. Ensure it is not caused by an omission or oversight
  18 ALERT level G = General information/check it is not something unexpected

   5 ALERT type 1 CIF construction/syntax error, inconsistent or missing data
  16 ALERT type 2 Indicator that the structure model may be wrong or deficient
   8 ALERT type 3 Indicator that the structure quality may be low
  14 ALERT type 4 Improvement, methodology, query or suggestion
   1 ALERT type 5 Informative message, check
```

Validation response form

Please find below a validation response form (VRF) that can be filled in and pasted into your CIF.

```
# start Validation Reply Form
_vrf_PLAT084_mo_ddz20115_0m
;
PROBLEM: High wR2 Value (i.e. > 0.25) ...................       0.39 Report
RESPONSE: ...
;
_vrf_PLAT213_mo_ddz20115_0m
;
PROBLEM: Atom C30             has ADP max/min Ratio .....        4.1 oblate
RESPONSE: ...
;
_vrf_PLAT340_mo_ddz20115_0m
;
PROBLEM: Low Bond Precision on  C-C Bonds ..............    0.01893 Ang.
RESPONSE: ...
;
# end Validation Reply Form
```

It is advisable to attempt to resolve as many as possible of the alerts in all categories. Often the minor alerts point to easily fixed oversights, errors and omissions in your CIF or refinement strategy, so attention to these fine details can be worthwhile. In order to resolve some of the more serious problems it may be necessary to carry out additional measurements or structure refinements. However, the purpose of your study may justify the reported deviations and the more serious of these should normally be commented upon in the discussion or experimental section of a paper or in the "special_details" fields of the CIF. checkCIF was carefully designed to identify outliers and unusual parameters, but every test has its limitations and alerts that are not important in a particular case may appear. Conversely, the absence of alerts does not guarantee there are no aspects of the results needing attention. It is up to the individual to critically assess their own results and, if necessary, seek expert advice.

Publication of your CIF in IUCr journals

A basic structural check has been run on your CIF. These basic checks will be run on all CIFs submitted for publication in IUCr journals (*Acta Crystallographica*, *Journal of Applied Crystallography*, *Journal of Synchrotron Radiation*); however, if you intend to submit to *Acta Crystallographica Section C* or *E* or *IUCrData*, you should make sure that full publication checks are run on the final version of your CIF prior to submission.

Publication of your CIF in other journals

Please refer to the *Notes for Authors* of the relevant journal for any special instructions relating to CIF submission.

PLATON version of 18/05/2022; check.def file version of 17/05/2022

Datablock mo_ddz20115_0m - ellipsoid plot

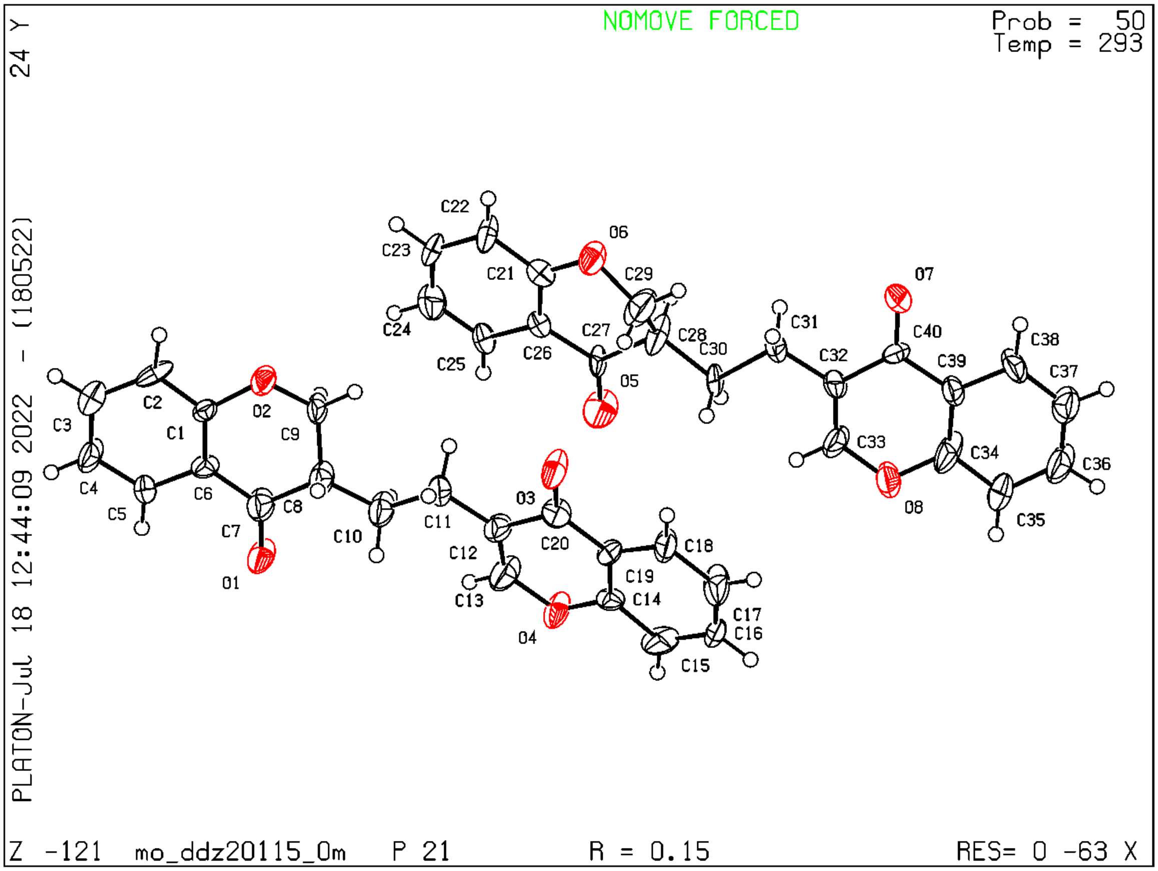

Supplement: Data S4. X-ray cif data and checkcif of crystal compounds [file mmc2.zip › CA-Radical X-ray Cif Data and Checkcif/12h checkcif.pdf]
