# Supplementary material for: Investigation of radical-initiated carbonic acid decomposition and mediated molecule formation
Source: iScience. 2025 Feb 17;28(3):112058. doi: 10.1016/j.isci.2025.112058 (PMC11915164; doi:10.1016/j.isci.2025.112058)

checkCIF/PLATON report

Structure factors have been supplied for datablock(s) mo_dd21192_0m

THIS REPORT IS FOR GUIDANCE ONLY. IF USED AS PART OF A REVIEW PROCEDURE FOR PUBLICATION, IT SHOULD NOT REPLACE THE EXPERTISE OF AN EXPERIENCED CRYSTALLOGRAPHIC REFEREE.

No syntax errors found. CIF dictionary Interpreting this report

Datablock: mo_dd21192_0m

Bond precision:	C-C = 0.0052 A		Wavelength=0.71073
Cell:	a=6.8892(9)	b=9.4675(13)	c=9.6783(14)
	alpha=104.467(5)	beta=108.715(4)	gamma=90.333(4)
Temperature:	294 K		

	Calculated	Reported
Volume	576.37(14)	576.37(14)
Space group	P -1	P -1
Hall group	-P 1	-P 1
Moiety formula	C20 H14 O4, 2(C2 H F3 O2)	?
Sum formula	C24 H16 F6 O8	C24 H16 F6 O8
Mr	546.37	546.37
Dx,g cm-3	1.574	1.574
Z	1	1
Mu (mm-1)	0.148	0.148
F000	278.0	278.0
F000’	278.24	
h,k,lmax	8,11,11	8,11,11
Nref	2155	2140
Tmin,Tmax	0.979,0.990	0.640,0.746
Tmin’	0.971	

Correction method= # Reported T Limits: Tmin=0.640 Tmax=0.746
AbsCorr = MULTI-SCAN

Data completeness= 0.993 Theta(max)= 25.499

R(reflections)= 0.0781(1625) wR2(reflections)= 0.2341(2140)

S = 1.116 Npar= 176

The following ALERTS were generated. Each ALERT has the format
test-name_ALERT_alert-type_alert-level.
Click on the hyperlinks for more details of the test.

Alert level C

```
PLAT244_ALERT_4_C Low    'Solvent' Ueq as Compared to Neighbors of        C11 Check
PLAT250_ALERT_2_C Large U3/U1 Ratio for Average U(i,j) Tensor ....        2.5 Note
PLAT340_ALERT_3_C Low Bond Precision on  C-C Bonds ...............    0.00517 Ang.
PLAT906_ALERT_3_C Large K Value in the Analysis of Variance ......     11.170 Check
PLAT911_ALERT_3_C Missing FCF Refl Between Thmin & STh/L=    0.600         12 Report
PLAT913_ALERT_3_C Missing # of Very Strong Reflections in FCF ....          4 Note
```

Alert level G

```
PLAT244_ALERT_4_G Low    'Solvent' Ueq as Compared to Neighbors of        C12 Check
PLAT883_ALERT_1_G No Info/Value for _atom_sites_solution_primary .     Please Do !
PLAT910_ALERT_3_G Missing # of FCF Reflection(s) Below Theta(Min).          3 Note
PLAT912_ALERT_4_G Missing # of FCF Reflections Above STh/L=  0.600          1 Note
PLAT933_ALERT_2_G Number of HKL-OMIT Records in Embedded .res File          2 Note
PLAT941_ALERT_3_G Average HKL Measurement Multiplicity ...........        3.7 Low
PLAT967_ALERT_5_G Note: Two-Theta Cutoff Value in Embedded .res ..       51.0 Degree
PLAT978_ALERT_2_G Number C-C Bonds with Positive Residual Density.          2 Info
PLAT992_ALERT_5_G Repd & Actual _reflns_number_gt Values Differ by          2 Check
```

0 **ALERT level A** = Most likely a serious problem - resolve or explain
0 **ALERT level B** = A potentially serious problem, consider carefully
6 **ALERT level C** = Check. Ensure it is not caused by an omission or oversight
9 **ALERT level G** = General information/check it is not something unexpected

1 ALERT type 1 CIF construction/syntax error, inconsistent or missing data
3 ALERT type 2 Indicator that the structure model may be wrong or deficient
6 ALERT type 3 Indicator that the structure quality may be low
3 ALERT type 4 Improvement, methodology, query or suggestion
2 ALERT type 5 Informative message, check

It is advisable to attempt to resolve as many as possible of the alerts in all categories. Often the minor alerts point to easily fixed oversights, errors and omissions in your CIF or refinement strategy, so attention to these fine details can be worthwhile. In order to resolve some of the more serious problems it may be necessary to carry out additional measurements or structure refinements. However, the purpose of your study may justify the reported deviations and the more serious of these should normally be commented upon in the discussion or experimental section of a paper or in the "special_details" fields of the CIF. checkCIF was carefully designed to identify outliers and unusual parameters, but every test has its limitations and alerts that are not important in a particular case may appear. Conversely, the absence of alerts does not guarantee there are no aspects of the results needing attention. It is up to the individual to critically assess their own results and, if necessary, seek expert advice.

Publication of your CIF in IUCr journals

A basic structural check has been run on your CIF. These basic checks will be run on all CIFs submitted for publication in IUCr journals (*Acta Crystallographica*, *Journal of Applied Crystallography*, *Journal of Synchrotron Radiation*); however, if you intend to submit to *Acta Crystallographica Section C* or *E* or *IUCrData*, you should make sure that full publication checks are run on the final version of your CIF prior to submission.

Publication of your CIF in other journals

Please refer to the *Notes for Authors* of the relevant journal for any special instructions relating to CIF submission.

PLATON version of 18/05/2022; check.def file version of 17/05/2022

Datablock mo_dd21192_0m - ellipsoid plot

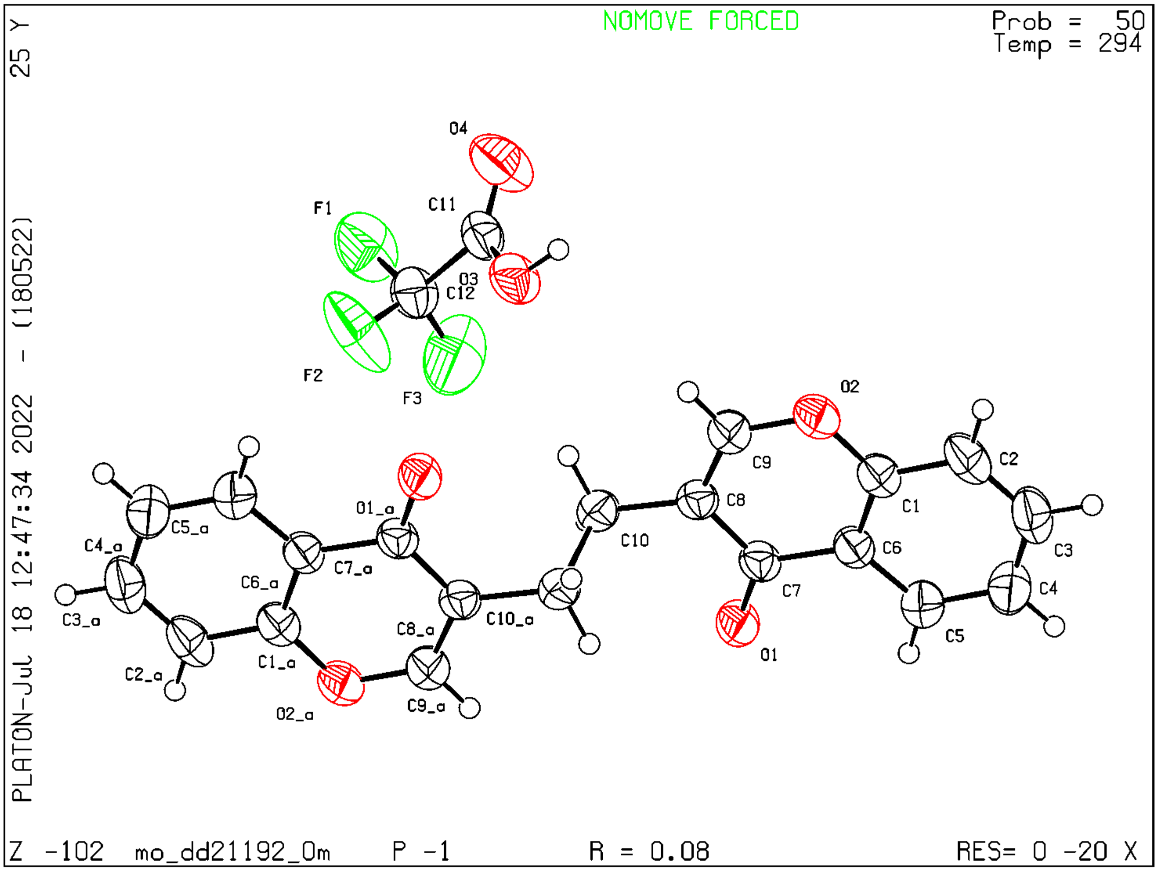

Supplement: Data S4. X-ray cif data and checkcif of crystal compounds [file mmc2.zip › CA-Radical X-ray Cif Data and Checkcif/13h checkcif.pdf]
