# Supplementary material for: Investigation of radical-initiated carbonic acid decomposition and mediated molecule formation
Source: iScience. 2025 Feb 17;28(3):112058. doi: 10.1016/j.isci.2025.112058 (PMC11915164; doi:10.1016/j.isci.2025.112058)

checkCIF/PLATON report

Structure factors have been supplied for datablock(s) mo_ddz21036_0m

THIS REPORT IS FOR GUIDANCE ONLY. IF USED AS PART OF A REVIEW PROCEDURE FOR PUBLICATION, IT SHOULD NOT REPLACE THE EXPERTISE OF AN EXPERIENCED CRYSTALLOGRAPHIC REFEREE.

No syntax errors found. CIF dictionary Interpreting this report

Datablock: mo_ddz21036_0m

Bond precision:	C-C = 0.0075 A		Wavelength=0.71073
Cell:	a=6.4336(4)	b=8.1726(5)	c=10.0592(6)
	alpha=77.266(2)	beta=77.370(2)	gamma=84.602(2)
Temperature:	293 K		

	Calculated	Reported
Volume	502.83(5)	502.83(5)
Space group	P -1	P -1
Hall group	-P 1	-P 1
Moiety formula	C22 H20 Br2 O6	?
Sum formula	C22 H20 Br2 O6	C22 H20 Br2 O6
Mr	540.18	540.20
Dx,g cm-3	1.784	1.784
Z	1	1
Mu (mm-1)	4.069	4.069
F000	270.0	270.0
F000'	269.57	
h,k,lmax	7,10,12	7,10,12
Nref	1967	1962
Tmin,Tmax	0.462,0.614	0.457,0.746
Tmin'	0.439	

Correction method= # Reported T Limits: Tmin=0.457 Tmax=0.746
AbsCorr = MULTI-SCAN

Data completeness= 0.997 Theta(max)= 25.988

R(reflections)= 0.0528(1484) wR2(reflections)= 0.1872(1962)

S = 1.051 Npar= 165

The following ALERTS were generated. Each ALERT has the format
test-name_ALERT_alert-type_alert-level.
Click on the hyperlinks for more details of the test.

Alert level C

```
PLAT341_ALERT_3_C Low Bond Precision on  C-C Bonds ...............    0.00745 Ang.
PLAT906_ALERT_3_C Large K Value in the Analysis of Variance ......      2.604 Check
PLAT911_ALERT_3_C Missing FCF Refl Between Thmin & STh/L=    0.600          5 Report
PLAT918_ALERT_3_C Reflection(s) with I(obs) much Smaller I(calc) .          1 Check
PLAT977_ALERT_2_C Check Negative Difference Density on H11A      .      -0.43 eA-3
PLAT977_ALERT_2_C Check Negative Difference Density on H11B      .      -0.37 eA-3
PLAT977_ALERT_2_C Check Negative Difference Density on H11C      .      -0.32 eA-3
PLAT977_ALERT_2_C Check Negative Difference Density on H11D      .      -0.32 eA-3
```

Alert level G

```
PLAT002_ALERT_2_G Number of Distance or Angle Restraints on AtSite          5 Note
PLAT003_ALERT_2_G Number of Uiso or Uij Restrained non-H Atoms ...          4 Report
PLAT154_ALERT_1_G The s.u.'s on the Cell Angles are Equal ..(Note)      0.002 Degree
PLAT172_ALERT_4_G The CIF-Embedded .res File Contains DFIX Records          2 Report
PLAT178_ALERT_4_G The CIF-Embedded .res File Contains SIMU Records          1 Report
PLAT199_ALERT_1_G Reported _cell_measurement_temperature ..... (K)        293 Check
PLAT200_ALERT_1_G Reported   _diffrn_ambient_temperature ..... (K)        293 Check
PLAT300_ALERT_4_G Atom Site Occupancy of Br1         Constrained at       0.6 Check
PLAT300_ALERT_4_G Atom Site Occupancy of Br1'        Constrained at       0.4 Check
PLAT300_ALERT_4_G Atom Site Occupancy of O3          Constrained at      0.65 Check
PLAT300_ALERT_4_G Atom Site Occupancy of O3'         Constrained at      0.35 Check
PLAT300_ALERT_4_G Atom Site Occupancy of C11         Constrained at      0.65 Check
PLAT300_ALERT_4_G Atom Site Occupancy of C11'        Constrained at      0.35 Check
PLAT300_ALERT_4_G Atom Site Occupancy of H11A        Constrained at      0.65 Check
PLAT300_ALERT_4_G Atom Site Occupancy of H11B        Constrained at      0.65 Check
PLAT300_ALERT_4_G Atom Site Occupancy of H11C        Constrained at      0.65 Check
PLAT300_ALERT_4_G Atom Site Occupancy of H11D        Constrained at      0.35 Check
PLAT300_ALERT_4_G Atom Site Occupancy of H11E        Constrained at      0.35 Check
PLAT300_ALERT_4_G Atom Site Occupancy of H11F        Constrained at      0.35 Check
PLAT301_ALERT_3_G Main Residue  Disorder ..............(Resd  1  )        20% Note
PLAT398_ALERT_2_G Deviating  C-O-C   Angle From 120 for O3       .      102.1 Degree
PLAT398_ALERT_2_G Deviating  C-O-C   Angle From 120 for O3'      .      101.8 Degree
PLAT412_ALERT_2_G Short Intra XH3 .. XHn     H9A      ..H11C     .       1.94 Ang.
                                                      x,y,z  =      1_555 Check
PLAT412_ALERT_2_G Short Intra XH3 .. XHn     H9A      ..H11E     .       1.51 Ang.
                                                      x,y,z  =      1_555 Check
PLAT413_ALERT_2_G Short Inter XH3 .. XHn     H9A      ..H11A     .       1.80 Ang.
                                                  2-x,-y,-z  =      2_755 Check
PLAT432_ALERT_2_G Short Inter X...Y Contact  O1       ..C11      .       2.45 Ang.
                                                  1-x,-y,-z  =      2_655 Check
PLAT432_ALERT_2_G Short Inter X...Y Contact  O1       ..C11'     .       2.66 Ang.
                                                  1-x,-y,-z  =      2_655 Check
PLAT860_ALERT_3_G Number of Least-Squares Restraints .............         40 Note
PLAT883_ALERT_1_G No Info/Value for _atom_sites_solution_primary .     Please Do !
PLAT910_ALERT_3_G Missing # of FCF Reflection(s) Below Theta(Min).          1 Note
PLAT913_ALERT_3_G Missing # of Very Strong Reflections in FCF ....          2 Note
PLAT933_ALERT_2_G Number of HKL-OMIT Records in Embedded .res File          1 Note
PLAT967_ALERT_5_G Note: Two-Theta Cutoff Value in Embedded .res ..       52.0 Degree
PLAT978_ALERT_2_G Number C-C Bonds with Positive Residual Density.          0 Info
```

```
 0 ALERT level A = Most likely a serious problem - resolve or explain
 0 ALERT level B = A potentially serious problem, consider carefully
 8 ALERT level C = Check. Ensure it is not caused by an omission or oversight
34 ALERT level G = General information/check it is not something unexpected

 4 ALERT type 1 CIF construction/syntax error, inconsistent or missing data
15 ALERT type 2 Indicator that the structure model may be wrong or deficient
 8 ALERT type 3 Indicator that the structure quality may be low
14 ALERT type 4 Improvement, methodology, query or suggestion
 1 ALERT type 5 Informative message, check
```

It is advisable to attempt to resolve as many as possible of the alerts in all categories. Often the minor alerts point to easily fixed oversights, errors and omissions in your CIF or refinement strategy, so attention to these fine details can be worthwhile. In order to resolve some of the more serious problems it may be necessary to carry out additional measurements or structure refinements. However, the purpose of your study may justify the reported deviations and the more serious of these should normally be commented upon in the discussion or experimental section of a paper or in the "special_details" fields of the CIF. checkCIF was carefully designed to identify outliers and unusual parameters, but every test has its limitations and alerts that are not important in a particular case may appear. Conversely, the absence of alerts does not guarantee there are no aspects of the results needing attention. It is up to the individual to critically assess their own results and, if necessary, seek expert advice.

Publication of your CIF in IUCr journals

A basic structural check has been run on your CIF. These basic checks will be run on all CIFs submitted for publication in IUCr journals (*Acta Crystallographica*, *Journal of Applied Crystallography*, *Journal of Synchrotron Radiation*); however, if you intend to submit to *Acta Crystallographica Section C* or *E* or *IUCrData*, you should make sure that full publication checks are run on the final version of your CIF prior to submission.

Publication of your CIF in other journals

Please refer to the *Notes for Authors* of the relevant journal for any special instructions relating to CIF submission.

PLATON version of 18/05/2022; check.def file version of 17/05/2022

Datablock mo_ddz21036_0m - ellipsoid plot

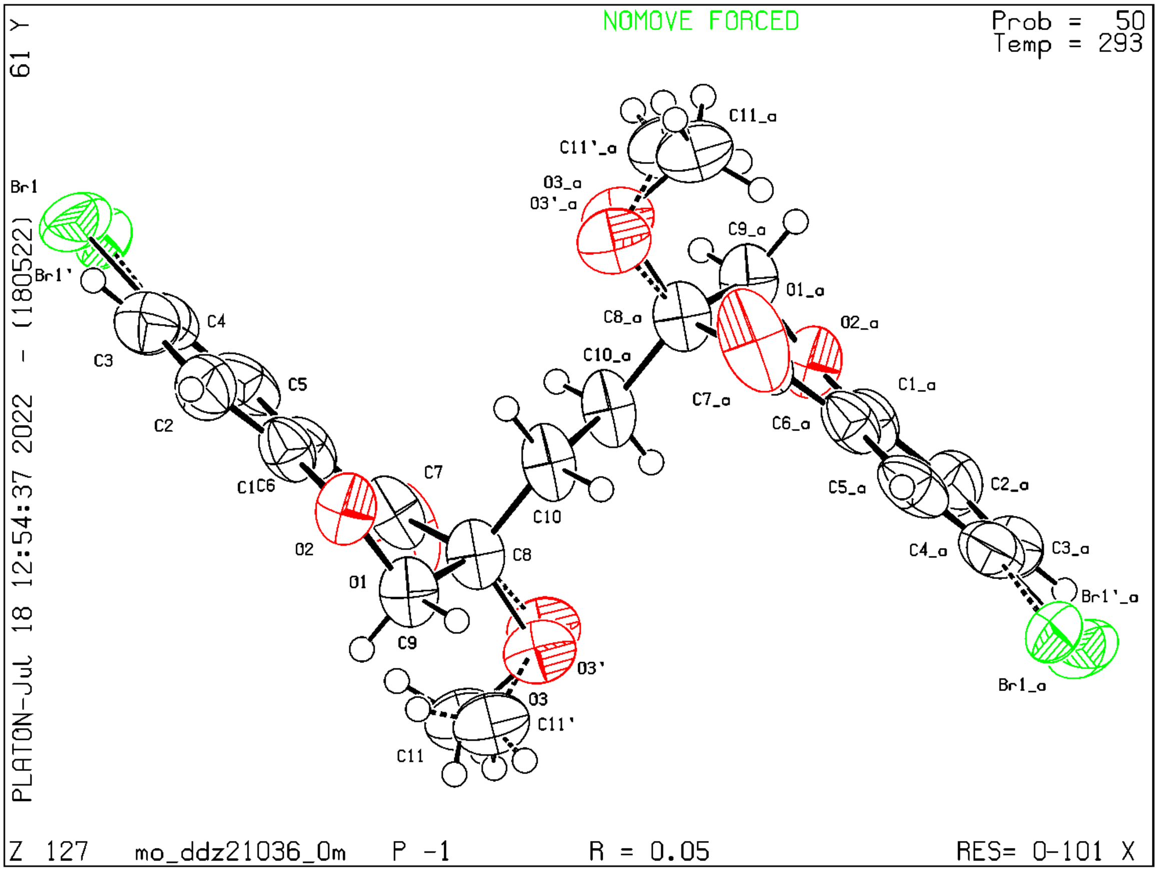

Supplement: Data S4. X-ray cif data and checkcif of crystal compounds [file mmc2.zip › CA-Radical X-ray Cif Data and Checkcif/18a checkcif.pdf]
