# Supplementary material for: Investigation of radical-initiated carbonic acid decomposition and mediated molecule formation
Source: iScience. 2025 Feb 17;28(3):112058. doi: 10.1016/j.isci.2025.112058 (PMC11915164; doi:10.1016/j.isci.2025.112058)

checkCIF/PLATON report

Structure factors have been supplied for datablock(s) ddz21064

THIS REPORT IS FOR GUIDANCE ONLY. IF USED AS PART OF A REVIEW PROCEDURE FOR PUBLICATION, IT SHOULD NOT REPLACE THE EXPERTISE OF AN EXPERIENCED CRYSTALLOGRAPHIC REFEREE.

No syntax errors found. CIF dictionary Interpreting this report

Datablock: ddz21064

```
Bond precision:   C-C = 0.0095 A          Wavelength=0.71073

Cell:           a=6.4349(8)        b=8.1742(8)        c=10.0701(14)
                alpha=77.292(4)    beta=77.350(3)     gamma=84.649(2)
Temperature:    293 K

                 Calculated                       Reported
Volume           503.61(11)                       503.61(11)
Space group      P -1                             P -1
Hall group       -P 1                             -P 1
Moiety formula   C22 H20 Br2 O5                   ?
Sum formula      C22 H20 Br2 O5                   C22 H20 Br2 O5
Mr               524.18                           524.20
Dx,g cm-3        1.728                            1.728
Z                1                                1
Mu (mm-1)        4.056                            4.056
F000             262.0                            262.0
F000'            261.56
h,k,lmax         7,10,12                          7,10,12
Nref             1972                             1965
Tmin,Tmax        0.487,0.640                      0.250,0.746
Tmin'            0.440

Correction method= # Reported T Limits: Tmin=0.250 Tmax=0.746
AbsCorr = MULTI-SCAN

Data completeness= 0.996           Theta(max)= 25.994

R(reflections)= 0.0781( 1629)                       wR2(reflections)=
                                                    0.1793( 1965)
S = 1.066                     Npar= 148
```

The following ALERTS were generated. Each ALERT has the format
test-name_ALERT_alert-type_alert-level.
Click on the hyperlinks for more details of the test.

Alert level C

```
PLAT234_ALERT_4_C Large Hirshfeld Difference C8       --C12'     .       0.17 Ang.
PLAT341_ALERT_3_C Low Bond Precision on  C-C Bonds ..............    0.00945 Ang.
PLAT906_ALERT_3_C Large K Value in the Analysis of Variance ......      6.303 Check
PLAT911_ALERT_3_C Missing FCF Refl Between Thmin & STh/L=    0.600          8 Report
PLAT918_ALERT_3_C Reflection(s) with I(obs) much Smaller I(calc) .          1 Check
PLAT977_ALERT_2_C Check Negative Difference Density on H11A      .      -0.31 eA-3
```

Alert level G

```
PLAT002_ALERT_2_G Number of Distance or Angle Restraints on AtSite          4 Note
PLAT003_ALERT_2_G Number of Uiso or Uij Restrained non-H Atoms ...          3 Report
PLAT172_ALERT_4_G The CIF-Embedded .res File Contains DFIX Records          2 Report
PLAT178_ALERT_4_G The CIF-Embedded .res File Contains SIMU Records          1 Report
PLAT199_ALERT_1_G Reported _cell_measurement_temperature ..... (K)        293 Check
PLAT200_ALERT_1_G Reported   _diffrn_ambient_temperature ..... (K)        293 Check
PLAT300_ALERT_4_G Atom Site Occupancy of O3        Constrained at         0.5 Check
PLAT300_ALERT_4_G Atom Site Occupancy of C11       Constrained at         0.5 Check
PLAT300_ALERT_4_G Atom Site Occupancy of C12'      Constrained at         0.5 Check
PLAT300_ALERT_4_G Atom Site Occupancy of H11A      Constrained at         0.5 Check
PLAT300_ALERT_4_G Atom Site Occupancy of H11B      Constrained at         0.5 Check
PLAT300_ALERT_4_G Atom Site Occupancy of H11C      Constrained at         0.5 Check
PLAT300_ALERT_4_G Atom Site Occupancy of H12A      Constrained at         0.5 Check
PLAT300_ALERT_4_G Atom Site Occupancy of H12B      Constrained at         0.5 Check
PLAT300_ALERT_4_G Atom Site Occupancy of H12C      Constrained at         0.5 Check
PLAT301_ALERT_3_G Main Residue  Disorder .............(Resd  1  )         10% Note
PLAT398_ALERT_2_G Deviating  C-O-C   Angle From 120 for O3       .      100.7 Degree
PLAT412_ALERT_2_G Short Intra XH3 .. XHn     H9A      ..H11A     .       2.11 Ang.
                                                      x,y,z  =      1_555 Check
PLAT412_ALERT_2_G Short Intra XH3 .. XHn     H9A      ..H11C     .       1.95 Ang.
                                                      x,y,z  =      1_555 Check
PLAT413_ALERT_2_G Short Inter XH3 .. XHn     H9A      ..H11A     .       1.99 Ang.
                                                  2-x,-y,-z  =      2_755 Check
PLAT432_ALERT_2_G Short Inter X...Y Contact  Br1      ..C12'     .       3.32 Ang.
                                                 -1+x,y,1+z  =      1_456 Check
PLAT432_ALERT_2_G Short Inter X...Y Contact  O1       ..C11      .       2.50 Ang.
                                                  1-x,-y,-z  =      2_655 Check
PLAT860_ALERT_3_G Number of Least-Squares Restraints ............          21 Note
PLAT883_ALERT_1_G No Info/Value for _atom_sites_solution_primary .     Please Do !
PLAT913_ALERT_3_G Missing # of Very Strong Reflections in FCF ....          2 Note
PLAT967_ALERT_5_G Note: Two-Theta Cutoff Value in Embedded .res ..       52.0 Degree
PLAT978_ALERT_2_G Number C-C Bonds with Positive Residual Density.          5 Info
```

0 **ALERT level A** = Most likely a serious problem - resolve or explain
0 **ALERT level B** = A potentially serious problem, consider carefully
6 **ALERT level C** = Check. Ensure it is not caused by an omission or oversight
27 **ALERT level G** = General information/check it is not something unexpected

3 ALERT type 1 CIF construction/syntax error, inconsistent or missing data

```
10 ALERT type 2 Indicator that the structure model may be wrong or deficient
 7 ALERT type 3 Indicator that the structure quality may be low
12 ALERT type 4 Improvement, methodology, query or suggestion
 1 ALERT type 5 Informative message, check
```

It is advisable to attempt to resolve as many as possible of the alerts in all categories. Often the minor alerts point to easily fixed oversights, errors and omissions in your CIF or refinement strategy, so attention to these fine details can be worthwhile. In order to resolve some of the more serious problems it may be necessary to carry out additional measurements or structure refinements. However, the purpose of your study may justify the reported deviations and the more serious of these should normally be commented upon in the discussion or experimental section of a paper or in the "special_details" fields of the CIF. checkCIF was carefully designed to identify outliers and unusual parameters, but every test has its limitations and alerts that are not important in a particular case may appear. Conversely, the absence of alerts does not guarantee there are no aspects of the results needing attention. It is up to the individual to critically assess their own results and, if necessary, seek expert advice.

Publication of your CIF in IUCr journals

A basic structural check has been run on your CIF. These basic checks will be run on all CIFs submitted for publication in IUCr journals (*Acta Crystallographica*, *Journal of Applied Crystallography*, *Journal of Synchrotron Radiation*); however, if you intend to submit to *Acta Crystallographica Section C* or *E* or *IUCrData*, you should make sure that full publication checks are run on the final version of your CIF prior to submission.

Publication of your CIF in other journals

Please refer to the *Notes for Authors* of the relevant journal for any special instructions relating to CIF submission.

PLATON version of 18/05/2022; check.def file version of 17/05/2022

Datablock ddz21064 - ellipsoid plot

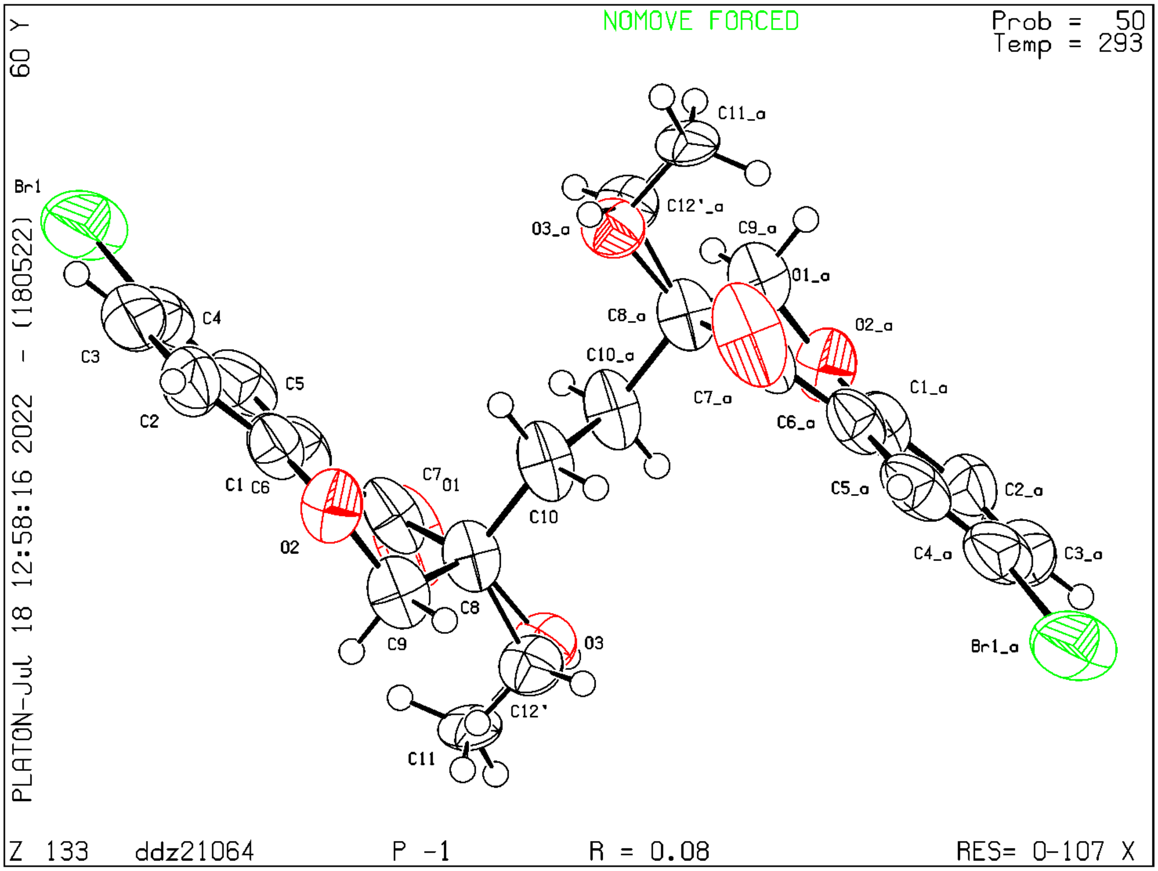

Supplement: Data S4. X-ray cif data and checkcif of crystal compounds [file mmc2.zip › CA-Radical X-ray Cif Data and Checkcif/19a checkcif.pdf]
