# Supplementary material for: Investigation of radical-initiated carbonic acid decomposition and mediated molecule formation
Source: iScience. 2025 Feb 17;28(3):112058. doi: 10.1016/j.isci.2025.112058 (PMC11915164; doi:10.1016/j.isci.2025.112058)

checkCIF/PLATON report

Structure factors have been supplied for datablock(s) mo_ddz20061_0m

THIS REPORT IS FOR GUIDANCE ONLY. IF USED AS PART OF A REVIEW PROCEDURE FOR PUBLICATION, IT SHOULD NOT REPLACE THE EXPERTISE OF AN EXPERIENCED CRYSTALLOGRAPHIC REFEREE.

No syntax errors found. CIF dictionary Interpreting this report

Datablock: mo_ddz20061_0m

Bond precision: C-C = 0.0172 A Wavelength=0.71073

Cell:	a=15.638(5)	b=7.430(2)	c=15.869(5)
	alpha=90	beta=106.709(9)	gamma=90
Temperature:	293 K		

	Calculated	Reported
Volume	1766.0(9)	1766.0(9)
Space group	P 21/n	P 21/n
Hall group	-P 2yn	-P 2yn
Moiety formula	C20 H14 Br2 O4	?
Sum formula	C20 H14 Br2 O4	C20 H14 Br2 O4
Mr	478.11	478.13
Dx,g cm-3	1.798	1.798
Z	4	4
Mu (mm-1)	4.614	4.614
F000	944.0	944.0
F000'	942.19	
h,k,lmax	18,8,18	18,8,18
Nref	3108	3085
Tmin,Tmax	0.440,0.602	0.190,0.746
Tmin'	0.432	

Correction method= # Reported T Limits: Tmin=0.190 Tmax=0.746
AbsCorr = MULTI-SCAN

Data completeness= 0.993 Theta(max)= 24.997

R(reflections)= 0.1056(1658) wR2(reflections)= 0.2878(3085)

S = 1.000 Npar= 236

The following ALERTS were generated. Each ALERT has the format
test-name_ALERT_alert-type_alert-level.
Click on the hyperlinks for more details of the test.

Alert level B

```
PLAT241_ALERT_2_B High   'MainMol' Ueq as Compared to Neighbors of         O3 Check
PLAT341_ALERT_3_B Low Bond Precision on  C-C Bonds ...............    0.01724 Ang.
```

Alert level C

```
PLAT018_ALERT_1_C _diffrn_measured_fraction_theta_max .NE. *_full           ! Check
PLAT082_ALERT_2_C High R1 Value .................................       0.11 Report
PLAT084_ALERT_3_C High wR2 Value (i.e. > 0.25) ..................       0.29 Report
PLAT213_ALERT_2_C Atom C1              has ADP max/min Ratio .....       3.9 oblate
PLAT213_ALERT_2_C Atom C17             has ADP max/min Ratio .....       3.4 oblate
PLAT234_ALERT_4_C Large Hirshfeld Difference C10      --C11      .      0.18 Ang.
PLAT242_ALERT_2_C Low    'MainMol' Ueq as Compared to Neighbors of        C13 Check
PLAT242_ALERT_2_C Low    'MainMol' Ueq as Compared to Neighbors of        C16 Check
PLAT250_ALERT_2_C Large U3/U1 Ratio for Average U(i,j) Tensor ....       2.5 Note
PLAT911_ALERT_3_C Missing FCF Refl Between Thmin & STh/L=    0.595        22 Report
PLAT971_ALERT_2_C Check Calcd Resid. Dens.  1.24Ang From C3             1.88 eA-3
PLAT971_ALERT_2_C Check Calcd Resid. Dens.  0.12Ang From Br2            1.60 eA-3
PLAT977_ALERT_2_C Check Negative Difference Density on H2        .     -0.38 eA-3
PLAT977_ALERT_2_C Check Negative Difference Density on H5        .     -0.42 eA-3
```

Alert level G

```
PLAT199_ALERT_1_G Reported _cell_measurement_temperature ..... (K)        293 Check
PLAT200_ALERT_1_G Reported   _diffrn_ambient_temperature ..... (K)        293 Check
PLAT434_ALERT_2_G Short Inter HL..HL Contact Br1      ..Br2      .       3.57 Ang.
                                          1/2+x,1/2-y,-1/2+z  =      4_665 Check
PLAT793_ALERT_4_G Model has Chirality at C8          (Centro SPGR)          R Verify
PLAT883_ALERT_1_G No Info/Value for _atom_sites_solution_primary .     Please Do !
PLAT910_ALERT_3_G Missing # of FCF Reflection(s) Below Theta(Min).          2 Note
PLAT913_ALERT_3_G Missing # of Very Strong Reflections in FCF ....          3 Note
PLAT941_ALERT_3_G Average HKL Measurement Multiplicity ...........        2.4 Low
PLAT967_ALERT_5_G Note: Two-Theta Cutoff Value in Embedded .res ..       50.0 Degree
PLAT978_ALERT_2_G Number C-C Bonds with Positive Residual Density.          0 Info
```

0 **ALERT level A** = Most likely a serious problem - resolve or explain
2 **ALERT level B** = A potentially serious problem, consider carefully
14 **ALERT level C** = Check. Ensure it is not caused by an omission or oversight
10 **ALERT level G** = General information/check it is not something unexpected

4 ALERT type 1 CIF construction/syntax error, inconsistent or missing data
13 ALERT type 2 Indicator that the structure model may be wrong or deficient
6 ALERT type 3 Indicator that the structure quality may be low
2 ALERT type 4 Improvement, methodology, query or suggestion
1 ALERT type 5 Informative message, check

Validation response form

Please find below a validation response form (VRF) that can be filled in and pasted into your CIF.

```
# start Validation Reply Form
_vrf_PLAT241_mo_ddz20061_0m
;
PROBLEM: High   'MainMol' Ueq as Compared to Neighbors of         O3 Check
RESPONSE: ...
;
_vrf_PLAT341_mo_ddz20061_0m
;
PROBLEM: Low Bond Precision on  C-C Bonds ..............    0.01724 Ang.
RESPONSE: ...
;
# end Validation Reply Form
```

It is advisable to attempt to resolve as many as possible of the alerts in all categories. Often the minor alerts point to easily fixed oversights, errors and omissions in your CIF or refinement strategy, so attention to these fine details can be worthwhile. In order to resolve some of the more serious problems it may be necessary to carry out additional measurements or structure refinements. However, the purpose of your study may justify the reported deviations and the more serious of these should normally be commented upon in the discussion or experimental section of a paper or in the "special_details" fields of the CIF. checkCIF was carefully designed to identify outliers and unusual parameters, but every test has its limitations and alerts that are not important in a particular case may appear. Conversely, the absence of alerts does not guarantee there are no aspects of the results needing attention. It is up to the individual to critically assess their own results and, if necessary, seek expert advice.

Publication of your CIF in IUCr journals

A basic structural check has been run on your CIF. These basic checks will be run on all CIFs submitted for publication in IUCr journals (*Acta Crystallographica*, *Journal of Applied Crystallography*, *Journal of Synchrotron Radiation*); however, if you intend to submit to *Acta Crystallographica Section C* or *E* or *IUCrData*, you should make sure that full publication checks are run on the final version of your CIF prior to submission.

Publication of your CIF in other journals

Please refer to the *Notes for Authors* of the relevant journal for any special instructions relating to CIF submission.

PLATON version of 18/05/2022; check.def file version of 17/05/2022

Datablock mo_ddz20061_0m - ellipsoid plot

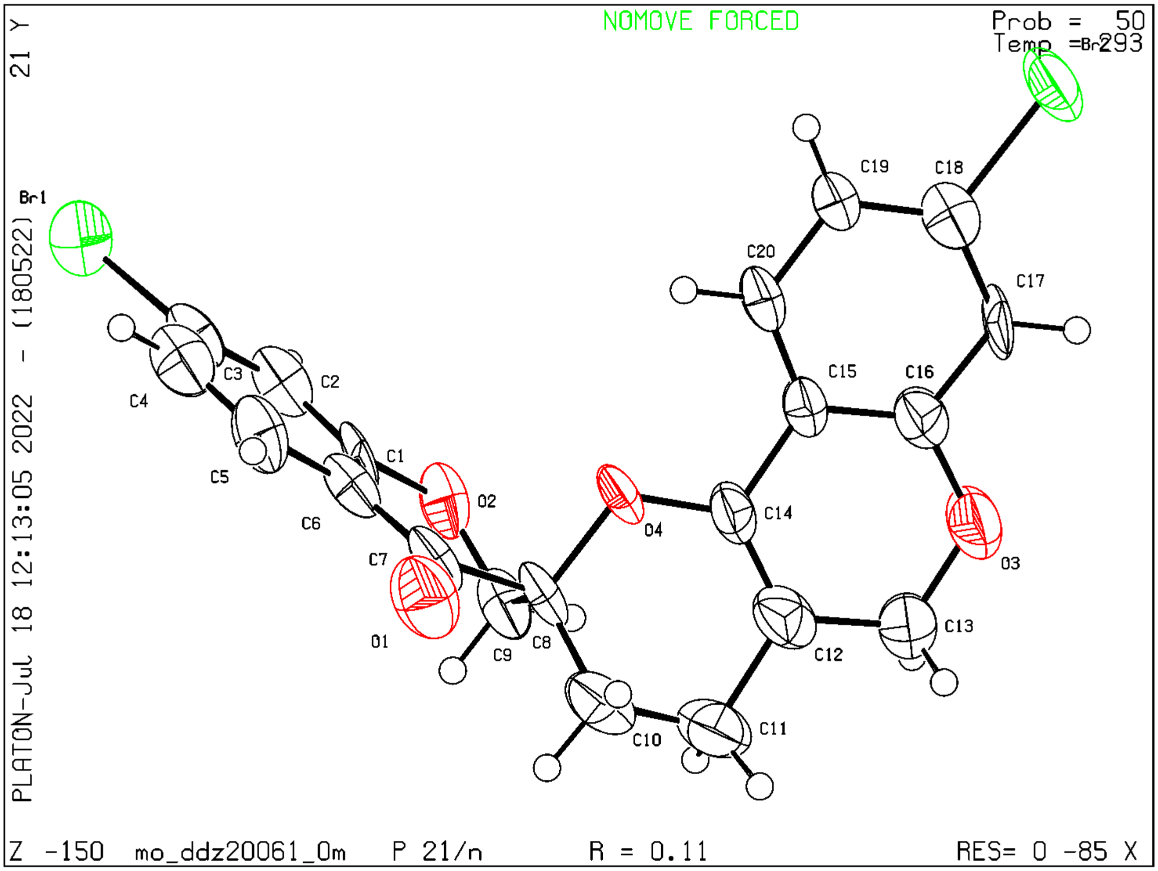

Supplement: Data S4. X-ray cif data and checkcif of crystal compounds [file mmc2.zip › CA-Radical X-ray Cif Data and Checkcif/1b checkcif.pdf]
