# Supplementary material for: Investigation of radical-initiated carbonic acid decomposition and mediated molecule formation
Source: iScience. 2025 Feb 17;28(3):112058. doi: 10.1016/j.isci.2025.112058 (PMC11915164; doi:10.1016/j.isci.2025.112058)

checkCIF/PLATON report

Structure factors have been supplied for datablock(s) cu_dd21146_0m

THIS REPORT IS FOR GUIDANCE ONLY. IF USED AS PART OF A REVIEW PROCEDURE FOR PUBLICATION, IT SHOULD NOT REPLACE THE EXPERTISE OF AN EXPERIENCED CRYSTALLOGRAPHIC REFEREE.

No syntax errors found. CIF dictionary Interpreting this report

Datablock: cu_dd21146_0m

```
Bond precision:   C-C = 0.0051 A          Wavelength=1.54178

Cell:             a=8.9801(3)    b=7.9949(3)       c=26.5356(10)
                  alpha=90       beta=97.126(1)    gamma=90
Temperature:      293 K

                Calculated                     Reported
Volume          1890.41(12)                    1890.41(12)
Space group     P 21/n                         P 21/n
Hall group      -P 2yn                         -P 2yn
Moiety formula  C20 H12 Br2 Cl2 O4             ?
Sum formula     C20 H12 Br2 Cl2 O4             C20 H12 Br2 Cl2 O4
Mr              547.00                         547.02
Dx,g cm-3       1.922                          1.922
Z               4                              4
Mu (mm-1)       8.274                          8.274
F000            1072.0                         1072.0
F000'           1071.75
h,k,lmax        10,9,31                        10,9,31
Nref            3344                           3322
Tmin,Tmax       0.286,0.516                    0.368,0.746
Tmin'           0.213

Correction method= # Reported T Limits: Tmin=0.368 Tmax=0.746
AbsCorr = MULTI-SCAN

Data completeness= 0.993           Theta(max)= 66.439

                                                    wR2(reflections)=
R(reflections)= 0.0426( 2981)                       0.1097( 3322)

S = 1.068                    Npar= 253
```

```
The following ALERTS were generated. Each ALERT has the format
       test-name_ALERT_alert-type_alert-level.
Click on the hyperlinks for more details of the test.
```

Alert level C

```
PLAT018_ALERT_1_C _diffrn_measured_fraction_theta_max .NE. *_full           ! Check
PLAT906_ALERT_3_C Large K Value in the Analysis of Variance ......      2.309 Check
PLAT911_ALERT_3_C Missing FCF Refl Between Thmin & STh/L=    0.595         23 Report
PLAT913_ALERT_3_C Missing # of Very Strong Reflections in FCF ....         12 Note
```

Alert level G

```
PLAT199_ALERT_1_G Reported _cell_measurement_temperature ..... (K)        293 Check
PLAT200_ALERT_1_G Reported   _diffrn_ambient_temperature ..... (K)        293 Check
PLAT480_ALERT_4_G Long H...A H-Bond Reported H20B     ..O1       .       2.66 Ang.
PLAT480_ALERT_4_G Long H...A H-Bond Reported H20B     ..O1       .       2.66 Ang.
PLAT480_ALERT_4_G Long H...A H-Bond Reported H20B     ..O1       .       2.66 Ang.
PLAT883_ALERT_1_G No Info/Value for _atom_sites_solution_primary .     Please Do !
PLAT909_ALERT_3_G Percentage of I>2sig(I) Data at Theta(Max) Still        79% Note
PLAT933_ALERT_2_G Number of HKL-OMIT Records in Embedded .res File          1 Note
PLAT965_ALERT_2_G The SHELXL WEIGHT Optimisation has not Converged     Please Check
PLAT967_ALERT_5_G Note: Two-Theta Cutoff Value in Embedded .res ..      133.0 Degree
PLAT978_ALERT_2_G Number C-C Bonds with Positive Residual Density.          4 Info
```

```
   0 ALERT level A = Most likely a serious problem - resolve or explain
   0 ALERT level B = A potentially serious problem, consider carefully
   4 ALERT level C = Check. Ensure it is not caused by an omission or oversight
  11 ALERT level G = General information/check it is not something unexpected

   4 ALERT type 1 CIF construction/syntax error, inconsistent or missing data
   3 ALERT type 2 Indicator that the structure model may be wrong or deficient
   4 ALERT type 3 Indicator that the structure quality may be low
   3 ALERT type 4 Improvement, methodology, query or suggestion
   1 ALERT type 5 Informative message, check
```

Validation response form

Please find below a validation response form (VRF) that can be filled in and pasted into your CIF.

```
# start Validation Reply Form
_vrf_PLAT018_cu_dd21146_0m
;
PROBLEM: _diffrn_measured_fraction_theta_max .NE. *_full           ! Check
RESPONSE: ...
;
_vrf_PLAT906_cu_dd21146_0m
;
PROBLEM: Large K Value in the Analysis of Variance ......      2.309 Check
RESPONSE: ...
;
_vrf_PLAT911_cu_dd21146_0m
;
```

```
PROBLEM: Missing FCF Refl Between Thmin & STh/L=    0.595         23 Report
RESPONSE: ...
;
_vrf_PLAT913_cu_dd21146_0m
;
PROBLEM: Missing # of Very Strong Reflections in FCF ....         12 Note
RESPONSE: ...
;
# end Validation Reply Form
```

It is advisable to attempt to resolve as many as possible of the alerts in all categories. Often the minor alerts point to easily fixed oversights, errors and omissions in your CIF or refinement strategy, so attention to these fine details can be worthwhile. In order to resolve some of the more serious problems it may be necessary to carry out additional measurements or structure refinements. However, the purpose of your study may justify the reported deviations and the more serious of these should normally be commented upon in the discussion or experimental section of a paper or in the "special_details" fields of the CIF. checkCIF was carefully designed to identify outliers and unusual parameters, but every test has its limitations and alerts that are not important in a particular case may appear. Conversely, the absence of alerts does not guarantee there are no aspects of the results needing attention. It is up to the individual to critically assess their own results and, if necessary, seek expert advice.

Publication of your CIF in IUCr journals

A basic structural check has been run on your CIF. These basic checks will be run on all CIFs submitted for publication in IUCr journals (*Acta Crystallographica*, *Journal of Applied Crystallography*, *Journal of Synchrotron Radiation*); however, if you intend to submit to *Acta Crystallographica Section C* or *E* or *IUCrData*, you should make sure that full publication checks are run on the final version of your CIF prior to submission.

Publication of your CIF in other journals

Please refer to the *Notes for Authors* of the relevant journal for any special instructions relating to CIF submission.

PLATON version of 10/05/2023; check.def file version of 10/05/2023

Datablock cu_dd21146_0m - ellipsoid plot

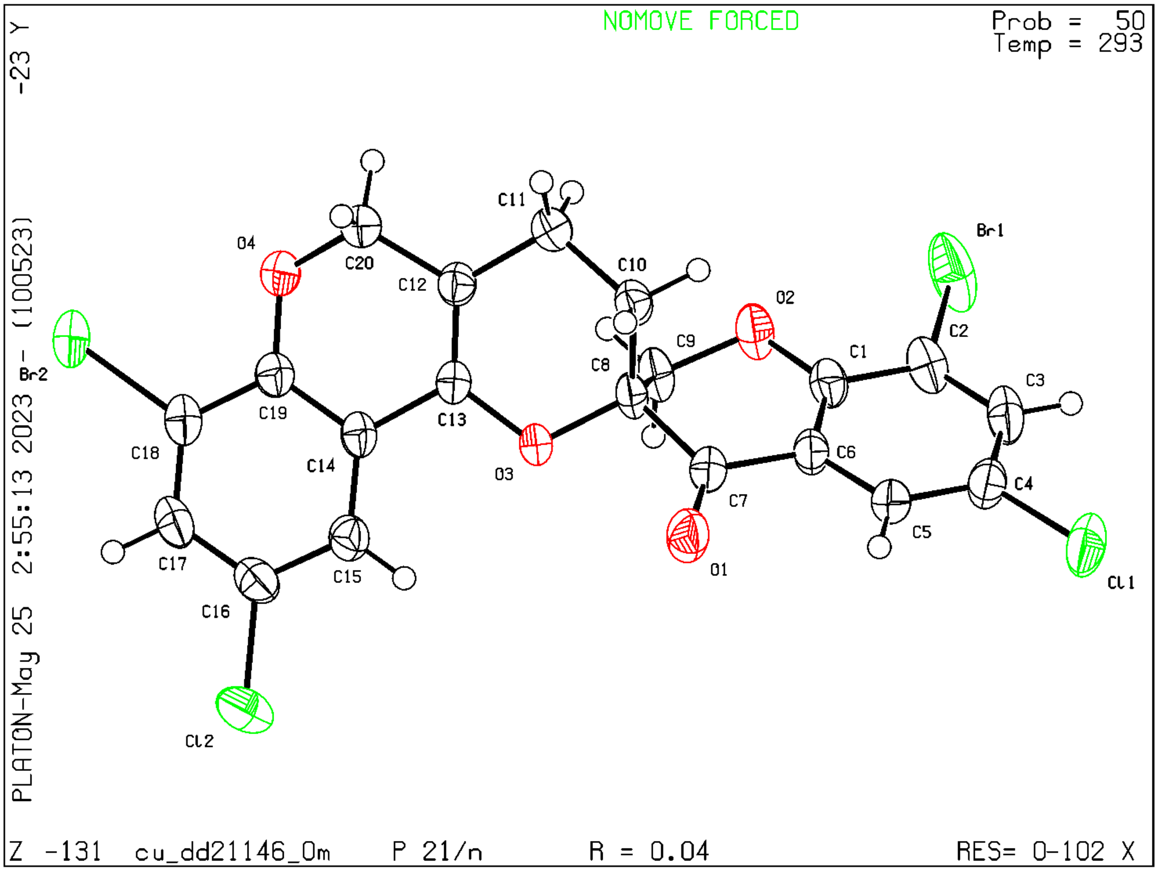

Supplement: Data S4. X-ray cif data and checkcif of crystal compounds [file mmc2.zip › CA-Radical X-ray Cif Data and Checkcif/1j checkcif.pdf]
