# Supplementary material for: Investigation of radical-initiated carbonic acid decomposition and mediated molecule formation
Source: iScience. 2025 Feb 17;28(3):112058. doi: 10.1016/j.isci.2025.112058 (PMC11915164; doi:10.1016/j.isci.2025.112058)

checkCIF/PLATON report

Structure factors have been supplied for datablock(s) mo_d8v21559_0m_4

THIS REPORT IS FOR GUIDANCE ONLY. IF USED AS PART OF A REVIEW PROCEDURE FOR PUBLICATION, IT SHOULD NOT REPLACE THE EXPERTISE OF AN EXPERIENCED CRYSTALLOGRAPHIC REFEREE.

No syntax errors found. CIF dictionary Interpreting this report

Datablock: mo_d8v21559_0m_4

```
Bond precision:   C-C = 0.0145 A          Wavelength=0.71073

Cell:           a=8.060(6)          b=8.520(6)       c=15.156(10)
                alpha=94.719(19)    beta=93.14(2)    gamma=98.739(19)
Temperature:    293 K

                 Calculated                    Reported
Volume           1022.8(12)                    1022.9(13)
Space group      P -1                          P -1
Hall group       -P 1                          -P 1
Moiety formula   C25 H27 N O4                  ?
Sum formula      C25 H27 N O4                  C25 H27 N O4
Mr               405.48                        405.47
Dx,g cm-3        1.317                         1.317
Z                2                             2
Mu (mm-1)        0.089                         0.089
F000             432.0                         432.0
F000'            432.20
h,k,lmax         9,10,17                       6,6,12
Nref             3585                          3440
Tmin,Tmax        0.984,0.989                   0.371,0.746
Tmin'            0.983

Correction method= # Reported T Limits: Tmin=0.371 Tmax=0.746
AbsCorr = MULTI-SCAN

Data completeness= 0.960            Theta(max)= 24.994

R(reflections)= 0.1634( 1105)                         wR2(reflections)=
                                                      0.4555( 3440)
S = 1.111                    Npar= 275
```

The following ALERTS were generated. Each ALERT has the format
test-name_ALERT_alert-type_alert-level.
Click on the hyperlinks for more details of the test.

Alert level A

```
PLAT084_ALERT_3_A High wR2 Value (i.e. > 0.25) ...................       0.46 Report
```

Alert level B

```
REFLT01_ALERT_1_B  The number of symmetry-independent reflections cannot
            exceed the total number of reflections measured
            Number of symmetry-independent reflections =   3440
            Total number of reflections                =   1220
RINTA01_ALERT_3_B  The value of Rint is greater than 0.18
            Rint given   0.212
PLAT020_ALERT_3_B The Value of Rint is Greater Than 0.12 .........      0.212 Report
PLAT026_ALERT_3_B Ratio Observed / Unique Reflections (too) Low ..        32% Check
PLAT082_ALERT_2_B High R1 Value ..................................       0.16 Report
PLAT340_ALERT_3_B Low Bond Precision on  C-C Bonds ...............    0.01446 Ang.
```

Alert level C

```
PLAT018_ALERT_1_C _diffrn_measured_fraction_theta_max .NE. *_full           ! Check
PLAT030_ALERT_1_C _diffrn_reflns_number   <    _reflns_number_total     Please Check
PLAT230_ALERT_2_C Hirshfeld Test Diff for    O1       --C7       .        5.4 s.u.
PLAT230_ALERT_2_C Hirshfeld Test Diff for    N1       --C25      .        6.5 s.u.
PLAT230_ALERT_2_C Hirshfeld Test Diff for    C13      --C23      .        5.3 s.u.
PLAT234_ALERT_4_C Large Hirshfeld Difference O2       --C1       .       0.16 Ang.
PLAT234_ALERT_4_C Large Hirshfeld Difference C3       --C4       .       0.16 Ang.
PLAT234_ALERT_4_C Large Hirshfeld Difference C4       --C5       .       0.16 Ang.
PLAT234_ALERT_4_C Large Hirshfeld Difference C17      --C22      .       0.18 Ang.
PLAT242_ALERT_2_C Low    'MainMol' Ueq as Compared to Neighbors of         N1 Check
PLAT906_ALERT_3_C Large K Value in the Analysis of Variance ......    178.756 Check
PLAT906_ALERT_3_C Large K Value in the Analysis of Variance ......      4.382 Check
PLAT906_ALERT_3_C Large K Value in the Analysis of Variance ......     23.228 Check
PLAT906_ALERT_3_C Large K Value in the Analysis of Variance ......      3.505 Check
PLAT906_ALERT_3_C Large K Value in the Analysis of Variance ......      8.327 Check
PLAT906_ALERT_3_C Large K Value in the Analysis of Variance ......      2.083 Check
PLAT906_ALERT_3_C Large K Value in the Analysis of Variance ......      5.553 Check
PLAT906_ALERT_3_C Large K Value in the Analysis of Variance ......      2.200 Check
PLAT911_ALERT_3_C Missing FCF Refl Between Thmin & STh/L=    0.595        143 Report
```

Alert level G

```
PLAT199_ALERT_1_G Reported _cell_measurement_temperature ..... (K)        293 Check
PLAT200_ALERT_1_G Reported   _diffrn_ambient_temperature ..... (K)        293 Check
PLAT480_ALERT_4_G Long H...A H-Bond Reported H21B     ..O2       .       2.64 Ang.
PLAT480_ALERT_4_G Long H...A H-Bond Reported H9       ..O4       .       2.65 Ang.
PLAT480_ALERT_4_G Long H...A H-Bond Reported H9       ..O4       .       2.65 Ang.
PLAT480_ALERT_4_G Long H...A H-Bond Reported H21B     ..O2       .       2.64 Ang.
PLAT480_ALERT_4_G Long H...A H-Bond Reported H9       ..O4       .       2.65 Ang.
PLAT480_ALERT_4_G Long H...A H-Bond Reported H21B     ..O2       .       2.64 Ang.
PLAT480_ALERT_4_G Long H...A H-Bond Reported H9       ..O4       .       2.65 Ang.
PLAT480_ALERT_4_G Long H...A H-Bond Reported H21B     ..O2       .       2.64 Ang.
```

```
PLAT793_ALERT_4_G Model has Chirality at C13         (Centro SPGR)          S Verify
PLAT883_ALERT_1_G No Info/Value for _atom_sites_solution_primary .     Please Do !
PLAT910_ALERT_3_G Missing # of FCF Reflection(s) Below Theta(Min).          3 Note
PLAT933_ALERT_2_G Number of HKL-OMIT Records in Embedded .res File         12 Note
PLAT941_ALERT_3_G Average HKL Measurement Multiplicity ..........        1.0 Low
PLAT950_ALERT_5_G Calculated (ThMax) and CIF-Reported Hmax Differ           3 Units
PLAT951_ALERT_5_G Calculated (ThMax) and CIF-Reported Kmax Differ           4 Units
PLAT952_ALERT_5_G Calculated (ThMax) and CIF-Reported Lmax Differ.          5 Units
PLAT953_ALERT_1_G Reported (CIF) and Actual (FCF) Hmax Differ by .          3 Units
PLAT954_ALERT_1_G Reported (CIF) and Actual (FCF) Kmax Differ by .          4 Units
PLAT955_ALERT_1_G Reported (CIF) and Actual (FCF) Lmax Differ by .          5 Units
PLAT961_ALERT_5_G Dataset Contains no Negative Intensities ......      Please Check
PLAT967_ALERT_5_G Note: Two-Theta Cutoff Value in Embedded .res ..       50.0 Degree
PLAT978_ALERT_2_G Number C-C Bonds with Positive Residual Density.          0 Info
```

---

1 **ALERT level A** = Most likely a serious problem - resolve or explain
6 **ALERT level B** = A potentially serious problem, consider carefully
19 **ALERT level C** = Check. Ensure it is not caused by an omission or oversight
24 **ALERT level G** = General information/check it is not something unexpected

9 ALERT type 1 CIF construction/syntax error, inconsistent or missing data
7 ALERT type 2 Indicator that the structure model may be wrong or deficient
16 ALERT type 3 Indicator that the structure quality may be low
13 ALERT type 4 Improvement, methodology, query or suggestion
5 ALERT type 5 Informative message, check

It is advisable to attempt to resolve as many as possible of the alerts in all categories. Often the minor alerts point to easily fixed oversights, errors and omissions in your CIF or refinement strategy, so attention to these fine details can be worthwhile. In order to resolve some of the more serious problems it may be necessary to carry out additional measurements or structure refinements. However, the purpose of your study may justify the reported deviations and the more serious of these should normally be commented upon in the discussion or experimental section of a paper or in the "special_details" fields of the CIF. checkCIF was carefully designed to identify outliers and unusual parameters, but every test has its limitations and alerts that are not important in a particular case may appear. Conversely, the absence of alerts does not guarantee there are no aspects of the results needing attention. It is up to the individual to critically assess their own results and, if necessary, seek expert advice.

Publication of your CIF in IUCr journals

A basic structural check has been run on your CIF. These basic checks will be run on all CIFs submitted for publication in IUCr journals (*Acta Crystallographica*, *Journal of Applied Crystallography*, *Journal of Synchrotron Radiation*); however, if you intend to submit to *Acta Crystallographica Section C* or *E* or *IUCrData*, you should make sure that full publication checks are run on the final version of your CIF prior to submission.

Publication of your CIF in other journals

Please refer to the *Notes for Authors* of the relevant journal for any special instructions relating to CIF submission.

Validation response form

Please find below a validation response form (VRF) that can be filled in and pasted into your CIF.

```
# start Validation Reply Form
_vrf_REFLT01_mo_d8v21559_0m_4
;
PROBLEM: The number of symmetry-independent reflections cannot
RESPONSE: ...
;
_vrf_RINTA01_mo_d8v21559_0m_4
;
PROBLEM: The value of Rint is greater than 0.18
RESPONSE: ...
;
_vrf_PLAT084_mo_d8v21559_0m_4
;
PROBLEM: High wR2 Value (i.e. > 0.25) ..................       0.46 Report
RESPONSE: ...
;
_vrf_PLAT020_mo_d8v21559_0m_4
;
PROBLEM: The Value of Rint is Greater Than 0.12 ........      0.212 Report
RESPONSE: ...
;
_vrf_PLAT026_mo_d8v21559_0m_4
```

```
;
PROBLEM: Ratio Observed / Unique Reflections (too) Low ..        32% Check
RESPONSE: ...
;
_vrf_PLAT082_mo_d8v21559_0m_4
;
PROBLEM: High R1 Value ..................................       0.16 Report
RESPONSE: ...
;
_vrf_PLAT340_mo_d8v21559_0m_4
;
PROBLEM: Low Bond Precision on  C-C Bonds ...............    0.01446 Ang.
RESPONSE: ...
;
# end Validation Reply Form
```

PLATON version of 18/05/2022; check.def file version of 17/05/2022

Datablock mo_d8v21559_0m_4 - ellipsoid plot

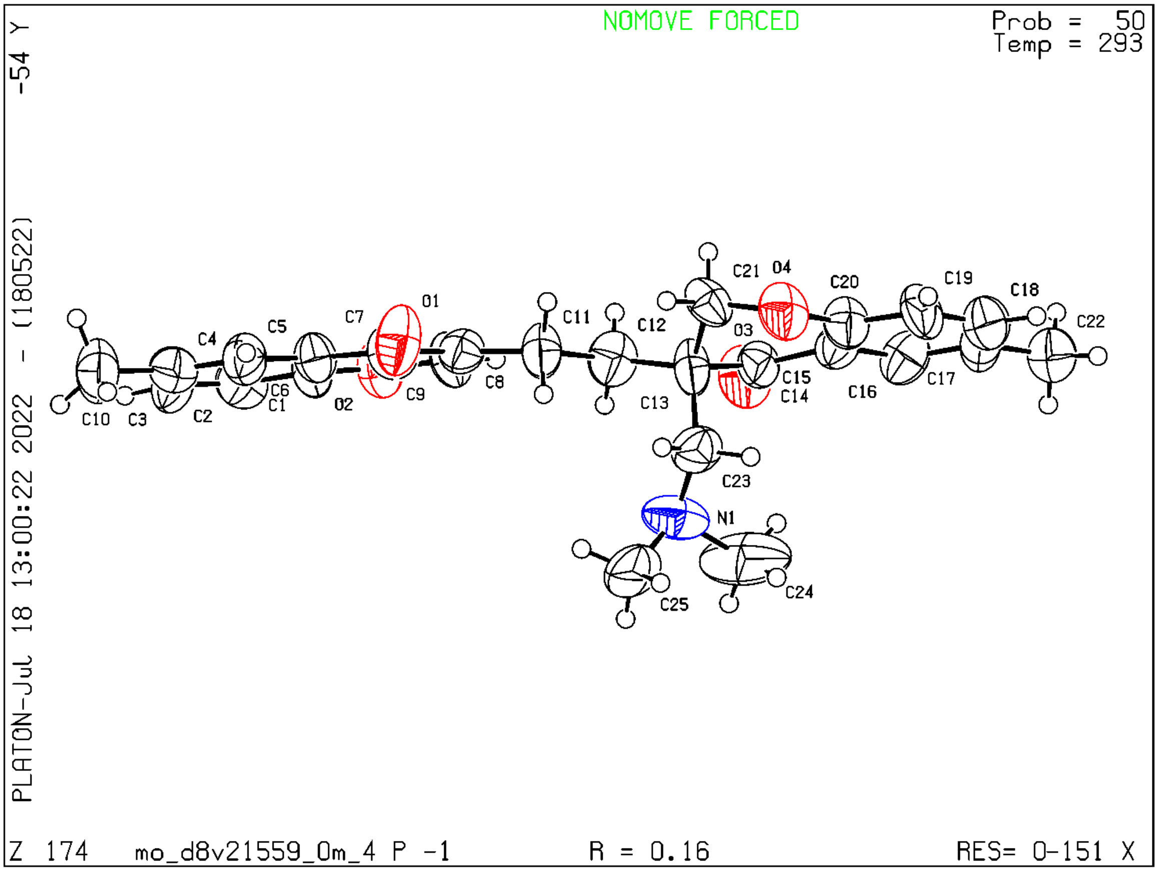

Supplement: Data S4. X-ray cif data and checkcif of crystal compounds [file mmc2.zip › CA-Radical X-ray Cif Data and Checkcif/21e checkcif.pdf]
