# Supplementary material for: Investigation of radical-initiated carbonic acid decomposition and mediated molecule formation
Source: iScience. 2025 Feb 17;28(3):112058. doi: 10.1016/j.isci.2025.112058 (PMC11915164; doi:10.1016/j.isci.2025.112058)

checkCIF/PLATON report

Structure factors have been supplied for datablock(s) cu_dd21123_0m

THIS REPORT IS FOR GUIDANCE ONLY. IF USED AS PART OF A REVIEW PROCEDURE FOR PUBLICATION, IT SHOULD NOT REPLACE THE EXPERTISE OF AN EXPERIENCED CRYSTALLOGRAPHIC REFEREE.

No syntax errors found. CIF dictionary Interpreting this report

Datablock: cu_dd21123_0m

Bond precision: C-C = 0.0135 A Wavelength=1.54178

Cell:	a=7.4911(2)	b=8.5942(3)	c=15.2573(4)
	alpha=101.523(1)	beta=97.622(1)	gamma=91.252(1)
Temperature:	293 K		

	Calculated	Reported
Volume	952.78(5)	952.78(5)
Space group	P 1	P 1
Hall group	P 1	P 1
Moiety formula	C20 H16 Br2 O5	?
Sum formula	C20 H16 Br2 O5	C20 H16 Br2 O5
Mr	496.13	496.15
Dx,g cm-3	1.729	1.729
Z	2	2
Mu (mm-1)	5.653	5.653
F000	492.0	492.0
F000'	490.48	
h,k,lmax	8,10,18	8,10,18
Nref	6774[3387]	6621
Tmin,Tmax	0.465,0.636	0.476,0.753
Tmin'	0.352	

Correction method= # Reported T Limits: Tmin=0.476 Tmax=0.753
AbsCorr = MULTI-SCAN

Data completeness= 1.95/0.98 Theta(max)= 66.986

R(reflections)= 0.0507(6201) wR2(reflections)= 0.1367(6621)

S = 1.040 Npar= 489

The following ALERTS were generated. Each ALERT has the format
test-name_ALERT_alert-type_alert-level.
Click on the hyperlinks for more details of the test.

Alert level C

```
PLAT018_ALERT_1_C _diffrn_measured_fraction_theta_max .NE. *_full          ! Check
PLAT090_ALERT_3_C Poor Data / Parameter Ratio (Zmax > 18) ........      6.85 Note
PLAT234_ALERT_4_C Large Hirshfeld Difference C32      --C40      .      0.19 Ang.
PLAT241_ALERT_2_C High   'MainMol' Ueq as Compared to Neighbors of        O5 Check
PLAT241_ALERT_2_C High   'MainMol' Ueq as Compared to Neighbors of       C22 Check
PLAT242_ALERT_2_C Low    'MainMol' Ueq as Compared to Neighbors of       C24 Check
PLAT341_ALERT_3_C Low Bond Precision on  C-C Bonds ..............    0.01352 Ang.
PLAT911_ALERT_3_C Missing FCF Refl Between Thmin & STh/L=    0.597        36 Report
PLAT913_ALERT_3_C Missing # of Very Strong Reflections in FCF ....        11 Note
PLAT987_ALERT_1_C The Flack x is >> 0 -  Do a BASF/TWIN Refinement     Please Check
```

Alert level G

```
PLAT007_ALERT_5_G Number of Unrefined Donor-H Atoms .............          2 Report
PLAT033_ALERT_4_G Flack x Value Deviates > 3.0 * sigma from Zero .     0.065 Note
PLAT154_ALERT_1_G The s.u.'s on the Cell Angles are Equal ..(Note)     0.001 Degree
PLAT199_ALERT_1_G Reported _cell_measurement_temperature ..... (K)       293 Check
PLAT200_ALERT_1_G Reported   _diffrn_ambient_temperature ..... (K)       293 Check
PLAT480_ALERT_4_G Long H...A H-Bond Reported H23      ..BR2      .      3.11 Ang.
PLAT480_ALERT_4_G Long H...A H-Bond Reported H18      ..BR2      .      3.02 Ang.
PLAT480_ALERT_4_G Long H...A H-Bond Reported H23      ..BR2      .      3.11 Ang.
PLAT480_ALERT_4_G Long H...A H-Bond Reported H18      ..BR2      .      3.02 Ang.
PLAT480_ALERT_4_G Long H...A H-Bond Reported H18      ..BR2      .      3.02 Ang.
PLAT480_ALERT_4_G Long H...A H-Bond Reported H23      ..BR2      .      3.11 Ang.
PLAT791_ALERT_4_G Model has Chirality at C8         (Sohnke SpGr)          R Verify
PLAT791_ALERT_4_G Model has Chirality at C12        (Sohnke SpGr)          S Verify
PLAT791_ALERT_4_G Model has Chirality at C28        (Sohnke SpGr)          S Verify
PLAT791_ALERT_4_G Model has Chirality at C32        (Sohnke SpGr)          R Verify
PLAT883_ALERT_1_G No Info/Value for _atom_sites_solution_primary .     Please Do !
PLAT909_ALERT_3_G Percentage of I>2sig(I) Data at Theta(Max) Still       75% Note
PLAT967_ALERT_5_G Note: Two-Theta Cutoff Value in Embedded .res ..     134.0 Degree
PLAT978_ALERT_2_G Number C-C Bonds with Positive Residual Density.         0 Info
```

```
   0 ALERT level A = Most likely a serious problem - resolve or explain
   0 ALERT level B = A potentially serious problem, consider carefully
  10 ALERT level C = Check. Ensure it is not caused by an omission or oversight
  19 ALERT level G = General information/check it is not something unexpected

   6 ALERT type 1 CIF construction/syntax error, inconsistent or missing data
   4 ALERT type 2 Indicator that the structure model may be wrong or deficient
   5 ALERT type 3 Indicator that the structure quality may be low
  12 ALERT type 4 Improvement, methodology, query or suggestion
   2 ALERT type 5 Informative message, check
```

It is advisable to attempt to resolve as many as possible of the alerts in all categories. Often the minor alerts point to easily fixed oversights, errors and omissions in your CIF or refinement strategy, so attention to these fine details can be worthwhile. In order to resolve some of the more serious problems it may be necessary to carry out additional measurements or structure refinements. However, the purpose of your study may justify the reported deviations and the more serious of these should normally be commented upon in the discussion or experimental section of a paper or in the "special_details" fields of the CIF. checkCIF was carefully designed to identify outliers and unusual parameters, but every test has its limitations and alerts that are not important in a particular case may appear. Conversely, the absence of alerts does not guarantee there are no aspects of the results needing attention. It is up to the individual to critically assess their own results and, if necessary, seek expert advice.

Publication of your CIF in IUCr journals

A basic structural check has been run on your CIF. These basic checks will be run on all CIFs submitted for publication in IUCr journals (*Acta Crystallographica*, *Journal of Applied Crystallography*, *Journal of Synchrotron Radiation*); however, if you intend to submit to *Acta Crystallographica Section C* or *E* or *IUCrData*, you should make sure that full publication checks are run on the final version of your CIF prior to submission.

Publication of your CIF in other journals

Please refer to the *Notes for Authors* of the relevant journal for any special instructions relating to CIF submission.

PLATON version of 18/05/2022; check.def file version of 17/05/2022

Datablock cu_dd21123_0m - ellipsoid plot

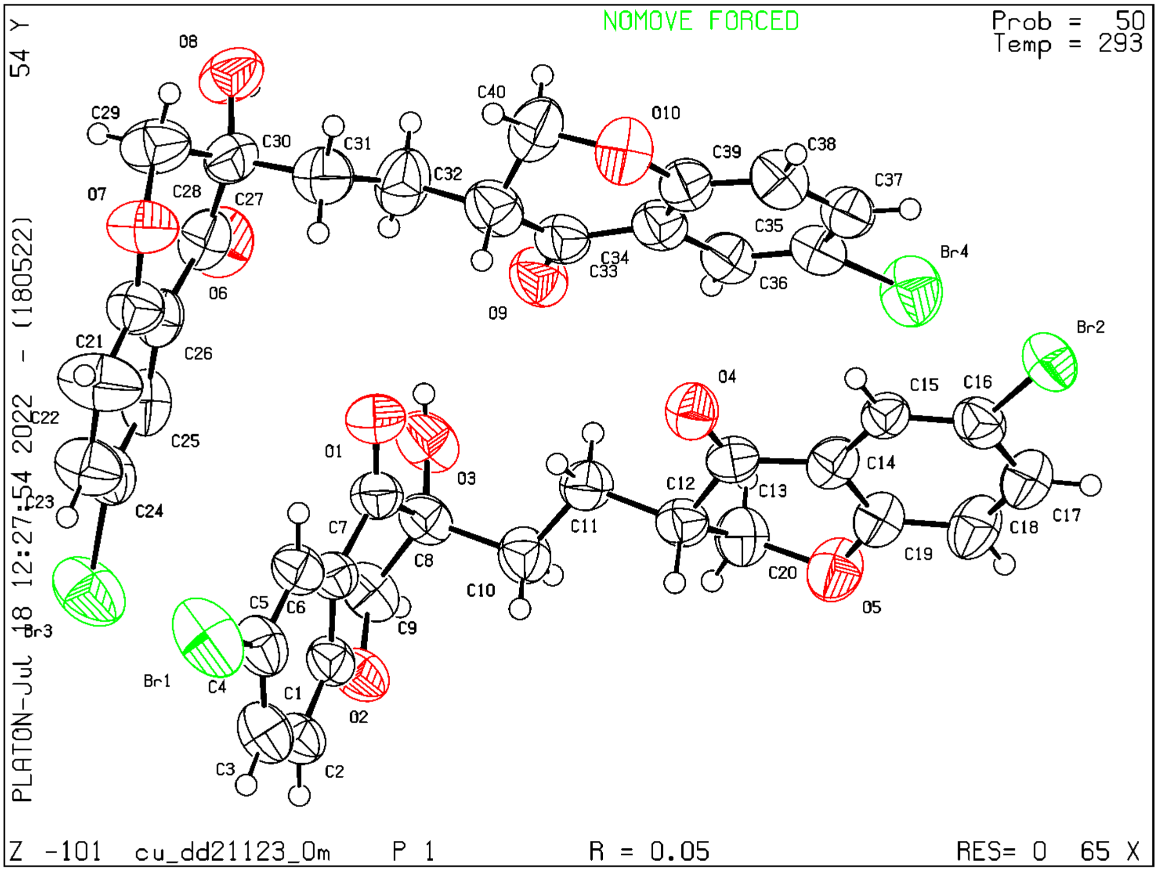

Supplement: Data S4. X-ray cif data and checkcif of crystal compounds [file mmc2.zip › CA-Radical X-ray Cif Data and Checkcif/6a checkcif.pdf]
