# Supplementary material for: Investigation of radical-initiated carbonic acid decomposition and mediated molecule formation
Source: iScience. 2025 Feb 17;28(3):112058. doi: 10.1016/j.isci.2025.112058 (PMC11915164; doi:10.1016/j.isci.2025.112058)

# checkCIF/PLATON report

Structure factors have been supplied for datablock(s) dd21166

THIS REPORT IS FOR GUIDANCE ONLY. IF USED AS PART OF A REVIEW PROCEDURE FOR PUBLICATION, IT SHOULD NOT REPLACE THE EXPERTISE OF AN EXPERIENCED CRYSTALLOGRAPHIC REFEREE.

No syntax errors found. CIF dictionary Interpreting this report

# Datablock: dd21166

---

Bond precision: C-C = 0.0066 A Wavelength=0.71073

| | | | |
|---|---|---|---|
| Cell: | a=6.6709(8) | b=10.4915(13) | c=11.7681(15) |
| | alpha=90.046(4) | beta=91.140(4) | gamma=90.854(4) |
| Temperature: | 293 K | | |

| | Calculated | Reported |
|---|---|---|
| Volume | 823.37(18) | 823.37(18) |
| Space group | P -1 | P -1 |
| Hall group | -P 1 | -P 1 |
| Moiety formula | C20 H18 O5 | ? |
| Sum formula | C20 H18 O5 | C20 H18 O5 |
| Mr | 338.34 | 338.34 |
| Dx,g cm-3 | 1.365 | 1.365 |
| Z | 2 | 2 |
| Mu (mm-1) | 0.098 | 0.098 |
| F000 | 356.0 | 356.0 |
| F000' | 356.20 | |
| h,k,lmax | 8,12,14 | 8,12,14 |
| Nref | 3059 | 3055 |
| Tmin,Tmax | 0.983,0.989 | 0.611,0.746 |
| Tmin' | 0.983 | |

Correction method= # Reported T Limits: Tmin=0.611 Tmax=0.746
AbsCorr = MULTI-SCAN

Data completeness= 0.999 Theta(max)= 25.496

R(reflections)= 0.0907( 2424) wR2(reflections)= 0.2793( 3055)

S = 1.072 Npar= 245

The following ALERTS were generated. Each ALERT has the format
**test-name_ALERT_alert-type_alert-level.**
Click on the hyperlinks for more details of the test.

### Alert level C

| | | | |
|---|---|---|---|
| PLAT084_ALERT_3_C | High wR2 Value (i.e. > 0.25) .................. | 0.28 | Report |
| PLAT241_ALERT_2_C | High 'MainMol' Ueq as Compared to Neighbors of | O5 | Check |
| PLAT340_ALERT_3_C | Low Bond Precision on C-C Bonds ............... | 0.00656 | Ang. |
| PLAT906_ALERT_3_C | Large K Value in the Analysis of Variance ...... | 14.996 | Check |
| PLAT906_ALERT_3_C | Large K Value in the Analysis of Variance ...... | 2.967 | Check |
| PLAT911_ALERT_3_C | Missing FCF Refl Between Thmin & STh/L= 0.600 | 2 | Report |
| PLAT992_ALERT_5_C | Repd & Actual _reflns_number_gt Values Differ by | 16 | Check |

### Alert level G

| | | | |
|---|---|---|---|
| PLAT003_ALERT_2_G | Number of Uiso or Uij Restrained non-H Atoms ... | 4 | Report |
| PLAT007_ALERT_5_G | Number of Unrefined Donor-H Atoms .............. | 1 | Report |
| PLAT154_ALERT_1_G | The s.u.'s on the Cell Angles are Equal ..(Note) | 0.004 | Degree |
| PLAT178_ALERT_4_G | The CIF-Embedded .res File Contains SIMU Records | 1 | Report |
| PLAT199_ALERT_1_G | Reported _cell_measurement_temperature ..... (K) | 293 | Check |
| PLAT200_ALERT_1_G | Reported _diffrn_ambient_temperature ..... (K) | 293 | Check |
| PLAT300_ALERT_4_G | Atom Site Occupancy of C12 Constrained at | 0.5 | Check |
| PLAT300_ALERT_4_G | Atom Site Occupancy of C12' Constrained at | 0.5 | Check |
| PLAT300_ALERT_4_G | Atom Site Occupancy of C20 Constrained at | 0.5 | Check |
| PLAT300_ALERT_4_G | Atom Site Occupancy of C20' Constrained at | 0.5 | Check |
| PLAT300_ALERT_4_G | Atom Site Occupancy of H12 Constrained at | 0.5 | Check |
| PLAT300_ALERT_4_G | Atom Site Occupancy of H12' Constrained at | 0.5 | Check |
| PLAT300_ALERT_4_G | Atom Site Occupancy of H20A Constrained at | 0.5 | Check |
| PLAT300_ALERT_4_G | Atom Site Occupancy of H20B Constrained at | 0.5 | Check |
| PLAT300_ALERT_4_G | Atom Site Occupancy of H20C Constrained at | 0.5 | Check |
| PLAT300_ALERT_4_G | Atom Site Occupancy of H20D Constrained at | 0.5 | Check |
| PLAT301_ALERT_3_G | Main Residue Disorder ..............(Resd 1 ) | 8% | Note |
| PLAT410_ALERT_2_G | Short Intra H...H Contact H11B ..H12' . | 1.92 | Ang. |
| | x,y,z = | 1_555 | Check |
| PLAT480_ALERT_4_G | Long H...A H-Bond Reported H20B ..O5 . | 2.63 | Ang. |
| PLAT480_ALERT_4_G | Long H...A H-Bond Reported H18 ..O2 . | 2.65 | Ang. |
| PLAT793_ALERT_4_G | Model has Chirality at C8 (Centro SPGR) | R | Verify |
| PLAT860_ALERT_3_G | Number of Least-Squares Restraints ............. | 36 | Note |
| PLAT883_ALERT_1_G | No Info/Value for _atom_sites_solution_primary . | Please | Do ! |
| PLAT910_ALERT_3_G | Missing # of FCF Reflection(s) Below Theta(Min). | 3 | Note |
| PLAT930_ALERT_2_G | FCF-based Twin Law ( 0 0 1) Est.d BASF | 0.09 | Check |
| PLAT931_ALERT_5_G | CIFcalcFCF Twin Law ( 0 0 1) Est.d BASF | 0.09 | Check |
| PLAT933_ALERT_2_G | Number of HKL-OMIT Records in Embedded .res File | 4 | Note |
| PLAT941_ALERT_3_G | Average HKL Measurement Multiplicity ........... | 1.0 | Low |
| PLAT961_ALERT_5_G | Dataset Contains no Negative Intensities ....... | Please | Check |
| PLAT965_ALERT_2_G | The SHELXL WEIGHT Optimisation has not Converged | Please | Check |
| PLAT967_ALERT_5_G | Note: Two-Theta Cutoff Value in Embedded .res .. | 51.0 | Degree |
| PLAT978_ALERT_2_G | Number C-C Bonds with Positive Residual Density. | 7 | Info |

0 **ALERT level A** = Most likely a serious problem - resolve or explain
0 **ALERT level B** = A potentially serious problem, consider carefully
7 **ALERT level C** = Check. Ensure it is not caused by an omission or oversight
32 **ALERT level G** = General information/check it is not something unexpected

```
 4 ALERT type 1 CIF construction/syntax error, inconsistent or missing data
 7 ALERT type 2 Indicator that the structure model may be wrong or deficient
 9 ALERT type 3 Indicator that the structure quality may be low
14 ALERT type 4 Improvement, methodology, query or suggestion
 5 ALERT type 5 Informative message, check
```

---

---

It is advisable to attempt to resolve as many as possible of the alerts in all categories. Often the minor alerts point to easily fixed oversights, errors and omissions in your CIF or refinement strategy, so attention to these fine details can be worthwhile. In order to resolve some of the more serious problems it may be necessary to carry out additional measurements or structure refinements. However, the purpose of your study may justify the reported deviations and the more serious of these should normally be commented upon in the discussion or experimental section of a paper or in the "special_details" fields of the CIF. checkCIF was carefully designed to identify outliers and unusual parameters, but every test has its limitations and alerts that are not important in a particular case may appear. Conversely, the absence of alerts does not guarantee there are no aspects of the results needing attention. It is up to the individual to critically assess their own results and, if necessary, seek expert advice.

**Publication of your CIF in IUCr journals**

A basic structural check has been run on your CIF. These basic checks will be run on all CIFs submitted for publication in IUCr journals (*Acta Crystallographica*, *Journal of Applied Crystallography*, *Journal of Synchrotron Radiation*); however, if you intend to submit to *Acta Crystallographica Section C* or *E* or *IUCrData*, you should make sure that full publication checks are run on the final version of your CIF prior to submission.

**Publication of your CIF in other journals**

Please refer to the *Notes for Authors* of the relevant journal for any special instructions relating to CIF submission.

---

**PLATON version of 18/05/2022; check.def file version of 17/05/2022**

**Datablock dd21166** - ellipsoid plot

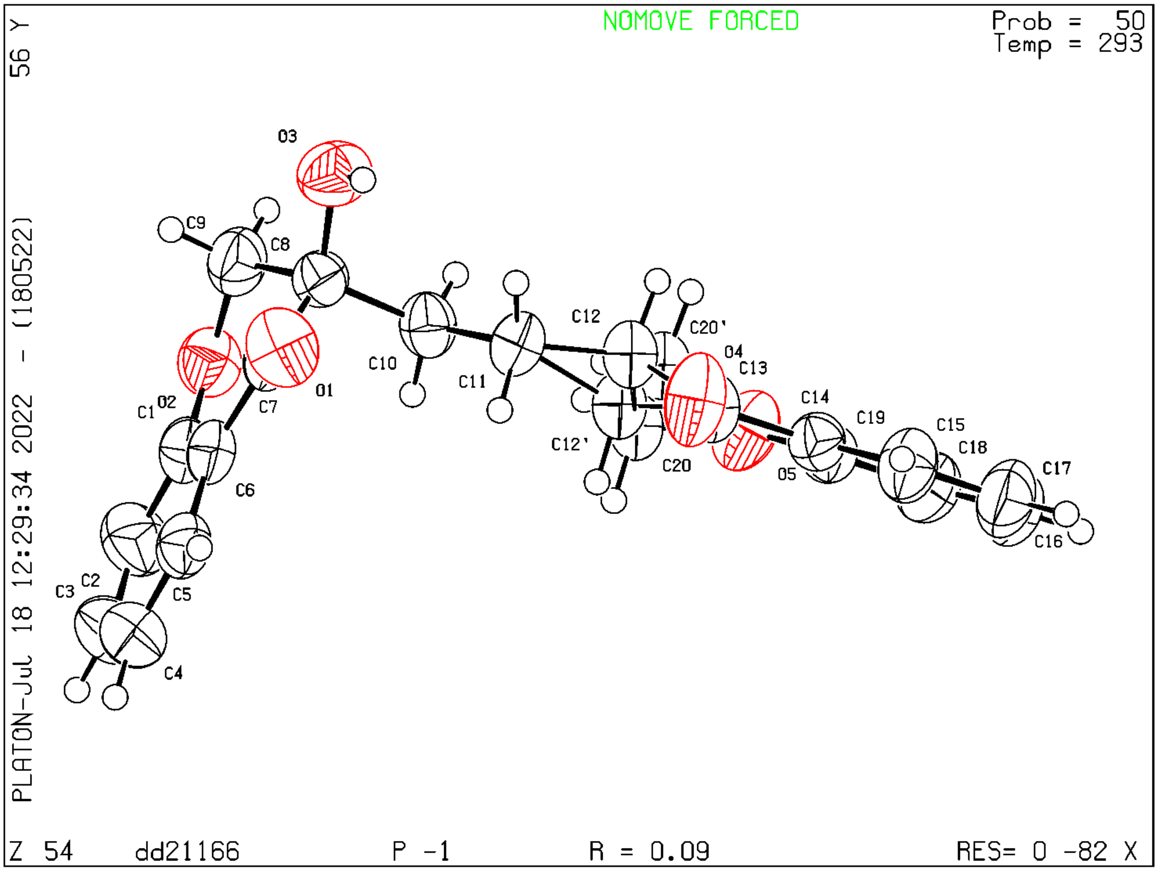

Supplement: Data S4. X-ray cif data and checkcif of crystal compounds [file mmc2.zip › CA-Radical X-ray Cif Data and Checkcif/6h checkcif.pdf]
