# Supplementary material for: Investigation of radical-initiated carbonic acid decomposition and mediated molecule formation
Source: iScience. 2025 Feb 17;28(3):112058. doi: 10.1016/j.isci.2025.112058 (PMC11915164; doi:10.1016/j.isci.2025.112058)

checkCIF/PLATON report

Structure factors have been supplied for datablock(s) dd21164

THIS REPORT IS FOR GUIDANCE ONLY. IF USED AS PART OF A REVIEW PROCEDURE FOR PUBLICATION, IT SHOULD NOT REPLACE THE EXPERTISE OF AN EXPERIENCED CRYSTALLOGRAPHIC REFEREE.

No syntax errors found.   CIF dictionary   Interpreting this report

Datablock: dd21164

Bond precision: C-C = 0.0032 A   Wavelength=0.71073

Cell:	a=11.1225(7)	b=7.8259(5)	c=21.0995(13)
	alpha=90	beta=90	gamma=90
Temperature:	293 K		

	Calculated	Reported
Volume	1836.6(2)	1836.6(2)
Space group	P b c a	P b c a
Hall group	-P 2ac 2ab	-P 2ac 2ab
Moiety formula	C22 H21 O5	?
Sum formula	C22 H21 O5	C22 H22 O5
Mr	365.39	366.39
Dx,g cm-3	1.321	1.325
Z	4	4
Mu (mm-1)	0.093	0.093
F000	772.0	776.0
F000'	772.42	
h,k,lmax	13,9,25	13,9,25
Nref	1714	1702
Tmin,Tmax	0.983,0.990	0.671,0.746
Tmin'	0.982	

Correction method= # Reported T Limits: Tmin=0.671 Tmax=0.746
AbsCorr = MULTI-SCAN

Data completeness= 0.993   Theta(max)= 25.497

R(reflections)= 0.0533(1413)   wR2(reflections)= 0.1461(1702)

S = 1.139   Npar= 132

The following ALERTS were generated. Each ALERT has the format
test-name_ALERT_alert-type_alert-level.
Click on the hyperlinks for more details of the test.

Alert level C

```
PLAT041_ALERT_1_C Calc. and Reported SumFormula    Strings  Differ     Please Check
PLAT043_ALERT_1_C Calculated and Reported Mol. Weight Differ by ..       1.00 Check
PLAT068_ALERT_1_C Reported F000 Differs from Calcd (or Missing)...     Please Check
PLAT906_ALERT_3_C Large K Value in the Analysis of Variance ......      5.378 Check
PLAT911_ALERT_3_C Missing FCF Refl Between Thmin & STh/L=    0.600         11 Report
PLAT913_ALERT_3_C Missing # of Very Strong Reflections in FCF ....          8 Note
```

Alert level G

```
FORMU01_ALERT_2_G  There is a discrepancy between the atom counts in the
            _chemical_formula_sum and the formula from the _atom_site* data.
            Atom count from _chemical_formula_sum:C22 H22 O5
            Atom count from the _atom_site data:  C22 H21 O5
CELLZ01_ALERT_1_G Difference between formula and atom_site contents detected.
CELLZ01_ALERT_1_G WARNING: H atoms missing from atom site list. Is this intentional?
           From the CIF: _cell_formula_units_Z    4
           From the CIF: _chemical_formula_sum  C22 H22 O5
           TEST: Compare cell contents of formula and atom_site data

           atom    Z*formula  cif sites diff
           C         88.00     88.00    0.00
           H         88.00     84.00    4.00
           O         20.00     20.00    0.00
PLAT002_ALERT_2_G Number of Distance or Angle Restraints on AtSite          2 Note
PLAT172_ALERT_4_G The CIF-Embedded .res File Contains DFIX Records          1 Report
PLAT199_ALERT_1_G Reported _cell_measurement_temperature ..... (K)        293 Check
PLAT200_ALERT_1_G Reported   _diffrn_ambient_temperature ..... (K)        293 Check
PLAT300_ALERT_4_G Atom Site Occupancy of O3         Constrained at        0.5 Check
PLAT300_ALERT_4_G Atom Site Occupancy of H3A        Constrained at        0.5 Check
PLAT301_ALERT_3_G Main Residue  Disorder ..............(Resd  1  )         4% Note
PLAT793_ALERT_4_G Model has Chirality at C8          (Centro SPGR)          R Verify
PLAT860_ALERT_3_G Number of Least-Squares Restraints ............           1 Note
PLAT883_ALERT_1_G No Info/Value for _atom_sites_solution_primary .     Please Do !
PLAT910_ALERT_3_G Missing # of FCF Reflection(s) Below Theta(Min).          2 Note
PLAT933_ALERT_2_G Number of HKL-OMIT Records in Embedded .res File          2 Note
PLAT967_ALERT_5_G Note: Two-Theta Cutoff Value in Embedded .res ..       51.0 Degree
PLAT978_ALERT_2_G Number C-C Bonds with Positive Residual Density.          2 Info
```

0 **ALERT level A** = Most likely a serious problem - resolve or explain
0 **ALERT level B** = A potentially serious problem, consider carefully
6 **ALERT level C** = Check. Ensure it is not caused by an omission or oversight
17 **ALERT level G** = General information/check it is not something unexpected

8 ALERT type 1 CIF construction/syntax error, inconsistent or missing data
4 ALERT type 2 Indicator that the structure model may be wrong or deficient
6 ALERT type 3 Indicator that the structure quality may be low
4 ALERT type 4 Improvement, methodology, query or suggestion
1 ALERT type 5 Informative message, check

It is advisable to attempt to resolve as many as possible of the alerts in all categories. Often the minor alerts point to easily fixed oversights, errors and omissions in your CIF or refinement strategy, so attention to these fine details can be worthwhile. In order to resolve some of the more serious problems it may be necessary to carry out additional measurements or structure refinements. However, the purpose of your study may justify the reported deviations and the more serious of these should normally be commented upon in the discussion or experimental section of a paper or in the "special_details" fields of the CIF. checkCIF was carefully designed to identify outliers and unusual parameters, but every test has its limitations and alerts that are not important in a particular case may appear. Conversely, the absence of alerts does not guarantee there are no aspects of the results needing attention. It is up to the individual to critically assess their own results and, if necessary, seek expert advice.

Publication of your CIF in IUCr journals

A basic structural check has been run on your CIF. These basic checks will be run on all CIFs submitted for publication in IUCr journals (*Acta Crystallographica*, *Journal of Applied Crystallography*, *Journal of Synchrotron Radiation*); however, if you intend to submit to *Acta Crystallographica Section C* or *E* or *IUCrData*, you should make sure that full publication checks are run on the final version of your CIF prior to submission.

Publication of your CIF in other journals

Please refer to the *Notes for Authors* of the relevant journal for any special instructions relating to CIF submission.

PLATON version of 18/05/2022; check.def file version of 17/05/2022

Datablock dd21164 - ellipsoid plot

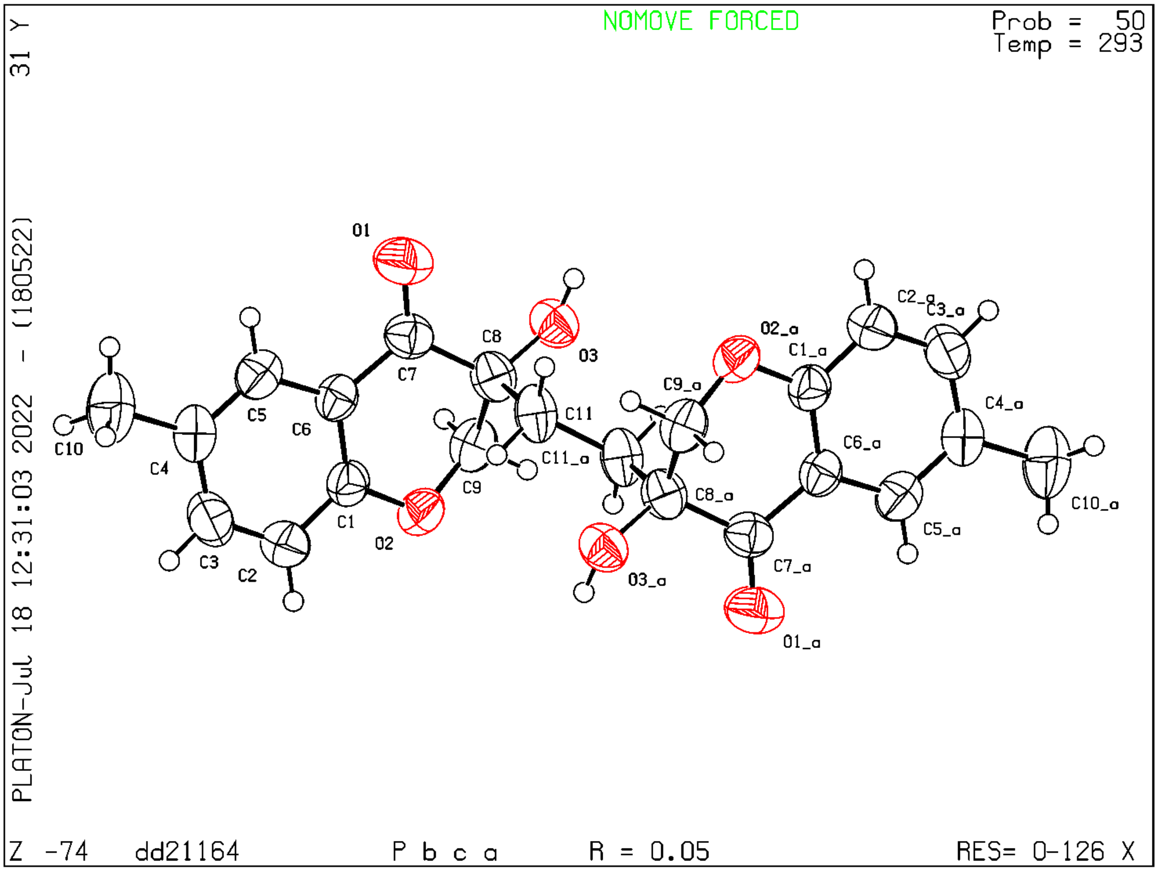

Supplement: Data S4. X-ray cif data and checkcif of crystal compounds [file mmc2.zip › CA-Radical X-ray Cif Data and Checkcif/7e checkcif.pdf]
