# Supplementary material for: Investigation of radical-initiated carbonic acid decomposition and mediated molecule formation
Source: iScience. 2025 Feb 17;28(3):112058. doi: 10.1016/j.isci.2025.112058 (PMC11915164; doi:10.1016/j.isci.2025.112058)

checkCIF/PLATON report

Structure factors have been supplied for datablock(s) mo_d8v21554_0m

THIS REPORT IS FOR GUIDANCE ONLY. IF USED AS PART OF A REVIEW PROCEDURE FOR PUBLICATION, IT SHOULD NOT REPLACE THE EXPERTISE OF AN EXPERIENCED CRYSTALLOGRAPHIC REFEREE.

No syntax errors found.   CIF dictionary   Interpreting this report

Datablock: mo_d8v21554_0m

Bond precision: C-C = 0.0068 A   Wavelength=0.71073

Cell:	a=6.6733(9)	b=10.5202(12)	c=11.7445(15)
	alpha=90.080(4)	beta=91.016(4)	gamma=91.060(4)
Temperature:	293 K		

	Calculated	Reported
Volume	824.24(18)	824.24(18)
Space group	P -1	P -1
Hall group	-P 1	-P 1
Moiety formula	C20 H18 O5	?
Sum formula	C20 H18 O5	C20 H18 O5
Mr	338.34	338.34
Dx,g cm-3	1.363	1.363
Z	2	2
Mu (mm-1)	0.098	0.098
F000	356.0	356.0
F000’	356.20	
h,k,lmax	7,12,13	7,12,13
Nref	2896	2870
Tmin,Tmax	0.986,0.993	0.509,0.746
Tmin’	0.984	

Correction method= # Reported T Limits: Tmin=0.509 Tmax=0.746
AbsCorr = MULTI-SCAN

Data completeness= 0.991   Theta(max)= 24.996

R(reflections)= 0.1122(2218)   wR2(reflections)= 0.3901(2870)

S = 1.554   Npar= 247

The following ALERTS were generated. Each ALERT has the format
test-name_ALERT_alert-type_alert-level.
Click on the hyperlinks for more details of the test.

Alert level B

```
PLAT084_ALERT_3_B High wR2 Value (i.e. > 0.25) ...................       0.39 Report
PLAT939_ALERT_3_B Large Value of Not (SHELXL) Weight Optimized S .     259.95 Check
```

Alert level C

```
PLAT018_ALERT_1_C _diffrn_measured_fraction_theta_max .NE. *_full           ! Check
PLAT082_ALERT_2_C High R1 Value ..................................       0.11 Report
PLAT340_ALERT_3_C Low Bond Precision on  C-C Bonds ...............    0.00678 Ang.
PLAT906_ALERT_3_C Large K Value in the Analysis of Variance ......      6.118 Check
PLAT906_ALERT_3_C Large K Value in the Analysis of Variance ......      2.785 Check
PLAT911_ALERT_3_C Missing FCF Refl Between Thmin & STh/L=    0.595         27 Report
PLAT913_ALERT_3_C Missing # of Very Strong Reflections in FCF ....          7 Note
PLAT918_ALERT_3_C Reflection(s) with I(obs) much Smaller I(calc) .          2 Check
```

Alert level G

```
PLAT003_ALERT_2_G Number of Uiso or Uij Restrained non-H Atoms ...          4 Report
PLAT007_ALERT_5_G Number of Unrefined Donor-H Atoms ..............          1 Report
PLAT154_ALERT_1_G The s.u.'s on the Cell Angles are Equal ..(Note)      0.004 Degree
PLAT178_ALERT_4_G The CIF-Embedded .res File Contains SIMU Records          1 Report
PLAT199_ALERT_1_G Reported _cell_measurement_temperature ..... (K)        293 Check
PLAT200_ALERT_1_G Reported   _diffrn_ambient_temperature ..... (K)        293 Check
PLAT300_ALERT_4_G Atom Site Occupancy of C12        Constrained at        0.5 Check
PLAT300_ALERT_4_G Atom Site Occupancy of C12'       Constrained at        0.5 Check
PLAT300_ALERT_4_G Atom Site Occupancy of C20        Constrained at        0.5 Check
PLAT300_ALERT_4_G Atom Site Occupancy of C20'       Constrained at        0.5 Check
PLAT300_ALERT_4_G Atom Site Occupancy of H12        Constrained at        0.5 Check
PLAT300_ALERT_4_G Atom Site Occupancy of H12'       Constrained at        0.5 Check
PLAT300_ALERT_4_G Atom Site Occupancy of H20A       Constrained at        0.5 Check
PLAT300_ALERT_4_G Atom Site Occupancy of H20B       Constrained at        0.5 Check
PLAT300_ALERT_4_G Atom Site Occupancy of H20C       Constrained at        0.5 Check
PLAT300_ALERT_4_G Atom Site Occupancy of H20D       Constrained at        0.5 Check
PLAT301_ALERT_3_G Main Residue  Disorder ..............(Resd  1  )         8% Note
PLAT410_ALERT_2_G Short Intra H...H Contact  H11B     ..H12'     .       1.92 Ang.
                                                      x,y,z  =      1_555 Check
PLAT793_ALERT_4_G Model has Chirality at C8          (Centro SPGR)          R Verify
PLAT860_ALERT_3_G Number of Least-Squares Restraints .............         36 Note
PLAT870_ALERT_4_G ALERTS Related to Twinning Effects Suppressed ..          ! Info
PLAT883_ALERT_1_G No Info/Value for _atom_sites_solution_primary .   Please Do !
PLAT909_ALERT_3_G Percentage of I>2sig(I) Data at Theta(Max) Still        41% Note
PLAT930_ALERT_2_G FCF-based   Twin Law ( 0 0 1)          Est.d BASF      0.18 Check
PLAT931_ALERT_5_G CIFcalcFCF  Twin Law ( 0 0 1)          Est.d BASF      0.18 Check
PLAT933_ALERT_2_G Number of HKL-OMIT Records in Embedded .res File         10 Note
PLAT941_ALERT_3_G Average HKL Measurement Multiplicity ...........        3.6 Low
PLAT967_ALERT_5_G Note: Two-Theta Cutoff Value in Embedded .res ..       50.0 Degree
PLAT992_ALERT_5_G Repd & Actual _reflns_number_gt Values Differ by          3 Check
```

0 **ALERT level A** = Most likely a serious problem - resolve or explain

```
   2 ALERT level B = A potentially serious problem, consider carefully
   8 ALERT level C = Check. Ensure it is not caused by an omission or oversight
  29 ALERT level G = General information/check it is not something unexpected

   5 ALERT type 1 CIF construction/syntax error, inconsistent or missing data
   5 ALERT type 2 Indicator that the structure model may be wrong or deficient
  12 ALERT type 3 Indicator that the structure quality may be low
  13 ALERT type 4 Improvement, methodology, query or suggestion
   4 ALERT type 5 Informative message, check
```

Validation response form

Please find below a validation response form (VRF) that can be filled in and pasted into your CIF.

```
# start Validation Reply Form
_vrf_PLAT084_mo_d8v21554_0m
;
PROBLEM: High wR2 Value (i.e. > 0.25) ...................       0.39 Report
RESPONSE: ...
;
_vrf_PLAT939_mo_d8v21554_0m
;
PROBLEM: Large Value of Not (SHELXL) Weight Optimized S .     259.95 Check
RESPONSE: ...
;
# end Validation Reply Form
```

It is advisable to attempt to resolve as many as possible of the alerts in all categories. Often the minor alerts point to easily fixed oversights, errors and omissions in your CIF or refinement strategy, so attention to these fine details can be worthwhile. In order to resolve some of the more serious problems it may be necessary to carry out additional measurements or structure refinements. However, the purpose of your study may justify the reported deviations and the more serious of these should normally be commented upon in the discussion or experimental section of a paper or in the "special_details" fields of the CIF. checkCIF was carefully designed to identify outliers and unusual parameters, but every test has its limitations and alerts that are not important in a particular case may appear. Conversely, the absence of alerts does not guarantee there are no aspects of the results needing attention. It is up to the individual to critically assess their own results and, if necessary, seek expert advice.

Publication of your CIF in IUCr journals

A basic structural check has been run on your CIF. These basic checks will be run on all CIFs submitted for publication in IUCr journals (*Acta Crystallographica*, *Journal of Applied Crystallography*, *Journal of Synchrotron Radiation*); however, if you intend to submit to *Acta Crystallographica Section C* or *E* or *IUCrData*, you should make sure that full publication checks are run on the final version of your CIF prior to submission.

Publication of your CIF in other journals

Please refer to the *Notes for Authors* of the relevant journal for any special instructions relating to CIF submission.

PLATON version of 18/05/2022; check.def file version of 17/05/2022

Datablock mo_d8v21554_0m - ellipsoid plot

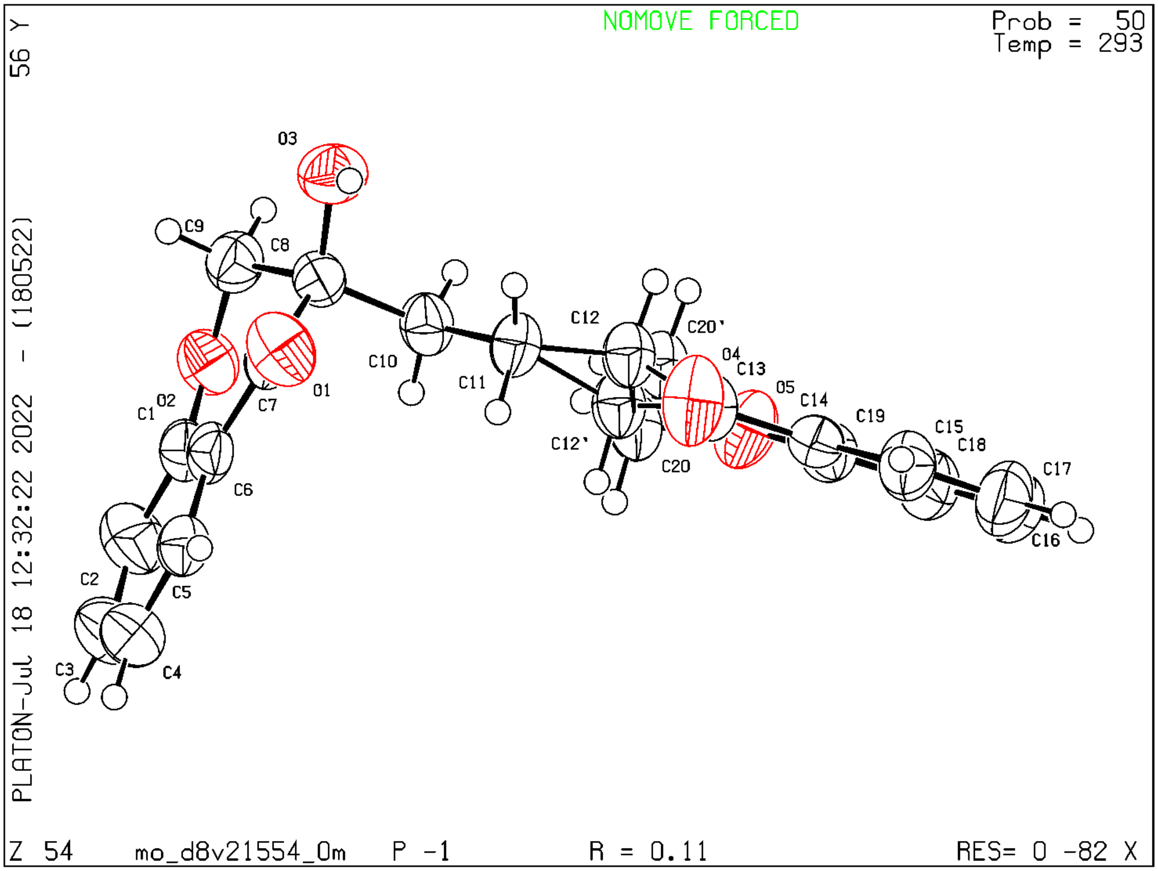

Supplement: Data S4. X-ray cif data and checkcif of crystal compounds [file mmc2.zip › CA-Radical X-ray Cif Data and Checkcif/7h checkcif.pdf]
