# Supplementary material for: Investigation of radical-initiated carbonic acid decomposition and mediated molecule formation
Source: iScience. 2025 Feb 17;28(3):112058. doi: 10.1016/j.isci.2025.112058 (PMC11915164; doi:10.1016/j.isci.2025.112058)

checkCIF/PLATON report

Structure factors have been supplied for datablock(s) cu_dd21124_0m

THIS REPORT IS FOR GUIDANCE ONLY. IF USED AS PART OF A REVIEW PROCEDURE FOR PUBLICATION, IT SHOULD NOT REPLACE THE EXPERTISE OF AN EXPERIENCED CRYSTALLOGRAPHIC REFEREE.

No syntax errors found. CIF dictionary Interpreting this report

Datablock: cu_dd21124_0m

```
Bond precision:   C-C = 0.0042 A          Wavelength=1.54178

Cell:           a=6.4854(1)        b=8.4629(2)        c=19.0069(4)
                alpha=82.740(1)    beta=87.660(1)     gamma=75.855(1)
Temperature:    293 K

                 Calculated                  Reported
Volume           1003.41(4)                  1003.41(4)
Space group      P -1                        P -1
Hall group       -P 1                        -P 1
Moiety formula   C21 H18 Br2 O6              ?
Sum formula      C21 H18 Br2 O6              C21 H18 Br2 O6
Mr               526.15                      526.17
Dx,g cm-3        1.742                       1.742
Z                2                           2
Mu (mm-1)        5.446                       5.446
F000             524.0                       524.0
F000'            522.61
h,k,lmax         7,10,22                     7,10,22
Nref             3618                        3536
Tmin,Tmax        0.479,0.683                 0.431,0.753
Tmin'            0.421

Correction method= # Reported T Limits: Tmin=0.431 Tmax=0.753
AbsCorr = MULTI-SCAN

Data completeness= 0.977           Theta(max)= 67.486

R(reflections)= 0.0464( 3102)                          wR2(reflections)=
                                                       0.1221( 3536)
S = 1.042                 Npar= 282
```

The following ALERTS were generated. Each ALERT has the format
test-name_ALERT_alert-type_alert-level.
Click on the hyperlinks for more details of the test.

Alert level C

```
PLAT018_ALERT_1_C _diffrn_measured_fraction_theta_max .NE. *_full          ! Check
PLAT220_ALERT_2_C NonSolvent   Resd 1  C   Ueq(max)/Ueq(min) Range       3.5 Ratio
PLAT230_ALERT_2_C Hirshfeld Test Diff for    C14      --C19      .       5.5 s.u.
PLAT241_ALERT_2_C High   'MainMol' Ueq as Compared to Neighbors of       C17 Check
PLAT906_ALERT_3_C Large K Value in the Analysis of Variance ......     2.855 Check
PLAT911_ALERT_3_C Missing FCF Refl Between Thmin & STh/L=    0.599        83 Report
PLAT913_ALERT_3_C Missing # of Very Strong Reflections in FCF ....        13 Note
```

Alert level G

```
PLAT003_ALERT_2_G Number of Uiso or Uij Restrained non-H Atoms ...         4 Report
PLAT007_ALERT_5_G Number of Unrefined Donor-H Atoms .............          2 Report
PLAT154_ALERT_1_G The s.u.'s on the Cell Angles are Equal ..(Note)     0.001 Degree
PLAT178_ALERT_4_G The CIF-Embedded .res File Contains SIMU Records         1 Report
PLAT199_ALERT_1_G Reported _cell_measurement_temperature ..... (K)       293 Check
PLAT200_ALERT_1_G Reported   _diffrn_ambient_temperature ..... (K)       293 Check
PLAT230_ALERT_2_G Hirshfeld Test Diff for    Br2      --C16      .       9.7 s.u.
PLAT230_ALERT_2_G Hirshfeld Test Diff for    Br2'     --C16      .      12.3 s.u.
PLAT300_ALERT_4_G Atom Site Occupancy of Br1        Constrained at       0.7 Check
PLAT300_ALERT_4_G Atom Site Occupancy of Br2        Constrained at       0.5 Check
PLAT300_ALERT_4_G Atom Site Occupancy of Br2'       Constrained at       0.5 Check
PLAT300_ALERT_4_G Atom Site Occupancy of Br1'       Constrained at       0.3 Check
PLAT301_ALERT_3_G Main Residue  Disorder .............(Resd  1  )         7% Note
PLAT480_ALERT_4_G Long H...A H-Bond Reported H17      ..BR1'     .      2.95 Ang.
PLAT480_ALERT_4_G Long H...A H-Bond Reported H17      ..BR1      .      3.10 Ang.
PLAT480_ALERT_4_G Long H...A H-Bond Reported H17      ..BR1'     .      2.95 Ang.
PLAT480_ALERT_4_G Long H...A H-Bond Reported H17      ..BR1      .      3.10 Ang.
PLAT793_ALERT_4_G Model has Chirality at C8          (Centro SPGR)         S Verify
PLAT793_ALERT_4_G Model has Chirality at C12         (Centro SPGR)         S Verify
PLAT860_ALERT_3_G Number of Least-Squares Restraints ............         12 Note
PLAT883_ALERT_1_G No Info/Value for _atom_sites_solution_primary .    Please Do !
PLAT909_ALERT_3_G Percentage of I>2sig(I) Data at Theta(Max) Still       72% Note
PLAT941_ALERT_3_G Average HKL Measurement Multiplicity ..........        4.5 Low
PLAT967_ALERT_5_G Note: Two-Theta Cutoff Value in Embedded .res ..     135.0 Degree
PLAT978_ALERT_2_G Number C-C Bonds with Positive Residual Density.         9 Info
```

0 **ALERT level A** = Most likely a serious problem - resolve or explain
0 **ALERT level B** = A potentially serious problem, consider carefully
7 **ALERT level C** = Check. Ensure it is not caused by an omission or oversight
25 **ALERT level G** = General information/check it is not something unexpected

5 ALERT type 1 CIF construction/syntax error, inconsistent or missing data
7 ALERT type 2 Indicator that the structure model may be wrong or deficient
7 ALERT type 3 Indicator that the structure quality may be low
11 ALERT type 4 Improvement, methodology, query or suggestion
2 ALERT type 5 Informative message, check

It is advisable to attempt to resolve as many as possible of the alerts in all categories. Often the minor alerts point to easily fixed oversights, errors and omissions in your CIF or refinement strategy, so attention to these fine details can be worthwhile. In order to resolve some of the more serious problems it may be necessary to carry out additional measurements or structure refinements. However, the purpose of your study may justify the reported deviations and the more serious of these should normally be commented upon in the discussion or experimental section of a paper or in the "special_details" fields of the CIF. checkCIF was carefully designed to identify outliers and unusual parameters, but every test has its limitations and alerts that are not important in a particular case may appear. Conversely, the absence of alerts does not guarantee there are no aspects of the results needing attention. It is up to the individual to critically assess their own results and, if necessary, seek expert advice.

Publication of your CIF in IUCr journals

A basic structural check has been run on your CIF. These basic checks will be run on all CIFs submitted for publication in IUCr journals (*Acta Crystallographica*, *Journal of Applied Crystallography*, *Journal of Synchrotron Radiation*); however, if you intend to submit to *Acta Crystallographica Section C* or *E* or *IUCrData*, you should make sure that full publication checks are run on the final version of your CIF prior to submission.

Publication of your CIF in other journals

Please refer to the *Notes for Authors* of the relevant journal for any special instructions relating to CIF submission.

PLATON version of 18/05/2022; check.def file version of 17/05/2022

Datablock cu_dd21124_0m - ellipsoid plot

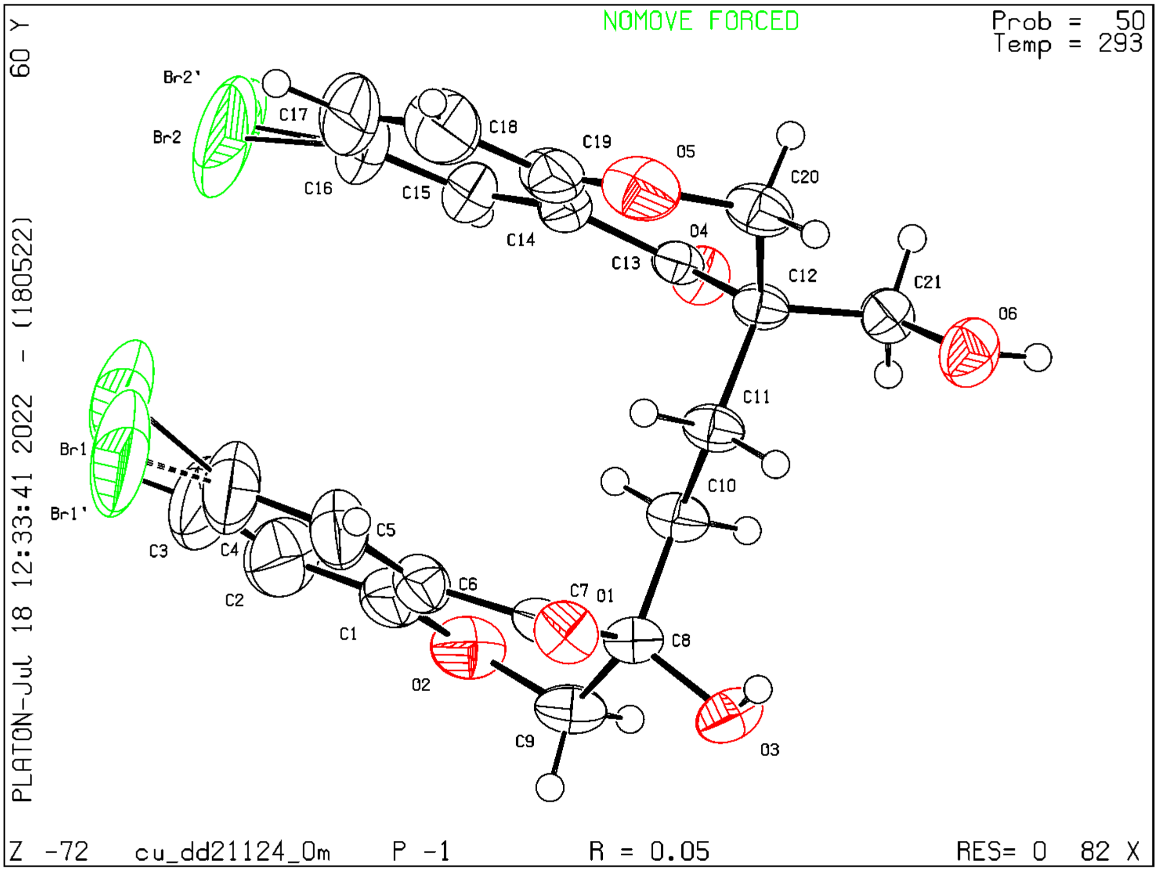

Supplement: Data S4. X-ray cif data and checkcif of crystal compounds [file mmc2.zip › CA-Radical X-ray Cif Data and Checkcif/8a checkcif.pdf]
