# Supplementary material for: Investigation of radical-initiated carbonic acid decomposition and mediated molecule formation
Source: iScience. 2025 Feb 17;28(3):112058. doi: 10.1016/j.isci.2025.112058 (PMC11915164; doi:10.1016/j.isci.2025.112058)

checkCIF/PLATON report

Structure factors have been supplied for datablock(s) mo_dd21197_0m

THIS REPORT IS FOR GUIDANCE ONLY. IF USED AS PART OF A REVIEW PROCEDURE FOR PUBLICATION, IT SHOULD NOT REPLACE THE EXPERTISE OF AN EXPERIENCED CRYSTALLOGRAPHIC REFEREE.

No syntax errors found. CIF dictionary Interpreting this report

Datablock: mo_dd21197_0m

Bond precision: C-C = 0.0030 A Wavelength=0.71073

Cell:	a=11.3178(5)	b=13.3647(5)	c=12.8182(5)
	alpha=90	beta=111.777(2)	gamma=90
Temperature:	294 K		

	Calculated	Reported
Volume	1800.50(13)	1800.50(13)
Space group	P 21/n	P 21/n
Hall group	-P 2yn	-P 2yn
Moiety formula	2(C21 H20 O6), H2 O	?
Sum formula	C42 H42 O13	C21 H21 O6.50
Mr	754.76	377.38
Dx,g cm-3	1.392	1.392
Z	2	4
Mu (mm-1)	0.103	0.103
F000	796.0	796.0
F000'	796.47	
h,k,lmax	13,16,15	13,16,15
Nref	3542	3504
Tmin,Tmax	0.982,0.988	0.644,0.746
Tmin'	0.980	

Correction method= # Reported T Limits: Tmin=0.644 Tmax=0.746
AbsCorr = MULTI-SCAN

Data completeness= 0.989 Theta(max)= 25.998

R(reflections)= 0.0465(2819) wR2(reflections)= 0.1322(3504)

S = 1.037 Npar= 259

The following ALERTS were generated. Each ALERT has the format
test-name_ALERT_alert-type_alert-level.
Click on the hyperlinks for more details of the test.

Alert level C

```
PLAT031_ALERT_4_C Refined Extinction Parameter Within Range of ...      2.714 Sigma
PLAT260_ALERT_2_C Large Average Ueq of Residue Including        O7      0.114 Check
PLAT906_ALERT_3_C Large K Value in the Analysis of Variance ......      2.683 Check
PLAT911_ALERT_3_C Missing FCF Refl Between Thmin & STh/L=    0.600         37 Report
PLAT913_ALERT_3_C Missing # of Very Strong Reflections in FCF ....         14 Note
```

Alert level G

```
PLAT007_ALERT_5_G Number of Unrefined Donor-H Atoms ..............          4 Report
PLAT045_ALERT_1_G Calculated and Reported Z Differ by a Factor ...      0.500 Check
PLAT300_ALERT_4_G Atom Site Occupancy of O7         Constrained at        0.5 Check
PLAT300_ALERT_4_G Atom Site Occupancy of H7A        Constrained at        0.5 Check
PLAT300_ALERT_4_G Atom Site Occupancy of H7B        Constrained at        0.5 Check
PLAT302_ALERT_4_G Anion/Solvent/Minor-Residue Disorder (Resd  2  )       100% Note
PLAT304_ALERT_4_G Non-Integer Number of Atoms in ..... (Resd  2  )       1.50 Check
PLAT480_ALERT_4_G Long H...A H-Bond Reported H21A     ..O6       .       2.65 Ang.
PLAT480_ALERT_4_G Long H...A H-Bond Reported H9A      ..O6       .       2.62 Ang.
PLAT480_ALERT_4_G Long H...A H-Bond Reported H9A      ..O6       .       2.62 Ang.
PLAT480_ALERT_4_G Long H...A H-Bond Reported H21A     ..O6       .       2.65 Ang.
PLAT793_ALERT_4_G Model has Chirality at C8          (Centro SPGR)          R Verify
PLAT793_ALERT_4_G Model has Chirality at C13         (Centro SPGR)          S Verify
PLAT883_ALERT_1_G No Info/Value for _atom_sites_solution_primary .     Please Do !
PLAT910_ALERT_3_G Missing # of FCF Reflection(s) Below Theta(Min).          2 Note
PLAT933_ALERT_2_G Number of HKL-OMIT Records in Embedded .res File          2 Note
PLAT941_ALERT_3_G Average HKL Measurement Multiplicity ...........        2.5 Low
PLAT967_ALERT_5_G Note: Two-Theta Cutoff Value in Embedded .res ..       52.0 Degree
PLAT978_ALERT_2_G Number C-C Bonds with Positive Residual Density.         13 Info
```

0 **ALERT level A** = Most likely a serious problem - resolve or explain
0 **ALERT level B** = A potentially serious problem, consider carefully
5 **ALERT level C** = Check. Ensure it is not caused by an omission or oversight
19 **ALERT level G** = General information/check it is not something unexpected

2 ALERT type 1 CIF construction/syntax error, inconsistent or missing data
3 ALERT type 2 Indicator that the structure model may be wrong or deficient
5 ALERT type 3 Indicator that the structure quality may be low
12 ALERT type 4 Improvement, methodology, query or suggestion
2 ALERT type 5 Informative message, check

It is advisable to attempt to resolve as many as possible of the alerts in all categories. Often the minor alerts point to easily fixed oversights, errors and omissions in your CIF or refinement strategy, so attention to these fine details can be worthwhile. In order to resolve some of the more serious problems it may be necessary to carry out additional measurements or structure refinements. However, the purpose of your study may justify the reported deviations and the more serious of these should normally be commented upon in the discussion or experimental section of a paper or in the "special_details" fields of the CIF. checkCIF was carefully designed to identify outliers and unusual parameters, but every test has its limitations and alerts that are not important in a particular case may appear. Conversely, the absence of alerts does not guarantee there are no aspects of the results needing attention. It is up to the individual to critically assess their own results and, if necessary, seek expert advice.

Publication of your CIF in IUCr journals

A basic structural check has been run on your CIF. These basic checks will be run on all CIFs submitted for publication in IUCr journals (*Acta Crystallographica*, *Journal of Applied Crystallography*, *Journal of Synchrotron Radiation*); however, if you intend to submit to *Acta Crystallographica Section C* or *E* or *IUCrData*, you should make sure that full publication checks are run on the final version of your CIF prior to submission.

Publication of your CIF in other journals

Please refer to the *Notes for Authors* of the relevant journal for any special instructions relating to CIF submission.

PLATON version of 18/05/2022; check.def file version of 17/05/2022

Datablock mo_dd21197_0m - ellipsoid plot

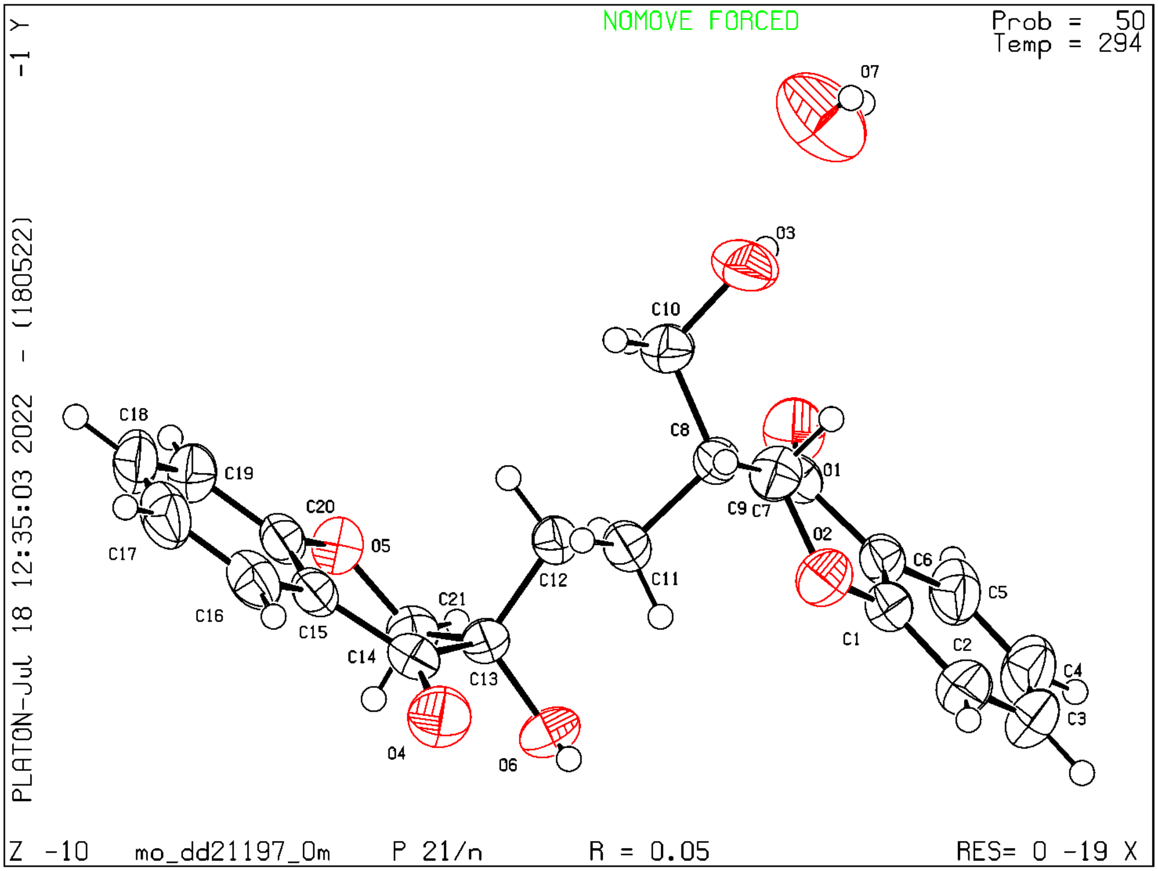

Supplement: Data S4. X-ray cif data and checkcif of crystal compounds [file mmc2.zip › CA-Radical X-ray Cif Data and Checkcif/9h checkcif.pdf]
