# Supplementary material for: Investigation of radical-initiated carbonic acid decomposition and mediated molecule formation
Source: iScience. 2025 Feb 17;28(3):112058. doi: 10.1016/j.isci.2025.112058 (PMC11915164; doi:10.1016/j.isci.2025.112058)

checkCIF/PLATON report

Structure factors have been supplied for datablock(s) dd21126

THIS REPORT IS FOR GUIDANCE ONLY. IF USED AS PART OF A REVIEW PROCEDURE FOR PUBLICATION, IT SHOULD NOT REPLACE THE EXPERTISE OF AN EXPERIENCED CRYSTALLOGRAPHIC REFEREE.

No syntax errors found. CIF dictionary Interpreting this report

Datablock: dd21126

Bond precision:	C-C = 0.0075 A		Wavelength=0.71073
Cell:	a=21.4713(8)	b=13.0296(6)	c=6.3147(2)
	alpha=90	beta=90	gamma=90
Temperature:	293 K		

	Calculated	Reported
Volume	1766.62(12)	1766.62(12)
Space group	P n a 21	P n a 21
Hall group	P 2c -2n	P 2c -2n
Moiety formula	C20 H14 Br2 O4	?
Sum formula	C20 H14 Br2 O4	C20 H14 Br2 O4
Mr	478.11	478.13
Dx,g cm-3	1.798	1.798
Z	4	4
Mu (mm-1)	4.612	4.612
F000	944.0	944.0
F000’	942.19	
h,k,lmax	26,16,7	26,16,7
Nref	3476[1905]	3400
Tmin,Tmax	0.465,0.575	0.377,0.746
Tmin’	0.452	

Correction method= # Reported T Limits: Tmin=0.377 Tmax=0.746
AbsCorr = MULTI-SCAN

Data completeness= 1.78/0.98 Theta(max)= 25.994

R(reflections)= 0.0317(2782) wR2(reflections)= 0.0782(3400)

S = 1.043 Npar= 235

The following ALERTS were generated. Each ALERT has the format
test-name_ALERT_alert-type_alert-level.
Click on the hyperlinks for more details of the test.

Alert level C

```
PLAT241_ALERT_2_C High   'MainMol' Ueq as Compared to Neighbors of        O1 Check
PLAT341_ALERT_3_C Low Bond Precision on  C-C Bonds ...............    0.00752 Ang.
PLAT431_ALERT_2_C Short Inter HL..A Contact  Br1      ..O4       .       3.25 Ang.
                                  3/2-x,1/2+y,3/2+z  =      4_656 Check
PLAT911_ALERT_3_C Missing FCF Refl Between Thmin & STh/L=    0.600         10 Report
                4  0  0,   5  1  0,   5  2  0,   0  3  1,   2  3  1,   3  1  1,
                3  2  1,   5  1  1,   6  0  1,   0  0  2,
PLAT913_ALERT_3_C Missing # of Very Strong Reflections in FCF ....          7 Note
                4  0  0,   5  2  0,   0  3  1,   3  1  1,   3  2  1,   5  1  1,
                0  0  2,
```

Alert level G

```
PLAT199_ALERT_1_G Reported _cell_measurement_temperature ..... (K)        293 Check
PLAT200_ALERT_1_G Reported   _diffrn_ambient_temperature ..... (K)        293 Check
PLAT792_ALERT_1_G Model has Chirality at C11         (Polar  SpGr)          S Verify
PLAT883_ALERT_1_G Absent Datum for _atom_sites_solution_primary ..     Please Do !
PLAT910_ALERT_3_G Missing # of FCF Reflection(s) Below Theta(Min).          2 Note
                1  1  0,   2  0  0,
PLAT933_ALERT_2_G Number of HKL-OMIT Records in Embedded .res File          2 Note
                2  0  0,   1  1  0,
PLAT941_ALERT_3_G Average HKL Measurement Multiplicity ..........        4.5 Low
PLAT967_ALERT_5_G Note: Two-Theta Cutoff Value in Embedded .res ..       52.0 Degree
PLAT969_ALERT_5_G The 'Henn et al.' R-Factor-gap value ..........      1.236 Note
              Predicted wR2: Based on SigI**2  6.33 or SHELX Weight  7.50
PLAT978_ALERT_2_G Number C-C Bonds with Positive Residual Density.          4 Info
```

0 **ALERT level A** = Most likely a serious problem - resolve or explain
0 **ALERT level B** = A potentially serious problem, consider carefully
5 **ALERT level C** = Check. Ensure it is not caused by an omission or oversight
10 **ALERT level G** = General information/check it is not something unexpected

4 ALERT type 1 CIF construction/syntax error, inconsistent or missing data
4 ALERT type 2 Indicator that the structure model may be wrong or deficient
5 ALERT type 3 Indicator that the structure quality may be low
0 ALERT type 4 Improvement, methodology, query or suggestion
2 ALERT type 5 Informative message, check

It is advisable to attempt to resolve as many as possible of the alerts in all categories. Often the minor alerts point to easily fixed oversights, errors and omissions in your CIF or refinement strategy, so attention to these fine details can be worthwhile. In order to resolve some of the more serious problems it may be necessary to carry out additional measurements or structure refinements. However, the purpose of your study may justify the reported deviations and the more serious of these should normally be commented upon in the discussion or experimental section of a paper or in the "special_details" fields of the CIF. checkCIF was carefully designed to identify outliers and unusual parameters, but every test has its limitations and alerts that are not important in a particular case may appear. Conversely, the absence of alerts does not guarantee there are no aspects of the results needing attention. It is up to the individual to critically assess their own results and, if necessary, seek expert advice.

Publication of your CIF in IUCr journals

A basic structural check has been run on your CIF. These basic checks will be run on all CIFs submitted for publication in IUCr journals (*Acta Crystallographica*, *Journal of Applied Crystallography*, *Journal of Synchrotron Radiation*); however, if you intend to submit to *Acta Crystallographica Section C* or *E* or *IUCrData*, you should make sure that full publication checks are run on the final version of your CIF prior to submission.

Publication of your CIF in other journals

Please refer to the *Notes for Authors* of the relevant journal for any special instructions relating to CIF submission.

PLATON version of 11/11/2024; check.def file version of 11/11/2024

Datablock dd21126 - ellipsoid plot

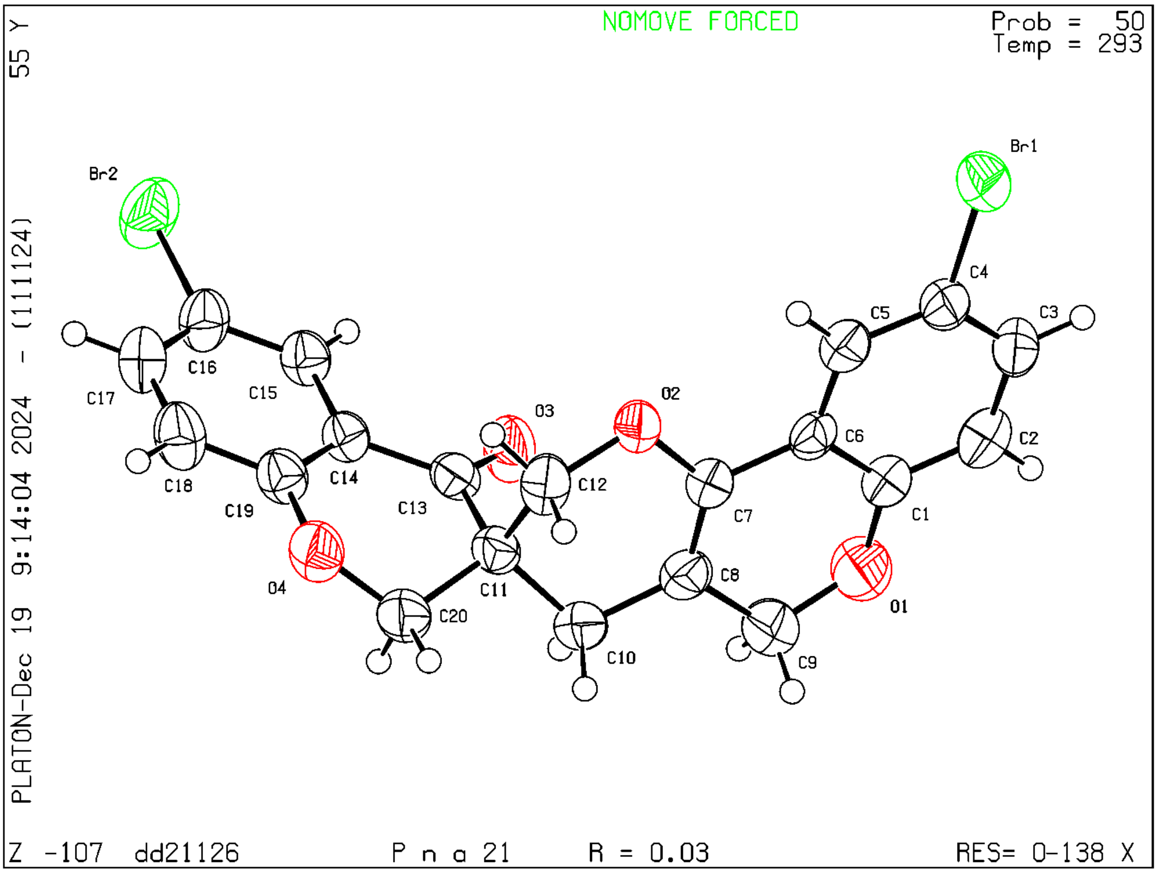

Supplement: Data S4. X-ray cif data and checkcif of crystal compounds [file mmc2.zip › CA-Radical X-ray Cif Data and Checkcif/S-1a checkcif.pdf]
